# A next-generation sequencing study on mechanisms by which restraint and social instability stresses of male mice alter offspring anxiety-like behavior

**DOI:** 10.1038/s41598-021-87060-x

**Published:** 2021-04-12

**Authors:** Qiao-Qiao Kong, Xiao-Dan Tian, Jia Wang, Hong-Jie Yuan, Shu-Fen Ning, Ming-Jiu Luo, Jing-He Tan

**Affiliations:** 1 Shandong Provincial Key Laboratory of Animal Biotechnology and Disease Control and Prevention, College of Animal Science and Veterinary Medicine, Shandong Agricultural University, Tai’an City, 271018 Shandong Province People’s Republic of China; 2 Tai’an City Central Hospital, Tai’an City, People’s Republic of China

**Keywords:** Neuroscience, Psychology

## Abstract

Pathophysiological mechanisms for depression/anxiety are largely unknown. Evidence for transgenerational transmission of acquired epigenetic marks remains limited. We bred unstressed (US) female mice with adolescently restraint-stressed (RS), social instability-stressed (SI) or US males to produce RS, SI and control F1 offspring, respectively. Compared to controls, while paternal RS decreased anxiety-like behavior (ALB) in both female and male offspring, paternal SI increased ALB only in female offspring. Next-generation sequencing and bioinformatics using RS and SI female offspring identified 5 candidate anxiety-transmitting (CAT) genes; each showed a consistent pattern of DNA methylation from F0 spermatozoa through F1 blastocysts to fetal and adult hippocampi. Further analyses validated 4 CAT genes, demonstrated that paternal SI caused ALB differences between male and female offspring through modifying the CAT genes, and indicated a strong correlation between inflammation and ALB pathogenesis and an important function for intronic DNA methylation in regulating ALB-related genes. In conclusion, this study identified important CAT genes and suggested the possibility that stresses on males might alter offspring’s ALB by modifying sperm DNA methylation.

## Introduction

Depression and anxiety disorders are among the major public health problems^1–3^, but the mechanisms for these diseases are largely unknown. Although studies using rodents indicate that maternal or paternal stresses during the prenatal^4^, neonatal^5^, adolescent^6,7^ and adult^7,8^ periods can cause psychological alterations in progeny, the underlying mechanisms are largely unclear. These mechanisms are well worth exploring because of their significant implications for evolutionary biology and disease etiology^9,10^.

Epigenetic modification in germ cells has been proposed as a mechanism for parental experiences to shape offspring neurodevelopment^11,12^. Studies using rodents indicated that the effects of paternal stress experiences^8,13^ and elevated glucocorticoid exposure^14^ could be transmitted to offspring through epigenetic marks. However, direct evidence for epigenetic reprogramming of the germline and its transgenerational transmission is far from being sufficient. For example, among studies that observed epigenetic alterations in spermatozoa, only a few analyzed noncoding RNA profiling^8,14^ or single gene DNA methylation^15^ following stress treatment of the sires; next-generation sequencing and systematic studies on the routes by which epigenetic signals are delivered from gametes through embryos to adult organs are lacking.

Both mouse models of restraint stress (RS) and social instability (SI) have been used to study the mechanisms by which stressors applied at different life stages cause mental alterations in the current or subsequent generations^16^. However, while Saavedra-Rodríguez and Feig^6^ reported that SI during adolescence of sires led to increased anxiety-like behavior (ALB) in F1 mouse offspring, others observed that paternal RS^7^ and other stresses^8,17^ during newborn, puberty or adulthood resulted in reduced ALB in mouse offspring. To verify the reported contradictory impacts, He et al.^7^ observed the effects of parental SI and RS during adolescence on offspring ALB, in a single study using the same group of mice. They unequivocally confirmed that compared to offspring from unstressed parents, while RS of adolescent mothers or fathers reduced ALB in both male and female offspring, their SI increased ALB predominantly in the female offspring. Thus, a direct comparison by next-generation sequencing between female offspring sired by adolescently RS- and SI-stressed mice would definitely reveal more in-depth knowledge on epigenetic transmission of anxiety.

**Figure 1:**
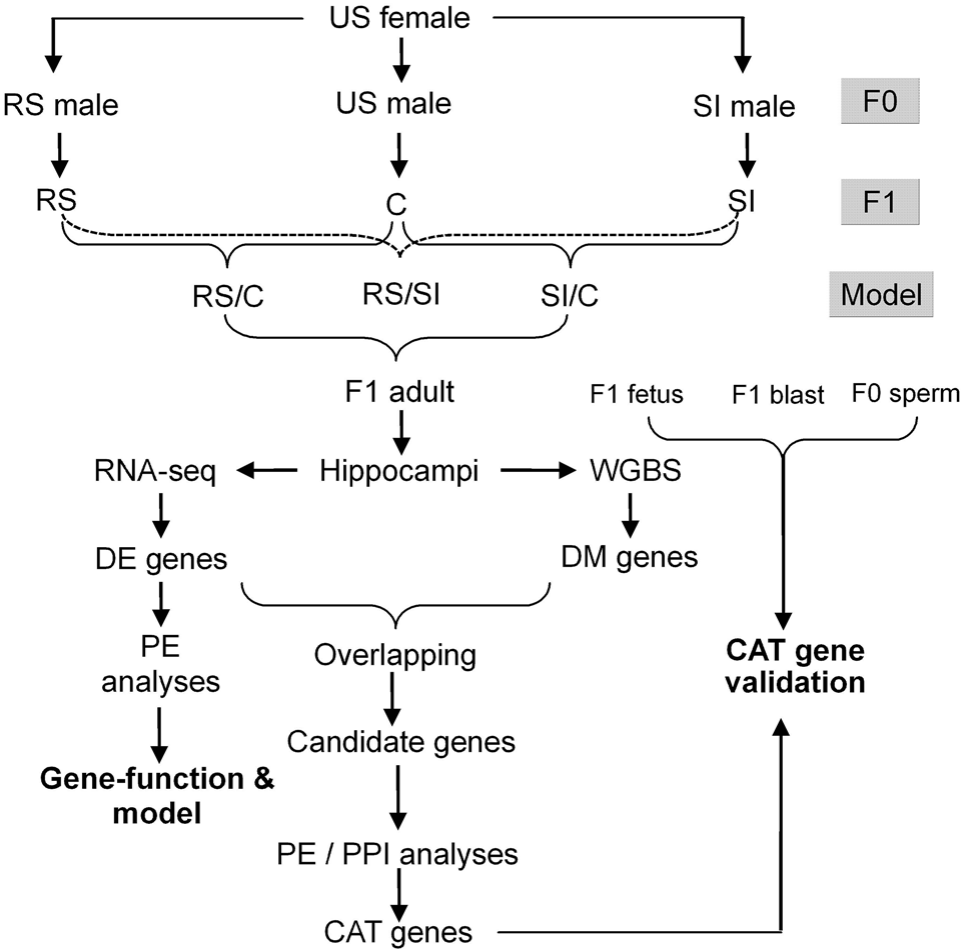
The overall design of the study. Unstressed (US) female mice were bred with restraint stressed (RS), social-instability stressed (SI) or US male mice to produce RS, SI and control (C) F1 offspring, respectively. The RS/C, SI/C and RS/SI models were set up between RS and C, SI and C or RS and SI samples, respectively. Total RNA and DNA were isolated from the hippocampi of the F1 female adult offspring and were subjected to RNA-Seq or WGBS to identify differentially expressed (DE) and methylated (DM) genes, respectively. The DE genes identified were first subjected to pathway enrichment (PE) analyses to verify gene function and model suitability, and then, they were overlapped with DM genes to obtain the candidate genes. Then, PE and PPI analyses were performed using the candidate genes to determine the candidate ALB-transmitting (CAT) genes. Finally, a DNA bisulfite sequencing was conducted using spermatozoa from F0 males, and F1 blastocysts and fetal hippocampi, to validate the CAT genes.

The objective of the present study was to observe epigenetically how RS and SI on adolescent mice cause different ALB in offspring. Unstressed (US) female mice were bred with RS, SI or US males to produce RS, SI and control (C) offspring, respectively (Fig. 1). Next-generation sequencing was conducted to identify differentially expressed (DE) and differentially methylated (DM) genes, bioinformatics was performed to verify gene function and to predict candidate ALB-transmitting (CAT) genes, and bisulfite sequencing was used to validate CAT genes.

## Results

### Paternal RS and SI stresses induced different anxiety-related phenotypes in offspring

**Figure 2:**
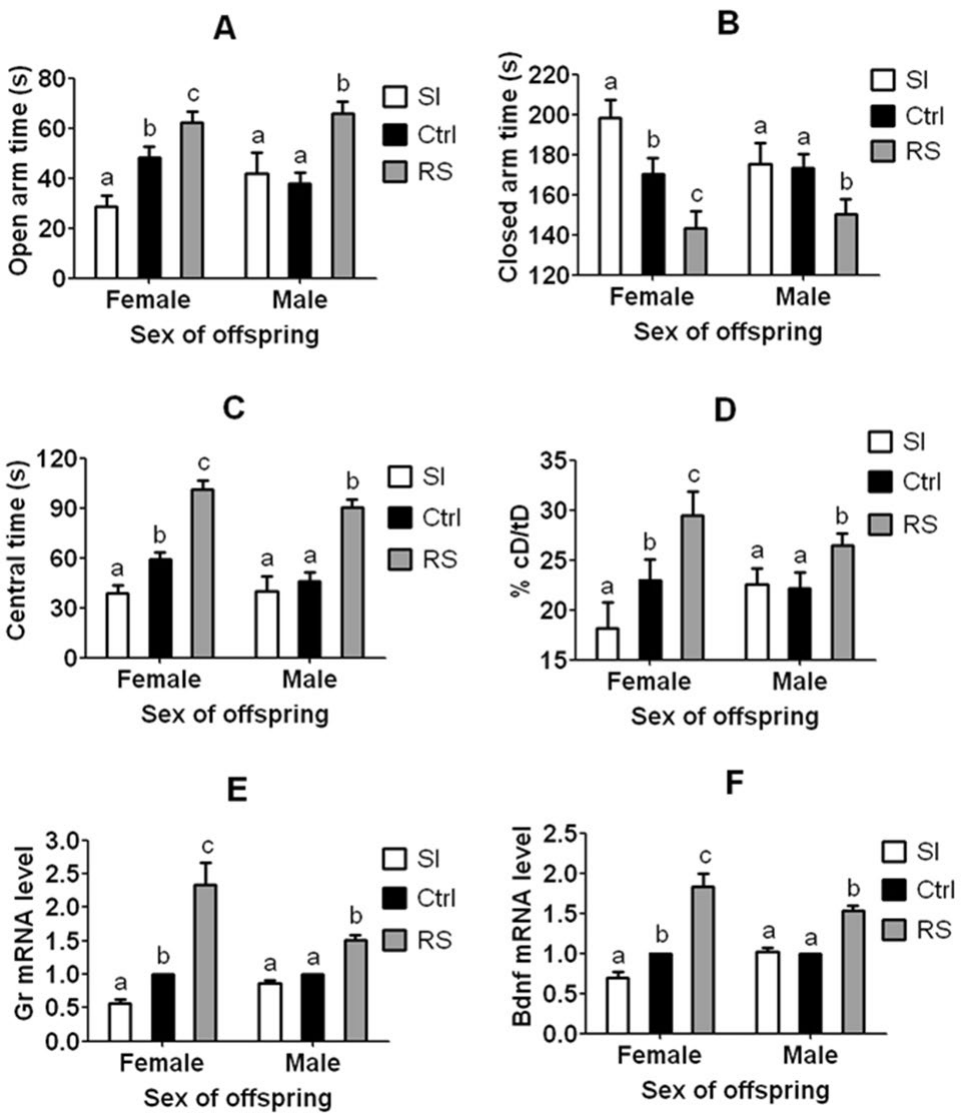
Anxiety-related phenotypes in female and male F1 offspring from F0 matings between unstressed mothers and social instability stressed (SI), restraint stressed (RS) or unstressed control (Ctrl) fathers. Graphs (**A**) and (**B**) show open and closed arm time of EPM, (**C**) and (**D**) show central area time and % central distance (cD)/total distance (tD) of OFT, and (**E**) and (**F**) show relative levels of hippocampal Gr and Bdnf mRNAs, respectively. The % of central distance (cD)/total distance (tD) of OFT was calculated by dividing the total distance a mouse traveled during the 5 min test by the distance it traveled within the central area. a–c: Values with a different letter above bars differ significantly (*P* < 0.05) within offspring sexes. Data from behavioral tests in graphs (**A**), (**B**), (**C**) and (**D**) were analyzed using LMM, whereas data from qRT-PCR in graphs (**E**) and (**F**) were analyzed using ANOVA. The *P* value refers to the fixed effect in the LMM procedure while it refers to the main effect in the ANOVA Test. Numbers and litters of F1 offspring used and the significance of difference (*P* values) for EPM, OFT and qRT-PCR are shown in Supplemental Tables S2, S3 and S4, respectively.

Both female and male offspring from RS sires showed significantly (*P* < 0.05) longer EPM open arm time (Fig. 2A, Table S2) and OFT central area time (Fig. 2C, Table S3) and significantly (*P* < 0.05) higher levels of hippocampal Gr (Fig. 2E, Table S4) and Bdnf mRNAs (Fig. 2F, Table S4) than control offspring from unstressed sires did. On the contrary, these anxiety-related parameters were significantly (*P* < 0.05) lower in female SI offspring, and did not differ (*P* > 0.05) in the male SI offspring relative to control offspring. While the closed arm time (Fig. 2B, Table S2) was opposite to open arm time during EPM, the ratio of central/total distance (Fig. 2D, Table S3) showed the same trend as the central time during OFT.

### Paternal RS and SI caused different patterns of gene expression in the hippocampi of F1 offspring

**Figure 3:**
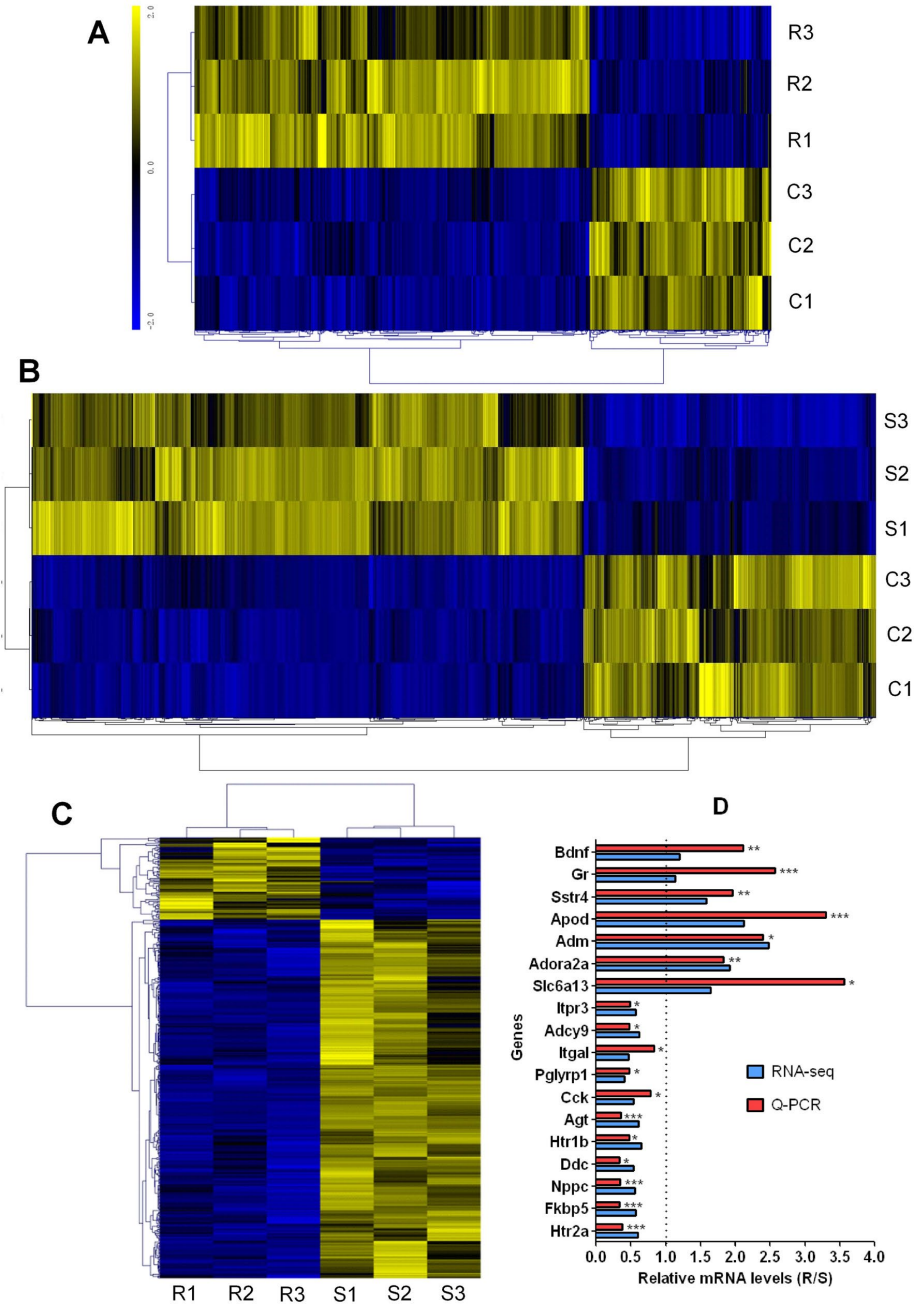
Identification of DE genes in the hippocampi of F1 female offspring sired by RS, SI or unstressed control (C) mice. Panels (**A**), (**B**) and (**C**) show heat maps showing the hierarchical cluster of DE genes identified by RNA-Seq. Three samples from the RS (R1, R2 and R3), SI (S1, S2 and S3) or C group (C1, C2 and C3) were analyzed. Each sample contained pooled RNA of three F1 mice, each from a different father. Relative mRNA levels in the RS offspring were calculated relative to those in the SI offspring. While yellow color indicates relatively upregulated genes, blue color indicates downregulated genes. Only genes with a significant change (Absolute fold change > 1.5, q < 0.05) are shown. Panel (**D**) shows qRT-PCR verification of RNA-Seq results for anxiety-related genes selected from DE genes in the RS/SI model. The qRT-PCR was performed using exactly the same samples as used for RNA-Seq. Each treatment was repeated three times with each replicate containing one sample as used for RNA-Seq. Relative mRNA levels in the RS offspring were calculated relative to those in the SI offspring, which were set to 1 (dotted line). *, ** and *** indicates significant difference from SI offspring at *P* < 0.05, 0.01 and 0.001 levels, respectively.

Our RNA-seq using hippocampi from female F1 offspring identified 1072 DE genes between RS and control (RS/C) offspring (Absolute fold change > 1.5, Q < 0.05, Fig. 3A, Dataset S1), among which 734 were upregulated and 338 were downregulated in RS relative to control offspring. The RNA-seq identified 1568 DE genes between SI and control (SI/C) offspring (Fig. 3B, Dataset S2) and among them, 1025 were upregulated and 543 were downregulated in SI offspring. Our RNA-Seq revealed 436 DE genes between RS and SI (RS/SI) offspring. Among these DE genes, 83 were up-regulated and 353 down-regulated in RS compared to SI offspring (Fig. 3C, Dataset S3).

To validate the RNA-Seq results, qRT-PCR was performed on 18 anxiety-related genes selected from DE genes in the RS/SI model using exactly the same samples as used for RNA-Seq. For all the 18 genes, qRT-PCR revealed an expression pattern similar to that detected by RNA-Seq (Fig. 3D). For most of the genes examined, the alteration amplitude detected by qRT-PCR was markedly larger than that detected by RNA-Seq, suggesting that qRT-PCR was more sensitive than RNA-Seq. Thus, our qRT-PCR results agreed with RNA-Seq data, and both confirmed that paternal RS and SI caused different patterns of gene expression.

### Pathway enrichment analysis of the DE genes

**Figure 4:**
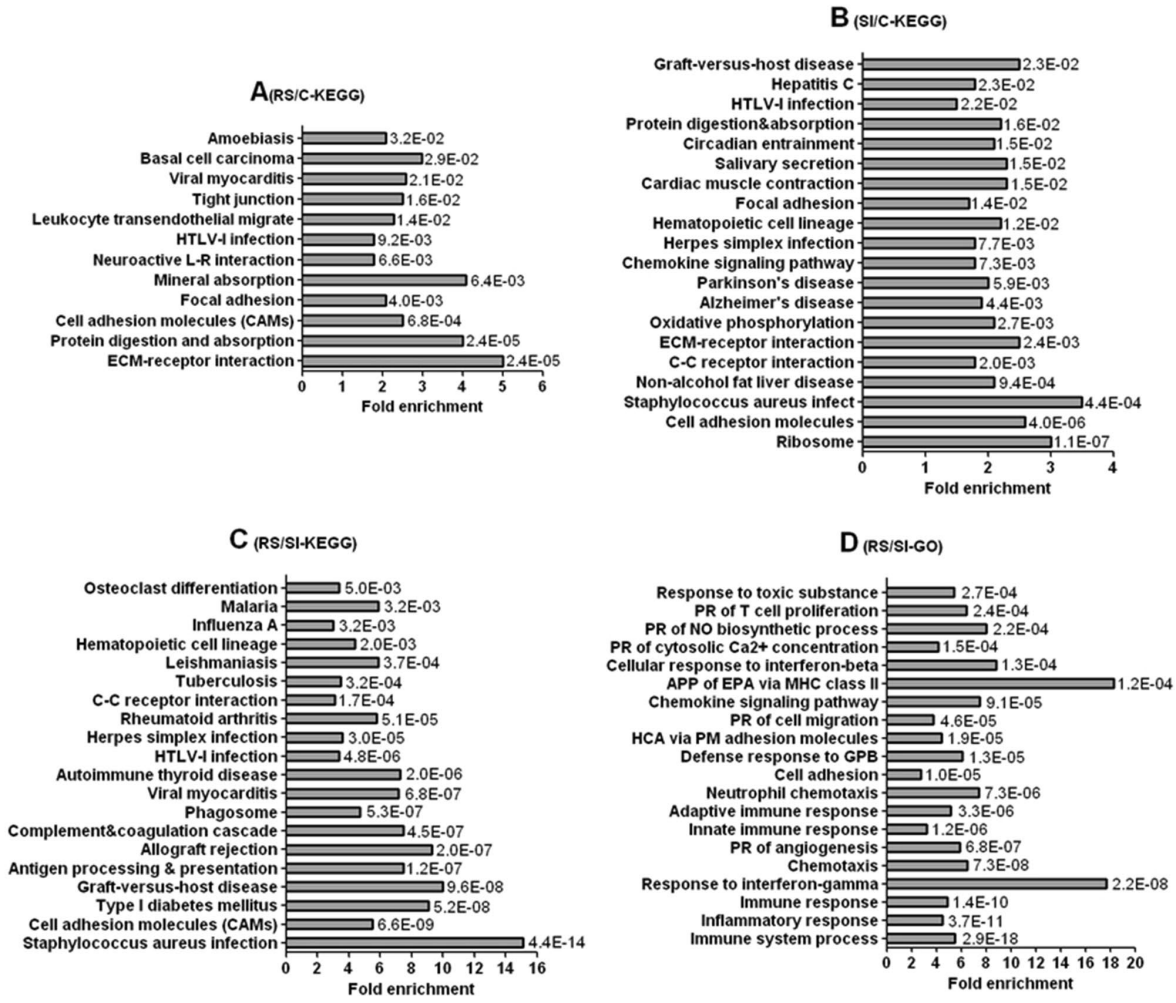
Pathway enrichment analysis of DE genes identified by the RNA-seq. Panel (**A**) shows the 12 KEGG pathways significantly enriched by DE genes between RS and control female offspring (RS/C), and panel (**B**) shows the top 20 KEGG pathways significantly enriched by DE genes between SI and control offspring (SI/C). Panels (**C**) and (**D**) show the top 20 KEGG pathways and GO biological process terms, respectively, which were significantly (*P* < 0.05) enriched by DE genes between RS and SI offspring (RS/SI). Bars are the fold enrichment of pathways and the values next to the bars are the *P* values. *APP* antigen processing and presentation, *C–C* cytokine–cytokine, *EPA* exogenous peptide antigen, *GPB* Gram-positive bacterium, *HCA* homophilic cell adhesion, *L–R* ligand–receptor, *PM* plasma membrane, *PR* positive regulation. A formal permission for the use of the KEGG pathway database was obtained from Kanehisa laboratories.

To predict functions, the DE genes identified by RNA-seq were analyzed using the enrichments of KEGG pathways and/or GO terms. The 1072 DE genes from RS/C model were significantly (*P* < 0.05) enriched in 12 KEGG pathways (Fig. 4A, Dataset S4). Eight of the significantly enriched pathways are related with inflammation or immunoreaction and 4 are concerned with neurodevelopment disorders. The 1568 DE genes from SI/C model were significantly (*P* < 0.05) enriched in 26 KEGG pathways and the top 20 are shown in Fig. 4B and Dataset S5. Eleven of the 20 pathways have relations with inflammation or immunoreaction, 8 with neurodevelopment diseases and 1 with both.

The 436 DE genes in RS/SI model were significantly (*P* < 0.05) enriched in 32 KEGG pathways (Dataset S6), and top 20 are shown in a *P* value order (Fig. 4C). All the top 20 significant pathways have relations with inflammation and/or immunoreaction, as the cell adhesion molecules play an important role in inflammatory diseases^18^, and the type I diabetes is an autoimmune disease^19^. The 436 DE genes were significantly (*P* < 0.05) enriched in 112 biological process GO terms (Dataset S7), and top 20 are shown in a *P* value order (Fig. 4D). All the 20 GO terms are concerned with inflammation and/or immunoreaction.

Thus, most of the most significantly enriched pathways from all the three models are concerned with inflammation and/or immunoreaction, and the rest have relations with neurodevelopment disorders, suggesting a strong correlation between inflammation/immunoreaction and ALB pathogenesis. Furthermore, while the fold enrichment was larger, the *P* value was smaller significantly in the RS/SI (Fig. 4C) than in RS/C (Fig. 4A) or SI/C KEGG pathways (Fig. 4B).

### Paternal RS and SI differently affected overall patterns of DNA methylation in hippocampi of the F1 offspring

We conducted WGBS using hippocampi to search for DM loci between RS and SI female F1 offspring, and identified a total of 4129 DM regions (DMRs) from RS compared to SI offspring (*P* < 0.00005, Dataset S8). Among the DMRs identified, 2624 (64%) were hyper-methylated and 1505 (36%) were hypo-methylated; 60%, 20% and 11% DMRs were distributed in intron, exon and promoter elements, respectively. As hyper-and hypo-methylation are usually associated with down- and up-regulation of gene expression, respectively^20^, these WGBS results were consistent with our RNA-seq results that more genes were down- than up-regulated in RS relative to SI offspring. These 4129 of DMRs were associated with 3416 annotated genes.

### Prediction of CAT genes

**Figure 5:**
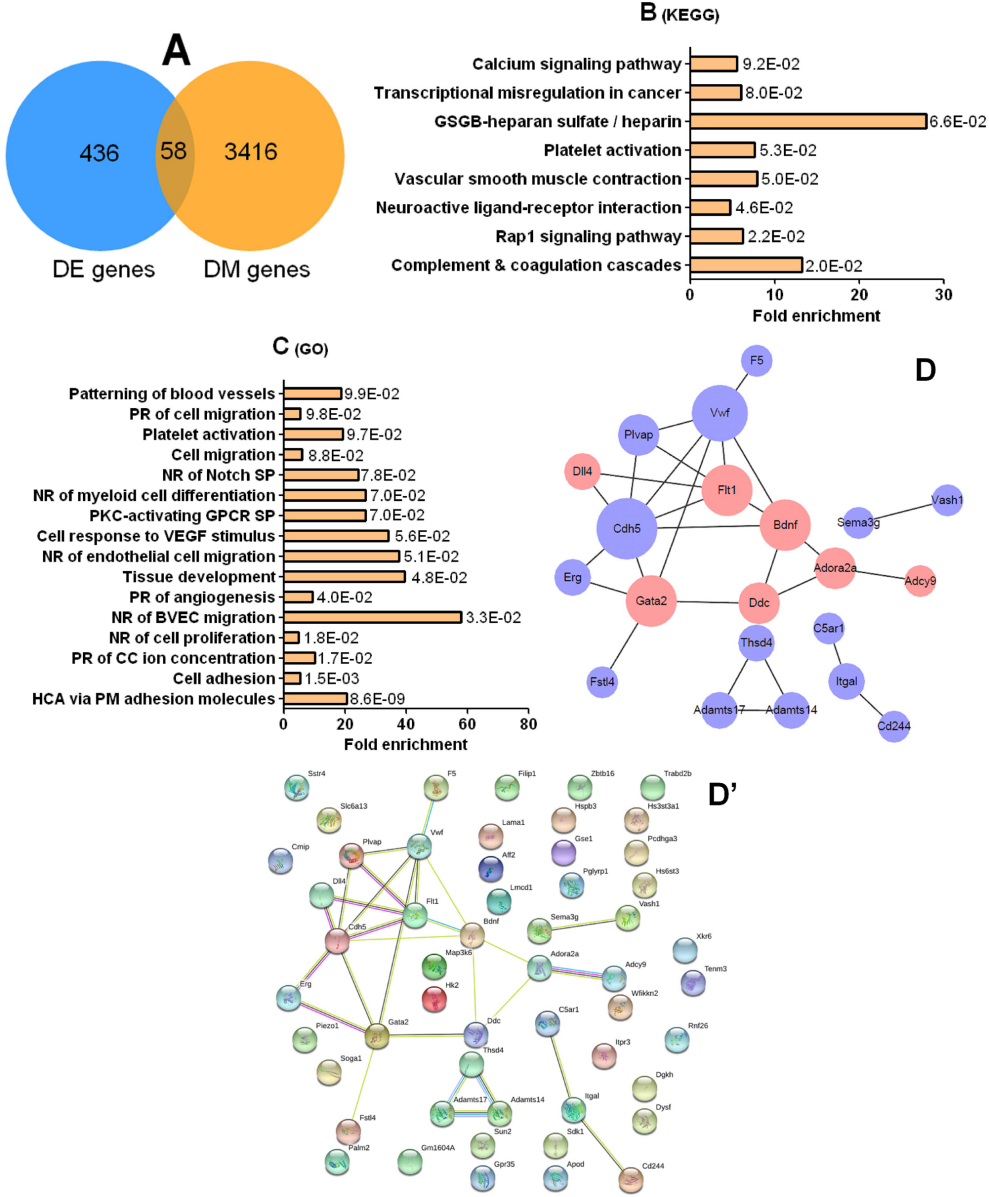
Selection and pathway enrichment or PPI analyses of candidate genes to predict the candidate ALB-transmitting (CAT) genes using RS/SI female offspring. Relative levels of DE and DM gene expression in the RS offspring were calculated relative to those in the SI offspring. In panel (**A**), 58 candidate genes were selected by overlapping the 436 DE genes with the 3416 DM genes. Panels (**B**) and (**C**) show the KEGG pathways and GO biological process terms, respectively, which were enriched by the 58 candidate genes. Bars are the fold enrichment of pathways and the values next to the bars are the *P* values. *BVEC* blood vessel endothelial cell, *CC* cytosolic calcium, *GPCR* G-protein coupled receptor, *GSGB* Glycosaminoglycan biosynthesis, *HCA* homophilic cell adhesion, *NR* negative regulation, *PKC* protein kinase C, *PM* plasma membrane, *PR* positive regulation, *SP* signaling pathway, *VEGF* vascular endothelial growth factor. Panels (**D**) and (**D′**) show the PPI network constructed with the 58 candidate genes and the Bdnf gene using Cytoscape. While (**D**) shows modules with only nodes of significant connectivity, (**D′**) shows the original picture with all the 59 genes. In graph (**D**), the size of each node was proportional to the degree of its connectivity. While the CAT genes were colored reddish, other genes were colored pale pinkish purple. A formal permission for the use of the KEGG pathway database was obtained from Kanehisa laboratories .

We compared the 436 DE genes and the 3416 DM genes in the RS/SI model and found that 58 genes were overlapped (Fig. 5A). Among the 58 candidate genes, 39 had DMRs solely on introns, 11 had DMRs on non-intron elements and 8 had DMRs over both intron and non-intron elements (Table S5). The 58 candidate genes were enriched in 8 KEGG pathways (*P* < 0.1, Fig. 5B), all of which are involved in immune/inflammation reactions and/or brain development/disorders (Supplementary passage 1). Among the 58 genes, Adcy9 and Adora2a were enriched in 4, Itpr3 in 3, and Vwf, C5ar1 and Flt1 in 2 of the 8 KEGG pathways. All these top enriched genes were involved in neural functions or disorders (Supplementary passage 2).

The 58 candidate genes were enriched in 16 GO terms of biological processes (Fig. 5C). While some relate to development and plasticity of the nervous system, most of the 16 terms have direct or indirect relations with structure and function of blood vessels. The blood–brain barrier dysfunction may both lead to and be induced by aging, multiple sclerosis, Alzheimer’s and Parkinson’s diseases and epilepsy (Supplementary passage 3). Furthermore, angiogenesis and inflammation are two highly linked processes. Among the most frequently enriched genes, Dll4 was enriched in 6/16 terms, Gata2 and Flt1 in 5, Cdh5 in 3, and Vwf, Adora2a, C5ar1 and Sstr4 in 2 terms. All the most frequently enriched genes have direct or indirect relations with brain neurogenesis and diseases (Supplementary passage 4).

A PPI network was constructed using the 58 candidate genes along with Bdnf gene (Fig. 5D,D′). Bdnf is a well-known anxiety-related gene, and our qRT-PCR and WGBS revealed a significant difference in its mRNA expression (*P* < 0.01, Fig. 3D) and DNA methylation (RS 66.59% vs. SI 93.76%, *P* < 0.05), respectively, between RS and SI offspring. The major module of PPI network contained 12 interconnected genes that had direct or indirect connections with Bdnf (Fig. 5D). Among the 12 genes, Adcy9, Adora2a, Itpr3, Dll4, Gata2, and Flt1 were top enriched in KEGG pathways or GO terms. Although the Ddc gene was not top enriched, it showed a direct connection with Bdnf in PPI network (Fig. 5D). Thus, Adcy9, Adora2a, Itpr3, Dll4, Gata2, Flt1, Ddc and Bdnf were selected as candidate CAT genes. Among the 8 genes, Adcy9, Itpr3, Flt1, Ddc and Bdnf had DMRs solely in introns, Adora2a and Gata2 had DMRs in promoter, exon and intron, and Dll4 had DMR solely in promoter (Table S5).

### Validation of CAT genes

**Figure 6:**
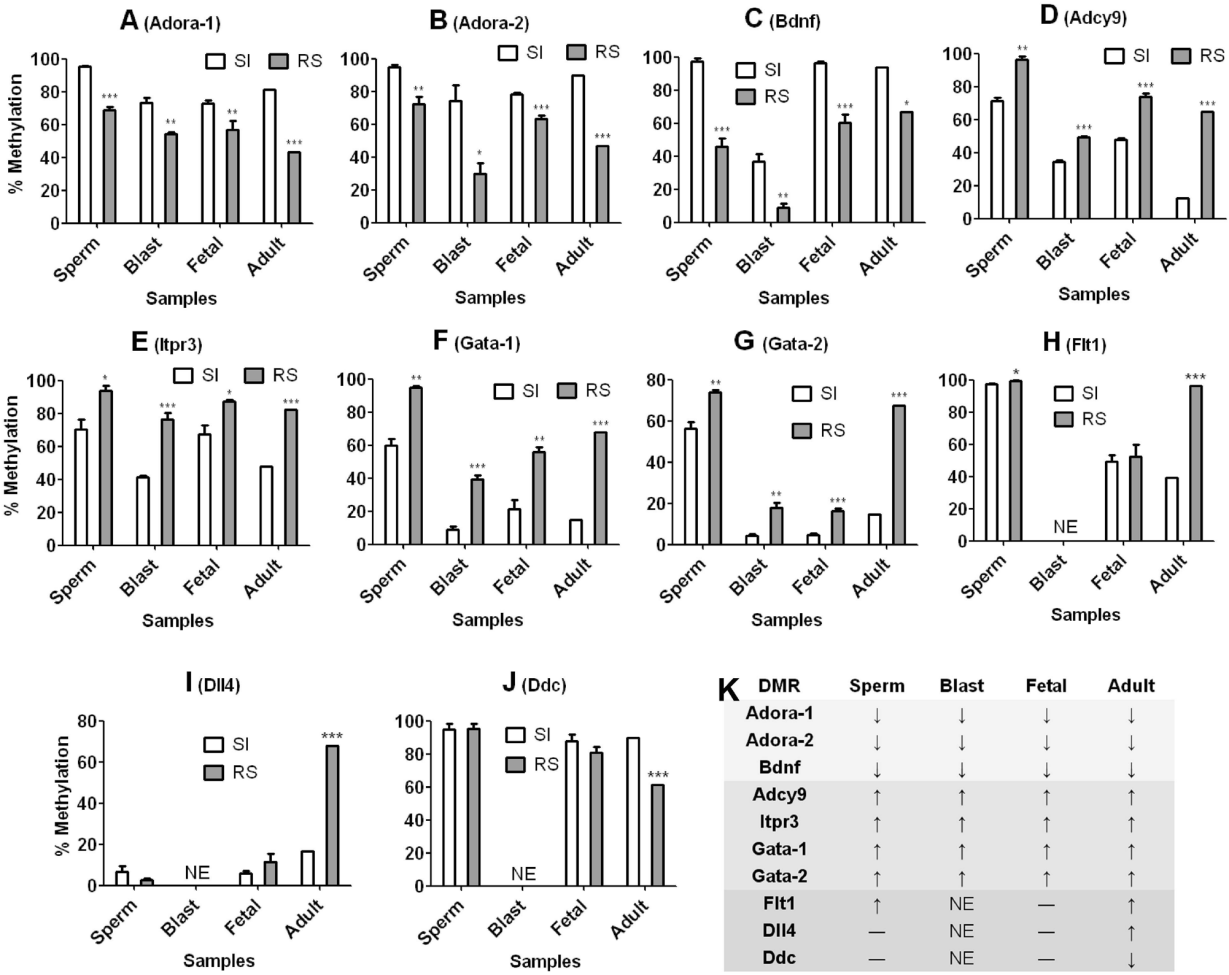
Bisulfite sequencing for the DMR methylation status of candidate CAT genes in F0 sperm and F1 blastocysts, fetal and adult hippocampi of the RS/SI model. Each treatment was repeated at least three times and each replicate contained spermatozoa, blastocysts or fetal hippocampi recovered from one mouse. Data were analyzed using Independent-Samples T Test. Graphs A to J show percentages of methylated CpGs. *, ** and *** indicates significant difference from SI samples at *P* < 0.05, 0.01 and 0.001 levels, respectively. Graph K summarizes methylation trends in RS samples relative to SI samples of different genes from sperm to blastocysts to fetal and adult hippocampi. Data for adult hippocampi were obtained from our WGBS (Dataset S8). *Adora-1 and -2* Adora2a-DMR1 and -DMR2, *Gata-1 and -2* Gata2-DMR1 and -DMR2, ‘↓’: lower methylation, ‘**↑**’: higher methylation, ‘—’: insignificant difference, *NE* not examined.

Our DMR bisulfite sequencing demonstrated that Adora2a, Bdnf, Acdy9, Itpr3 and Gata2 in F0 sperm and F1 blastocysts and fetal hippocampi shared the same trend of methylation with respective genes in adult hippocampi (Figs. 6 and S1). Thus, while percentages of methylated CpGs in Adora2a and Bdnf were significantly (*P* < 0.05) lower, those in Acdy9, Itpr3 and Gata2 were significantly (*P* < 0.05) higher in RS than in SI samples. Methylation levels of Dll4 and Ddc in sperm and fetal hippocampi, however, did not differ significantly (*P* > 0.05) between RS and SI samples, suggesting a methylation trend different from that in adult hippocampi where Dll4 and Ddc were hyper- and hypo-methylated, respectively, in RS samples. Although Flt1 was hyper-methylated (*P* < 0.05) in RS than SI sperm, consistent with that in adult hippocampi, the difference was insignificant (*P* > 0.05) between RS and SI fetal hippocampi. Furthermore, we observed an overall decline in methylation from sperm to blastocysts, and the methylation level increased again in fetal or adult hippocampi (Fig. 6A–G), suggesting that our CAT genes had undergone the massive demethylation routinely observed in early embryos. The results confirmed Adora2a, Bdnf, Acdy9, Itpr3 and Gata2 as CAT genes because they each showed a consistent pattern of methylation from F0 sperm to F1 blastocysts, fetal and adult hippocampi.

### Effects of SI or RS on sperm DNA-methylation: validation involving samples from unstressed control sires

**Figure 7:**
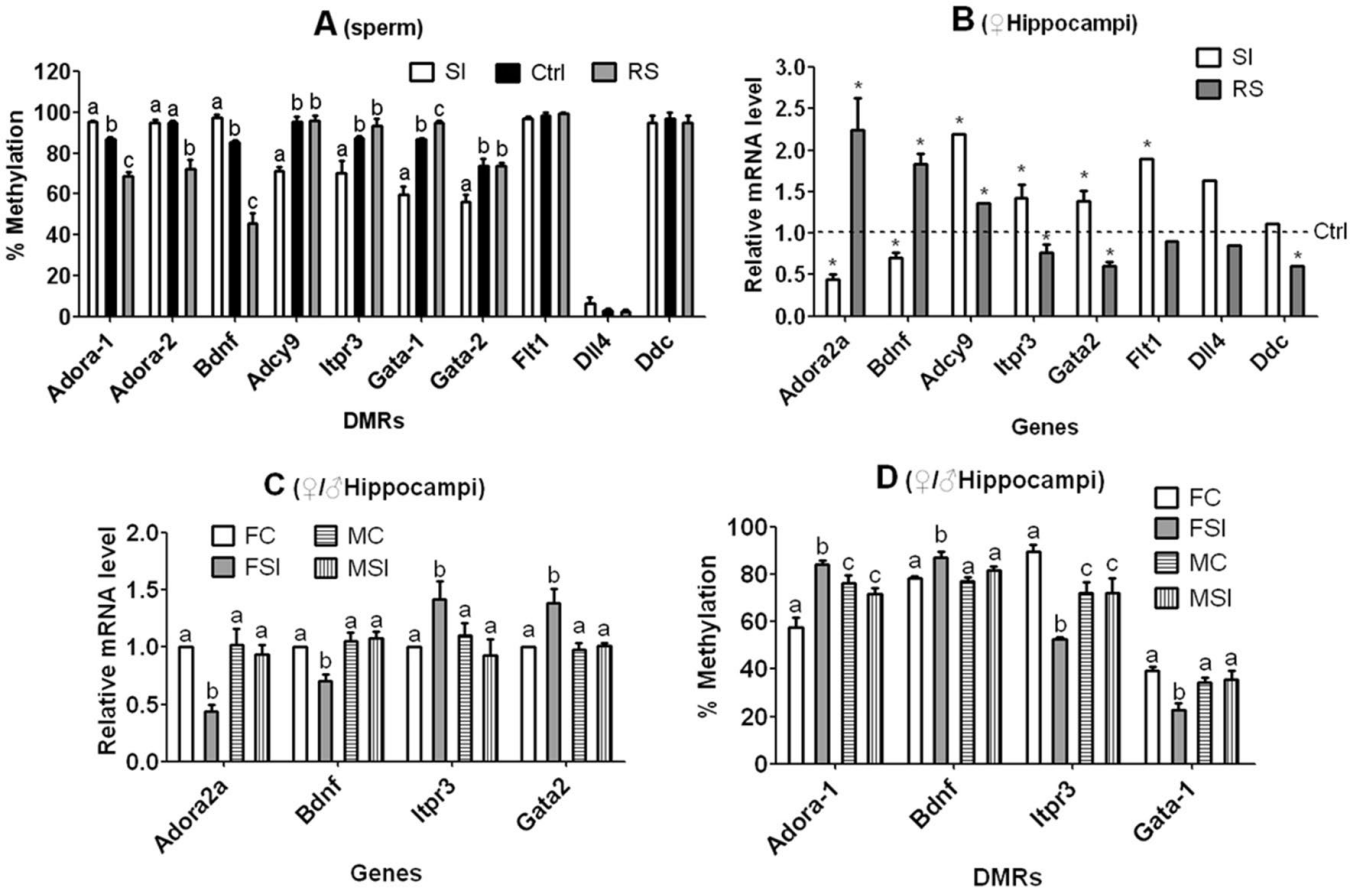
Validation of the CAT genes in experiments involving samples from unstressed control fathers and SI male offspring. Each treatment was repeated at least three times and each replicate contained spermatozoa or hippocampi recovered from one mouse. Data were analyzed using ANOVA. Graph (**A**) compares DNA methylation levels (Bisulphate sequencing results) of the 8 candidate CAT genes between sperm from unstressed control (Ctrl), SI and RS mice. Data for SI and RS mice were taken from Fig. 6. Graph (**B**) compares mRNA levels in hippocampi of adult female offspring between Ctrl (dotted line), SI and RS fathers using data from qRT-PCR (for Adora2a, Bdnf, Itpr3, and Gata2) or RNA-seq (for the rest 4 genes). All the 8 candidate CAT genes except for Adcy9 showed correct trends of expression with control values in between the SI and RS values, and all the 5 CAT genes displayed a significant difference from controls. Graphs (**C**) and (**D**) compare mRNA levels (qRT-PCR results) and DNA methylation (bisulphate sequencing results) levels, respectively, of the 4 control sample-verified CAT genes in adult hippocampi between female control (FC) and SI (FSI) or male control (MC) and SI (MSI) offspring. In graph (**C**), the value for FC offspring was set to 1 and the other values were expressed relative to it. Each treatment was repeated at least 6 times in graph (**C**) and 3 times in graph (**D**) with each replicate including one individual. a–c: Values with a different letter above bars differ significantly (*P* < 0.05) within genes or DMRs. * indicates significant (q < 0.05) difference from control values.

To verify that SI and RS did cause DNA-methylation changes in male germline, we carried out DMR bisulphate sequencing of the 8 candidate CAT genes on sperm from unstressed control mice, and compared results with those in sperm from SI or RS mice shown in Fig. 6. Among the 10 DMRs examined, 6 showed expected trends of methylation with the control values either well (*P* < 0.05) in between SI and RS (Adora2a-DMR1, Bdnf and Gata2-DMR1) or similar (*P* > 0.05) to both (Flt1, Dll4 and Ddc) (Figs. 7A, S1 and S2). The Itpr3 DMR also showed the expected methylation trend although the difference between control and RS sperm did not reach a significant level (*P* = 0.148). Thus, 7 of the 10 DMRs examined and at least one DMR in each of the 4 CAT genes showed correct methylation trends when sperm from unstressed mice were considered. Furthermore, Adora2a, Bdnf, Gata2 and Itpr3 each had a DMR with correct methylation trend, just opposite to their mRNA levels in hippocampi of female offspring (Fig. 7B). Thus, our experiments involving samples from unstressed control males showed that SI and RS on male mice induced significant DNA methylation changes in sperm, and confirmed that Adora2a, Bdnf, Gata2 and Itpr3 were CAT genes.

### Exploring the mechanisms by which paternal SI cause different ALB between male and female offspring

We then compared levels of mRNA expression and DMR methylation of the 4 CAT genes in adult hippocampi between female and male offspring sired by SI fathers. The mRNA levels of all the 4 genes did not differ (*P* > 0.05) between male and female control offspring (Fig. 7C). While paternal SI changed mRNA levels of all the 4 genes significantly (*P* < 0.05) in an ALB-correlated manner in female offspring, it did not affect those in male offspring (*P* > 0.05). In all the 4 DMRs examined, while paternal SI changed their methylation levels significantly (*P* < 0.05) in an opposite direction to their mRNA levels in female offspring, it did not affect those in male offspring (*P* > 0.05) (Figs. 7D and S3). The results further confirmed Adora2a, Bdnf, Gata2 and Itpr3 as CAT genes, and indicated that paternal SI caused sex-linked ALB difference in offspring through mechanisms involving the CAT genes.

## Discussion

We have observed epigenetically how RS and SI on adolescent males induced different ALB in offspring. Among the 3 models tested in this study, the RS/SI model was found more suitable to produce significant differences than the RS/C or SI/C models for study of ALB pathogenesis and for identification of CAT genes. Our next-generation sequencing and bioinformatics using the RS and SI female offspring identified 5 candidate CAT genes; each showed a consistent pattern of DNA methylation from F0 spermatozoa through F1 blastocysts to fetal and adult hippocampi. Our analysis involving sperm samples from unstressed control sires further validated 4 as the CAT genes. DNA methylation changes have been reported in sperm following male stresses^15,21^. Among the 4 CAT genes, Bdnf is well known for its anxiolytic effect. The Adora2a-knockout mice showed more anxiety than the wild-type mice did^22,23^. Overexpression of human GATA-1 and GATA-2 impaired spine formation and caused depressive behavior in rats^24^. Furthermore, Mice lacking Itpr3 showed abnormal behavioral and electrophysiological responses to sweet, umami and bitter substances that trigger G-protein-coupled receptor activation^25^. Thus, the present results suggested that stresses on males might alter offspring’s ALB by modifying sperm DNA methylation. Sperm miRNAs as mediators of stress phenotypes have been reported by Rodgers et al.^8^ and Short et al.^14^, who observed that sperm miRNA injection into zygotes mimicked the effects of mating stressed males.

Most of the KEGG pathways significantly enriched by DE genes between RS or SI and control offspring were related with inflammation or immunoreaction, and the rest were concerned with neurodevelopment disorders. Furthermore, both our KEGG and GO analyses on DE genes between RS and SI female offspring showed that all the top 20 significant pathways/terms had relationship with inflammation and/or immunoreaction. Growing evidence suggests that inflammation and immune dysregulation may play an important role in anxiety and depressive disorders^26^. For example, inflammation elevation is observed in men with current anxiety disorders, and immune dysregulation is especially obvious in persons with a late-onset anxiety disorder^27^. Psychiatric disorders have been found with elevated concentrations of inflammatory signals^28^. Chronic stress, one of the most pertinent risk factors of depression and anxiety, can induce behavioral and affective-like problems through neuroimmune alterations^29^.

In addition, stress or inflammation can enhance anxiety through disturbing tryptophan metabolism in the kynurenine pathway^30^. For example, it was reported that the activated immune-inflammatory pathways activated indoleamine-2,3-dioxynease (IDO) and the tryptophan catabolite (TRYCAT) pathway thereby facilitating tryptophan degradation and increasing the production of TRYCATs, which produced an overall anxiogenic effect^31^. Furthermore, the effects of immune activation on IDO were more significant in women than in men, suggesting that females may show increased levels of anxiogenic TRYCATs following activation of immune-inflammatory pathways. This might help us to explain why anxiety disorders are seen more frequently in women than in men and why the anxiogenic effects of our SI on male mice were transmitted preferentially to female offspring.

The present results demonstrated that in the hippocampi of F1 female offspring, only a few (11%) of DMRs overlapped with promoters, whereas a greater proportion (60%) of DMRs located in the intronic regions. Among the 58 candidate genes, 47 had DMRs in introns. Furthermore, all the 5 candidate CAT genes had DMRs in introns. Thus, it seemed that the expression of ALB-related genes in hippocampi was regulated mainly by DNA methylation in intronic regions. Previous studies suggested that DNA methylation in the intronic region might be involved in pathogenesis of psychiatric disorders. For example, DNA methylation in intronic regions has been described during neuronal development in mouse hippocampus^32^. Xiao et al.^33^ demonstrated that in the brain of schizophrenia and bipolar disorder patients, only a few DMRs overlapped with promoters, whereas a greater proportion occurs in introns and intergenic regions, suggesting that DNA methylation changes located in introns might alter important psychiatric disorder-related biological processes. Furthermore, Zhao et al.^34^ observed that parts of intronic DMRs overlapped with some intragenic miRNAs, and the intronic DMR-related miRNAs played important roles in major psychosis.

Both the present results and our previous study^7^ showed that compared to controls, while paternal RS decreased ALB in both female and male offspring, paternal SI increased ALB in female offspring while having no effects on male offspring. Our qRT-PCR indicated that while both female and male offspring from RS sires showed significantly higher levels of Gr and Bdnf mRNAs than control offspring, the levels of Gr and Bdnf mRNAs were significantly lower in female SI offspring, and did not differ in the male SI offspring relative to control offspring (Fig. 2). Furthermore, while paternal SI changed mRNA levels of all the 4 CAT genes significantly in an ALB-correlated manner in female offspring, it did not affect those in male offspring (Fig. 7C). In all the 4 DMRs examined, while paternal SI changed their methylation levels significantly in an opposite direction to their mRNA levels in female offspring, it did not affect those in male offspring (Fig. 7D). Taken together, the results confirmed that the 4 CAT genes and the Gr gene were really involved in ALB regulation.

Although both previous studies^7,8^ and the present results indicated that paternal SI increased ALB of female offspring while having no effect on that of male offspring, the mechanisms by which paternal SI cause sex-linked ALB difference in offspring is largely unknown. By comparing levels of mRNA expression and DMR methylation of the 4 CAT genes in adult hippocampi between female and male offspring sired by SI fathers, this study showed that paternal SI might cause sex-linked ALB difference in offspring through mechanisms involving the Adora2a, Bdnf, Gata2 and Itpr3 genes. In their efforts to elucidate the mechanisms involved in the SI transgenerational effects, Saavedra-Rodríguez and Feig^6^ observed that the calcineurin inhibitors, Rcan1 and Rcan2, were elevated in the hippocampal CA1 region of female offspring sired by SI mice. Furthermore, Dickson et al.^35^ reported that SI of male mice elevated miR-409-3p levels in sperm, and injection of miR-409-3p inhibitor into zygotes resulted in anxiolytic effect in female, but not male offspring.

In summary, our study demonstrated that RS and SI female offspring were suitable models for research on ALB epigenetic inheritance. Adora2a, Bdnf, Itpr3 and Gata2 were among the CAT genes. Paternal SI caused sex-linked ALB difference in offspring through mechanisms involving the CAT genes. Furthermore, our results also demonstrated a strong correlation between inflammation or immune dysregulation and ALB pathogenesis, and an important function for intronic DNA methylation in regulating ALB-related genes. In conclusion, the results suggested the possibility that paternal stress might alter offspring’s ALB by modifying sperm DNA methylation of the CAT genes and via mechanisms involving inflammation and immunoreaction. Beyond contributing to our understanding of the epigenetic mechanisms by which parental experiences cause psychological alterations in offspring, our work will contribute to prediction, prevention and treatment of human anxiety and depression disorders.

## Methods

This study was carried out in compliance with the ARRIVE guidelines. All methods were performed in accordance with the relevant guidelines and regulations. The methods used for animal care and handling were approved by the Shandong Agricultural University Animal Care and Use Committee, P. R. China (Permit number: SDAUA-2001-001). Unless otherwise mentioned, all chemicals and reagents were purchased from Sigma Chemical Co. (St. Louis, MO, USA).

### Mice and stress treatment

Mice of the Kunming strain, which were originally derived from ICR (CD-1) mice, were bred in this laboratory. The mice were kept in a room with a constant temperature of 22–25 °C and light–dark cycles of 14 h/10 h with the dark beginning at 8:00 pm. Adolescent male mice were 26–28 days after birth at the initiation of the stress treatment. For RS treatments, an individual mouse was placed in a micro-cage constructed by the authors, which was placed in an ordinary home cage^36^. The micro-cage offered the same photoperiod and controlled temperature as in the home cage. While in the micro-cage, mice could move back and forth to some extent, but they could not turn around. Food and water were consumed ad libitum during the restraint sessions. The restraint was conducted for 8 h per day (from 8:00 a.m. to 16:00 p.m.) for 8 weeks. By measuring serum cortisol levels and food/water intake, our previous study demonstrated that this long-term RS system stressed mice consistently while having no effects on food and water intake^7^.

The SI treatment was conducted using procedures reported previously by Schmidt et al.^37^, who observed that the SI treatment persistently altered HPA axis function and behavior. The cage mates in each cage were changed twice per week so that four mice from different cages were put together in a new cage. Efforts were made to minimize the likelihood of a repeated encounter of the same mice throughout the 8 weeks of experiment. Control mice were housed singly in regular home cages while the stressed mice were receiving RS or SI treatment. At the end of the RS or SI treatment, the mice were housed regularly in the home cage before breeding.

### Breeding

On day 5 after the end of the RS or SI treatment, each stressed male F0 mice (12 weeks after birth) were mated with two unstressed females (8 weeks after birth) to produce F1 offspring. The female mice were examined for vaginal plugs the next morning, and those showing a plug were removed from the mating cage and fed alone until parturition. In this study, the morning when vaginal plugs were detected was designated as embryonic day 0.5 (E0.5). The F1 offspring were weaned on postnatal day 21 and left undisturbed until behavior testing, which took place at 2 months of age. Three to five siblings of the same sex were housed in the same cage until testing.

### Elevated plus-maze (EPM) test and open field test (OFT)

The device and procedures used for EPM and OFT tests were those reported previously^7^. The EPM device consisted of two open arms (30 × 6 cm), alternating at right angles with two closed arms (30 × 6 × 15 cm). The central platform delimited by the four arms was 36 cm^2^. The whole maze was elevated 50 cm above the floor. The OFT device consisted of a black square floor of 50 cm × 50 cm and white walls 45 cm tall. The floor was divided into 25 squares of 10 cm × 10 cm, of which those 16 squares touching a wall were designated as peripheral area and the 9 remaining squares as the central area.

The test room was maintained at 22–25 °C with continuously provided 50–55 dB white noises. Illumination levels were provided by four 36-W lamp tubes that give a light intensity of 357 lx. The tests were always performed between 8:00 and 11:00 a.m. Immediately before the test, mice were individually put in the test room for 30 min for them to habituate to the test environment. To begin EPM test, a mouse was placed on the central platform, facing an open arm, and was allowed to explore the maze for 5 min. Following a four-paw criterion, numbers of entries and time spent in each arm over the total exploration in both open and closed arms were calculated using an Any-maze software (Stoelting Co, Illinois, USA). The OFT was performed one week after the EPM test. To start the OFT test, a mouse was placed in the central area and was allowed to explore the open field for 5 min. Then the numbers of central entries, time spent in central area and distance traveled in the open field were calculated using the Any-maze software. The % of central distance (cD)/total distance (tD) of OFT was calculated by dividing the total distance a mouse traveled during the 5 min test by the distance it traveled within the central area. After each trial, the device was cleaned with 10% ethanol to effectively remove the scent of the previously tested animal.

### Recovery of hippocampi, blastocysts and spermatozoa

Hippocampi were recovered either from female and male F1 adults at 2 months of age in the afternoon on the same day after the OFT test, or from female F1 fetuses on E17-19 before birth. Of the two hippocampi collected from each F1 offspring, one was used for RNA sequencing and the other for whole genome bisulfite sequencing (WGBS). Blastocysts were collected by flushing uterine horns with M2 medium on E3.5 from female mice that had been bred with stressed F0 males. Mature spermatozoa were recovered from F0 male mice as reported previously^38^. Briefly, immediately at the end of RS or SI, caudae epididymides and vas deferens were perforated and squeezed in M2 media. The sperm masses recovered were mixed by pipetting, and the resultant suspension was transferred to a 15-ml Falcon tube for swimming up for 30 min at 37 °C. Then, the upper part suspension containing motile sperm was recovered, washed twice in PBS and once in water by centrifugation (3000 × g for 5 min). Finally, the sperm suspension was incubated in cell lysis buffer and the lysates produced were stored at − 80 °C until use.

### Quantitative real-time PCR (qRT-PCR)

Briefly, 50–100 mg of hippocampal tissue were homogenized using 1 ml Trizol reagent. The RNAs isolated were re-suspended in DEPC-dH_2_O. To assess the RNA purity and integrity, we determined the A_260_/A_280_ ratio (1.8–2.0) and conducted electrophoresis in 1% agarose. For cDNA synthesis, we performed reverse transcription with Superscript III Reverse Transcriptase (18080-044, Invitrogen Australia Pty. Ltd). The final cDNA product was stored at − 20 °C before use.

Table S1 shows the gene-specific primers used for qRT-PCR. We quantified mRNA using the Mx3005P real-time PCR instrument (Stratagene, Valencia, CA). We conducted amplification reactions using a 10-μl reaction volume including 1-µl cDNA, 5-µl 2× SYBR Green Master Mix (ST600548, Stratagene), 0.15-µl ROX, 3.05-µl RNase-free water, and 0.4-µl forward and 0.4-µl reverse gene-specific primers (10 µM). Conditions for the cycle amplification are as follows: (a) 10 min of denaturation at 95 °C; and (b) 40 cycles at 95 °C for 5 s and at 60 °C for 20 s. We normalized the gene expression to gapdh as the internal control. Then, by using the 2^− (ΔΔCT)^ method, we expressed all values relative to the control samples.

### RNA-sequencing

Three samples from RS (designated as R1, R2 and R3), SI (S1, S2 and S3) or control groups (C1, C2 and C3) were analyzed. Each sample contained pooled RNA of three F1 mice, each from a different father. The mRNA sequencing was performed by the Annoroad Gene Technology Corporation (Beijing). Briefly, the purity, integrity and concentration of RNA extracted from hippocampi were assessed. 2 µg RNA per sample was used to generate sequencing libraries with a NEBNext Ultra RNA Library Prep Kit for Illumina (#E7530L, NEB, USA). Clustering of the index-coded samples was performed on a cBot cluster generation system (Illumina). After cluster generation, the libraries were sequenced on an HiSeq sequencing platform (Illumina) and 150 bp paired-end reads were generated. The RNA-seq data generated an average of 45170529, 44022102 and 43767022 clean reads from Ctrl, SI and RS sample, of which approximately 87.70%, 88.45% and 88.44% were mapped to the mouse genome, respectively. High-quality clean reads were used for transcriptome assembly (accomplished in Trinity v.20140717). Differential gene expression was analyzed using DESeq v1.14.0. Genes with a fold change of > 1.5 and a difference of q < 0.05 were identified as differentially expressed (DE) genes.

### WGBS of hippocampal DNA from F1 offspring

The hippocampi used for WGBS were from the same F1 offspring as used for RNA-Seq. DNA from the three samples of nine mice was pooled and used as one sample for analysis. WGBS was conducted by the Annoroad Gene Technology Co. (Beijing, China). Briefly, 1 µg DNA per sample was used to generate sequencing libraries with the Illumina TruSeq DNA Library preparation kit. End-repair, A-tailing, ligation of methylation adaptors and subsequent bisulfite conversion of the sequencing libraries was performed according to protocol. Bisulfite converted adaptor-ligated fragments were amplified by PCR. The libraries were subjected to paired-end 100 bp sequencing on a HiSeq sequencing platform (Illumina). Our WGBS yielded 567790000, 581040000 and 567045000 clean reads from Ctrl, SI and RS samples, of which 73.96%, 71.78% and 72.36% were aligned to the mouse reference sequence, respectively, with genome depth averaging above 20×. More than 85% cytosines were covered by at least one read in each sample. The bisulfite conversion rate was 99.69%, 99.47% and 98.74%, respectively.

### Pathway enrichment and protein–protein interaction analyses

The KEGG and GO enrichments of DE, DM or candidate genes were implemented using DAVID (the Database for Annotation, Visualization and Integrated Discovery) with genes in the whole genome as the data background. Only those GO terms or KEGG pathways that showed a *P* value of < 0.05 were judged as enriched significantly. We used the Search Tool for the Retrieval of Interacting Genes (STRING) and Cytoscape software for construction of the protein–protein interaction (PPI) network.

### Bisulfite sequencing of the candidate AT genes

Each treatment was repeated at least three times and each replicate contained spermatozoa, blastocysts or fetal hippocampi recovered from one mouse. Genomic DNA was isolated from F0 spermatozoa and fetal hippocampi using Minibest Universal Genomic DNA Extraction Kit (9765, Takara). Bisulfite conversion of DNA was performed using an EpiTect Bisulfite Kit (59104, QIAGEN). To isolate genomic DNA from F1 blastocysts, cells were incubated in lysis buffer containing proteinase K (9765, Takara) at 56 °C for 1–1.5 h. Following boiling and denaturation with 0.3 M NaOH, DNA was mixed with low melting point agarose to form beads, which were treated with 5 M bisulfite solution at 50 °C for 8–10 h in the dark. The beads were desulfonated in 0.3 M NaOH. The converted DNA was then amplified by nested PCR with two pair primer sequences shown in Table S1. The PCR products were purified using Gel Extraction kit (CWBiotech), and the purified products were cloned into the pMD18-T Vector (6011, Takara) and transformed into Escherichia coli DH5α cells (CW0808, CWBiotech). Positive clones were confirmed by PCR. At least 10 clones for each sample were sequenced.

### Data analysis

In this study, we analyzed offspring behavioral data, which included data from every individual in a litter, using the Linear Mixed Models (LMM) procedure. We analyzed other data, which included only one individual from a litter either using ANOVA when each measure contained 3 or more sets of data, or using Independent-Samples T Test when each measure contained only two sets of data. Arc sine transformation was performed only when percentage data were not normally distributed (< 0.3 or > 0.7). For ANOVA, a Duncan multiple comparison test was used to determine differences. We used the Statistics Package for Social Science software (SPSS 20.0; SPSS Inc., Chicago, IL, USA) for data analysis. We adopted the LMM procedure because our offspring data were longitudinal (parents to offspring) data that nested litter effect. The LMM procedure takes both the fixed effects (parental stress effects) and the random effects (litter effects) into account. Random effects are considered to accommodate among-subject variation^39^. Our LMM used the variance components structure as the covariance structure. We expressed data as mean ± S.E.M. and considered differences significant when the *P* value was < 0.05. The *P* value refers to the main effect (treatment) in both the LMM procedure and ANOVA or Independent-Samples T Test.

In this study, we analyzed offspring behavioral data in Fig. 2A, B, C & D, which included data from every individual in a litter, using the Linear Mixed Models (LMM) procedure. Data in Fig. 2E-F & Fig. 7. Fig. 3D & Fig. 6 (except Adult group), which contained 3 or more groups, were analyzed using ANOVA . In Fig. 3D & Fig. 6, data which contained only two sets of data, were analyzed using Independent-Samples T Test. We tested the data normality distribution using P-P Plots method in SPSS. Arc sine transformation was performed only when percentage data were not normally distributed (< 0.3 or > 0.7). For ANOVA, a Duncan multiple comparison test was used to determine differences. We used the Statistics Package for Social Science software (SPSS 20.0; SPSS Inc., Chicago, IL, USA) for data analysis. We adopted the LMM procedure because our offspring data were longitudinal (parents to offspring) data that nested litter effect. The LMM procedure takes both the fixed effects (parental stress effects) and the random effects (litter effects) into account. Random effects are considered to accommodate among-subject variation^39^. Our LMM used the variance components structure as the covariance structure. Differential gene expression from RNA-sequencing in Dataset S1, S2 & S3 were analysed using DESeq v1.14.0. DSS-single is a statistical method for detecting differential methylation regions (DMRs) from WGBS data, used here for Dataset S8 and the adult group in Fig. 6. We expressed data as mean ± S.E.M. and considered differences significant when the *P* value was < 0.05. The *P* value refers to the main effect (treatment) in both the LMM procedure and ANOVA or Independent-Samples T Test.

## Supplementary Information

Supplementary Information 1.

Supplementary Information 2.

Supplementary Information 3.

Supplementary Information 4.

## Data Availability

The RNA-seq and WGBS data reported in this paper are available from the OMIX (https://ngdc.cncb.ac.cn/omix/release/OMIX697) and Genome Sequence Archive (https://ngdc.cncb.ac.cn/gsa/browse/CRA004404) repositories. All other data used and analyzed during the current study are available from the corresponding author by reasonable request.
