# Author Correction: A next-generation sequencing study on mechanisms by which restraint and social instability stresses of male mice alter offspring anxiety-like behavior

**DOI:** 10.1038/s41598-022-15820-4

**Published:** 2022-07-05

**Authors:** Qiao-Qiao Kong, Xiao-Dan Tian, Jia Wang, Hong-Jie Yuan, Shu-Fen Ning, Ming-Jiu Luo, Jing-He Tan

**Affiliations:** 1 Shandong Provincial Key Laboratory of Animal Biotechnology and Disease Control and Prevention, College of Animal Science and Veterinary Medicine, Shandong Agricultural University, Tai’an City, 271018 Shandong Province People’s Republic of China; 2 Tai’an City Central Hospital, Tai’an City, People’s Republic of China

Correction to: *Scientific Reports* https://doi.org/10.1038/s41598-021-87060-x, published online 12 April 2021

The original version of this Article contained errors.

Full information on the statistical tests that were carried out, and sample sizes, were not included. Therefore, in the Methods section, under the “Data analysis” subheading,

“In this study, we analyzed offspring behavioral data, which included data from every individual in a litter, using the Linear Mixed Models (LMM) procedure. We analyzed other data, which included only one individual from a litter either using ANOVA when each measure contained 3 or more sets of data, or using Independent-Samples T Test when each measure contained only two sets of data. Arc sine transformation was performed only when percentage data were not normally distributed (< 0.3 or > 0.7). For ANOVA, a Duncan multiple comparison test was used to determine differences. We used the Statistics Package for Social Science software (SPSS 20.0; SPSS Inc., Chicago, IL, USA) for data analysis. We adopted the LMM procedure because our offspring data were longitudinal (parents to offspring) data that nested litter effect. The LMM procedure takes both the fixed effects (parental stress effects) and the random effects (litter effects) into account. Random effects are considered to accommodate among-subject variation^39^. Our LMM used the variance components structure as the covariance structure. We expressed data as mean ± S.E.M. and considered differences significant when the P value was < 0.05. The P value refers to the main effect (treatment) in both the LMM procedure and ANOVA or Independent-Samples T Test.”

now reads:

“In this study, we analyzed offspring behavioral data in Fig. 2A, B, C & D, which included data from every individual in a litter, using the Linear Mixed Models (LMM) procedure. Data in Fig. 2E-F & Fig.7. Fig.3D & Fig.6 (except Adult group), which contained 3 or more groups, were analyzed using ANOVA. In Fig.3D & Fig.6, data which contained only two sets of data, were analyzed using Independent-Samples T Test. We tested the data normality distribution using P–P Plots method in SPSS. Arc sine transformation was performed only when percentage data were not normally distributed (< 0.3 or > 0.7). For ANOVA, a Duncan multiple comparison test was used to determine differences. We used the Statistics Package for Social Science software (SPSS 20.0; SPSS Inc., Chicago, IL, USA) for data analysis. We adopted the LMM procedure because our offspring data were longitudinal (parents to offspring) data that nested litter effect. The LMM procedure takes both the fixed effects (parental stress effects) and the random effects (litter effects) into account. Random effects are considered to accommodate among-subject variation^39^. Our LMM used the variance components structure as the covariance structure. Differential gene expression from RNA-sequencing in Dataset S1, S2 & S3 were analysed using DESeq v1.14.0. DSS-single is a statistical method for detecting differential methylation regions (DMRs) from WGBS data, used here for Dataset S8 and the adult group in Fig. 6. We expressed data as mean ± S.E.M. and considered differences significant when the *P* value was < 0.05. The *P* value refers to the main effect (treatment) in both the LMM procedure and ANOVA or Independent-Samples T Test.”

In addition, the Data Availability section was omitted, now reads:

“The RNA-seq and WGBS data reported in this paper are available from the OMIX (https://ngdc.cncb.ac.cn/omix/release/OMIX697) and Genome Sequence Archive (https://ngdc.cncb.ac.cn/gsa/browse/CRA004404) repositories. All other data used and analyzed during the current study are available from the corresponding author by reasonable request.”

The original Article been corrected.

