# Supplementary material for: A next-generation sequencing study on mechanisms by which restraint and social instability stresses of male mice alter offspring anxiety-like behavior
Source: Sci Rep. 2021 Apr 12;11:7952. doi: 10.1038/s41598-021-87060-x (PMC8042048; doi:10.1038/s41598-021-87060-x)
Supplement: Supplementary file 1 — Supplementary Information 1. [file 41598_2021_87060_MOESM1_ESM.pdf]

**Dataset S1. List of DE genes between RS and control (RS/C) female offspring**

| Gene | GeneNam | RS-1_cour | RS-2_cour | RS-3_cour | C-1_count | C-2_count | C-3_count | FoldChang | p | q | Up/Down |
|---|---|---|---|---|---|---|---|---|---|---|---|
| ENSMUS( | F5 | 801 | 791 | 712 | 109 | 124 | 119 | 6.469424 | 2E-199 | 5.6E-195 | up |
| ENSMUS( | Enpp2 | 41636 | 41391 | 36875 | 11044 | 10994 | 11708 | 3.511323 | 5.7E-199 | 8E-195 | up |
| ENSMUS( | Kl | 2436 | 2445 | 2152 | 779 | 727 | 814 | 2.995291 | 4E-170 | 2.8E-166 | up |
| ENSMUS( | Tmem72 | 677 | 719 | 656 | 162 | 164 | 167 | 4.117115 | 5E-126 | 2.3E-122 | up |
| ENSMUS( | Folr1 | 855 | 904 | 739 | 229 | 243 | 258 | 3.378882 | 4E-116 | 1.6E-112 | up |
| ENSMUS( | Trpm3 | 1007 | 997 | 830 | 290 | 289 | 306 | 3.159187 | 1.2E-115 | 4.3E-112 | up |
| ENSMUS( | Ace | 1024 | 988 | 909 | 312 | 324 | 322 | 3.013225 | 4.6E-110 | 1.4E-106 | up |
| ENSMUS( | Cldn2 | 489 | 490 | 412 | 87 | 95 | 94 | 4.973975 | 6.5E-109 | 1.8E-105 | up |
| ENSMUS( | Ttr | 109762 | 113318 | 105239 | 30271 | 30545 | 33592 | 3.443544 | 6E-104 | 1.5E-100 | up |
| ENSMUS( | Snhg11 | 14200 | 13849 | 11912 | 6018 | 6357 | 6623 | 2.077128 | 2.6E-100 | 6.1E-97 | up |
| ENSMUS( | Car12 | 2058 | 2046 | 1806 | 837 | 863 | 870 | 2.271305 | 2.58E-96 | 4.84E-93 | up |
| ENSMUS( | 1500015O | 826 | 985 | 828 | 239 | 215 | 263 | 3.641971 | 5.13E-96 | 9.02E-93 | up |
| ENSMUS( | Clic6 | 1097 | 1151 | 1027 | 417 | 405 | 416 | 2.614169 | 2.81E-94 | 4.39E-91 | up |
| ENSMUS( | Col8a1 | 405 | 460 | 417 | 100 | 87 | 109 | 4.291332 | 1.56E-86 | 2.19E-83 | up |
| ENSMUS( | Prlr | 711 | 772 | 712 | 232 | 255 | 285 | 2.819266 | 2.52E-82 | 3.22E-79 | up |
| ENSMUS( | Mfrp | 243 | 239 | 227 | 23 | 32 | 25 | 8.764601 | 2.83E-82 | 3.45E-79 | up |
| ENSMUS( | Pnisr | 3659 | 3631 | 3052 | 1762 | 1733 | 1755 | 1.942591 | 4.29E-79 | 5.03E-76 | up |
| ENSMUS( | Ttc14 | 3020 | 3042 | 2637 | 1445 | 1483 | 1455 | 1.95898 | 3.31E-77 | 3.73E-74 | up |
| ENSMUS( | Sostdc1 | 552 | 586 | 527 | 178 | 183 | 209 | 2.892877 | 4.37E-70 | 4.09E-67 | up |
| ENSMUS( | Nktr | 663 | 643 | 503 | 183 | 204 | 190 | 3.086269 | 9.21E-68 | 8.09E-65 | up |
| ENSMUS( | Sulf1 | 1027 | 1059 | 919 | 258 | 264 | 348 | 3.42285 | 4.23E-67 | 3.6E-64 | up |
| ENSMUS( | Htr2c | 1688 | 1765 | 1531 | 778 | 846 | 843 | 1.996348 | 2.78E-66 | 2.3E-63 | up |
| ENSMUS( | Trank1 | 3181 | 3217 | 2933 | 1623 | 1687 | 1732 | 1.830201 | 2.14E-64 | 1.67E-61 | up |
| ENSMUS( | Kcne2 | 446 | 472 | 427 | 103 | 102 | 134 | 3.935419 | 2.47E-64 | 1.88E-61 | up |
| ENSMUS( | Leng8 | 2022 | 2021 | 1726 | 943 | 1023 | 998 | 1.921128 | 8.38E-64 | 6.2E-61 | up |
| ENSMUS( | Slc4a5 | 418 | 428 | 406 | 61 | 90 | 87 | 5.220211 | 1.79E-57 | 1.23E-54 | up |
| ENSMUS( | Lbp | 883 | 853 | 752 | 248 | 318 | 309 | 2.81169 | 4.13E-54 | 2.58E-51 | up |
| ENSMUS( | Igfbp2 | 1766 | 1772 | 1685 | 987 | 933 | 963 | 1.791831 | 8.2E-50 | 4.91E-47 | up |
| ENSMUS( | Zfp445 | 2953 | 2887 | 2540 | 1565 | 1596 | 1725 | 1.694829 | 5.93E-49 | 3.47E-46 | up |
| ENSMUS( | Snrnp70 | 2675 | 2943 | 2654 | 1536 | 1493 | 1505 | 1.803892 | 8.04E-49 | 4.61E-46 | up |
| ENSMUS( | Elovl7 | 755 | 862 | 697 | 317 | 355 | 380 | 2.174394 | 3.72E-47 | 2.05E-44 | up |
| ENSMUS( | Ryr3 | 694 | 673 | 534 | 257 | 279 | 255 | 2.365462 | 6.42E-47 | 3.47E-44 | up |
| ENSMUS( | Hook1 | 1621 | 1622 | 1382 | 785 | 884 | 902 | 1.777095 | 2.58E-46 | 1.34E-43 | up |
| ENSMUS( | Slc31a1 | 1105 | 1128 | 994 | 561 | 563 | 570 | 1.881596 | 6.57E-46 | 3.36E-43 | up |
| ENSMUS( | Smg1 | 2215 | 2173 | 1956 | 1205 | 1206 | 1281 | 1.698327 | 3.55E-45 | 1.78E-42 | up |
| ENSMUS( | Lgals3bp | 718 | 679 | 597 | 308 | 304 | 346 | 2.056722 | 6.39E-43 | 2.99E-40 | up |
| ENSMUS( | Gabrb2 | 984 | 940 | 738 | 342 | 402 | 378 | 2.336733 | 1.25E-41 | 5.58E-39 | up |
| ENSMUS( | Atp11b | 1540 | 1619 | 1332 | 814 | 889 | 873 | 1.72044 | 2.2E-41 | 9.52E-39 | up |
| ENSMUS( | Eml5 | 1264 | 1157 | 1109 | 652 | 618 | 688 | 1.783324 | 6.76E-41 | 2.88E-38 | up |
| ENSMUS( | Ints6l | 1277 | 1199 | 1061 | 663 | 636 | 667 | 1.775568 | 8.13E-41 | 3.36E-38 | up |
| ENSMUS( | Aqp1 | 365 | 420 | 345 | 59 | 94 | 99 | 4.447057 | 1.27E-39 | 5.12E-37 | up |
| ENSMUS( | Zcchc7 | 1302 | 1260 | 1140 | 677 | 683 | 740 | 1.742869 | 3.4E-39 | 1.35E-36 | up |
| ENSMUS( | Steap2 | 451 | 468 | 395 | 178 | 188 | 212 | 2.247631 | 4.73E-38 | 1.82E-35 | up |
| ENSMUS( | Vat1l | 1227 | 1216 | 1022 | 612 | 656 | 697 | 1.741314 | 5.91E-38 | 2.24E-35 | up |
| ENSMUS( | Cgnl1 | 413 | 442 | 417 | 153 | 157 | 129 | 2.861806 | 6.55E-38 | 2.46E-35 | up |
| ENSMUS( | Rnpc3 | 563 | 574 | 459 | 242 | 271 | 249 | 2.063681 | 2.96E-37 | 1.07E-34 | up |
| ENSMUS( | Strip2 | 1219 | 1173 | 1017 | 642 | 636 | 663 | 1.733387 | 3.63E-37 | 1.29E-34 | up |
| ENSMUS( | Prpf39 | 995 | 1054 | 884 | 495 | 561 | 572 | 1.780243 | 4.05E-37 | 1.42E-34 | up |
| ENSMUS( | Gm6356 | 84 | 98 | 65 | 5 | 6 | 7 | 13.52893 | 1.01E-36 | 3.49E-34 | up |
| ENSMUS( | 4932438A | 2105 | 2053 | 1784 | 1133 | 1240 | 1271 | 1.610829 | 1.39E-36 | 4.77E-34 | up |
| ENSMUS( | Lmbrd2 | 343 | 301 | 262 | 102 | 109 | 115 | 2.742668 | 4.09E-36 | 1.37E-33 | up |
| ENSMUS( | Tmem181a | 2401 | 2347 | 1947 | 1285 | 1325 | 1410 | 1.643293 | 3.11E-35 | 9.93E-33 | up |
| ENSMUS( | Atp2b3 | 1050 | 1135 | 934 | 590 | 609 | 613 | 1.698756 | 3.01E-33 | 9.18E-31 | up |
| ENSMUS( | Tia1 | 2250 | 2333 | 2032 | 1416 | 1403 | 1377 | 1.556216 | 3.19E-33 | 9.64E-31 | up |
| ENSMUS( | Phf20l1 | 1563 | 1661 | 1400 | 917 | 962 | 956 | 1.610306 | 3.34E-33 | 9.98E-31 | up |
| ENSMUS( | Igf2 | 3911 | 3971 | 3672 | 2474 | 2301 | 2343 | 1.604037 | 4.32E-32 | 1.23E-29 | up |
| ENSMUS( | Mdm4 | 1470 | 1432 | 1210 | 841 | 819 | 850 | 1.615743 | 4.6E-32 | 1.29E-29 | up |
| ENSMUS( | Col11a1 | 276 | 290 | 241 | 106 | 107 | 95 | 2.581252 | 6.69E-32 | 1.84E-29 | up |
| ENSMUS( | Thoc2 | 1538 | 1494 | 1318 | 850 | 912 | 917 | 1.603865 | 8.35E-32 | 2.28E-29 | up |
| ENSMUS( | Nvl | 1172 | 1086 | 1012 | 632 | 630 | 683 | 1.662365 | 1.77E-31 | 4.73E-29 | up |
| ENSMUS( | Cab39l | 1550 | 1537 | 1389 | 925 | 938 | 896 | 1.601931 | 4.99E-31 | 1.31E-28 | up |
| ENSMUS( | Zfp871 | 233 | 249 | 183 | 72 | 72 | 81 | 2.911979 | 1.09E-30 | 2.76E-28 | up |
| ENSMUS( | Slc12a2 | 2256 | 2239 | 1969 | 1357 | 1379 | 1451 | 1.525252 | 3.21E-30 | 7.9E-28 | up |
| ENSMUS( | Prr32 | 297 | 288 | 261 | 117 | 121 | 106 | 2.425913 | 4.91E-30 | 1.2E-27 | up |
| ENSMUS( | Cldn1 | 263 | 284 | 237 | 100 | 104 | 102 | 2.528731 | 5.51E-30 | 1.34E-27 | up |
| ENSMUS( | Cep290 | 281 | 299 | 261 | 116 | 114 | 112 | 2.428066 | 7.2E-30 | 1.73E-27 | up |
| ENSMUS( | Gm10031 | 285 | 304 | 237 | 95 | 115 | 124 | 2.442841 | 1.19E-29 | 2.8E-27 | up |
| ENSMUS( | Abcc5 | 2223 | 2154 | 2072 | 1400 | 1415 | 1413 | 1.508803 | 1.21E-28 | 2.67E-26 | up |
| ENSMUS( | Ccnt2 | 1727 | 1647 | 1454 | 1038 | 963 | 1002 | 1.58623 | 2.45E-28 | 5.26E-26 | up |
| ENSMUS( | Col8a2 | 278 | 315 | 271 | 82 | 112 | 90 | 3.006469 | 6.47E-28 | 1.32E-25 | up |
| ENSMUS( | Col9a3 | 631 | 586 | 522 | 238 | 273 | 315 | 2.083854 | 7.01E-28 | 1.4E-25 | up |
| ENSMUS( | Sacs | 1864 | 1935 | 1697 | 1165 | 1189 | 1226 | 1.517047 | 6.94E-28 | 1.4E-25 | up |
| ENSMUS( | Vps13c | 1219 | 1203 | 1058 | 728 | 702 | 730 | 1.590606 | 7.58E-28 | 1.5E-25 | up |
| ENSMUS( | Srek1 | 1389 | 1424 | 1218 | 822 | 814 | 912 | 1.563339 | 8.23E-28 | 1.62E-25 | up |
| ENSMUS( | Vps13a | 850 | 788 | 699 | 445 | 463 | 460 | 1.686041 | 1.45E-27 | 2.75E-25 | up |
| ENSMUS( | Dusp11 | 1424 | 1375 | 1220 | 853 | 864 | 833 | 1.555273 | 2.33E-27 | 4.34E-25 | up |
| ENSMUS( | Usp34 | 1906 | 1787 | 1546 | 1112 | 1074 | 1132 | 1.557662 | 5.25E-27 | 9.59E-25 | up |
| ENSMUS( | Slco1c1 | 1765 | 1800 | 1524 | 1058 | 1093 | 1170 | 1.51356 | 7.01E-27 | 1.25E-24 | up |

| | | | | | | | | | | | |
|---|---|---|---|---|---|---|---|---|---|---|---|
| ENSMUS( | Sfi1 | 613 | 604 | 549 | 305 | 346 | 335 | 1.77079 | 9.55E-27 | 1.67E-24 | up |
| ENSMUS( | Ccnl1 | 1159 | 1148 | 1046 | 699 | 693 | 703 | 1.581464 | 1.16E-26 | 2.01E-24 | up |
| ENSMUS( | Pik3c2a | 530 | 537 | 444 | 265 | 255 | 297 | 1.826295 | 2.84E-26 | 4.75E-24 | up |
| ENSMUS( | Ccnl2 | 1688 | 1783 | 1606 | 1077 | 1156 | 1108 | 1.502269 | 6.9E-26 | 1.12E-23 | up |
| ENSMUS( | Tet2 | 601 | 532 | 486 | 282 | 267 | 323 | 1.835609 | 1.07E-25 | 1.72E-23 | up |
| ENSMUS( | Otx2 | 360 | 360 | 261 | 136 | 124 | 150 | 2.355196 | 1.42E-25 | 2.26E-23 | up |
| ENSMUS( | Frem1 | 214 | 186 | 190 | 63 | 81 | 72 | 2.704527 | 1.85E-25 | 2.92E-23 | up |
| ENSMUS( | Myef2 | 1172 | 1134 | 976 | 658 | 709 | 717 | 1.554766 | 8.98E-25 | 1.32E-22 | up |
| ENSMUS( | Cep162 | 425 | 436 | 356 | 215 | 182 | 200 | 2.008664 | 2.02E-24 | 2.91E-22 | up |
| ENSMUS( | Ubn2 | 940 | 887 | 780 | 469 | 527 | 570 | 1.645903 | 2.6E-24 | 3.71E-22 | up |
| ENSMUS( | Atp11c | 627 | 708 | 591 | 377 | 343 | 344 | 1.785627 | 4.54E-24 | 6.38E-22 | up |
| ENSMUS( | Atm | 243 | 247 | 191 | 88 | 86 | 107 | 2.391882 | 4.97E-24 | 6.95E-22 | up |
| ENSMUS( | Spint2 | 643 | 649 | 573 | 353 | 353 | 393 | 1.677973 | 1.91E-23 | 2.6E-21 | up |
| ENSMUS( | Sgms2 | 170 | 187 | 186 | 71 | 59 | 70 | 2.692319 | 2.19E-23 | 2.97E-21 | up |
| ENSMUS( | Abca4 | 292 | 283 | 221 | 88 | 57 | 94 | 3.28441 | 7.55E-23 | 9.97E-21 | up |
| ENSMUS( | Paxbp1 | 786 | 698 | 651 | 427 | 411 | 396 | 1.706896 | 1.01E-22 | 1.3E-20 | up |
| ENSMUS( | Focad | 644 | 613 | 482 | 295 | 312 | 331 | 1.826895 | 1.3E-22 | 1.63E-20 | up |
| ENSMUS( | Usp33 | 920 | 876 | 664 | 399 | 420 | 486 | 1.857968 | 1.93E-22 | 2.39E-20 | up |
| ENSMUS( | Trpm7 | 1308 | 1193 | 1133 | 789 | 802 | 795 | 1.504996 | 3.6E-22 | 4.38E-20 | up |
| ENSMUS( | Tmprss11a | 185 | 172 | 162 | 37 | 29 | 53 | 4.333109 | 4.93E-22 | 5.9E-20 | up |
| ENSMUS( | Fam208b | 435 | 414 | 361 | 205 | 213 | 239 | 1.820182 | 6.24E-22 | 7.43E-20 | up |
| ENSMUS( | Chuk | 758 | 729 | 688 | 442 | 433 | 474 | 1.595308 | 1.31E-21 | 1.54E-19 | up |
| ENSMUS( | Nr2c2 | 466 | 408 | 316 | 179 | 180 | 200 | 2.095393 | 1.48E-21 | 1.73E-19 | up |
| ENSMUS( | Zfc3h1 | 1082 | 1186 | 1011 | 659 | 738 | 759 | 1.503968 | 1.89E-21 | 2.17E-19 | up |
| ENSMUS( | Sema3b | 220 | 225 | 185 | 79 | 98 | 90 | 2.328527 | 2.24E-21 | 2.56E-19 | up |
| ENSMUS( | Alg10b | 400 | 387 | 344 | 188 | 198 | 223 | 1.836936 | 3E-21 | 3.4E-19 | up |
| ENSMUS( | Kcnj13 | 202 | 152 | 171 | 56 | 57 | 64 | 2.940745 | 4.31E-21 | 4.87E-19 | up |
| ENSMUS( | Hectd2 | 414 | 406 | 383 | 199 | 143 | 167 | 2.333626 | 5.2E-21 | 5.83E-19 | up |
| ENSMUS( | Zfp932 | 747 | 790 | 652 | 452 | 434 | 485 | 1.576439 | 6.41E-21 | 7.15E-19 | up |
| ENSMUS( | Wdr75 | 847 | 871 | 800 | 519 | 526 | 566 | 1.546825 | 6.76E-21 | 7.51E-19 | up |
| ENSMUS( | Mga | 1244 | 1182 | 1003 | 743 | 768 | 738 | 1.502916 | 1.39E-20 | 1.49E-18 | up |
| ENSMUS( | Eml6 | 263 | 270 | 209 | 96 | 123 | 122 | 2.147014 | 1.52E-20 | 1.62E-18 | up |
| ENSMUS( | Unc13b | 780 | 754 | 597 | 382 | 438 | 429 | 1.6814 | 1.79E-20 | 1.87E-18 | up |
| ENSMUS( | Lnpep | 194 | 207 | 153 | 69 | 83 | 74 | 2.412673 | 2.72E-20 | 2.77E-18 | up |
| ENSMUS( | Trpv4 | 167 | 148 | 143 | 54 | 52 | 62 | 2.698911 | 3.91E-20 | 3.89E-18 | up |
| ENSMUS( | Pcolce2 | 256 | 240 | 196 | 111 | 101 | 107 | 2.136034 | 7.06E-20 | 6.84E-18 | up |
| ENSMUS( | Zkscan3 | 815 | 724 | 683 | 424 | 474 | 431 | 1.651075 | 8.97E-20 | 8.52E-18 | up |
| ENSMUS( | Stx3 | 771 | 698 | 711 | 402 | 449 | 482 | 1.621669 | 1.11E-19 | 1.05E-17 | up |
| ENSMUS( | Zfp322a | 833 | 816 | 749 | 480 | 527 | 541 | 1.532458 | 1.29E-19 | 1.21E-17 | up |
| ENSMUS( | Zkscan16 | 281 | 276 | 202 | 76 | 84 | 109 | 2.785101 | 1.5E-19 | 1.4E-17 | up |
| ENSMUS( | Calml4 | 371 | 439 | 383 | 222 | 190 | 212 | 1.890229 | 1.73E-19 | 1.61E-17 | up |
| ENSMUS( | Sfrp1 | 410 | 380 | 382 | 212 | 216 | 231 | 1.761837 | 2.09E-19 | 1.92E-17 | up |
| ENSMUS( | Oca2 | 144 | 130 | 117 | 46 | 42 | 46 | 2.879819 | 3.36E-19 | 3.03E-17 | up |
| ENSMUS( | Sh3d19 | 514 | 522 | 444 | 278 | 308 | 299 | 1.650981 | 4.02E-19 | 3.61E-17 | up |
| ENSMUS( | Klhl34 | 479 | 466 | 393 | 265 | 262 | 257 | 1.682176 | 5.66E-19 | 5.02E-17 | up |
| ENSMUS( | C2cd5 | 825 | 915 | 800 | 536 | 528 | 604 | 1.507171 | 7.03E-19 | 6.18E-17 | up |
| ENSMUS( | Atp7a | 99 | 101 | 79 | 22 | 27 | 27 | 3.621879 | 9.97E-19 | 8.55E-17 | up |
| ENSMUS( | Brwd3 | 431 | 542 | 415 | 229 | 254 | 259 | 1.847223 | 1.39E-18 | 1.18E-16 | up |
| ENSMUS( | Guf1 | 727 | 792 | 689 | 445 | 493 | 490 | 1.528898 | 1.44E-18 | 1.22E-16 | up |
| ENSMUS( | Pnn | 1806 | 1783 | 1538 | 1002 | 1184 | 1190 | 1.500836 | 1.54E-18 | 1.29E-16 | up |
| ENSMUS( | Ctnnal1 | 470 | 462 | 385 | 259 | 255 | 263 | 1.67136 | 2.39E-18 | 1.97E-16 | up |
| ENSMUS( | Bst2 | 202 | 221 | 239 | 92 | 80 | 102 | 2.404219 | 2.9E-18 | 2.35E-16 | up |
| ENSMUS( | Lepr | 222 | 202 | 180 | 101 | 86 | 85 | 2.187039 | 4.02E-18 | 3.17E-16 | up |
| ENSMUS( | Car14 | 285 | 283 | 238 | 143 | 119 | 148 | 1.940425 | 7.26E-18 | 5.61E-16 | up |
| ENSMUS( | Wdr86 | 147 | 153 | 129 | 52 | 54 | 58 | 2.584281 | 7.31E-18 | 5.63E-16 | up |
| ENSMUS( | Fam178a | 993 | 980 | 902 | 587 | 593 | 698 | 1.516093 | 8.07E-18 | 6.2E-16 | up |
| ENSMUS( | Dnmt3a | 797 | 789 | 678 | 450 | 500 | 535 | 1.506925 | 8.79E-18 | 6.73E-16 | up |
| ENSMUS( | Ccdc39 | 509 | 468 | 482 | 280 | 270 | 247 | 1.810074 | 9.38E-18 | 7.15E-16 | up |
| ENSMUS( | Pqlc3 | 195 | 228 | 190 | 88 | 97 | 99 | 2.134191 | 1.18E-17 | 8.92E-16 | up |
| ENSMUS( | Zfp397 | 845 | 790 | 680 | 497 | 518 | 505 | 1.501903 | 1.52E-17 | 1.15E-15 | up |
| ENSMUS( | Flnb | 579 | 587 | 481 | 340 | 351 | 343 | 1.570209 | 2.35E-17 | 1.73E-15 | up |
| ENSMUS( | Kcnh7 | 577 | 615 | 530 | 349 | 353 | 388 | 1.562025 | 2.51E-17 | 1.83E-15 | up |
| ENSMUS( | Ints13 | 645 | 639 | 587 | 393 | 385 | 424 | 1.539777 | 2.98E-17 | 2.17E-15 | up |
| ENSMUS( | Cspp1 | 517 | 509 | 449 | 316 | 302 | 293 | 1.597088 | 4.36E-17 | 3.06E-15 | up |
| ENSMUS( | Prkdc | 531 | 609 | 530 | 325 | 350 | 358 | 1.599564 | 4.55E-17 | 3.19E-15 | up |
| ENSMUS( | Itga8 | 502 | 493 | 390 | 277 | 258 | 285 | 1.663978 | 6.16E-17 | 4.27E-15 | up |
| ENSMUS( | Wdr78 | 223 | 240 | 201 | 101 | 103 | 121 | 2.02074 | 6.48E-17 | 4.47E-15 | up |
| ENSMUS( | Dock7 | 727 | 726 | 620 | 450 | 434 | 477 | 1.503929 | 7.99E-17 | 5.45E-15 | up |
| ENSMUS( | Gm15446 | 511 | 547 | 516 | 339 | 321 | 330 | 1.57327 | 9.86E-17 | 6.7E-15 | up |
| ENSMUS( | Slc2a12 | 486 | 427 | 470 | 151 | 180 | 221 | 2.494041 | 9.98E-17 | 6.76E-15 | up |
| ENSMUS( | Zfp26 | 359 | 413 | 371 | 202 | 229 | 226 | 1.722795 | 1.08E-16 | 7.28E-15 | up |
| ENSMUS( | Xkr4 | 212 | 200 | 164 | 78 | 92 | 99 | 2.114335 | 1.99E-16 | 1.28E-14 | up |
| ENSMUS( | Npr3 | 638 | 581 | 609 | 374 | 379 | 375 | 1.605153 | 3.35E-16 | 2.09E-14 | up |
| ENSMUS( | Cul4b | 1057 | 1052 | 914 | 681 | 582 | 663 | 1.549204 | 3.48E-16 | 2.16E-14 | up |
| ENSMUS( | Zmym6 | 542 | 495 | 475 | 316 | 303 | 338 | 1.563033 | 6.59E-16 | 3.93E-14 | up |
| ENSMUS( | Krt18 | 200 | 210 | 158 | 94 | 84 | 90 | 2.085362 | 6.83E-16 | 4.06E-14 | up |
| ENSMUS( | Tbc1d32 | 348 | 373 | 353 | 218 | 192 | 194 | 1.758107 | 8.44E-16 | 4.97E-14 | up |
| ENSMUS( | Mndal | 138 | 112 | 117 | 48 | 38 | 53 | 2.61623 | 8.48E-16 | 4.98E-14 | up |
| ENSMUS( | Drc7 | 228 | 267 | 232 | 134 | 120 | 129 | 1.876288 | 1.83E-15 | 1.04E-13 | up |
| ENSMUS( | Rdh5 | 200 | 202 | 192 | 100 | 89 | 80 | 2.179306 | 1.84E-15 | 1.04E-13 | up |

| | | | | | | | | | | | |
|---|---|---|---|---|---|---|---|---|---|---|---|
| ENSMUS( | Fras1 | 206 | 231 | 222 | 99 | 114 | 89 | 2.158325 | 1.86E-15 | 1.05E-13 | up |
| ENSMUS( | Wfdc2 | 137 | 137 | 125 | 49 | 51 | 61 | 2.455352 | 2.09E-15 | 1.18E-13 | up |
| ENSMUS( | Kif13b | 137 | 117 | 117 | 50 | 50 | 44 | 2.543086 | 2.26E-15 | 1.27E-13 | up |
| ENSMUS( | Nbeal1 | 591 | 606 | 561 | 292 | 375 | 358 | 1.698767 | 2.4E-15 | 1.33E-13 | up |
| ENSMUS( | Zfyve16 | 512 | 433 | 407 | 256 | 255 | 285 | 1.678625 | 2.45E-15 | 1.36E-13 | up |
| ENSMUS( | Nrp1 | 639 | 600 | 531 | 376 | 374 | 411 | 1.50602 | 2.46E-15 | 1.36E-13 | up |
| ENSMUS( | Usp40 | 377 | 358 | 315 | 207 | 211 | 204 | 1.665332 | 3.32E-15 | 1.78E-13 | up |
| ENSMUS( | Slc13a4 | 578 | 604 | 566 | 372 | 395 | 383 | 1.504011 | 4.93E-15 | 2.58E-13 | up |
| ENSMUS( | Zfp329 | 506 | 456 | 432 | 265 | 313 | 291 | 1.585753 | 5.58E-15 | 2.89E-13 | up |
| ENSMUS( | Hipk2 | 166 | 158 | 142 | 65 | 64 | 78 | 2.227715 | 5.64E-15 | 2.92E-13 | up |
| ENSMUS( | Celsr3 | 574 | 588 | 516 | 356 | 364 | 378 | 1.510131 | 5.66E-15 | 2.93E-13 | up |
| ENSMUS( | Krr1 | 518 | 519 | 510 | 344 | 310 | 348 | 1.529117 | 8.54E-15 | 4.35E-13 | up |
| ENSMUS( | Tbc1d8b | 428 | 421 | 396 | 221 | 259 | 277 | 1.629827 | 8.59E-15 | 4.37E-13 | up |
| ENSMUS( | Zmym1 | 254 | 290 | 238 | 133 | 110 | 149 | 1.972871 | 1.05E-14 | 5.24E-13 | up |
| ENSMUS( | Mdn1 | 465 | 480 | 513 | 285 | 260 | 311 | 1.692693 | 1.12E-14 | 5.57E-13 | up |
| ENSMUS( | Iqgap1 | 471 | 466 | 442 | 282 | 259 | 235 | 1.754046 | 1.2E-14 | 5.94E-13 | up |
| ENSMUS( | Pxdn | 787 | 793 | 691 | 464 | 526 | 465 | 1.540271 | 1.24E-14 | 6.12E-13 | up |
| ENSMUS( | Ncam2 | 498 | 406 | 368 | 229 | 242 | 251 | 1.738276 | 1.38E-14 | 6.73E-13 | up |
| ENSMUS( | Slc16a6 | 160 | 182 | 140 | 82 | 65 | 75 | 2.138479 | 2.07E-14 | 9.91E-13 | up |
| ENSMUS( | Myo9b | 484 | 512 | 454 | 316 | 290 | 331 | 1.530289 | 2.74E-14 | 1.31E-12 | up |
| ENSMUS( | Sacm1l | 581 | 579 | 439 | 279 | 268 | 337 | 1.784019 | 3.82E-14 | 1.78E-12 | up |
| ENSMUS( | Rreb1 | 964 | 866 | 742 | 527 | 449 | 493 | 1.724771 | 3.83E-14 | 1.78E-12 | up |
| ENSMUS( | Sorbs1 | 544 | 545 | 459 | 320 | 336 | 359 | 1.506121 | 4.29E-14 | 1.98E-12 | up |
| ENSMUS( | Cd46 | 186 | 181 | 152 | 88 | 73 | 91 | 2.032368 | 7.87E-14 | 3.56E-12 | up |
| ENSMUS( | Ints2 | 442 | 404 | 372 | 240 | 272 | 256 | 1.56659 | 8.01E-14 | 3.62E-12 | up |
| ENSMUS( | Zfp970 | 288 | 304 | 273 | 158 | 174 | 170 | 1.704106 | 8.04E-14 | 3.63E-12 | up |
| ENSMUS( | Col19a1 | 124 | 93 | 80 | 35 | 30 | 30 | 3.070405 | 8.06E-14 | 3.63E-12 | up |
| ENSMUS( | Kcna1 | 301 | 313 | 259 | 146 | 177 | 148 | 1.827346 | 9.06E-14 | 4.07E-12 | up |
| ENSMUS( | Zc3h11a | 69 | 65 | 59 | 22 | 16 | 14 | 3.647683 | 1.33E-13 | 5.86E-12 | up |
| ENSMUS( | Sec61a2 | 662 | 664 | 558 | 396 | 439 | 391 | 1.514913 | 1.35E-13 | 5.91E-12 | up |
| ENSMUS( | Ryr1 | 196 | 187 | 200 | 94 | 103 | 103 | 1.928552 | 1.73E-13 | 7.52E-12 | up |
| ENSMUS( | Abca8b | 571 | 518 | 562 | 336 | 349 | 347 | 1.586353 | 1.74E-13 | 7.54E-12 | up |
| ENSMUS( | Zbtb16 | 260 | 283 | 239 | 137 | 143 | 165 | 1.738629 | 1.94E-13 | 8.35E-12 | up |
| ENSMUS( | Postn | 213 | 170 | 163 | 88 | 87 | 84 | 2.079105 | 2.16E-13 | 9.2E-12 | up |
| ENSMUS( | Zfp950 | 555 | 586 | 493 | 362 | 370 | 338 | 1.505637 | 2.31E-13 | 9.82E-12 | up |
| ENSMUS( | Ago3 | 987 | 990 | 862 | 622 | 651 | 534 | 1.547868 | 2.87E-13 | 1.21E-11 | up |
| ENSMUS( | Col4a3 | 53 | 62 | 39 | 13 | 11 | 11 | 4.310535 | 3.28E-13 | 1.37E-11 | up |
| ENSMUS( | AC153134 | 372 | 363 | 307 | 219 | 186 | 223 | 1.637256 | 3.52E-13 | 1.46E-11 | up |
| ENSMUS( | Dennd1b | 411 | 389 | 341 | 246 | 235 | 241 | 1.558974 | 5.4E-13 | 2.17E-11 | up |
| ENSMUS( | Spaca6 | 363 | 335 | 301 | 159 | 204 | 155 | 1.901458 | 5.77E-13 | 2.31E-11 | up |
| ENSMUS( | Ercc6l2 | 460 | 508 | 400 | 301 | 298 | 299 | 1.50154 | 7.21E-13 | 2.84E-11 | up |
| ENSMUS( | Lrriq1 | 86 | 96 | 78 | 34 | 34 | 25 | 2.748421 | 8.09E-13 | 3.17E-11 | up |
| ENSMUS( | Fam120c | 496 | 452 | 351 | 255 | 264 | 255 | 1.65036 | 9.31E-13 | 3.61E-11 | up |
| ENSMUS( | Plekhn1 | 438 | 458 | 395 | 286 | 277 | 280 | 1.51172 | 1.2E-12 | 4.61E-11 | up |
| ENSMUS( | Slc4a2 | 734 | 662 | 555 | 326 | 406 | 380 | 1.730328 | 1.22E-12 | 4.68E-11 | up |
| ENSMUS( | Usp28 | 380 | 405 | 358 | 226 | 257 | 225 | 1.594527 | 1.28E-12 | 4.85E-11 | up |
| ENSMUS( | Gabpb2 | 420 | 367 | 348 | 237 | 243 | 245 | 1.546572 | 2.28E-12 | 8.37E-11 | up |
| ENSMUS( | Rsbn1 | 310 | 333 | 272 | 194 | 170 | 195 | 1.61508 | 2.47E-12 | 9.01E-11 | up |
| ENSMUS( | Msx1 | 349 | 386 | 337 | 232 | 222 | 227 | 1.555313 | 3.22E-12 | 1.15E-10 | up |
| ENSMUS( | Adam17 | 308 | 311 | 258 | 182 | 166 | 155 | 1.716243 | 3.36E-12 | 1.2E-10 | up |
| ENSMUS( | Ankrd26 | 284 | 285 | 251 | 169 | 164 | 157 | 1.650976 | 4.21E-12 | 1.47E-10 | up |
| ENSMUS( | Zkscan2 | 491 | 413 | 442 | 270 | 295 | 274 | 1.58821 | 4.88E-12 | 1.71E-10 | up |
| ENSMUS( | Lrrc45 | 414 | 386 | 330 | 239 | 238 | 252 | 1.529169 | 5.17E-12 | 1.8E-10 | up |
| ENSMUS( | Steap1 | 156 | 112 | 125 | 35 | 26 | 16 | 5.007803 | 5.52E-12 | 1.91E-10 | up |
| ENSMUS( | Slc16a4 | 192 | 213 | 175 | 82 | 111 | 100 | 1.956109 | 5.57E-12 | 1.93E-10 | up |
| ENSMUS( | Fos | 311 | 327 | 313 | 204 | 195 | 195 | 1.58414 | 8.18E-12 | 2.76E-10 | up |
| ENSMUS( | Gm3411 | 51 | 46 | 39 | 6 | 13 | 11 | 4.485491 | 9.79E-12 | 3.28E-10 | up |
| ENSMUS( | Gm3636 | 94 | 109 | 80 | 38 | 32 | 43 | 2.470823 | 1E-11 | 3.34E-10 | up |
| ENSMUS( | Cfap54 | 419 | 422 | 411 | 279 | 266 | 247 | 1.562652 | 1.11E-11 | 3.68E-10 | up |
| ENSMUS( | Slc7a6 | 381 | 366 | 346 | 223 | 249 | 234 | 1.530886 | 1.15E-11 | 3.81E-10 | up |
| ENSMUS( | Scube3 | 85 | 100 | 81 | 36 | 25 | 39 | 2.630649 | 1.67E-11 | 5.42E-10 | up |
| ENSMUS( | Mtmr9 | 412 | 411 | 360 | 249 | 241 | 289 | 1.502055 | 1.74E-11 | 5.63E-10 | up |
| ENSMUS( | Heatr1 | 496 | 452 | 378 | 277 | 273 | 308 | 1.524509 | 2.08E-11 | 6.65E-10 | up |
| ENSMUS( | Npas4 | 146 | 135 | 141 | 70 | 49 | 54 | 2.410593 | 2.7E-11 | 8.49E-10 | up |
| ENSMUS( | Nsun7 | 180 | 161 | 160 | 94 | 88 | 82 | 1.874036 | 3.51E-11 | 1.09E-09 | up |
| ENSMUS( | Zbtb37 | 147 | 153 | 90 | 44 | 57 | 57 | 2.421844 | 4.39E-11 | 1.35E-09 | up |
| ENSMUS( | Cfap69 | 259 | 275 | 287 | 148 | 170 | 157 | 1.715426 | 5.19E-11 | 1.57E-09 | up |
| ENSMUS( | Stxbp5l | 288 | 278 | 238 | 119 | 121 | 175 | 1.920116 | 6.51E-11 | 1.93E-09 | up |
| ENSMUS( | Atr | 197 | 201 | 177 | 107 | 98 | 116 | 1.771156 | 6.84E-11 | 2.02E-09 | up |
| ENSMUS( | Adamts1 | 359 | 351 | 281 | 213 | 203 | 223 | 1.528447 | 6.9E-11 | 2.03E-09 | up |
| ENSMUS( | Krt8 | 158 | 121 | 109 | 57 | 47 | 66 | 2.253189 | 7.05E-11 | 2.07E-09 | up |
| ENSMUS( | Ap4e1 | 143 | 146 | 140 | 79 | 61 | 76 | 1.965803 | 7.48E-11 | 2.19E-09 | up |
| ENSMUS( | Zfp507 | 178 | 190 | 155 | 93 | 103 | 88 | 1.814449 | 8.81E-11 | 2.56E-09 | up |
| ENSMUS( | D130043K | 265 | 308 | 265 | 164 | 180 | 178 | 1.587788 | 9.02E-11 | 2.62E-09 | up |
| ENSMUS( | Cfp | 166 | 174 | 128 | 70 | 83 | 92 | 1.884333 | 1.06E-10 | 3.04E-09 | up |
| ENSMUS( | St6galnac2 | 99 | 102 | 69 | 18 | 29 | 36 | 3.215302 | 1.1E-10 | 3.17E-09 | up |
| ENSMUS( | Mdfic | 246 | 303 | 263 | 150 | 175 | 155 | 1.673205 | 1.66E-10 | 4.66E-09 | up |
| ENSMUS( | Ankrd16 | 411 | 394 | 309 | 239 | 245 | 228 | 1.538902 | 1.73E-10 | 4.82E-09 | up |
| ENSMUS( | Akap17b | 410 | 423 | 386 | 231 | 286 | 269 | 1.534902 | 2.07E-10 | 5.73E-09 | up |
| ENSMUS( | Cdc7 | 350 | 368 | 346 | 185 | 236 | 235 | 1.608283 | 2.62E-10 | 7.11E-09 | up |

| | | | | | | | | | | | |
|---|---|---|---|---|---|---|---|---|---|---|---|
| ENSMUS( | Zfp974 | 209 | 226 | 191 | 117 | 127 | 122 | 1.688893 | 2.64E-10 | 7.16E-09 | up |
| ENSMUS( | Col11a2 | 285 | 282 | 226 | 156 | 171 | 167 | 1.582091 | 2.7E-10 | 7.32E-09 | up |
| ENSMUS( | Tarbp1 | 220 | 206 | 199 | 108 | 120 | 138 | 1.693149 | 3.03E-10 | 8.11E-09 | up |
| ENSMUS( | Nos1ap | 396 | 414 | 309 | 168 | 231 | 200 | 1.840146 | 3.05E-10 | 8.15E-09 | up |
| ENSMUS( | Med12 | 285 | 236 | 235 | 131 | 161 | 160 | 1.655642 | 3.12E-10 | 8.3E-09 | up |
| ENSMUS( | Klhl11 | 161 | 143 | 125 | 65 | 77 | 55 | 2.141823 | 3.12E-10 | 8.3E-09 | up |
| ENSMUS( | Tenm1 | 427 | 419 | 302 | 204 | 178 | 235 | 1.83215 | 3.33E-10 | 8.77E-09 | up |
| ENSMUS( | Thbs3 | 212 | 210 | 193 | 126 | 118 | 116 | 1.686835 | 4.05E-10 | 1.05E-08 | up |
| ENSMUS( | Rnf32 | 423 | 360 | 351 | 235 | 232 | 275 | 1.512865 | 4.09E-10 | 1.06E-08 | up |
| ENSMUS( | Pus10 | 296 | 294 | 270 | 159 | 191 | 200 | 1.548736 | 4.89E-10 | 1.25E-08 | up |
| ENSMUS( | Abhd18 | 339 | 304 | 294 | 182 | 212 | 217 | 1.518234 | 5.32E-10 | 1.35E-08 | up |
| ENSMUS( | Col5a2 | 143 | 150 | 131 | 82 | 68 | 71 | 1.892359 | 6.03E-10 | 1.52E-08 | up |
| ENSMUS( | F11r | 234 | 236 | 235 | 120 | 146 | 158 | 1.651349 | 6.28E-10 | 1.58E-08 | up |
| ENSMUS( | Robo3 | 164 | 169 | 143 | 86 | 87 | 86 | 1.813322 | 6.7E-10 | 1.67E-08 | up |
| ENSMUS( | Ttc37 | 312 | 342 | 256 | 195 | 184 | 199 | 1.550588 | 7.45E-10 | 1.84E-08 | up |
| ENSMUS( | Klhl3 | 147 | 145 | 118 | 61 | 80 | 72 | 1.898837 | 9.7E-10 | 2.35E-08 | up |
| ENSMUS( | Zfp185 | 162 | 165 | 163 | 90 | 64 | 59 | 2.268414 | 1.09E-09 | 2.61E-08 | up |
| ENSMUS( | Ttll4 | 311 | 295 | 271 | 183 | 196 | 190 | 1.522813 | 1.12E-09 | 2.68E-08 | up |
| ENSMUS( | Pkd1 | 851 | 746 | 643 | 423 | 506 | 513 | 1.533454 | 1.18E-09 | 2.8E-08 | up |
| ENSMUS( | Spg11 | 198 | 197 | 152 | 101 | 100 | 114 | 1.711697 | 1.35E-09 | 3.18E-08 | up |
| ENSMUS( | Szt2 | 279 | 302 | 253 | 184 | 177 | 176 | 1.53216 | 1.47E-09 | 3.44E-08 | up |
| ENSMUS( | Mc3r | 33 | 22 | 30 | 7 | 4 | 4 | 5.595456 | 1.76E-09 | 4.08E-08 | up |
| ENSMUS( | Slc16a9 | 64 | 56 | 61 | 24 | 16 | 25 | 2.763771 | 1.88E-09 | 4.33E-08 | up |
| ENSMUS( | Angptl2 | 165 | 148 | 127 | 68 | 79 | 91 | 1.827734 | 2.13E-09 | 4.86E-08 | up |
| ENSMUS( | Defb11 | 49 | 64 | 41 | 13 | 18 | 9 | 3.765406 | 2.14E-09 | 4.88E-08 | up |
| ENSMUS( | Serac1 | 190 | 226 | 187 | 120 | 120 | 120 | 1.654263 | 2.15E-09 | 4.92E-08 | up |
| ENSMUS( | Ackr4 | 39 | 35 | 26 | 9 | 8 | 5 | 4.437359 | 2.37E-09 | 5.35E-08 | up |
| ENSMUS( | Rrh | 36 | 24 | 42 | 5 | 6 | 8 | 5.394878 | 2.43E-09 | 5.48E-08 | up |
| ENSMUS( | Scara5 | 43 | 47 | 51 | 15 | 11 | 17 | 3.265754 | 2.47E-09 | 5.54E-08 | up |
| ENSMUS( | Dcc | 59 | 59 | 32 | 14 | 14 | 13 | 3.571098 | 2.48E-09 | 5.56E-08 | up |
| ENSMUS( | Kcnma1 | 430 | 397 | 305 | 239 | 240 | 244 | 1.539995 | 2.74E-09 | 6.11E-08 | up |
| ENSMUS( | Cox17 | 94 | 91 | 70 | 39 | 36 | 26 | 2.473003 | 3.19E-09 | 7E-08 | up |
| ENSMUS( | Rgs11 | 262 | 241 | 270 | 117 | 155 | 149 | 1.825661 | 3.57E-09 | 7.76E-08 | up |
| ENSMUS( | Nxpe4 | 277 | 305 | 244 | 181 | 161 | 195 | 1.518624 | 3.69E-09 | 7.99E-08 | up |
| ENSMUS( | B3galnt2 | 308 | 259 | 222 | 160 | 155 | 160 | 1.635927 | 4.1E-09 | 8.82E-08 | up |
| ENSMUS( | Atad2 | 208 | 210 | 175 | 115 | 109 | 132 | 1.645696 | 4.14E-09 | 8.91E-08 | up |
| ENSMUS( | Mccc1 | 294 | 285 | 228 | 163 | 182 | 178 | 1.52064 | 4.28E-09 | 9.17E-08 | up |
| ENSMUS( | Odf2l | 237 | 269 | 236 | 163 | 158 | 151 | 1.55275 | 4.35E-09 | 9.31E-08 | up |
| ENSMUS( | Alms1 | 148 | 124 | 136 | 54 | 63 | 82 | 2.040692 | 4.54E-09 | 9.68E-08 | up |
| ENSMUS( | Gm14410 | 245 | 273 | 227 | 155 | 153 | 167 | 1.549642 | 4.82E-09 | 1.02E-07 | up |
| ENSMUS( | Mitf | 331 | 260 | 292 | 175 | 182 | 183 | 1.620149 | 5.45E-09 | 1.14E-07 | up |
| ENSMUS( | Uprt | 50 | 41 | 36 | 13 | 14 | 10 | 3.370432 | 5.73E-09 | 1.19E-07 | up |
| ENSMUS( | Ccdc84 | 196 | 206 | 168 | 111 | 105 | 126 | 1.646251 | 7.89E-09 | 1.61E-07 | up |
| ENSMUS( | N4bp2 | 289 | 312 | 278 | 198 | 155 | 184 | 1.617426 | 8.4E-09 | 1.71E-07 | up |
| ENSMUS( | Cep135 | 218 | 232 | 197 | 111 | 137 | 147 | 1.621379 | 8.46E-09 | 1.72E-07 | up |
| ENSMUS( | Faxc | 260 | 229 | 217 | 124 | 152 | 163 | 1.592659 | 8.82E-09 | 1.78E-07 | up |
| ENSMUS( | Fam135b | 108 | 93 | 70 | 45 | 37 | 38 | 2.21459 | 1.09E-08 | 2.15E-07 | up |
| ENSMUS( | Ankzf1 | 253 | 245 | 255 | 172 | 158 | 148 | 1.558721 | 1.71E-08 | 3.25E-07 | up |
| ENSMUS( | Tubgcp4 | 201 | 227 | 203 | 120 | 102 | 143 | 1.714334 | 1.95E-08 | 3.67E-07 | up |
| ENSMUS( | Ccdc141 | 327 | 339 | 295 | 198 | 231 | 195 | 1.51977 | 2E-08 | 3.77E-07 | up |
| ENSMUS( | Zfp459 | 59 | 60 | 39 | 19 | 23 | 15 | 2.709963 | 2.1E-08 | 3.94E-07 | up |
| ENSMUS( | Cntnap5b | 202 | 233 | 136 | 110 | 96 | 99 | 1.832172 | 2.29E-08 | 4.28E-07 | up |
| ENSMUS( | Slc8a1 | 183 | 189 | 124 | 92 | 89 | 84 | 1.834356 | 2.58E-08 | 4.77E-07 | up |
| ENSMUS( | Fhad1 | 155 | 147 | 140 | 70 | 87 | 95 | 1.739327 | 2.95E-08 | 5.36E-07 | up |
| ENSMUS( | BC005561 | 73 | 86 | 65 | 27 | 33 | 39 | 2.238354 | 3.11E-08 | 5.64E-07 | up |
| ENSMUS( | Chrna7 | 203 | 199 | 223 | 96 | 124 | 96 | 1.962715 | 3.19E-08 | 5.77E-07 | up |
| ENSMUS( | Htr4 | 217 | 204 | 178 | 117 | 127 | 129 | 1.585618 | 3.38E-08 | 6.1E-07 | up |
| ENSMUS( | Xpo4 | 107 | 126 | 95 | 59 | 46 | 65 | 1.904739 | 3.71E-08 | 6.64E-07 | up |
| ENSMUS( | Yeats2 | 432 | 463 | 338 | 222 | 276 | 278 | 1.566248 | 4.65E-08 | 8.16E-07 | up |
| ENSMUS( | Zfp995 | 158 | 138 | 154 | 96 | 79 | 83 | 1.727203 | 4.8E-08 | 8.38E-07 | up |
| ENSMUS( | Fndc1 | 161 | 146 | 146 | 75 | 90 | 98 | 1.708625 | 5.03E-08 | 8.74E-07 | up |
| ENSMUS( | Rbm47 | 62 | 74 | 58 | 21 | 22 | 34 | 2.500237 | 5.91E-08 | 1.01E-06 | up |
| ENSMUS( | Uvssa | 195 | 206 | 158 | 110 | 106 | 130 | 1.594347 | 6.75E-08 | 1.15E-06 | up |
| ENSMUS( | Mcm6 | 315 | 258 | 235 | 148 | 158 | 192 | 1.604479 | 7.51E-08 | 1.27E-06 | up |
| ENSMUS( | Pdpr | 268 | 219 | 213 | 143 | 146 | 153 | 1.564931 | 7.96E-08 | 1.33E-06 | up |
| ENSMUS( | Tnfrsf25 | 127 | 144 | 107 | 77 | 67 | 67 | 1.761582 | 1.12E-07 | 1.83E-06 | up |
| ENSMUS( | Zfp955a | 161 | 185 | 107 | 60 | 34 | 54 | 2.997481 | 1.12E-07 | 1.83E-06 | up |
| ENSMUS( | Spag16 | 137 | 132 | 134 | 70 | 42 | 74 | 2.15009 | 1.13E-07 | 1.85E-06 | up |
| ENSMUS( | Slc39a4 | 111 | 107 | 63 | 39 | 46 | 38 | 2.233127 | 1.18E-07 | 1.91E-06 | up |
| ENSMUS( | Ifi203 | 109 | 111 | 105 | 52 | 56 | 65 | 1.863156 | 1.26E-07 | 2.02E-06 | up |
| ENSMUS( | Ocln | 316 | 326 | 291 | 172 | 225 | 210 | 1.520977 | 1.29E-07 | 2.07E-06 | up |
| ENSMUS( | Sptlc3 | 22 | 25 | 26 | 5 | 4 | 6 | 4.846205 | 1.46E-07 | 2.31E-06 | up |
| ENSMUS( | Ttf1 | 216 | 197 | 167 | 126 | 112 | 130 | 1.554219 | 1.64E-07 | 2.59E-06 | up |
| ENSMUS( | Tmem74 | 171 | 167 | 143 | 80 | 90 | 112 | 1.688975 | 1.7E-07 | 2.67E-06 | up |
| ENSMUS( | Bbof1 | 115 | 101 | 113 | 64 | 55 | 59 | 1.831822 | 2.04E-07 | 3.16E-06 | up |
| ENSMUS( | Stab2 | 90 | 58 | 66 | 15 | 24 | 30 | 3.085711 | 2.16E-07 | 3.34E-06 | up |
| ENSMUS( | Perp | 202 | 247 | 209 | 122 | 121 | 159 | 1.623359 | 2.28E-07 | 3.51E-06 | up |
| ENSMUS( | Gm14391 | 91 | 92 | 80 | 44 | 38 | 26 | 2.39042 | 2.28E-07 | 3.51E-06 | up |
| ENSMUS( | Slc9a7 | 66 | 78 | 73 | 38 | 29 | 34 | 2.126744 | 2.45E-07 | 3.75E-06 | up |
| ENSMUS( | Vcl | 205 | 189 | 156 | 109 | 127 | 111 | 1.560811 | 2.49E-07 | 3.79E-06 | up |

| | | | | | | | | | | | | |
|---|---|---|---|---|---|---|---|---|---|---|---|---|
| ENSMUS( | Gprin3 | 157 | 168 | 135 | 80 | 86 | 109 | 1.655157 | 2.65E-07 | 4.02E-06 | up |
| ENSMUS( | Myo5c | 69 | 65 | 72 | 21 | 21 | 36 | 2.639145 | 2.78E-07 | 4.2E-06 | up |
| ENSMUS( | 4930447C( | 118 | 139 | 85 | 60 | 53 | 58 | 1.961246 | 2.86E-07 | 4.31E-06 | up |
| ENSMUS( | Polq | 78 | 89 | 89 | 47 | 42 | 39 | 1.979879 | 2.89E-07 | 4.35E-06 | up |
| ENSMUS( | Spef2 | 129 | 159 | 133 | 69 | 83 | 96 | 1.683509 | 2.9E-07 | 4.37E-06 | up |
| ENSMUS( | Tubgcp6 | 204 | 206 | 179 | 116 | 135 | 128 | 1.535195 | 2.98E-07 | 4.47E-06 | up |
| ENSMUS( | Adamts10 | 190 | 190 | 152 | 109 | 105 | 120 | 1.571082 | 2.99E-07 | 4.48E-06 | up |
| ENSMUS( | Myo7a | 75 | 109 | 80 | 44 | 43 | 37 | 2.096339 | 3.26E-07 | 4.85E-06 | up |
| ENSMUS( | Serpinb8 | 93 | 103 | 73 | 47 | 45 | 32 | 2.124156 | 3.28E-07 | 4.88E-06 | up |
| ENSMUS( | Glb1l | 130 | 120 | 103 | 65 | 44 | 67 | 1.980034 | 3.34E-07 | 4.96E-06 | up |
| ENSMUS( | Hdx | 168 | 162 | 142 | 89 | 90 | 109 | 1.621333 | 3.4E-07 | 5.05E-06 | up |
| ENSMUS( | Slc37a2 | 61 | 60 | 56 | 25 | 20 | 31 | 2.310115 | 3.56E-07 | 5.27E-06 | up |
| ENSMUS( | Zfp109 | 58 | 70 | 62 | 32 | 24 | 29 | 2.210499 | 4.33E-07 | 6.28E-06 | up |
| ENSMUS( | Gm3373 | 24 | 29 | 27 | 8 | 7 | 3 | 4.360858 | 4.91E-07 | 7.07E-06 | up |
| ENSMUS( | Dnhd1 | 72 | 73 | 58 | 27 | 32 | 35 | 2.133005 | 4.99E-07 | 7.17E-06 | up |
| ENSMUS( | Pabpn1 | 299 | 269 | 343 | 196 | 197 | 196 | 1.539485 | 5.2E-07 | 7.45E-06 | up |
| ENSMUS( | Gmnc | 169 | 179 | 160 | 109 | 107 | 103 | 1.572832 | 5.52E-07 | 7.86E-06 | up |
| ENSMUS( | Pon3 | 103 | 100 | 110 | 52 | 53 | 66 | 1.821977 | 5.67E-07 | 8.07E-06 | up |
| ENSMUS( | Cdh7 | 144 | 177 | 138 | 88 | 86 | 108 | 1.609971 | 6.84E-07 | 9.56E-06 | up |
| ENSMUS( | Gm1110 | 23 | 22 | 37 | 5 | 6 | 7 | 4.588732 | 7.47E-07 | 1.04E-05 | up |
| ENSMUS( | Klf7 | 130 | 145 | 113 | 73 | 65 | 90 | 1.682249 | 7.51E-07 | 1.04E-05 | up |
| ENSMUS( | Lama5 | 148 | 120 | 132 | 70 | 80 | 85 | 1.688899 | 7.99E-07 | 1.1E-05 | up |
| ENSMUS( | Fam227a | 102 | 112 | 113 | 57 | 60 | 66 | 1.774971 | 8.36E-07 | 1.15E-05 | up |
| ENSMUS( | Epn3 | 135 | 118 | 127 | 66 | 73 | 84 | 1.692656 | 9.15E-07 | 1.25E-05 | up |
| ENSMUS( | Sstr4 | 91 | 72 | 67 | 29 | 44 | 41 | 1.994754 | 9.94E-07 | 1.35E-05 | up |
| ENSMUS( | Gm9938 | 46 | 36 | 42 | 14 | 19 | 13 | 2.667664 | 1.09E-06 | 1.46E-05 | up |
| ENSMUS( | Zfp870 | 208 | 265 | 208 | 141 | 154 | 147 | 1.521111 | 1.35E-06 | 1.79E-05 | up |
| ENSMUS( | Gm2163 | 124 | 97 | 86 | 61 | 44 | 55 | 1.888506 | 1.37E-06 | 1.81E-05 | up |
| ENSMUS( | Wdfy2 | 44 | 52 | 32 | 17 | 16 | 16 | 2.560806 | 1.42E-06 | 1.87E-05 | up |
| ENSMUS( | Mrs2 | 218 | 218 | 200 | 99 | 147 | 136 | 1.649578 | 1.44E-06 | 1.89E-05 | up |
| ENSMUS( | Rtel1 | 176 | 218 | 173 | 104 | 123 | 139 | 1.533758 | 1.49E-06 | 1.95E-05 | up |
| ENSMUS( | Metap1d | 179 | 212 | 196 | 125 | 121 | 136 | 1.523465 | 1.49E-06 | 1.95E-05 | up |
| ENSMUS( | Onecut2 | 120 | 137 | 114 | 80 | 62 | 62 | 1.79114 | 1.55E-06 | 2.03E-05 | up |
| ENSMUS( | Lonrf3 | 117 | 89 | 83 | 55 | 47 | 39 | 2.012644 | 1.71E-06 | 2.22E-05 | up |
| ENSMUS( | Col27a1 | 100 | 96 | 86 | 54 | 51 | 49 | 1.806025 | 1.9E-06 | 2.44E-05 | up |
| ENSMUS( | Inmt | 55 | 62 | 59 | 25 | 31 | 25 | 2.150899 | 2.28E-06 | 2.9E-05 | up |
| ENSMUS( | Chdh | 118 | 111 | 94 | 58 | 67 | 60 | 1.721049 | 2.34E-06 | 2.97E-05 | up |
| ENSMUS( | Ccdc171 | 63 | 62 | 32 | 19 | 21 | 19 | 2.596231 | 2.37E-06 | 3.01E-05 | up |
| ENSMUS( | Cdh3 | 38 | 43 | 25 | 8 | 13 | 16 | 2.824824 | 2.38E-06 | 3.01E-05 | up |
| ENSMUS( | Irs1 | 103 | 101 | 85 | 33 | 47 | 59 | 2.062481 | 2.39E-06 | 3.02E-05 | up |
| ENSMUS( | Pla2g5 | 111 | 107 | 100 | 48 | 66 | 43 | 1.997683 | 2.55E-06 | 3.21E-05 | up |
| ENSMUS( | Tbc1d2 | 78 | 104 | 77 | 34 | 52 | 45 | 1.953321 | 2.66E-06 | 3.33E-05 | up |
| ENSMUS( | Tc2n | 45 | 42 | 48 | 20 | 21 | 14 | 2.427295 | 2.7E-06 | 3.37E-05 | up |
| ENSMUS( | Zfp966 | 66 | 62 | 47 | 28 | 22 | 31 | 2.128413 | 2.83E-06 | 3.53E-05 | up |
| ENSMUS( | Slc25a30 | 92 | 87 | 85 | 47 | 42 | 26 | 2.256645 | 2.83E-06 | 3.53E-05 | up |
| ENSMUS( | Mns1 | 165 | 155 | 123 | 100 | 78 | 98 | 1.580392 | 3.21E-06 | 3.97E-05 | up |
| ENSMUS( | Col20a1 | 80 | 74 | 47 | 31 | 27 | 33 | 2.165707 | 3.22E-06 | 3.97E-05 | up |
| ENSMUS( | Zfp809 | 167 | 188 | 161 | 102 | 121 | 114 | 1.513891 | 3.31E-06 | 4.09E-05 | up |
| ENSMUS( | Sult1c2 | 23 | 21 | 28 | 7 | 4 | 8 | 3.789187 | 3.43E-06 | 4.22E-05 | up |
| ENSMUS( | Gpr165 | 105 | 108 | 77 | 49 | 49 | 65 | 1.75478 | 3.82E-06 | 4.65E-05 | up |
| ENSMUS( | Piezo1 | 54 | 74 | 41 | 18 | 29 | 23 | 2.368822 | 4.15E-06 | 5.02E-05 | up |
| ENSMUS( | 5430403G | 110 | 130 | 100 | 58 | 52 | 78 | 1.790285 | 4.2E-06 | 5.08E-05 | up |
| ENSMUS( | Sik2 | 94 | 75 | 73 | 33 | 48 | 48 | 1.857913 | 4.44E-06 | 5.34E-05 | up |
| ENSMUS( | Muc3a | 126 | 91 | 109 | 41 | 63 | 49 | 2.112828 | 4.47E-06 | 5.36E-05 | up |
| ENSMUS( | Pde11a | 95 | 99 | 89 | 45 | 62 | 49 | 1.792886 | 4.55E-06 | 5.46E-05 | up |
| ENSMUS( | Mypn | 29 | 31 | 26 | 7 | 7 | 13 | 3.168996 | 4.73E-06 | 5.67E-05 | up |
| ENSMUS( | Map3k15 | 53 | 58 | 58 | 21 | 24 | 34 | 2.13302 | 5.22E-06 | 6.21E-05 | up |
| ENSMUS( | Cfap43 | 151 | 131 | 133 | 83 | 90 | 87 | 1.579339 | 5.35E-06 | 6.34E-05 | up |
| ENSMUS( | Caprin2 | 158 | 160 | 132 | 77 | 70 | 109 | 1.741372 | 5.7E-06 | 6.71E-05 | up |
| ENSMUS( | Gm16432 | 181 | 169 | 130 | 75 | 88 | 115 | 1.706408 | 5.89E-06 | 6.92E-05 | up |
| ENSMUS( | Dynlt1c | 163 | 178 | 162 | 113 | 100 | 118 | 1.504312 | 6.29E-06 | 7.35E-05 | up |
| ENSMUS( | Trpc5 | 119 | 78 | 96 | 35 | 52 | 35 | 2.375475 | 6.32E-06 | 7.37E-05 | up |
| ENSMUS( | Arntl2 | 120 | 130 | 112 | 67 | 70 | 83 | 1.629255 | 6.57E-06 | 7.62E-05 | up |
| ENSMUS( | Ppp1r3b | 90 | 82 | 71 | 36 | 45 | 51 | 1.821498 | 7.6E-06 | 8.7E-05 | up |
| ENSMUS( | Zdhhc23 | 89 | 59 | 54 | 30 | 20 | 34 | 2.371472 | 7.76E-06 | 8.86E-05 | up |
| ENSMUS( | Crhr2 | 78 | 87 | 87 | 35 | 53 | 40 | 1.952826 | 8.33E-06 | 9.48E-05 | up |
| ENSMUS( | Fam228b | 95 | 96 | 83 | 52 | 50 | 53 | 1.745342 | 8.4E-06 | 9.54E-05 | up |
| ENSMUS( | Pggt1b | 154 | 156 | 105 | 80 | 77 | 90 | 1.651505 | 8.64E-06 | 9.79E-05 | up |
| ENSMUS( | Pcdhga8 | 58 | 46 | 37 | 22 | 11 | 11 | 3.127099 | 8.67E-06 | 9.81E-05 | up |
| ENSMUS( | Gpr63 | 53 | 56 | 47 | 25 | 23 | 24 | 2.13738 | 8.81E-06 | 9.96E-05 | up |
| ENSMUS( | D130040H | 72 | 66 | 65 | 22 | 39 | 29 | 2.23373 | 9.06E-06 | 0.000102 | up |
| ENSMUS( | 4931406P1 | 141 | 107 | 95 | 63 | 56 | 74 | 1.753662 | 9.28E-06 | 0.000104 | up |
| ENSMUS( | Lcor | 48 | 54 | 31 | 21 | 15 | 19 | 2.366446 | 9.67E-06 | 0.000108 | up |
| ENSMUS( | Pcdhga11 | 41 | 33 | 25 | 14 | 10 | 12 | 2.696423 | 9.89E-06 | 0.00011 | up |
| ENSMUS( | Lekr1 | 150 | 162 | 130 | 92 | 92 | 102 | 1.525656 | 1.11E-05 | 0.000123 | up |
| ENSMUS( | Capn6 | 94 | 105 | 88 | 64 | 51 | 51 | 1.703176 | 1.12E-05 | 0.000123 | up |
| ENSMUS( | L3mbtl1 | 55 | 45 | 38 | 16 | 18 | 27 | 2.240558 | 1.18E-05 | 0.000129 | up |
| ENSMUS( | Cd55 | 223 | 281 | 206 | 158 | 156 | 132 | 1.565278 | 1.19E-05 | 0.00013 | up |
| ENSMUS( | Slc16a12 | 164 | 130 | 104 | 67 | 82 | 82 | 1.696111 | 1.3E-05 | 0.000141 | up |
| ENSMUS( | Lig4 | 209 | 211 | 217 | 125 | 161 | 126 | 1.531596 | 1.44E-05 | 0.000156 | up |

| | | | | | | | | | | | |
|---|---|---|---|---|---|---|---|---|---|---|---|
| ENSMUS( | Gm853 | 17 | 11 | 17 | 4 | 1 | 3 | 5.593039 | 1.45E-05 | 0.000156 | up |
| ENSMUS( | Tat | 8 | 10 | 8 | 1 | 0 | 0 | 24.96287 | 1.53E-05 | 0.000164 | up |
| ENSMUS( | Sfrp5 | 42 | 45 | 39 | 16 | 21 | 11 | 2.580657 | 1.57E-05 | 0.000167 | up |
| ENSMUS( | Msantd2 | 332 | 282 | 281 | 229 | 171 | 181 | 1.519271 | 1.56E-05 | 0.000167 | up |
| ENSMUS( | Zfp551 | 61 | 52 | 29 | 15 | 24 | 17 | 2.47523 | 1.65E-05 | 0.000176 | up |
| ENSMUS( | Nek4 | 236 | 205 | 159 | 122 | 144 | 126 | 1.504347 | 1.81E-05 | 0.00019 | up |
| ENSMUS( | Col6a5 | 21 | 27 | 26 | 6 | 6 | 11 | 3.217683 | 1.92E-05 | 0.000201 | up |
| ENSMUS( | Cfap44 | 133 | 143 | 106 | 78 | 80 | 84 | 1.554669 | 1.94E-05 | 0.000203 | up |
| ENSMUS( | BC067074 | 50 | 64 | 47 | 24 | 27 | 27 | 2.036378 | 1.95E-05 | 0.000204 | up |
| ENSMUS( | Fan1 | 89 | 90 | 54 | 32 | 44 | 42 | 1.937528 | 1.99E-05 | 0.000207 | up |
| ENSMUS( | Pcdh15 | 211 | 244 | 229 | 123 | 144 | 177 | 1.531984 | 2.01E-05 | 0.000209 | up |
| ENSMUS( | Gm38394 | 20 | 13 | 8 | 2 | 1 | 3 | 6.715596 | 2.03E-05 | 0.000211 | up |
| ENSMUS( | Gm3558 | 33 | 32 | 26 | 14 | 9 | 10 | 2.707895 | 2.15E-05 | 0.000223 | up |
| ENSMUS( | Nid2 | 113 | 144 | 99 | 49 | 76 | 61 | 1.885457 | 2.19E-05 | 0.000227 | up |
| ENSMUS( | Slc22a21 | 38 | 45 | 35 | 20 | 14 | 16 | 2.322707 | 2.31E-05 | 0.000238 | up |
| ENSMUS( | Pon1 | 37 | 32 | 30 | 10 | 11 | 17 | 2.588679 | 2.32E-05 | 0.00024 | up |
| ENSMUS( | Hfm1 | 50 | 53 | 54 | 26 | 19 | 31 | 2.054774 | 2.41E-05 | 0.000248 | up |
| ENSMUS( | Mki67 | 71 | 77 | 57 | 35 | 31 | 44 | 1.840077 | 2.65E-05 | 0.000271 | up |
| ENSMUS( | Tex9 | 50 | 47 | 47 | 23 | 27 | 18 | 2.089245 | 3.07E-05 | 0.00031 | up |
| ENSMUS( | Itga1 | 66 | 54 | 47 | 30 | 29 | 19 | 2.097984 | 3.14E-05 | 0.000316 | up |
| ENSMUS( | Pop1 | 118 | 105 | 95 | 54 | 69 | 72 | 1.613446 | 3.15E-05 | 0.000317 | up |
| ENSMUS( | Nek5 | 69 | 50 | 49 | 16 | 22 | 33 | 2.352226 | 3.19E-05 | 0.00032 | up |
| ENSMUS( | Slc16a8 | 68 | 83 | 51 | 6 | 29 | 31 | 3.036112 | 3.5E-05 | 0.000349 | up |
| ENSMUS( | Sntb1 | 70 | 59 | 46 | 27 | 28 | 35 | 1.916874 | 3.69E-05 | 0.000365 | up |
| ENSMUS( | Zfp994 | 294 | 325 | 230 | 204 | 159 | 184 | 1.525171 | 3.8E-05 | 0.000375 | up |
| ENSMUS( | Gm16485 | 186 | 191 | 177 | 116 | 84 | 142 | 1.607466 | 4.08E-05 | 0.000399 | up |
| ENSMUS( | Fap | 81 | 76 | 68 | 30 | 15 | 49 | 2.383127 | 4.11E-05 | 0.000402 | up |
| ENSMUS( | Sned1 | 128 | 110 | 93 | 74 | 53 | 71 | 1.646681 | 4.18E-05 | 0.000408 | up |
| ENSMUS( | Large2 | 20 | 28 | 25 | 4 | 4 | 10 | 4.068801 | 4.55E-05 | 0.000442 | up |
| ENSMUS( | Syt10 | 51 | 52 | 60 | 18 | 32 | 25 | 2.165001 | 4.66E-05 | 0.000451 | up |
| ENSMUS( | Dgkq | 149 | 168 | 133 | 80 | 109 | 104 | 1.517712 | 4.67E-05 | 0.000452 | up |
| ENSMUS( | Serpinb1b | 99 | 99 | 67 | 56 | 53 | 44 | 1.695224 | 4.73E-05 | 0.000458 | up |
| ENSMUS( | Cdca7l | 29 | 39 | 31 | 16 | 12 | 12 | 2.437965 | 5.08E-05 | 0.000488 | up |
| ENSMUS( | Zkscan7 | 95 | 82 | 93 | 45 | 57 | 59 | 1.667561 | 5.08E-05 | 0.000488 | up |
| ENSMUS( | mt-Nd4l | 45 | 0 | 4 | 4 | 2 | 1 | 6.667471 | 5.33E-05 | 0.000509 | up |
| ENSMUS( | Adam32 | 28 | 13 | 15 | 6 | 3 | 4 | 4.222735 | 5.41E-05 | 0.000516 | up |
| ENSMUS( | Ccdc191 | 121 | 144 | 131 | 84 | 84 | 92 | 1.509452 | 5.55E-05 | 0.000529 | up |
| ENSMUS( | Cndp1 | 17 | 33 | 30 | 5 | 11 | 6 | 3.61413 | 5.68E-05 | 0.000541 | up |
| ENSMUS( | Rbm41 | 140 | 121 | 90 | 45 | 52 | 77 | 1.99357 | 5.86E-05 | 0.000556 | up |
| ENSMUS( | Alpk1 | 85 | 90 | 62 | 53 | 39 | 41 | 1.746181 | 5.89E-05 | 0.000558 | up |
| ENSMUS( | Ccdc187 | 82 | 80 | 58 | 24 | 47 | 31 | 2.120825 | 5.96E-05 | 0.000564 | up |
| ENSMUS( | Tubd1 | 165 | 146 | 151 | 86 | 98 | 121 | 1.50514 | 6.17E-05 | 0.00058 | up |
| ENSMUS( | Slc24a5 | 97 | 77 | 60 | 44 | 39 | 44 | 1.810058 | 6.63E-05 | 0.000617 | up |
| ENSMUS( | Zfp599 | 104 | 123 | 97 | 74 | 63 | 68 | 1.558043 | 7.77E-05 | 0.000712 | up |
| ENSMUS( | Iqub | 130 | 114 | 111 | 72 | 75 | 83 | 1.527831 | 8.4E-05 | 0.000763 | up |
| ENSMUS( | Usp18 | 35 | 28 | 37 | 15 | 12 | 15 | 2.369815 | 8.48E-05 | 0.00077 | up |
| ENSMUS( | Eif2ak2 | 163 | 163 | 142 | 109 | 107 | 83 | 1.54017 | 8.6E-05 | 0.00078 | up |
| ENSMUS( | Itpripl2 | 117 | 121 | 104 | 56 | 82 | 70 | 1.625355 | 9.13E-05 | 0.000822 | up |
| ENSMUS( | Wdr72 | 25 | 37 | 21 | 9 | 14 | 8 | 2.619905 | 9.37E-05 | 0.000842 | up |
| ENSMUS( | Igfn1 | 18 | 37 | 37 | 9 | 11 | 12 | 2.87607 | 9.53E-05 | 0.000854 | up |
| ENSMUS( | Kcnk9 | 28 | 31 | 23 | 12 | 12 | 7 | 2.58862 | 9.55E-05 | 0.000855 | up |
| ENSMUS( | Col4a4 | 70 | 66 | 84 | 28 | 45 | 35 | 2.030893 | 9.61E-05 | 0.000861 | up |
| ENSMUS( | Fam46c | 123 | 120 | 109 | 76 | 80 | 73 | 1.517406 | 0.000104 | 0.000919 | up |
| ENSMUS( | Catsperg2 | 11 | 9 | 8 | 1 | 1 | 1 | 9.196192 | 0.00011 | 0.000968 | up |
| ENSMUS( | Iigp1 | 108 | 153 | 104 | 75 | 74 | 57 | 1.739081 | 0.000126 | 0.001096 | up |
| ENSMUS( | Cldn3 | 14 | 24 | 23 | 9 | 5 | 4 | 3.343414 | 0.000145 | 0.001246 | up |
| ENSMUS( | Lmx1a | 31 | 39 | 23 | 13 | 15 | 11 | 2.332783 | 0.000151 | 0.001285 | up |
| ENSMUS( | Malt1 | 146 | 105 | 110 | 77 | 52 | 71 | 1.782423 | 0.000159 | 0.001348 | up |
| ENSMUS( | Eps8l2 | 29 | 30 | 21 | 9 | 9 | 13 | 2.547291 | 0.000161 | 0.001362 | up |
| ENSMUS( | Olfr648 | 21 | 21 | 14 | 3 | 7 | 7 | 3.253452 | 0.000169 | 0.001418 | up |
| ENSMUS( | Parp14 | 60 | 79 | 68 | 40 | 38 | 42 | 1.708288 | 0.000178 | 0.00149 | up |
| ENSMUS( | Sfxn2 | 60 | 54 | 49 | 32 | 22 | 34 | 1.831147 | 0.000192 | 0.001595 | up |
| ENSMUS( | 1700109H | 32 | 24 | 20 | 4 | 8 | 12 | 3.146426 | 0.000192 | 0.001599 | up |
| ENSMUS( | Wars2 | 147 | 86 | 111 | 60 | 77 | 67 | 1.670011 | 0.000195 | 0.001619 | up |
| ENSMUS( | Gcfc2 | 86 | 67 | 72 | 41 | 45 | 48 | 1.663715 | 0.000195 | 0.00162 | up |
| ENSMUS( | Plcxd3 | 45 | 56 | 26 | 17 | 6 | 11 | 3.630812 | 0.000199 | 0.001646 | up |
| ENSMUS( | Tet1 | 217 | 244 | 167 | 85 | 133 | 147 | 1.699522 | 0.000205 | 0.001691 | up |
| ENSMUS( | Itpripl1 | 85 | 76 | 91 | 35 | 30 | 60 | 2.018538 | 0.000212 | 0.001737 | up |
| ENSMUS( | Il18bp | 88 | 84 | 64 | 49 | 44 | 50 | 1.624 | 0.000221 | 0.001796 | up |
| ENSMUS( | Adamts3 | 111 | 75 | 64 | 43 | 47 | 48 | 1.781298 | 0.000225 | 0.001827 | up |
| ENSMUS( | Nek10 | 19 | 21 | 15 | 3 | 7 | 7 | 3.203268 | 0.000228 | 0.001844 | up |
| ENSMUS( | Palm2 | 24 | 41 | 37 | 13 | 18 | 12 | 2.351688 | 0.000231 | 0.001867 | up |
| ENSMUS( | 9930111J2 | 19 | 13 | 21 | 4 | 8 | 4 | 3.298596 | 0.000237 | 0.001909 | up |
| ENSMUS( | Acad10 | 71 | 65 | 63 | 31 | 41 | 44 | 1.701773 | 0.000267 | 0.002125 | up |
| ENSMUS( | Flt3 | 125 | 124 | 81 | 59 | 64 | 82 | 1.584651 | 0.000274 | 0.002177 | up |
| ENSMUS( | Dnah7b | 101 | 80 | 79 | 61 | 49 | 53 | 1.57307 | 0.00029 | 0.002284 | up |
| ENSMUS( | Slc26a10 | 109 | 107 | 86 | 58 | 69 | 69 | 1.520108 | 0.000293 | 0.002302 | up |
| ENSMUS( | Zfp960 | 62 | 71 | 71 | 45 | 29 | 40 | 1.774198 | 0.000294 | 0.002311 | up |
| ENSMUS( | Fam228a | 26 | 29 | 34 | 7 | 5 | 15 | 3.320149 | 0.000297 | 0.002331 | up |
| ENSMUS( | Stard9 | 132 | 105 | 92 | 56 | 75 | 79 | 1.547671 | 0.000318 | 0.002469 | up |

| | | | | | | | | | | | |
|---|---|---|---|---|---|---|---|---|---|---|---|
| ENSMUS( | Gbp9 | 87 | 106 | 65 | 51 | 38 | 58 | 1.726087 | 0.000331 | 0.00256 | up |
| ENSMUS( | Plek2 | 24 | 31 | 35 | 15 | 14 | 10 | 2.289142 | 0.000341 | 0.00263 | up |
| ENSMUS( | Dnase1l2 | 40 | 32 | 28 | 16 | 16 | 14 | 2.137253 | 0.000343 | 0.002644 | up |
| ENSMUS( | Dnah6 | 169 | 164 | 128 | 120 | 92 | 87 | 1.512528 | 0.000348 | 0.002676 | up |
| ENSMUS( | Pcdhac2 | 192 | 158 | 132 | 69 | 105 | 110 | 1.67708 | 0.000348 | 0.002679 | up |
| ENSMUS( | Dpep1 | 16 | 22 | 19 | 4 | 9 | 6 | 2.97528 | 0.000355 | 0.002725 | up |
| ENSMUS( | Podn | 101 | 86 | 79 | 54 | 59 | 56 | 1.553047 | 0.000363 | 0.002778 | up |
| ENSMUS( | Zfp458 | 76 | 70 | 72 | 48 | 40 | 44 | 1.634261 | 0.000368 | 0.002813 | up |
| ENSMUS( | Dclre1c | 176 | 177 | 145 | 76 | 105 | 140 | 1.539238 | 0.000383 | 0.002917 | up |
| ENSMUS( | Traf3ip2 | 68 | 87 | 77 | 45 | 54 | 44 | 1.604907 | 0.000387 | 0.002938 | up |
| ENSMUS( | Tjp3 | 52 | 50 | 54 | 25 | 30 | 31 | 1.802974 | 0.000398 | 0.003017 | up |
| ENSMUS( | Csf2rb2 | 41 | 48 | 39 | 18 | 26 | 22 | 1.916581 | 0.000413 | 0.003121 | up |
| ENSMUS( | Soga1 | 98 | 104 | 60 | 45 | 60 | 48 | 1.675722 | 0.000414 | 0.003127 | up |
| ENSMUS( | Pyroxd1 | 86 | 78 | 64 | 53 | 32 | 40 | 1.791617 | 0.000431 | 0.003234 | up |
| ENSMUS( | Gm42878 | 23 | 20 | 20 | 11 | 6 | 6 | 2.691848 | 0.000445 | 0.00333 | up |
| ENSMUS( | Cntnap5c | 60 | 56 | 42 | 28 | 26 | 34 | 1.769527 | 0.000453 | 0.00338 | up |
| ENSMUS( | Trmt13 | 74 | 102 | 72 | 43 | 53 | 55 | 1.621952 | 0.000459 | 0.003426 | up |
| ENSMUS( | Crb3 | 44 | 43 | 45 | 10 | 25 | 21 | 2.3483 | 0.000461 | 0.003436 | up |
| ENSMUS( | Tnfsf10 | 52 | 63 | 51 | 25 | 29 | 40 | 1.753071 | 0.000463 | 0.003447 | up |
| ENSMUS( | Fam46a | 193 | 185 | 126 | 116 | 77 | 87 | 1.761373 | 0.000468 | 0.003478 | up |
| ENSMUS( | Ripk4 | 42 | 50 | 25 | 23 | 15 | 16 | 2.108947 | 0.000473 | 0.003513 | up |
| ENSMUS( | Gm2026 | 59 | 53 | 47 | 33 | 32 | 24 | 1.755744 | 0.00048 | 0.003554 | up |
| ENSMUS( | Zgrf1 | 104 | 113 | 66 | 53 | 64 | 53 | 1.629047 | 0.00049 | 0.003625 | up |
| ENSMUS( | Rtl1 | 55 | 58 | 65 | 23 | 38 | 37 | 1.812474 | 0.000517 | 0.003802 | up |
| ENSMUS( | Itk | 34 | 21 | 21 | 8 | 8 | 15 | 2.43381 | 0.000554 | 0.004042 | up |
| ENSMUS( | Nynrin | 33 | 19 | 14 | 6 | 10 | 6 | 2.928216 | 0.000558 | 0.004065 | up |
| ENSMUS( | Gm14137 | 21 | 14 | 12 | 6 | 6 | 2 | 3.263254 | 0.000566 | 0.00411 | up |
| ENSMUS( | Ngfr | 33 | 45 | 27 | 19 | 16 | 16 | 2.017395 | 0.000567 | 0.004119 | up |
| ENSMUS( | Tmem27 | 59 | 53 | 29 | 5 | 20 | 15 | 3.46226 | 0.000573 | 0.004155 | up |
| ENSMUS( | Plekhh2 | 143 | 137 | 113 | 68 | 102 | 86 | 1.514883 | 0.00058 | 0.004199 | up |
| ENSMUS( | Marveld2 | 64 | 51 | 48 | 34 | 26 | 33 | 1.729232 | 0.000606 | 0.004364 | up |
| ENSMUS( | Steap4 | 36 | 27 | 16 | 2 | 9 | 3 | 5.500117 | 0.000611 | 0.004393 | up |
| ENSMUS( | Marveld3 | 9 | 21 | 11 | 2 | 3 | 5 | 4.068119 | 0.000655 | 0.004663 | up |
| ENSMUS( | Ccdc170 | 35 | 25 | 25 | 11 | 12 | 15 | 2.21443 | 0.000673 | 0.004767 | up |
| ENSMUS( | Cox8b | 58 | 69 | 50 | 40 | 27 | 37 | 1.675509 | 0.000677 | 0.004792 | up |
| ENSMUS( | Gm3591 | 14 | 22 | 14 | 6 | 1 | 3 | 4.886047 | 0.000743 | 0.005197 | up |
| ENSMUS( | Nusap1 | 65 | 70 | 57 | 43 | 39 | 34 | 1.628258 | 0.000765 | 0.005335 | up |
| ENSMUS( | Mfsd7c | 71 | 64 | 40 | 22 | 32 | 38 | 1.872062 | 0.000789 | 0.005477 | up |
| ENSMUS( | Mpp7 | 118 | 116 | 143 | 84 | 54 | 63 | 1.862501 | 0.000795 | 0.005507 | up |
| ENSMUS( | Olfr539 | 67 | 65 | 57 | 39 | 36 | 39 | 1.636508 | 0.000809 | 0.005596 | up |
| ENSMUS( | Tmem184a | 15 | 21 | 13 | 5 | 7 | 4 | 2.998725 | 0.000813 | 0.005623 | up |
| ENSMUS( | Dok6 | 47 | 76 | 32 | 30 | 21 | 20 | 2.121122 | 0.000818 | 0.005648 | up |
| ENSMUS( | Aff2 | 49 | 44 | 41 | 27 | 22 | 24 | 1.810905 | 0.000821 | 0.005664 | up |
| ENSMUS( | Slco1a5 | 17 | 27 | 27 | 9 | 9 | 11 | 2.442712 | 0.000837 | 0.005754 | up |
| ENSMUS( | Chrm5 | 24 | 18 | 12 | 6 | 9 | 4 | 2.766337 | 0.000847 | 0.005809 | up |
| ENSMUS( | D930048N | 50 | 39 | 37 | 27 | 19 | 16 | 1.993511 | 0.000867 | 0.00593 | up |
| ENSMUS( | Sytl3 | 17 | 16 | 14 | 7 | 4 | 4 | 3.073653 | 0.000874 | 0.00597 | up |
| ENSMUS( | Cdk1 | 18 | 27 | 25 | 12 | 9 | 8 | 2.386109 | 0.000879 | 0.005997 | up |
| ENSMUS( | Klhl31 | 13 | 7 | 12 | 2 | 3 | 2 | 4.546421 | 0.000895 | 0.006093 | up |
| ENSMUS( | Zfp619 | 93 | 97 | 75 | 61 | 50 | 63 | 1.501126 | 0.00091 | 0.006177 | up |
| ENSMUS( | Map3k19 | 126 | 105 | 76 | 65 | 62 | 67 | 1.553219 | 0.000935 | 0.006325 | up |
| ENSMUS( | Vgll3 | 69 | 79 | 59 | 42 | 42 | 45 | 1.581672 | 0.001001 | 0.006711 | up |
| ENSMUS( | 4933428G | 12 | 18 | 13 | 6 | 4 | 3 | 3.239818 | 0.001009 | 0.006759 | up |
| ENSMUS( | Prkg1 | 118 | 90 | 77 | 45 | 64 | 66 | 1.607557 | 0.001031 | 0.006876 | up |
| ENSMUS( | Masp2 | 53 | 47 | 57 | 27 | 29 | 35 | 1.719892 | 0.001032 | 0.006883 | up |
| ENSMUS( | Styk1 | 25 | 28 | 16 | 4 | 9 | 12 | 2.729165 | 0.001054 | 0.007002 | up |
| ENSMUS( | Arhgef5 | 81 | 67 | 57 | 34 | 50 | 44 | 1.579531 | 0.001104 | 0.007289 | up |
| ENSMUS( | Nkain3 | 73 | 65 | 54 | 32 | 42 | 44 | 1.607089 | 0.001115 | 0.007358 | up |
| ENSMUS( | Wisp1 | 25 | 24 | 20 | 13 | 9 | 7 | 2.330066 | 0.001128 | 0.007426 | up |
| ENSMUS( | Mtcp1 | 64 | 69 | 51 | 33 | 40 | 39 | 1.618964 | 0.001147 | 0.007528 | up |
| ENSMUS( | Iqcd | 22 | 26 | 27 | 9 | 13 | 11 | 2.260106 | 0.001164 | 0.007627 | up |
| ENSMUS( | Lancl3 | 21 | 23 | 17 | 7 | 11 | 6 | 2.494311 | 0.001166 | 0.007632 | up |
| ENSMUS( | Zfp54 | 49 | 34 | 38 | 16 | 24 | 25 | 1.847884 | 0.001167 | 0.007637 | up |
| ENSMUS( | Tsnaxip1 | 26 | 25 | 27 | 11 | 15 | 9 | 2.203867 | 0.001171 | 0.007654 | up |
| ENSMUS( | Chmp4c | 25 | 39 | 20 | 7 | 11 | 16 | 2.440419 | 0.00123 | 0.007981 | up |
| ENSMUS( | Gramd1c | 93 | 87 | 75 | 57 | 63 | 47 | 1.501999 | 0.00124 | 0.00804 | up |
| ENSMUS( | Zcwpw1 | 82 | 84 | 79 | 41 | 62 | 49 | 1.595651 | 0.001255 | 0.008119 | up |
| ENSMUS( | Adamts5 | 48 | 45 | 58 | 25 | 33 | 27 | 1.769823 | 0.001258 | 0.008138 | up |
| ENSMUS( | Rab20 | 31 | 28 | 29 | 15 | 13 | 14 | 2.073493 | 0.00128 | 0.008257 | up |
| ENSMUS( | Klf12 | 35 | 19 | 17 | 6 | 9 | 2 | 4.054949 | 0.001284 | 0.008279 | up |
| ENSMUS( | Kif23 | 25 | 16 | 18 | 10 | 7 | 6 | 2.52046 | 0.001288 | 0.008299 | up |
| ENSMUS( | Zfp568 | 88 | 84 | 55 | 52 | 41 | 47 | 1.588381 | 0.0013 | 0.008362 | up |
| ENSMUS( | Prss41 | 49 | 61 | 51 | 27 | 24 | 40 | 1.758181 | 0.001394 | 0.008903 | up |
| ENSMUS( | Zfp382 | 52 | 45 | 54 | 26 | 28 | 34 | 1.709728 | 0.00142 | 0.009055 | up |
| ENSMUS( | Pigl | 93 | 85 | 107 | 64 | 60 | 60 | 1.540561 | 0.001447 | 0.009213 | up |
| ENSMUS( | Krtcap3 | 21 | 15 | 26 | 9 | 9 | 6 | 2.572553 | 0.001454 | 0.009255 | up |
| ENSMUS( | Crip3 | 29 | 24 | 18 | 10 | 10 | 11 | 2.251046 | 0.001469 | 0.009334 | up |
| ENSMUS( | Mak | 49 | 64 | 51 | 25 | 36 | 37 | 1.658629 | 0.001471 | 0.009343 | up |
| ENSMUS( | Ubap1l | 16 | 22 | 14 | 8 | 7 | 4 | 2.670594 | 0.001472 | 0.009346 | up |
| ENSMUS( | Dgkh | 46 | 61 | 49 | 27 | 36 | 21 | 1.828416 | 0.001483 | 0.009397 | up |

| | | | | | | | | | | | |
|---|---|---|---|---|---|---|---|---|---|---|---|
| ENSMUS( | Rttn | 91 | 89 | 75 | 44 | 56 | 67 | 1.512341 | 0.001508 | 0.009525 | up |
| ENSMUS( | Slco5a1 | 61 | 58 | 43 | 38 | 24 | 30 | 1.727357 | 0.00151 | 0.00953 | up |
| ENSMUS( | Kif27 | 66 | 53 | 39 | 28 | 25 | 37 | 1.728939 | 0.001561 | 0.00981 | up |
| ENSMUS( | Cep152 | 83 | 76 | 85 | 50 | 50 | 61 | 1.507441 | 0.001609 | 0.010069 | up |
| ENSMUS( | Daw1 | 36 | 37 | 31 | 17 | 18 | 19 | 1.902074 | 0.001616 | 0.010111 | up |
| ENSMUS( | Egr2 | 77 | 43 | 50 | 36 | 30 | 24 | 1.855683 | 0.001666 | 0.010381 | up |
| ENSMUS( | Mcmdc2 | 6 | 12 | 6 | 1 | 1 | 2 | 5.935695 | 0.001718 | 0.01068 | up |
| ENSMUS( | Gm20939 | 39 | 39 | 35 | 16 | 21 | 24 | 1.837356 | 0.001831 | 0.011288 | up |
| ENSMUS( | Hist1h2bg | 14 | 7 | 7 | 3 | 1 | 2 | 4.566722 | 0.001886 | 0.011568 | up |
| ENSMUS( | Gzmm | 25 | 37 | 27 | 10 | 17 | 17 | 2.004699 | 0.00191 | 0.01171 | up |
| ENSMUS( | Prcd | 59 | 44 | 46 | 31 | 30 | 27 | 1.670215 | 0.001947 | 0.011898 | up |
| ENSMUS( | Frem2 | 66 | 64 | 63 | 43 | 40 | 39 | 1.564072 | 0.001987 | 0.01211 | up |
| ENSMUS( | Gm21750 | 5 | 5 | 6 | 0 | 0 | 1 | 16.52317 | 0.001994 | 0.01215 | up |
| ENSMUS( | Pot1b | 61 | 52 | 70 | 37 | 29 | 43 | 1.676679 | 0.001996 | 0.012157 | up |
| ENSMUS( | Cabp4 | 6 | 5 | 5 | 0 | 1 | 0 | 15.68519 | 0.002002 | 0.012193 | up |
| ENSMUS( | Kctd16 | 66 | 80 | 79 | 34 | 39 | 60 | 1.690061 | 0.002074 | 0.012586 | up |
| ENSMUS( | Ptchd1 | 29 | 30 | 24 | 16 | 12 | 7 | 2.317306 | 0.002106 | 0.012753 | up |
| ENSMUS( | Gbp8 | 21 | 18 | 24 | 8 | 10 | 9 | 2.325508 | 0.002273 | 0.01364 | up |
| ENSMUS( | Hs6st3 | 30 | 34 | 24 | 17 | 16 | 11 | 1.95759 | 0.002339 | 0.014006 | up |
| ENSMUS( | Htr2a | 57 | 64 | 45 | 27 | 39 | 36 | 1.603901 | 0.002372 | 0.014188 | up |
| ENSMUS( | Helz2 | 50 | 44 | 31 | 22 | 25 | 24 | 1.728303 | 0.002429 | 0.014502 | up |
| ENSMUS( | Zfp977 | 23 | 18 | 17 | 9 | 5 | 10 | 2.390132 | 0.002503 | 0.014889 | up |
| ENSMUS( | Rad9b | 51 | 74 | 60 | 28 | 29 | 47 | 1.770934 | 0.002602 | 0.015383 | up |
| ENSMUS( | Sass6 | 66 | 72 | 46 | 38 | 38 | 40 | 1.557001 | 0.002642 | 0.015584 | up |
| ENSMUS( | Aldob | 48 | 36 | 51 | 25 | 26 | 25 | 1.768449 | 0.002648 | 0.015616 | up |
| ENSMUS( | Gm11639 | 15 | 5 | 5 | 1 | 1 | 2 | 6.160833 | 0.002659 | 0.015661 | up |
| ENSMUS( | Zfp979 | 73 | 77 | 71 | 41 | 47 | 58 | 1.502925 | 0.002691 | 0.015826 | up |
| ENSMUS( | Mycbpap | 49 | 67 | 51 | 41 | 15 | 28 | 1.956008 | 0.002769 | 0.016226 | up |
| ENSMUS( | Spag17 | 17 | 11 | 10 | 3 | 3 | 6 | 3.141621 | 0.002795 | 0.016342 | up |
| ENSMUS( | Syne4 | 59 | 31 | 56 | 28 | 21 | 30 | 1.844948 | 0.002829 | 0.016526 | up |
| ENSMUS( | Ybx2 | 44 | 39 | 37 | 22 | 25 | 21 | 1.741523 | 0.002852 | 0.016641 | up |
| ENSMUS( | Glp2r | 13 | 17 | 13 | 4 | 5 | 6 | 2.840628 | 0.002869 | 0.016732 | up |
| ENSMUS( | Hyal1 | 42 | 39 | 48 | 25 | 28 | 22 | 1.708064 | 0.003022 | 0.017501 | up |
| ENSMUS( | Abhd14b | 103 | 90 | 88 | 53 | 47 | 79 | 1.560147 | 0.003075 | 0.017761 | up |
| ENSMUS( | Wdr63 | 62 | 69 | 39 | 27 | 21 | 40 | 1.901834 | 0.003091 | 0.017834 | up |
| ENSMUS( | Wfdc6a | 5 | 3 | 7 | 0 | 1 | 0 | 14.96526 | 0.003163 | 0.018193 | up |
| ENSMUS( | Adamts6 | 32 | 52 | 36 | 18 | 27 | 16 | 1.935325 | 0.003184 | 0.018292 | up |
| ENSMUS( | Gm9772 | 6 | 4 | 5 | 0 | 0 | 1 | 15.42446 | 0.003212 | 0.018434 | up |
| ENSMUS( | Muc15 | 13 | 17 | 12 | 4 | 6 | 1 | 3.71762 | 0.003215 | 0.018446 | up |
| ENSMUS( | Dok3 | 65 | 81 | 56 | 46 | 41 | 45 | 1.50567 | 0.00322 | 0.018474 | up |
| ENSMUS( | Lif | 16 | 25 | 19 | 10 | 8 | 8 | 2.275193 | 0.003255 | 0.018642 | up |
| ENSMUS( | Oprd1 | 46 | 31 | 40 | 24 | 21 | 21 | 1.755898 | 0.00328 | 0.018769 | up |
| ENSMUS( | Smyd1 | 33 | 41 | 26 | 23 | 14 | 17 | 1.813519 | 0.003423 | 0.019481 | up |
| ENSMUS( | Nlrp1a | 15 | 8 | 13 | 1 | 5 | 2 | 4.47589 | 0.003424 | 0.019482 | up |
| ENSMUS( | Dnase1 | 54 | 60 | 30 | 17 | 31 | 21 | 2.038389 | 0.003528 | 0.019987 | up |
| ENSMUS( | Gen1 | 35 | 31 | 31 | 21 | 16 | 15 | 1.838581 | 0.003568 | 0.020177 | up |
| ENSMUS( | Gm45902 | 68 | 75 | 53 | 41 | 44 | 43 | 1.506169 | 0.003644 | 0.020565 | up |
| ENSMUS( | Gm4951 | 24 | 16 | 24 | 11 | 9 | 9 | 2.192645 | 0.003667 | 0.020663 | up |
| ENSMUS( | Mylk3 | 16 | 19 | 17 | 10 | 5 | 5 | 2.554642 | 0.003719 | 0.020915 | up |
| ENSMUS( | Sycp2 | 42 | 37 | 34 | 23 | 24 | 17 | 1.736379 | 0.003755 | 0.021086 | up |
| ENSMUS( | Itgb1bp2 | 6 | 10 | 12 | 1 | 4 | 2 | 4.009421 | 0.003773 | 0.021164 | up |
| ENSMUS( | Myh7 | 11 | 14 | 12 | 2 | 6 | 4 | 3.060159 | 0.003774 | 0.021167 | up |
| ENSMUS( | Pabpc5 | 21 | 27 | 4 | 5 | 7 | 5 | 2.937684 | 0.003856 | 0.021552 | up |
| ENSMUS( | Kif22 | 57 | 64 | 75 | 34 | 42 | 47 | 1.592131 | 0.003864 | 0.02159 | up |
| ENSMUS( | Aire | 25 | 22 | 27 | 15 | 12 | 9 | 2.032413 | 0.003901 | 0.021746 | up |
| ENSMUS( | Pask | 57 | 30 | 39 | 25 | 23 | 20 | 1.827049 | 0.004201 | 0.023128 | up |
| ENSMUS( | Gm43951 | 18 | 15 | 17 | 1 | 7 | 1 | 5.477879 | 0.004231 | 0.023255 | up |
| ENSMUS( | Col6a3 | 70 | 80 | 59 | 43 | 37 | 57 | 1.508113 | 0.004338 | 0.023762 | up |
| ENSMUS( | Cgn | 32 | 45 | 54 | 21 | 22 | 29 | 1.824681 | 0.004358 | 0.023854 | up |
| ENSMUS( | Gm43517 | 41 | 32 | 27 | 16 | 18 | 21 | 1.793784 | 0.004391 | 0.024017 | up |
| ENSMUS( | Olfr1307 | 10 | 27 | 15 | 7 | 8 | 4 | 2.67743 | 0.004423 | 0.024163 | up |
| ENSMUS( | Mecom | 57 | 52 | 51 | 33 | 33 | 35 | 1.567671 | 0.004636 | 0.025205 | up |
| ENSMUS( | Dnah3 | 30 | 20 | 36 | 14 | 13 | 15 | 2.050489 | 0.005021 | 0.026956 | up |
| ENSMUS( | Cyp27b1 | 6 | 11 | 12 | 3 | 1 | 4 | 3.64491 | 0.005087 | 0.02723 | up |
| ENSMUS( | Ccdc68 | 11 | 16 | 9 | 5 | 3 | 4 | 2.936947 | 0.005141 | 0.02746 | up |
| ENSMUS( | Cd300lg | 5 | 7 | 7 | 1 | 2 | 0 | 6.203274 | 0.005173 | 0.027604 | up |
| ENSMUS( | Rab11fip1 | 44 | 57 | 62 | 37 | 29 | 34 | 1.621826 | 0.005595 | 0.029564 | up |
| ENSMUS( | Ankdd1a | 13 | 11 | 10 | 4 | 2 | 5 | 3.062939 | 0.005621 | 0.029679 | up |
| ENSMUS( | Glb1l2 | 48 | 50 | 58 | 34 | 26 | 39 | 1.571297 | 0.00567 | 0.029913 | up |
| ENSMUS( | Olfr1507 | 15 | 5 | 5 | 1 | 1 | 3 | 4.955994 | 0.005829 | 0.030599 | up |
| ENSMUS( | Hist2h4 | 14 | 20 | 5 | 4 | 3 | 5 | 3.154249 | 0.005842 | 0.030649 | up |
| ENSMUS( | Snx29 | 35 | 32 | 42 | 16 | 24 | 23 | 1.729018 | 0.005877 | 0.030793 | up |
| ENSMUS( | Gm3002 | 21 | 28 | 23 | 9 | 12 | 15 | 1.987813 | 0.005967 | 0.0312 | up |
| ENSMUS( | Ranbp17 | 97 | 107 | 80 | 63 | 41 | 80 | 1.526192 | 0.005983 | 0.031276 | up |
| ENSMUS( | Dnah17 | 9 | 17 | 16 | 4 | 4 | 8 | 2.636241 | 0.005998 | 0.03133 | up |
| ENSMUS( | Vmn2r84 | 34 | 35 | 25 | 11 | 22 | 14 | 1.966497 | 0.006042 | 0.031541 | up |
| ENSMUS( | Insl5 | 11 | 23 | 18 | 2 | 7 | 0 | 5.655137 | 0.006071 | 0.031672 | up |
| ENSMUS( | Mthfs | 45 | 43 | 47 | 29 | 31 | 23 | 1.609137 | 0.006073 | 0.031673 | up |
| ENSMUS( | Fancb | 49 | 39 | 44 | 19 | 33 | 24 | 1.722165 | 0.006117 | 0.03184 | up |
| ENSMUS( | Clvs1 | 125 | 184 | 152 | 70 | 117 | 84 | 1.684209 | 0.006127 | 0.031885 | up |

| | | | | | | | | | | | |
|---|---|---|---|---|---|---|---|---|---|---|---|
| ENSMUS( | Col14a1 | 22 | 29 | 21 | 9 | 6 | 16 | 2.309556 | 0.006347 | 0.032855 | up |
| ENSMUS( | Clcn1 | 41 | 33 | 29 | 22 | 16 | 21 | 1.719689 | 0.006597 | 0.034007 | up |
| ENSMUS( | Atrip | 10 | 14 | 6 | 4 | 2 | 3 | 3.242875 | 0.00671 | 0.03448 | up |
| ENSMUS( | Uba7 | 40 | 44 | 41 | 22 | 22 | 32 | 1.635656 | 0.006997 | 0.035659 | up |
| ENSMUS( | Gm9821 | 15 | 7 | 9 | 2 | 1 | 5 | 3.879308 | 0.007079 | 0.036024 | up |
| ENSMUS( | Slc39a2 | 36 | 50 | 35 | 27 | 27 | 19 | 1.626349 | 0.007102 | 0.036113 | up |
| ENSMUS( | Gm10767 | 30 | 45 | 42 | 22 | 13 | 27 | 1.881392 | 0.007116 | 0.036172 | up |
| ENSMUS( | Krt20 | 11 | 17 | 12 | 5 | 7 | 2 | 2.792669 | 0.007131 | 0.036236 | up |
| ENSMUS( | Gm14443 | 53 | 49 | 37 | 30 | 29 | 28 | 1.569343 | 0.00719 | 0.036502 | up |
| ENSMUS( | Ak9 | 41 | 39 | 33 | 24 | 23 | 20 | 1.659282 | 0.007273 | 0.036828 | up |
| ENSMUS( | Klf8 | 51 | 69 | 48 | 43 | 33 | 34 | 1.500591 | 0.00731 | 0.036979 | up |
| ENSMUS( | Ttc21a | 63 | 51 | 37 | 37 | 23 | 27 | 1.697373 | 0.007423 | 0.037432 | up |
| ENSMUS( | Pcdhgc4 | 66 | 74 | 66 | 29 | 54 | 45 | 1.596149 | 0.00748 | 0.03768 | up |
| ENSMUS( | Tube1 | 50 | 58 | 55 | 32 | 43 | 30 | 1.535594 | 0.007553 | 0.038014 | up |
| ENSMUS( | Gimap8 | 30 | 30 | 41 | 19 | 17 | 20 | 1.802647 | 0.007586 | 0.038153 | up |
| ENSMUS( | Zfp973 | 9 | 5 | 12 | 3 | 2 | 0 | 5.123739 | 0.007703 | 0.038652 | up |
| ENSMUS( | Ano5 | 57 | 63 | 45 | 35 | 32 | 41 | 1.505965 | 0.007793 | 0.039005 | up |
| ENSMUS( | Pcdhgb5 | 32 | 35 | 18 | 11 | 17 | 17 | 1.851828 | 0.008132 | 0.040428 | up |
| ENSMUS( | Slc10a1 | 3 | 7 | 3 | 1 | 0 | 0 | 12.36312 | 0.008212 | 0.04077 | up |
| ENSMUS( | 4930590J( | 26 | 21 | 22 | 11 | 12 | 12 | 1.951363 | 0.008247 | 0.040928 | up |
| ENSMUS( | Trim43b | 9 | 8 | 3 | 2 | 2 | 0 | 4.763403 | 0.008333 | 0.041258 | up |
| ENSMUS( | Dchs2 | 17 | 11 | 8 | 4 | 6 | 3 | 2.698756 | 0.008377 | 0.041429 | up |
| ENSMUS( | Pm20d2 | 32 | 21 | 37 | 14 | 12 | 21 | 1.925081 | 0.008515 | 0.042018 | up |
| ENSMUS( | Gk5 | 57 | 100 | 80 | 48 | 49 | 58 | 1.517693 | 0.008573 | 0.042269 | up |
| ENSMUS( | Fam81b | 25 | 33 | 34 | 18 | 17 | 17 | 1.758 | 0.008785 | 0.043195 | up |
| ENSMUS( | Gapdhs | 33 | 32 | 15 | 14 | 10 | 16 | 1.953369 | 0.008826 | 0.043352 | up |
| ENSMUS( | 2900055J2 | 26 | 32 | 16 | 12 | 12 | 14 | 1.906293 | 0.009047 | 0.04422 | up |
| ENSMUS( | Naip1 | 10 | 10 | 9 | 2 | 3 | 4 | 3.206092 | 0.009168 | 0.044759 | up |
| ENSMUS( | Tert | 11 | 17 | 14 | 7 | 3 | 7 | 2.450448 | 0.009172 | 0.044762 | up |
| ENSMUS( | Dqx1 | 21 | 13 | 10 | 5 | 6 | 7 | 2.402025 | 0.009366 | 0.045581 | up |
| ENSMUS( | Dnah11 | 54 | 46 | 37 | 30 | 34 | 23 | 1.543649 | 0.009555 | 0.046358 | up |
| ENSMUS( | Clic3 | 27 | 36 | 21 | 7 | 19 | 14 | 2.067684 | 0.010091 | 0.048588 | up |
| ENSMUS( | Otogl | 49 | 40 | 46 | 20 | 35 | 26 | 1.654405 | 0.010559 | 0.050503 | up |
| ENSMUS( | Nlrc5 | 17 | 36 | 16 | 10 | 9 | 13 | 2.116929 | 0.011171 | 0.052968 | up |
| ENSMUS( | Gm28729 | 26 | 24 | 23 | 10 | 13 | 16 | 1.859443 | 0.011299 | 0.053441 | up |
| ENSMUS( | Ccdc167 | 66 | 72 | 53 | 30 | 52 | 41 | 1.53085 | 0.011971 | 0.056082 | up |
| ENSMUS( | Eps8l1 | 9 | 17 | 20 | 7 | 6 | 6 | 2.420403 | 0.012022 | 0.056293 | up |
| ENSMUS( | Kif20a | 26 | 29 | 23 | 16 | 16 | 11 | 1.780978 | 0.012332 | 0.057468 | up |
| ENSMUS( | Prss56 | 29 | 37 | 24 | 22 | 12 | 14 | 1.834448 | 0.012391 | 0.05767 | up |
| ENSMUS( | Efhc2 | 68 | 59 | 51 | 47 | 29 | 39 | 1.523193 | 0.012445 | 0.057869 | up |
| ENSMUS( | Gm3264 | 10 | 11 | 14 | 2 | 4 | 7 | 2.721085 | 0.012593 | 0.058431 | up |
| ENSMUS( | Zfp820 | 23 | 30 | 18 | 11 | 13 | 14 | 1.837709 | 0.012767 | 0.05914 | up |
| ENSMUS( | Actbl2 | 7 | 13 | 10 | 4 | 2 | 0 | 4.844317 | 0.012921 | 0.059661 | up |
| ENSMUS( | Gucy2f | 20 | 30 | 29 | 15 | 18 | 11 | 1.777433 | 0.013119 | 0.060403 | up |
| ENSMUS( | Bpifc | 9 | 0 | 4 | 0 | 1 | 0 | 12.76332 | 0.013351 | 0.061213 | up |
| ENSMUS( | Clnk | 7 | 1 | 7 | 1 | 1 | 0 | 7.414716 | 0.013382 | 0.061288 | up |
| ENSMUS( | Fbxo48 | 5 | 15 | 3 | 3 | 1 | 1 | 4.395134 | 0.013432 | 0.061463 | up |
| ENSMUS( | Plag1 | 35 | 31 | 33 | 13 | 25 | 17 | 1.784544 | 0.013603 | 0.062028 | up |
| ENSMUS( | Sec1 | 32 | 44 | 33 | 22 | 25 | 20 | 1.602794 | 0.013602 | 0.062028 | up |
| ENSMUS( | Corin | 19 | 23 | 21 | 14 | 7 | 5 | 2.374598 | 0.014415 | 0.065155 | up |
| ENSMUS( | Mapkapk5 | 10 | 15 | 9 | 5 | 4 | 4 | 2.563383 | 0.014708 | 0.066182 | up |
| ENSMUS( | Bspry | 35 | 36 | 31 | 21 | 15 | 26 | 1.629983 | 0.014793 | 0.06647 | up |
| ENSMUS( | Serpina3f | 4 | 18 | 13 | 4 | 5 | 3 | 2.886695 | 0.014965 | 0.067069 | up |
| ENSMUS( | Kif24 | 25 | 20 | 21 | 11 | 8 | 16 | 1.875959 | 0.015058 | 0.06742 | up |
| ENSMUS( | Nup210l | 20 | 15 | 14 | 8 | 7 | 8 | 2.100081 | 0.01508 | 0.067509 | up |
| ENSMUS( | BC016579 | 8 | 11 | 6 | 3 | 3 | 0 | 4.008535 | 0.01573 | 0.069886 | up |
| ENSMUS( | Zfp990 | 45 | 28 | 34 | 14 | 18 | 28 | 1.778184 | 0.01596 | 0.070772 | up |
| ENSMUS( | Pcdhac1 | 30 | 10 | 19 | 9 | 7 | 11 | 2.171007 | 0.016325 | 0.072118 | up |
| ENSMUS( | Sds | 10 | 9 | 13 | 3 | 5 | 4 | 2.667644 | 0.016381 | 0.072318 | up |
| ENSMUS( | Prox2 | 46 | 41 | 35 | 26 | 30 | 16 | 1.660455 | 0.016394 | 0.072355 | up |
| ENSMUS( | Sap18b | 47 | 30 | 34 | 36 | 7 | 5 | 2.242697 | 0.016484 | 0.072718 | up |
| ENSMUS( | Ifi44 | 37 | 20 | 25 | 13 | 20 | 12 | 1.796432 | 0.016573 | 0.073043 | up |
| ENSMUS( | Wnt3a | 8 | 7 | 5 | 2 | 1 | 2 | 3.931324 | 0.017098 | 0.07498 | up |
| ENSMUS( | 2310007B( | 5 | 7 | 4 | 0 | 2 | 1 | 5.265717 | 0.017521 | 0.076403 | up |
| ENSMUS( | Wdr49 | 28 | 22 | 26 | 18 | 10 | 15 | 1.750875 | 0.017689 | 0.077007 | up |
| ENSMUS( | Pgpep1l | 13 | 18 | 20 | 8 | 7 | 10 | 2.039852 | 0.018311 | 0.079287 | up |
| ENSMUS( | Jhy | 26 | 30 | 25 | 15 | 12 | 20 | 1.70935 | 0.018772 | 0.080896 | up |
| ENSMUS( | Oacyl | 33 | 17 | 14 | 12 | 10 | 9 | 2.013829 | 0.018771 | 0.080896 | up |
| ENSMUS( | Adamts16 | 35 | 43 | 41 | 30 | 26 | 21 | 1.526389 | 0.020212 | 0.086204 | up |
| ENSMUS( | Slc19a3 | 53 | 54 | 39 | 28 | 25 | 42 | 1.519218 | 0.020236 | 0.08629 | up |
| ENSMUS( | 1810010D | 4 | 3 | 4 | 0 | 1 | 0 | 10.84708 | 0.020351 | 0.08674 | up |
| ENSMUS( | Guca1b | 4 | 3 | 4 | 0 | 0 | 1 | 11.34847 | 0.020385 | 0.086805 | up |
| ENSMUS( | 1700020D | 6 | 3 | 2 | 0 | 0 | 1 | 11.12258 | 0.020553 | 0.087354 | up |
| ENSMUS( | Gm10184 | 22 | 17 | 8 | 7 | 7 | 7 | 2.176497 | 0.020624 | 0.087556 | up |
| ENSMUS( | Tm6sf2 | 23 | 36 | 24 | 14 | 11 | 22 | 1.751163 | 0.020831 | 0.088279 | up |
| ENSMUS( | Olfr1152 | 7 | 5 | 11 | 1 | 3 | 3 | 3.335464 | 0.021055 | 0.089092 | up |
| ENSMUS( | Gipr | 20 | 21 | 19 | 13 | 7 | 12 | 1.854132 | 0.021698 | 0.091308 | up |
| ENSMUS( | Tmem221 | 5 | 11 | 15 | 3 | 4 | 4 | 2.844267 | 0.021736 | 0.091451 | up |
| ENSMUS( | Dcdc2c | 1 | 4 | 6 | 0 | 0 | 1 | 11.56771 | 0.022078 | 0.092695 | up |
| ENSMUS( | Dennd2d | 21 | 16 | 6 | 7 | 5 | 6 | 2.31241 | 0.022621 | 0.094609 | up |

| | | | | | | | | | | | |
|---|---|---|---|---|---|---|---|---|---|---|---|
| ENSMUS( | Cldn9 | 32 | 24 | 22 | 10 | 20 | 9 | 1.964071 | 0.022613 | 0.094609 | up |
| ENSMUS( | Ghsr | 42 | 37 | 39 | 24 | 21 | 32 | 1.523747 | 0.023217 | 0.096656 | up |
| ENSMUS( | Map4k1 | 29 | 30 | 26 | 8 | 12 | 23 | 1.975246 | 0.023295 | 0.096896 | up |
| ENSMUS( | Cage1 | 58 | 53 | 36 | 30 | 23 | 42 | 1.5253 | 0.023521 | 0.097764 | up |
| ENSMUS( | Chek1 | 28 | 43 | 40 | 22 | 24 | 24 | 1.575901 | 0.024158 | 0.09985 | up |
| ENSMUS( | Kcnrg | 19 | 8 | 11 | 5 | 5 | 6 | 2.34811 | 0.024236 | 0.100098 | up |
| ENSMUS( | Slc3a1 | 4 | 4 | 5 | 0 | 1 | 1 | 6.568006 | 0.024414 | 0.100684 | up |
| ENSMUS( | Tcf21 | 5 | 5 | 3 | 1 | 0 | 1 | 6.388906 | 0.024598 | 0.101253 | up |
| ENSMUS( | Traf3ip3 | 14 | 23 | 11 | 4 | 4 | 11 | 2.504704 | 0.024847 | 0.102174 | up |
| ENSMUS( | Gm5737 | 7 | 4 | 6 | 0 | 2 | 2 | 4.281607 | 0.025401 | 0.103965 | up |
| ENSMUS( | Lmntd2 | 6 | 4 | 5 | 2 | 1 | 0 | 4.849551 | 0.025666 | 0.104924 | up |
| ENSMUS( | Gm3526 | 4 | 6 | 5 | 2 | 0 | 1 | 4.916821 | 0.025716 | 0.105086 | up |
| ENSMUS( | Chaf1a | 31 | 35 | 31 | 16 | 18 | 27 | 1.582231 | 0.02632 | 0.107012 | up |
| ENSMUS( | Adam21 | 18 | 9 | 11 | 2 | 8 | 4 | 2.681616 | 0.026867 | 0.108916 | up |
| ENSMUS( | Ccdc7b | 17 | 22 | 17 | 13 | 5 | 9 | 2.040573 | 0.026864 | 0.108916 | up |
| ENSMUS( | Plbd1 | 25 | 31 | 28 | 20 | 14 | 17 | 1.628955 | 0.027141 | 0.109837 | up |
| ENSMUS( | Ptgds | 15333 | 15353 | 14647 | 30371 | 29741 | 32459 | 0.484878 | 1.2E-93 | 1.78E-90 | down |
| ENSMUS( | Atp6v1g2 | 5224 | 5013 | 4853 | 9259 | 9057 | 9428 | 0.538148 | 3.02E-71 | 2.93E-68 | down |
| ENSMUS( | H2-K1 | 71 | 82 | 64 | 273 | 349 | 341 | 0.222601 | 1.63E-62 | 1.15E-59 | down |
| ENSMUS( | Apod | 3547 | 3449 | 3304 | 5633 | 5552 | 5853 | 0.598208 | 1.8E-55 | 1.18E-52 | down |
| ENSMUS( | Ptms | 5134 | 5072 | 4282 | 7786 | 8142 | 8059 | 0.59583 | 1.79E-55 | 1.18E-52 | down |
| ENSMUS( | Cnih2 | 6513 | 6545 | 6079 | 9988 | 9810 | 10249 | 0.629903 | 2E-46 | 1.06E-43 | down |
| ENSMUS( | Gm8797 | 125 | 132 | 133 | 315 | 375 | 380 | 0.361921 | 6.01E-45 | 2.96E-42 | down |
| ENSMUS( | Wbp2 | 4753 | 4753 | 4137 | 7003 | 6795 | 7432 | 0.634818 | 2.15E-44 | 1.04E-41 | down |
| ENSMUS( | Ctxn1 | 4596 | 4386 | 3961 | 6983 | 6564 | 6781 | 0.628567 | 8.11E-42 | 3.68E-39 | down |
| ENSMUS( | Syn1 | 6006 | 5917 | 5293 | 9198 | 9049 | 8781 | 0.6287 | 1.95E-41 | 8.58E-39 | down |
| ENSMUS( | Vegfb | 776 | 749 | 654 | 1167 | 1260 | 1344 | 0.571027 | 1.26E-40 | 5.15E-38 | down |
| ENSMUS( | Epn1 | 6253 | 6293 | 5316 | 8761 | 8938 | 9166 | 0.656165 | 1.84E-38 | 7.19E-36 | down |
| ENSMUS( | Scn1b | 5100 | 5221 | 4551 | 7103 | 7451 | 7582 | 0.66377 | 1.51E-36 | 5.1E-34 | down |
| ENSMUS( | Necab2 | 1119 | 1098 | 1055 | 1614 | 1734 | 1890 | 0.619 | 4.96E-36 | 1.64E-33 | down |
| ENSMUS( | Cdk5r2 | 2604 | 2671 | 2178 | 3934 | 3732 | 3989 | 0.630571 | 1.9E-35 | 6.15E-33 | down |
| ENSMUS( | Cxcl14 | 1496 | 1540 | 1382 | 2326 | 2214 | 2311 | 0.637217 | 3.82E-35 | 1.21E-32 | down |
| ENSMUS( | Calb2 | 729 | 661 | 612 | 1032 | 1114 | 1155 | 0.599549 | 2.17E-32 | 6.28E-30 | down |
| ENSMUS( | Fmod | 177 | 173 | 142 | 354 | 360 | 366 | 0.449079 | 4.84E-32 | 1.35E-29 | down |
| ENSMUS( | Kctd13 | 1267 | 1294 | 1195 | 1828 | 1956 | 1979 | 0.644871 | 1.15E-31 | 3.11E-29 | down |
| ENSMUS( | Tmsb10 | 1710 | 1722 | 1583 | 2452 | 2394 | 2669 | 0.660343 | 2.56E-31 | 6.79E-29 | down |
| ENSMUS( | Sncb | 3998 | 3921 | 3236 | 5678 | 5798 | 5951 | 0.6312 | 8.92E-31 | 2.3E-28 | down |
| ENSMUS( | Hapln4 | 1324 | 1294 | 1214 | 1841 | 1988 | 1981 | 0.652463 | 3.19E-30 | 7.9E-28 | down |
| ENSMUS( | Eno1b | 1052 | 1063 | 1044 | 1662 | 1612 | 1791 | 0.618129 | 1.48E-29 | 3.46E-27 | down |
| ENSMUS( | Fscn1 | 1573 | 1636 | 1380 | 2301 | 2220 | 2312 | 0.662774 | 1.49E-29 | 3.47E-27 | down |
| ENSMUS( | Me1 | 1497 | 1633 | 1431 | 2177 | 2281 | 2316 | 0.665727 | 3.85E-29 | 8.72E-27 | down |
| ENSMUS( | Dact3 | 1557 | 1528 | 1396 | 2471 | 2217 | 2346 | 0.629176 | 1.41E-28 | 3.07E-26 | down |
| ENSMUS( | Sf3b4 | 562 | 557 | 466 | 867 | 863 | 864 | 0.602463 | 1.07E-27 | 2.08E-25 | down |
| ENSMUS( | Htra1 | 2625 | 2668 | 2583 | 3862 | 3910 | 4080 | 0.658166 | 2.17E-27 | 4.07E-25 | down |
| ENSMUS( | Prrt1 | 2100 | 2017 | 1850 | 3259 | 2999 | 2937 | 0.640383 | 3.05E-27 | 5.64E-25 | down |
| ENSMUS( | Zfp385a | 596 | 575 | 496 | 876 | 923 | 894 | 0.610682 | 4.9E-27 | 9E-25 | down |
| ENSMUS( | Cxx1a | 680 | 758 | 661 | 1115 | 1069 | 1105 | 0.630678 | 6.81E-27 | 1.22E-24 | down |
| ENSMUS( | Mt1 | 6372 | 6005 | 5950 | 9235 | 9047 | 9659 | 0.649428 | 8.21E-27 | 1.44E-24 | down |
| ENSMUS( | Rnf208 | 1434 | 1318 | 1159 | 1908 | 1972 | 2143 | 0.641345 | 9.79E-26 | 1.57E-23 | down |
| ENSMUS( | mt-Atp6 | 171 | 146 | 159 | 185 | 930 | 147 | 0.371469 | 2E-25 | 3.14E-23 | down |
| ENSMUS( | Spp1 | 172 | 191 | 161 | 343 | 321 | 375 | 0.498608 | 5.46E-25 | 8.26E-23 | down |
| ENSMUS( | Hbb-bs | 1269 | 1436 | 995 | 2005 | 2164 | 2356 | 0.558422 | 2.76E-24 | 3.92E-22 | down |
| ENSMUS( | Laptm5 | 667 | 611 | 577 | 899 | 949 | 995 | 0.64536 | 7.24E-23 | 9.6E-21 | down |
| ENSMUS( | Ass1 | 359 | 374 | 287 | 537 | 605 | 614 | 0.572622 | 8.69E-23 | 1.14E-20 | down |
| ENSMUS( | Fxyd7 | 537 | 592 | 527 | 838 | 821 | 911 | 0.637716 | 1.01E-22 | 1.3E-20 | down |
| ENSMUS( | Epop | 276 | 287 | 264 | 522 | 494 | 474 | 0.548251 | 1.27E-22 | 1.6E-20 | down |
| ENSMUS( | Vgf | 979 | 1003 | 878 | 1525 | 1423 | 1426 | 0.645238 | 1.76E-22 | 2.19E-20 | down |
| ENSMUS( | Begain | 1528 | 1643 | 1485 | 2540 | 2183 | 2305 | 0.654493 | 3.44E-22 | 4.21E-20 | down |
| ENSMUS( | Abhd17a | 1298 | 1440 | 1292 | 2152 | 1945 | 2143 | 0.638785 | 4.63E-21 | 5.21E-19 | down |
| ENSMUS( | Elmo1 | 813 | 737 | 674 | 1080 | 1133 | 1084 | 0.665814 | 8.52E-21 | 9.43E-19 | down |
| ENSMUS( | Trp53i11 | 892 | 871 | 743 | 1130 | 1330 | 1338 | 0.651811 | 8.42E-20 | 8.08E-18 | down |
| ENSMUS( | Zyx | 496 | 461 | 434 | 717 | 730 | 692 | 0.642263 | 9.97E-20 | 9.43E-18 | down |
| ENSMUS( | Hmga1-rs1 | 262 | 245 | 238 | 375 | 437 | 451 | 0.584385 | 1.01E-18 | 8.67E-17 | down |
| ENSMUS( | Rab4a | 566 | 544 | 472 | 733 | 779 | 847 | 0.662657 | 1.88E-18 | 1.57E-16 | down |
| ENSMUS( | Efemp1 | 307 | 292 | 285 | 456 | 466 | 509 | 0.611895 | 2.67E-18 | 2.19E-16 | down |
| ENSMUS( | C1qc | 527 | 494 | 500 | 794 | 731 | 776 | 0.654344 | 3.07E-18 | 2.48E-16 | down |
| ENSMUS( | H2-D1 | 504 | 489 | 474 | 686 | 758 | 753 | 0.661142 | 4.88E-18 | 3.84E-16 | down |
| ENSMUS( | Eif3f | 1726 | 1840 | 1351 | 2334 | 2594 | 2537 | 0.648446 | 2.27E-17 | 1.68E-15 | down |
| ENSMUS( | Plvap | 140 | 150 | 116 | 244 | 246 | 279 | 0.520891 | 2.42E-17 | 1.78E-15 | down |
| ENSMUS( | Trnp1 | 1578 | 1694 | 1636 | 2658 | 2461 | 2544 | 0.63408 | 6.61E-17 | 4.54E-15 | down |
| ENSMUS( | H2-Aa | 30 | 29 | 34 | 101 | 99 | 89 | 0.319423 | 7.17E-17 | 4.91E-15 | down |
| ENSMUS( | Pcdh8 | 549 | 551 | 451 | 695 | 795 | 831 | 0.659829 | 1.27E-16 | 8.5E-15 | down |
| ENSMUS( | Slc6a20a | 239 | 271 | 246 | 418 | 408 | 401 | 0.609331 | 1.69E-16 | 1.1E-14 | down |
| ENSMUS( | Serpinf1 | 265 | 247 | 210 | 408 | 364 | 408 | 0.603318 | 2.25E-16 | 1.43E-14 | down |
| ENSMUS( | Trim8 | 1180 | 1136 | 882 | 1621 | 1607 | 1589 | 0.65314 | 2.57E-16 | 1.63E-14 | down |
| ENSMUS( | Slc22a6 | 95 | 97 | 81 | 163 | 198 | 205 | 0.476766 | 3.67E-16 | 2.27E-14 | down |
| ENSMUS( | Gltp | 485 | 505 | 438 | 668 | 781 | 727 | 0.648339 | 5.72E-16 | 3.45E-14 | down |
| ENSMUS( | Igsf21 | 281 | 261 | 237 | 432 | 390 | 425 | 0.616767 | 6.1E-16 | 3.64E-14 | down |
| ENSMUS( | Tox2 | 335 | 304 | 254 | 460 | 512 | 477 | 0.607153 | 8.79E-16 | 5.15E-14 | down |
| ENSMUS( | Upp2 | 257 | 230 | 212 | 352 | 391 | 393 | 0.608053 | 1.29E-15 | 7.48E-14 | down |

| | | | | | | | | | | | |
|---|---|---|---|---|---|---|---|---|---|---|---|
| ENSMUS( | Rhog | 381 | 375 | 329 | 555 | 583 | 533 | 0.640513 | 1.42E-15 | 8.18E-14 | down |
| ENSMUS( | Rps18 | 399 | 290 | 311 | 689 | 849 | 906 | 0.405458 | 1.61E-15 | 9.26E-14 | down |
| ENSMUS( | Rgma | 758 | 732 | 528 | 1058 | 1095 | 1044 | 0.619964 | 2.92E-15 | 1.58E-13 | down |
| ENSMUS( | Slc13a3 | 318 | 294 | 256 | 435 | 434 | 479 | 0.63579 | 3.64E-15 | 1.94E-13 | down |
| ENSMUS( | Zfp703 | 229 | 227 | 206 | 359 | 351 | 369 | 0.606373 | 3.78E-15 | 2.01E-13 | down |
| ENSMUS( | Sox10 | 545 | 435 | 384 | 754 | 717 | 796 | 0.59305 | 4.52E-15 | 2.39E-13 | down |
| ENSMUS( | Bri3 | 340 | 340 | 335 | 498 | 474 | 563 | 0.65575 | 5.52E-15 | 2.88E-13 | down |
| ENSMUS( | Lyz2 | 221 | 254 | 199 | 337 | 357 | 392 | 0.61297 | 1.17E-14 | 5.82E-13 | down |
| ENSMUS( | Cbln1 | 57 | 47 | 50 | 126 | 105 | 140 | 0.411553 | 1.23E-14 | 6.08E-13 | down |
| ENSMUS( | Pnpla2 | 319 | 290 | 278 | 426 | 450 | 484 | 0.645476 | 1.47E-14 | 7.13E-13 | down |
| ENSMUS( | Tesc | 353 | 338 | 323 | 515 | 489 | 517 | 0.659325 | 1.89E-14 | 9.12E-13 | down |
| ENSMUS( | Foxo6 | 225 | 234 | 174 | 371 | 336 | 350 | 0.588906 | 2.73E-14 | 1.3E-12 | down |
| ENSMUS( | Hba-a1 | 1183 | 1133 | 1148 | 1536 | 1899 | 2007 | 0.631971 | 6.04E-14 | 2.76E-12 | down |
| ENSMUS( | Lcat | 450 | 481 | 491 | 704 | 697 | 789 | 0.644843 | 2.98E-13 | 1.25E-11 | down |
| ENSMUS( | Jund | 313 | 267 | 247 | 487 | 462 | 432 | 0.590112 | 5.42E-13 | 2.18E-11 | down |
| ENSMUS( | Jup | 341 | 325 | 252 | 486 | 491 | 475 | 0.621849 | 6.99E-13 | 2.76E-11 | down |
| ENSMUS( | Gm9008 | 3 | 4 | 1 | 27 | 28 | 28 | 0.093205 | 9.95E-13 | 3.84E-11 | down |
| ENSMUS( | Camk2n2 | 769 | 938 | 868 | 1654 | 1497 | 1495 | 0.54851 | 1.16E-12 | 4.46E-11 | down |
| ENSMUS( | Zic2 | 295 | 296 | 300 | 434 | 425 | 467 | 0.666395 | 1.23E-12 | 4.69E-11 | down |
| ENSMUS( | Atn1 | 178 | 167 | 160 | 274 | 268 | 288 | 0.601923 | 1.36E-12 | 5.15E-11 | down |
| ENSMUS( | Samd1 | 254 | 265 | 205 | 370 | 356 | 381 | 0.64434 | 2.16E-12 | 7.96E-11 | down |
| ENSMUS( | Cldn5 | 141 | 138 | 128 | 223 | 249 | 220 | 0.581015 | 5.77E-12 | 1.98E-10 | down |
| ENSMUS( | Lrrc8a | 214 | 257 | 221 | 356 | 360 | 369 | 0.630804 | 6.02E-12 | 2.06E-10 | down |
| ENSMUS( | Fth1 | 35184 | 41004 | 35304 | 57642 | 56235 | 55778 | 0.649314 | 1.37E-11 | 4.48E-10 | down |
| ENSMUS( | Cd74 | 110 | 138 | 121 | 233 | 199 | 220 | 0.559874 | 2E-11 | 6.41E-10 | down |
| ENSMUS( | Igfbp6 | 136 | 156 | 142 | 228 | 225 | 265 | 0.599301 | 2.18E-11 | 6.93E-10 | down |
| ENSMUS( | Chst12 | 165 | 181 | 142 | 258 | 247 | 281 | 0.612624 | 2.71E-11 | 8.52E-10 | down |
| ENSMUS( | Plin4 | 84 | 67 | 53 | 141 | 159 | 139 | 0.456306 | 2.05E-10 | 5.69E-09 | down |
| ENSMUS( | Foxc2 | 12 | 9 | 7 | 41 | 29 | 47 | 0.235505 | 2.33E-10 | 6.38E-09 | down |
| ENSMUS( | Gm5741 | 6 | 6 | 6 | 24 | 31 | 43 | 0.183099 | 2.34E-10 | 6.42E-09 | down |
| ENSMUS( | Gjb2 | 178 | 160 | 164 | 283 | 254 | 256 | 0.625981 | 2.49E-10 | 6.81E-09 | down |
| ENSMUS( | H2-DMa | 243 | 246 | 203 | 313 | 350 | 411 | 0.637187 | 2.69E-10 | 7.29E-09 | down |
| ENSMUS( | Adra2a | 154 | 128 | 133 | 218 | 215 | 242 | 0.608969 | 2.83E-10 | 7.61E-09 | down |
| ENSMUS( | Pcna-ps2 | 36 | 35 | 40 | 76 | 94 | 87 | 0.429512 | 4.04E-10 | 1.05E-08 | down |
| ENSMUS( | Mospd3 | 185 | 207 | 187 | 318 | 280 | 293 | 0.642333 | 4.23E-10 | 1.1E-08 | down |
| ENSMUS( | Necab3 | 210 | 208 | 210 | 324 | 315 | 300 | 0.661906 | 4.58E-10 | 1.18E-08 | down |
| ENSMUS( | Fbln1 | 162 | 163 | 173 | 269 | 240 | 285 | 0.622816 | 4.69E-10 | 1.2E-08 | down |
| ENSMUS( | Paqr6 | 179 | 189 | 182 | 255 | 318 | 297 | 0.626648 | 4.88E-10 | 1.25E-08 | down |
| ENSMUS( | Aldh1a2 | 185 | 223 | 204 | 347 | 301 | 382 | 0.589303 | 4.88E-10 | 1.25E-08 | down |
| ENSMUS( | Slc22a8 | 231 | 229 | 211 | 355 | 330 | 321 | 0.658521 | 5.65E-10 | 1.43E-08 | down |
| ENSMUS( | Fam43b | 139 | 138 | 117 | 213 | 191 | 242 | 0.602731 | 6.19E-10 | 1.55E-08 | down |
| ENSMUS( | Hbb-bt | 281 | 277 | 204 | 351 | 408 | 426 | 0.633369 | 1.1E-09 | 2.64E-08 | down |
| ENSMUS( | Pex14 | 156 | 190 | 165 | 256 | 269 | 269 | 0.636812 | 1.97E-09 | 4.52E-08 | down |
| ENSMUS( | Tcf7l2 | 123 | 129 | 118 | 183 | 203 | 216 | 0.608752 | 2.37E-09 | 5.35E-08 | down |
| ENSMUS( | Sult1a1 | 258 | 240 | 192 | 343 | 353 | 330 | 0.661616 | 2.68E-09 | 5.98E-08 | down |
| ENSMUS( | Hes5 | 58 | 43 | 45 | 95 | 109 | 96 | 0.480668 | 3.01E-09 | 6.66E-08 | down |
| ENSMUS( | Tac2 | 143 | 124 | 111 | 201 | 199 | 209 | 0.612389 | 3.68E-09 | 7.98E-08 | down |
| ENSMUS( | Slc6a12 | 53 | 67 | 50 | 101 | 121 | 111 | 0.503543 | 4.02E-09 | 8.7E-08 | down |
| ENSMUS( | Col1a1 | 118 | 109 | 97 | 180 | 158 | 203 | 0.592037 | 4.15E-09 | 8.92E-08 | down |
| ENSMUS( | Gm11127 | 11 | 7 | 7 | 36 | 36 | 30 | 0.240893 | 4.66E-09 | 9.91E-08 | down |
| ENSMUS( | C1ql1 | 149 | 174 | 129 | 241 | 225 | 232 | 0.637582 | 7.38E-09 | 1.52E-07 | down |
| ENSMUS( | Mid1 | 132 | 132 | 144 | 198 | 240 | 225 | 0.611429 | 8.14E-09 | 1.66E-07 | down |
| ENSMUS( | Foxc1 | 74 | 85 | 76 | 169 | 138 | 133 | 0.527027 | 1.04E-08 | 2.07E-07 | down |
| ENSMUS( | Mypop | 185 | 197 | 153 | 252 | 307 | 267 | 0.638084 | 1.11E-08 | 2.19E-07 | down |
| ENSMUS( | H2afx | 205 | 272 | 290 | 419 | 383 | 435 | 0.617477 | 1.33E-08 | 2.59E-07 | down |
| ENSMUS( | Mex3d | 124 | 147 | 124 | 218 | 192 | 210 | 0.629402 | 1.46E-08 | 2.81E-07 | down |
| ENSMUS( | Akr7a5 | 227 | 253 | 246 | 415 | 333 | 393 | 0.630428 | 1.64E-08 | 3.14E-07 | down |
| ENSMUS( | Crabp2 | 43 | 50 | 45 | 83 | 94 | 98 | 0.497345 | 4.01E-08 | 7.12E-07 | down |
| ENSMUS( | Sox21 | 187 | 173 | 173 | 281 | 238 | 273 | 0.665972 | 4.86E-08 | 8.47E-07 | down |
| ENSMUS( | Prr7 | 170 | 219 | 206 | 394 | 328 | 351 | 0.54933 | 5.32E-08 | 9.18E-07 | down |
| ENSMUS( | Gpr151 | 13 | 31 | 25 | 73 | 76 | 74 | 0.307213 | 6.89E-08 | 1.17E-06 | down |
| ENSMUS( | Ssbp4 | 1256 | 1353 | 1385 | 2143 | 1995 | 1923 | 0.652856 | 6.96E-08 | 1.18E-06 | down |
| ENSMUS( | Nkd1 | 114 | 132 | 122 | 182 | 185 | 204 | 0.638942 | 1.03E-07 | 1.69E-06 | down |
| ENSMUS( | Chchd10 | 245 | 215 | 261 | 380 | 348 | 378 | 0.647939 | 1.05E-07 | 1.72E-06 | down |
| ENSMUS( | Pth1r | 64 | 61 | 71 | 111 | 108 | 129 | 0.560766 | 1.43E-07 | 2.28E-06 | down |
| ENSMUS( | Cdkn1c | 42 | 40 | 35 | 82 | 83 | 73 | 0.484344 | 1.46E-07 | 2.32E-06 | down |
| ENSMUS( | Eomes | 13 | 10 | 6 | 37 | 33 | 28 | 0.288119 | 2.09E-07 | 3.23E-06 | down |
| ENSMUS( | Dlk1 | 69 | 81 | 70 | 105 | 134 | 157 | 0.551499 | 2.7E-07 | 4.09E-06 | down |
| ENSMUS( | Chadl | 103 | 97 | 100 | 170 | 158 | 150 | 0.620818 | 2.82E-07 | 4.26E-06 | down |
| ENSMUS( | Cd248 | 31 | 26 | 25 | 68 | 61 | 54 | 0.441358 | 2.91E-07 | 4.38E-06 | down |
| ENSMUS( | Cdh1 | 19 | 18 | 13 | 40 | 37 | 57 | 0.368284 | 3.1E-07 | 4.63E-06 | down |
| ENSMUS( | Ifi202b | 1 | 2 | 1 | 15 | 9 | 19 | 0.091602 | 3.11E-07 | 4.64E-06 | down |
| ENSMUS( | Ctxn3 | 34 | 54 | 40 | 85 | 80 | 109 | 0.462895 | 3.15E-07 | 4.7E-06 | down |
| ENSMUS( | Ppp1r18 | 171 | 158 | 128 | 245 | 200 | 245 | 0.652423 | 3.82E-07 | 5.61E-06 | down |
| ENSMUS( | Prg4 | 63 | 59 | 46 | 97 | 103 | 103 | 0.54579 | 4.8E-07 | 6.93E-06 | down |
| ENSMUS( | Ddc | 86 | 100 | 76 | 144 | 145 | 137 | 0.605667 | 4.89E-07 | 7.05E-06 | down |
| ENSMUS( | Thbd | 169 | 132 | 122 | 205 | 230 | 234 | 0.624016 | 5.26E-07 | 7.52E-06 | down |
| ENSMUS( | Dap | 104 | 130 | 100 | 155 | 168 | 193 | 0.640045 | 5.28E-07 | 7.55E-06 | down |
| ENSMUS( | Ctgf | 141 | 134 | 123 | 193 | 187 | 214 | 0.662732 | 5.76E-07 | 8.18E-06 | down |
| ENSMUS( | H1fx | 66 | 93 | 58 | 126 | 152 | 134 | 0.517642 | 5.92E-07 | 8.39E-06 | down |

| | | | | | | | | | | | |
|---|---|---|---|---|---|---|---|---|---|---|---|
| ENSMUS( | Fam180a | 38 | 43 | 31 | 91 | 78 | 64 | 0.471188 | 9.26E-07 | 1.26E-05 | down |
| ENSMUS( | Itih2 | 107 | 96 | 101 | 144 | 166 | 164 | 0.635935 | 9.44E-07 | 1.28E-05 | down |
| ENSMUS( | Prr5 | 109 | 97 | 81 | 155 | 141 | 156 | 0.625525 | 1.06E-06 | 1.42E-05 | down |
| ENSMUS( | Medag | 17 | 24 | 22 | 48 | 57 | 42 | 0.42423 | 1.45E-06 | 1.91E-05 | down |
| ENSMUS( | Fam83d | 31 | 29 | 23 | 58 | 58 | 60 | 0.464327 | 1.87E-06 | 2.41E-05 | down |
| ENSMUS( | Omd | 50 | 33 | 29 | 92 | 77 | 77 | 0.446575 | 2.32E-06 | 2.95E-05 | down |
| ENSMUS( | Tgfb1 | 89 | 73 | 73 | 112 | 120 | 151 | 0.608422 | 2.54E-06 | 3.19E-05 | down |
| ENSMUS( | Dnajc30 | 158 | 154 | 175 | 241 | 231 | 260 | 0.661577 | 2.83E-06 | 3.53E-05 | down |
| ENSMUS( | Acta1 | 84 | 84 | 73 | 116 | 133 | 137 | 0.61724 | 3.13E-06 | 3.88E-05 | down |
| ENSMUS( | Tpm2 | 93 | 90 | 75 | 116 | 166 | 147 | 0.593784 | 3.44E-06 | 4.23E-05 | down |
| ENSMUS( | Fam181b | 83 | 89 | 92 | 138 | 123 | 153 | 0.633626 | 3.51E-06 | 4.3E-05 | down |
| ENSMUS( | Fgfbp3 | 89 | 94 | 85 | 129 | 144 | 145 | 0.634501 | 4.03E-06 | 4.89E-05 | down |
| ENSMUS( | Cpne9 | 144 | 143 | 137 | 194 | 244 | 197 | 0.660247 | 5.2E-06 | 6.19E-05 | down |
| ENSMUS( | H2-Q4 | 58 | 52 | 64 | 96 | 99 | 106 | 0.575478 | 6.78E-06 | 7.85E-05 | down |
| ENSMUS( | Clec3b | 57 | 56 | 65 | 100 | 96 | 103 | 0.592025 | 7.34E-06 | 8.42E-05 | down |
| ENSMUS( | Slc2a5 | 78 | 78 | 56 | 108 | 107 | 128 | 0.608666 | 7.47E-06 | 8.55E-05 | down |
| ENSMUS( | Tox | 157 | 107 | 142 | 201 | 232 | 218 | 0.61942 | 8.61E-06 | 9.76E-05 | down |
| ENSMUS( | Samd3 | 5 | 5 | 2 | 14 | 19 | 21 | 0.216803 | 8.82E-06 | 9.97E-05 | down |
| ENSMUS( | Kti12 | 92 | 100 | 101 | 132 | 147 | 163 | 0.658829 | 9.62E-06 | 0.000108 | down |
| ENSMUS( | Dusp28 | 208 | 228 | 267 | 353 | 390 | 342 | 0.644612 | 1.13E-05 | 0.000124 | down |
| ENSMUS( | Pgm5 | 59 | 70 | 49 | 98 | 87 | 111 | 0.592682 | 1.18E-05 | 0.000129 | down |
| ENSMUS( | Irx2 | 3 | 2 | 5 | 15 | 28 | 11 | 0.186062 | 1.27E-05 | 0.000138 | down |
| ENSMUS( | Sphk1 | 30 | 46 | 32 | 63 | 74 | 71 | 0.512349 | 1.72E-05 | 0.000182 | down |
| ENSMUS( | Gp1bb | 71 | 60 | 74 | 141 | 104 | 115 | 0.564832 | 1.83E-05 | 0.000192 | down |
| ENSMUS( | Ccdc153 | 147 | 128 | 102 | 198 | 166 | 193 | 0.665792 | 1.86E-05 | 0.000195 | down |
| ENSMUS( | Gm11627 | 66 | 72 | 65 | 95 | 112 | 117 | 0.620826 | 2.11E-05 | 0.000219 | down |
| ENSMUS( | Itgal | 16 | 14 | 8 | 33 | 43 | 25 | 0.366166 | 2.65E-05 | 0.000271 | down |
| ENSMUS( | F8a | 165 | 217 | 190 | 291 | 316 | 265 | 0.648542 | 3.37E-05 | 0.000336 | down |
| ENSMUS( | Ascl1 | 100 | 99 | 89 | 162 | 144 | 131 | 0.649472 | 3.4E-05 | 0.00034 | down |
| ENSMUS( | Sln | 14 | 18 | 6 | 41 | 43 | 31 | 0.319602 | 3.99E-05 | 0.000392 | down |
| ENSMUS( | Col9a2 | 63 | 80 | 68 | 105 | 108 | 115 | 0.636822 | 4.76E-05 | 0.00046 | down |
| ENSMUS( | Tagln2 | 175 | 144 | 165 | 234 | 273 | 221 | 0.658447 | 5.98E-05 | 0.000565 | down |
| ENSMUS( | Pou4f1 | 3 | 0 | 0 | 26 | 18 | 28 | 0.039921 | 6.09E-05 | 0.000574 | down |
| ENSMUS( | Gpr88 | 86 | 91 | 78 | 158 | 132 | 113 | 0.622496 | 6.2E-05 | 0.000583 | down |
| ENSMUS( | Fgfbp1 | 41 | 32 | 28 | 59 | 71 | 53 | 0.542373 | 6.59E-05 | 0.000615 | down |
| ENSMUS( | Il17d | 95 | 127 | 116 | 169 | 174 | 168 | 0.655538 | 7.39E-05 | 0.000682 | down |
| ENSMUS( | Adamts2 | 107 | 111 | 79 | 120 | 155 | 166 | 0.663997 | 7.9E-05 | 0.000723 | down |
| ENSMUS( | Hhatl | 92 | 100 | 77 | 109 | 144 | 152 | 0.65634 | 9.74E-05 | 0.000871 | down |
| ENSMUS( | Cyp1b1 | 94 | 128 | 96 | 160 | 152 | 163 | 0.660709 | 0.000101 | 0.000895 | down |
| ENSMUS( | Tcf7l1 | 63 | 61 | 43 | 80 | 83 | 104 | 0.616141 | 0.000101 | 0.000901 | down |
| ENSMUS( | Chrna3 | 35 | 34 | 34 | 58 | 56 | 69 | 0.558531 | 0.000106 | 0.000934 | down |
| ENSMUS( | Shb | 75 | 90 | 70 | 129 | 112 | 109 | 0.661071 | 0.000125 | 0.001085 | down |
| ENSMUS( | Rps2-ps6 | 19 | 26 | 17 | 42 | 48 | 35 | 0.486596 | 0.000139 | 0.001198 | down |
| ENSMUS( | Alx4 | 29 | 28 | 20 | 50 | 38 | 60 | 0.512613 | 0.000143 | 0.001229 | down |
| ENSMUS( | Krt1 | 58 | 78 | 42 | 106 | 97 | 109 | 0.558939 | 0.000148 | 0.001263 | down |
| ENSMUS( | Anxa1 | 59 | 65 | 70 | 93 | 108 | 97 | 0.646758 | 0.000149 | 0.001269 | down |
| ENSMUS( | S100a9 | 29 | 28 | 29 | 41 | 56 | 61 | 0.541421 | 0.000151 | 0.001288 | down |
| ENSMUS( | Foxf2 | 24 | 17 | 14 | 36 | 40 | 37 | 0.477978 | 0.000212 | 0.001739 | down |
| ENSMUS( | Gm2564 | 38 | 109 | 45 | 119 | 114 | 120 | 0.532135 | 0.00024 | 0.001934 | down |
| ENSMUS( | Zfp503 | 54 | 76 | 42 | 95 | 98 | 104 | 0.568131 | 0.000258 | 0.002061 | down |
| ENSMUS( | Doc2a | 33 | 43 | 31 | 54 | 64 | 64 | 0.580129 | 0.000305 | 0.002384 | down |
| ENSMUS( | Gabra6 | 3 | 2 | 0 | 12 | 14 | 9 | 0.135757 | 0.000398 | 0.003017 | down |
| ENSMUS( | Ror2 | 7 | 3 | 5 | 16 | 17 | 15 | 0.309484 | 0.000469 | 0.003486 | down |
| ENSMUS( | Foxd1 | 13 | 21 | 12 | 36 | 29 | 31 | 0.469802 | 0.000513 | 0.003779 | down |
| ENSMUS( | Serf1 | 50 | 64 | 64 | 94 | 82 | 98 | 0.645724 | 0.0007 | 0.004934 | down |
| ENSMUS( | Hsf2bp | 2 | 7 | 3 | 12 | 18 | 17 | 0.250858 | 0.000739 | 0.005177 | down |
| ENSMUS( | Rhov | 55 | 64 | 73 | 95 | 114 | 93 | 0.632434 | 0.000764 | 0.005331 | down |
| ENSMUS( | Clec4a2 | 30 | 36 | 24 | 51 | 55 | 48 | 0.573794 | 0.000771 | 0.005372 | down |
| ENSMUS( | Gm5617 | 49 | 33 | 55 | 102 | 76 | 83 | 0.523111 | 0.000777 | 0.005399 | down |
| ENSMUS( | Anpep | 43 | 36 | 48 | 62 | 66 | 80 | 0.60971 | 0.000778 | 0.005403 | down |
| ENSMUS( | Vstm2l | 277 | 357 | 359 | 645 | 536 | 462 | 0.598236 | 0.000793 | 0.005498 | down |
| ENSMUS( | 4933407L2 | 18 | 8 | 9 | 28 | 30 | 32 | 0.382748 | 0.000818 | 0.005652 | down |
| ENSMUS( | Gpr182 | 45 | 28 | 40 | 64 | 57 | 77 | 0.568354 | 0.000822 | 0.005672 | down |
| ENSMUS( | Dnase1l1 | 20 | 22 | 24 | 40 | 41 | 40 | 0.541988 | 0.00091 | 0.006177 | down |
| ENSMUS( | Ubc | 3029 | 3126 | 1599 | 4796 | 4887 | 3813 | 0.559312 | 0.000994 | 0.006674 | down |
| ENSMUS( | Ebf3 | 19 | 21 | 22 | 36 | 37 | 42 | 0.536186 | 0.001016 | 0.006794 | down |
| ENSMUS( | Cenpo | 39 | 31 | 42 | 65 | 62 | 57 | 0.604963 | 0.001091 | 0.007217 | down |
| ENSMUS( | Cox6a2 | 24 | 36 | 31 | 56 | 42 | 55 | 0.589603 | 0.00112 | 0.007383 | down |
| ENSMUS( | Slfn8 | 8 | 12 | 10 | 28 | 17 | 24 | 0.430076 | 0.001141 | 0.007492 | down |
| ENSMUS( | Rin3 | 26 | 29 | 21 | 43 | 48 | 42 | 0.561902 | 0.001184 | 0.007723 | down |
| ENSMUS( | Gm266 | 49 | 51 | 56 | 90 | 75 | 71 | 0.654968 | 0.001215 | 0.007891 | down |
| ENSMUS( | Bmyc | 53 | 66 | 68 | 95 | 81 | 105 | 0.662338 | 0.001315 | 0.008446 | down |
| ENSMUS( | Ncf2 | 40 | 40 | 40 | 63 | 65 | 61 | 0.628537 | 0.001328 | 0.008526 | down |
| ENSMUS( | Wnt6 | 11 | 8 | 8 | 28 | 21 | 16 | 0.407443 | 0.001384 | 0.008849 | down |
| ENSMUS( | Tekt4 | 28 | 35 | 27 | 51 | 49 | 50 | 0.591777 | 0.00148 | 0.009385 | down |
| ENSMUS( | H2-Q6 | 61 | 37 | 55 | 84 | 76 | 94 | 0.599668 | 0.001494 | 0.009456 | down |
| ENSMUS( | Wnt10a | 36 | 43 | 35 | 71 | 52 | 59 | 0.617438 | 0.001505 | 0.009512 | down |
| ENSMUS( | Alox8 | 45 | 41 | 28 | 46 | 64 | 80 | 0.591821 | 0.001516 | 0.00956 | down |
| ENSMUS( | Gng8 | 17 | 20 | 25 | 31 | 41 | 42 | 0.544346 | 0.001559 | 0.009801 | down |
| ENSMUS( | Sh2b2 | 59 | 67 | 42 | 78 | 81 | 93 | 0.655306 | 0.001565 | 0.009829 | down |

| | | | | | | | | | | | |
|---|---|---|---|---|---|---|---|---|---|---|---|
| ENSMUS( | Klf4 | 35 | 36 | 28 | 53 | 45 | 63 | 0.60713 | 0.001635 | 0.010216 | down |
| ENSMUS( | Ramp3 | 40 | 40 | 32 | 61 | 64 | 52 | 0.622088 | 0.001754 | 0.010886 | down |
| ENSMUS( | Btnl2 | 2 | 0 | 1 | 6 | 7 | 8 | 0.142231 | 0.001848 | 0.011384 | down |
| ENSMUS( | Heyl | 47 | 41 | 50 | 58 | 75 | 76 | 0.657823 | 0.001872 | 0.011501 | down |
| ENSMUS( | Ret | 31 | 41 | 28 | 50 | 57 | 54 | 0.61159 | 0.00198 | 0.012071 | down |
| ENSMUS( | Nat1 | 22 | 28 | 21 | 45 | 30 | 54 | 0.544543 | 0.001985 | 0.012099 | down |
| ENSMUS( | Mrgprf | 25 | 14 | 26 | 46 | 34 | 62 | 0.459225 | 0.002197 | 0.013228 | down |
| ENSMUS( | Nat8f2 | 32 | 26 | 27 | 43 | 50 | 48 | 0.596665 | 0.002221 | 0.013358 | down |
| ENSMUS( | Gm10762 | 60 | 53 | 46 | 62 | 90 | 86 | 0.660256 | 0.002365 | 0.014147 | down |
| ENSMUS( | Cxcl5 | 18 | 23 | 10 | 34 | 39 | 30 | 0.481757 | 0.002403 | 0.014348 | down |
| ENSMUS( | Lsm2 | 78 | 111 | 78 | 147 | 131 | 117 | 0.664461 | 0.002887 | 0.016823 | down |
| ENSMUS( | Tuba8 | 60 | 81 | 74 | 121 | 86 | 115 | 0.662038 | 0.002895 | 0.01686 | down |
| ENSMUS( | Tmem252 | 71 | 56 | 41 | 84 | 83 | 91 | 0.639244 | 0.003034 | 0.017553 | down |
| ENSMUS( | Mettl11b | 29 | 31 | 18 | 47 | 42 | 41 | 0.5865 | 0.003065 | 0.017715 | down |
| ENSMUS( | Hlx | 5 | 2 | 3 | 7 | 10 | 17 | 0.293416 | 0.003124 | 0.018005 | down |
| ENSMUS( | Tusc1 | 76 | 70 | 97 | 131 | 104 | 131 | 0.662916 | 0.003132 | 0.018036 | down |
| ENSMUS( | Ly6g6d | 2 | 1 | 2 | 6 | 10 | 8 | 0.208376 | 0.003181 | 0.018283 | down |
| ENSMUS( | Ccdc3 | 15 | 17 | 19 | 30 | 32 | 32 | 0.539992 | 0.003247 | 0.018606 | down |
| ENSMUS( | Fancf | 33 | 38 | 24 | 37 | 62 | 59 | 0.592206 | 0.00327 | 0.018726 | down |
| ENSMUS( | Ecel1 | 48 | 49 | 38 | 69 | 61 | 71 | 0.661738 | 0.003306 | 0.01889 | down |
| ENSMUS( | Cbln3 | 11 | 9 | 9 | 17 | 18 | 28 | 0.457672 | 0.003412 | 0.019424 | down |
| ENSMUS( | Cnmd | 60 | 60 | 48 | 102 | 71 | 78 | 0.657703 | 0.003607 | 0.020371 | down |
| ENSMUS( | Fermt3 | 88 | 56 | 59 | 96 | 110 | 100 | 0.654269 | 0.003694 | 0.020799 | down |
| ENSMUS( | Il1rn | 0 | 0 | 1 | 2 | 5 | 8 | 0.071939 | 0.003743 | 0.021037 | down |
| ENSMUS( | Egflam | 33 | 20 | 22 | 48 | 42 | 39 | 0.5723 | 0.003744 | 0.021037 | down |
| ENSMUS( | Ccl5 | 12 | 10 | 10 | 32 | 19 | 19 | 0.449422 | 0.003816 | 0.021368 | down |
| ENSMUS( | Pcsk1n | 2053 | 2637 | 3240 | 8427 | 4161 | 4215 | 0.467973 | 0.00401 | 0.022279 | down |
| ENSMUS( | Mcpt4 | 9 | 6 | 12 | 20 | 23 | 18 | 0.443341 | 0.004027 | 0.022342 | down |
| ENSMUS( | Zfp575 | 56 | 49 | 59 | 109 | 72 | 68 | 0.651239 | 0.004071 | 0.022558 | down |
| ENSMUS( | Gpr50 | 7 | 7 | 3 | 11 | 16 | 17 | 0.377396 | 0.004208 | 0.023157 | down |
| ENSMUS( | Cpz | 1 | 1 | 1 | 6 | 6 | 7 | 0.156681 | 0.004327 | 0.023712 | down |
| ENSMUS( | Hs3st3b1 | 10 | 6 | 9 | 19 | 15 | 22 | 0.444837 | 0.004416 | 0.024142 | down |
| ENSMUS( | Gpr84 | 4 | 11 | 8 | 21 | 21 | 14 | 0.405198 | 0.004599 | 0.025027 | down |
| ENSMUS( | Nppc | 49 | 54 | 43 | 54 | 79 | 86 | 0.660205 | 0.004735 | 0.025674 | down |
| ENSMUS( | Vnn1 | 10 | 13 | 6 | 18 | 15 | 33 | 0.432551 | 0.004785 | 0.025923 | down |
| ENSMUS( | Scarf2 | 36 | 36 | 33 | 52 | 56 | 53 | 0.644545 | 0.004911 | 0.026509 | down |
| ENSMUS( | Cd79b | 3 | 3 | 4 | 10 | 7 | 15 | 0.313863 | 0.004924 | 0.026565 | down |
| ENSMUS( | Ccdc87 | 34 | 31 | 36 | 42 | 51 | 63 | 0.645831 | 0.004937 | 0.026622 | down |
| ENSMUS( | Emilin1 | 25 | 25 | 21 | 38 | 40 | 40 | 0.593679 | 0.004958 | 0.02671 | down |
| ENSMUS( | A230065H | 33 | 40 | 41 | 48 | 62 | 62 | 0.65978 | 0.004992 | 0.026831 | down |
| ENSMUS( | Sox3 | 17 | 12 | 17 | 32 | 30 | 23 | 0.536407 | 0.005019 | 0.026946 | down |
| ENSMUS( | Fcgbp | 17 | 13 | 21 | 33 | 33 | 28 | 0.541207 | 0.005041 | 0.027022 | down |
| ENSMUS( | Slc47a1 | 46 | 45 | 31 | 73 | 61 | 52 | 0.641517 | 0.005156 | 0.027524 | down |
| ENSMUS( | Tmem52 | 12 | 9 | 23 | 33 | 28 | 41 | 0.43791 | 0.005343 | 0.028423 | down |
| ENSMUS( | Pdzph1 | 25 | 30 | 20 | 40 | 32 | 51 | 0.601502 | 0.005405 | 0.028685 | down |
| ENSMUS( | Six1 | 4 | 5 | 3 | 7 | 15 | 13 | 0.33776 | 0.005536 | 0.029328 | down |
| ENSMUS( | Cyp4a12b | 0 | 0 | 1 | 3 | 5 | 6 | 0.076588 | 0.005609 | 0.029632 | down |
| ENSMUS( | Il13ra2 | 18 | 20 | 19 | 34 | 34 | 31 | 0.569544 | 0.005675 | 0.029932 | down |
| ENSMUS( | Gm6563 | 39 | 22 | 25 | 58 | 56 | 40 | 0.548496 | 0.005955 | 0.031147 | down |
| ENSMUS( | Rhoj | 71 | 44 | 50 | 74 | 85 | 91 | 0.653163 | 0.005993 | 0.031317 | down |
| ENSMUS( | Slc5a7 | 21 | 19 | 15 | 30 | 35 | 31 | 0.563576 | 0.00609 | 0.031743 | down |
| ENSMUS( | Tnnt2 | 21 | 30 | 21 | 42 | 41 | 35 | 0.599971 | 0.006096 | 0.031756 | down |
| ENSMUS( | Ccl21a | 1 | 3 | 0 | 6 | 14 | 8 | 0.135962 | 0.006284 | 0.0326 | down |
| ENSMUS( | Best1 | 5 | 5 | 7 | 17 | 8 | 18 | 0.396041 | 0.007013 | 0.035731 | down |
| ENSMUS( | Nxph4 | 13 | 14 | 11 | 23 | 24 | 25 | 0.520548 | 0.007687 | 0.038586 | down |
| ENSMUS( | Adra1b | 24 | 27 | 19 | 39 | 36 | 39 | 0.603934 | 0.007719 | 0.038699 | down |
| ENSMUS( | Lmod1 | 13 | 6 | 18 | 27 | 29 | 29 | 0.439147 | 0.008278 | 0.041053 | down |
| ENSMUS( | Tmie | 43 | 37 | 38 | 64 | 43 | 73 | 0.650348 | 0.008626 | 0.042478 | down |
| ENSMUS( | Pctp | 8 | 12 | 12 | 25 | 15 | 23 | 0.505301 | 0.008871 | 0.043519 | down |
| ENSMUS( | Ncf4 | 20 | 19 | 17 | 28 | 38 | 29 | 0.581616 | 0.009124 | 0.044573 | down |
| ENSMUS( | Gm10638 | 5 | 8 | 10 | 19 | 15 | 16 | 0.459919 | 0.009218 | 0.044968 | down |
| ENSMUS( | Sowahb | 40 | 33 | 32 | 44 | 56 | 56 | 0.666064 | 0.009727 | 0.04709 | down |
| ENSMUS( | Eva1b | 14 | 23 | 17 | 26 | 33 | 33 | 0.581049 | 0.0098 | 0.047407 | down |
| ENSMUS( | Derl3 | 16 | 19 | 20 | 30 | 30 | 33 | 0.588324 | 0.010276 | 0.049344 | down |
| ENSMUS( | Gch1 | 5 | 5 | 6 | 17 | 20 | 5 | 0.374959 | 0.010989 | 0.052274 | down |
| ENSMUS( | Gm7347 | 0 | 1 | 0 | 5 | 2 | 5 | 0.079316 | 0.011219 | 0.053137 | down |
| ENSMUS( | Hs3st3a1 | 22 | 32 | 20 | 59 | 36 | 30 | 0.577864 | 0.011398 | 0.05382 | down |
| ENSMUS( | Zbtb7c | 23 | 28 | 25 | 41 | 44 | 34 | 0.630604 | 0.011427 | 0.053912 | down |
| ENSMUS( | Cox16 | 16 | 14 | 8 | 21 | 22 | 27 | 0.531874 | 0.011548 | 0.054417 | down |
| ENSMUS( | Ttc9b | 302 | 385 | 451 | 837 | 516 | 594 | 0.581008 | 0.011551 | 0.054421 | down |
| ENSMUS( | C3ar1 | 20 | 16 | 22 | 29 | 32 | 35 | 0.60284 | 0.011911 | 0.055866 | down |
| ENSMUS( | Tmem114 | 31 | 38 | 25 | 55 | 42 | 44 | 0.653714 | 0.011952 | 0.05602 | down |
| ENSMUS( | Kctd14 | 71 | 49 | 49 | 85 | 102 | 68 | 0.651511 | 0.012152 | 0.056779 | down |
| ENSMUS( | Cst6 | 54 | 74 | 36 | 78 | 73 | 94 | 0.65584 | 0.013001 | 0.059998 | down |
| ENSMUS( | Lefty2 | 14 | 13 | 15 | 34 | 20 | 23 | 0.53971 | 0.013116 | 0.060402 | down |
| ENSMUS( | Car13 | 44 | 36 | 30 | 49 | 68 | 47 | 0.658991 | 0.013603 | 0.062028 | down |
| ENSMUS( | Nkx2-1 | 5 | 10 | 6 | 10 | 14 | 23 | 0.443966 | 0.014194 | 0.064343 | down |
| ENSMUS( | Chac1 | 49 | 73 | 40 | 71 | 92 | 76 | 0.664473 | 0.014257 | 0.064577 | down |
| ENSMUS( | Dsc3 | 42 | 23 | 41 | 71 | 76 | 47 | 0.542184 | 0.014453 | 0.065255 | down |
| ENSMUS( | Wif1 | 20 | 15 | 13 | 34 | 23 | 25 | 0.57418 | 0.014476 | 0.065326 | down |

| | | | | | | | | | | | |
|---|---|---|---|---|---|---|---|---|---|---|---|
| ENSMUS( | Fancc | 57 | 61 | 32 | 75 | 78 | 69 | 0.659334 | 0.014739 | 0.066301 | down |
| ENSMUS( | Tspan8 | 9 | 7 | 5 | 15 | 12 | 18 | 0.458717 | 0.015452 | 0.068888 | down |
| ENSMUS( | Slc22a2 | 15 | 17 | 14 | 20 | 29 | 30 | 0.57685 | 0.015584 | 0.069368 | down |
| ENSMUS( | Ip6k3 | 4 | 3 | 6 | 8 | 12 | 13 | 0.397722 | 0.015814 | 0.070223 | down |
| ENSMUS( | Styxl1 | 12 | 9 | 3 | 24 | 14 | 18 | 0.413616 | 0.016176 | 0.07158 | down |
| ENSMUS( | Prph | 45 | 51 | 25 | 64 | 59 | 62 | 0.638281 | 0.016287 | 0.071967 | down |
| ENSMUS( | Prrx2 | 14 | 9 | 16 | 23 | 24 | 23 | 0.556768 | 0.017227 | 0.07545 | down |
| ENSMUS( | Ddt | 11 | 19 | 15 | 31 | 20 | 26 | 0.577782 | 0.017557 | 0.076502 | down |
| ENSMUS( | Wnt1 | 0 | 1 | 0 | 3 | 5 | 3 | 0.086223 | 0.017859 | 0.077688 | down |
| ENSMUS( | Cebpb | 23 | 30 | 26 | 48 | 38 | 34 | 0.649203 | 0.018279 | 0.079182 | down |
| ENSMUS( | Tpsb2 | 9 | 6 | 8 | 20 | 14 | 13 | 0.483547 | 0.018708 | 0.080668 | down |
| ENSMUS( | Gipc3 | 29 | 38 | 18 | 54 | 39 | 43 | 0.609033 | 0.019148 | 0.082251 | down |
| ENSMUS( | Cox4i2 | 7 | 9 | 5 | 6 | 24 | 18 | 0.431118 | 0.019521 | 0.083583 | down |
| ENSMUS( | Enpp3 | 10 | 7 | 14 | 14 | 21 | 26 | 0.513418 | 0.019596 | 0.083853 | down |
| ENSMUS( | Asgr1 | 36 | 45 | 23 | 55 | 43 | 60 | 0.644601 | 0.019951 | 0.085203 | down |
| ENSMUS( | Adamts13 | 16 | 15 | 17 | 22 | 25 | 33 | 0.598504 | 0.020564 | 0.087358 | down |
| ENSMUS( | Birc7 | 5 | 6 | 6 | 15 | 13 | 10 | 0.442048 | 0.020569 | 0.087367 | down |
| ENSMUS( | Gm765 | 1 | 0 | 1 | 4 | 7 | 2 | 0.154337 | 0.021131 | 0.089336 | down |
| ENSMUS( | Acr | 17 | 13 | 9 | 19 | 29 | 20 | 0.56126 | 0.021782 | 0.091606 | down |
| ENSMUS( | Chat | 2 | 0 | 1 | 4 | 4 | 7 | 0.199967 | 0.022394 | 0.093869 | down |
| ENSMUS( | Chrnb4 | 12 | 15 | 15 | 34 | 32 | 11 | 0.535838 | 0.022588 | 0.094557 | down |
| ENSMUS( | Cartpt | 23 | 20 | 27 | 53 | 31 | 29 | 0.613474 | 0.023072 | 0.096183 | down |
| ENSMUS( | Chst5 | 20 | 13 | 18 | 25 | 31 | 27 | 0.610365 | 0.023593 | 0.098004 | down |
| ENSMUS( | Cma1 | 5 | 4 | 7 | 13 | 8 | 15 | 0.446868 | 0.023657 | 0.098167 | down |
| ENSMUS( | Nhlh2 | 40 | 24 | 30 | 47 | 44 | 50 | 0.660205 | 0.023826 | 0.098681 | down |
| ENSMUS( | Nthl1 | 42 | 37 | 19 | 49 | 59 | 45 | 0.623554 | 0.023919 | 0.099008 | down |
| ENSMUS( | Unc5cl | 4 | 5 | 5 | 10 | 10 | 13 | 0.422609 | 0.024048 | 0.099463 | down |
| ENSMUS( | Cited1 | 7 | 8 | 11 | 16 | 18 | 16 | 0.52001 | 0.0243 | 0.100304 | down |
| ENSMUS( | H2-Q2 | 3 | 1 | 3 | 8 | 4 | 10 | 0.320352 | 0.024372 | 0.100542 | down |
| ENSMUS( | Dmrta1 | 0 | 3 | 2 | 6 | 9 | 5 | 0.248235 | 0.024441 | 0.100722 | down |
| ENSMUS( | Wnt8b | 4 | 3 | 7 | 11 | 7 | 15 | 0.430519 | 0.024449 | 0.100742 | down |
| ENSMUS( | Cited4 | 13 | 16 | 5 | 20 | 19 | 28 | 0.494071 | 0.025232 | 0.103499 | down |
| ENSMUS( | Zfp580 | 141 | 200 | 191 | 346 | 235 | 217 | 0.658821 | 0.026215 | 0.106688 | down |
| ENSMUS( | Adm | 20 | 16 | 10 | 22 | 36 | 21 | 0.568215 | 0.026344 | 0.107058 | down |

**Dataset S2. List of DE genes between SI and control (SI/C) female offspring**

| Gene | GeneNam | SI-1_coun | SI-2_coun | SI-3_coun | C-1_count | C-2_count | C-3_count | FoldChang | p | q | Up/Down |
|---|---|---|---|---|---|---|---|---|---|---|---|
| ENSMUS( | F5 | 1239 | 1258 | 1116 | 109 | 124 | 119 | 9.696382 | 0 | 0 | up |
| ENSMUS( | Enpp2 | 44784 | 43071 | 38222 | 11044 | 10994 | 11708 | 3.525476 | 0 | 0 | up |
| ENSMUS( | Lcn2 | 1047 | 916 | 826 | 95 | 82 | 97 | 9.589203 | 1.13E-278 | 7.94E-275 | up |
| ENSMUS( | Kl | 2902 | 2832 | 2496 | 779 | 727 | 814 | 3.347744 | 2.27E-252 | 1.07E-248 | up |
| ENSMUS( | Trpm3 | 1508 | 1529 | 1416 | 290 | 289 | 306 | 4.758556 | 2.14E-251 | 8.62E-248 | up |
| ENSMUS( | Ace | 1428 | 1568 | 1381 | 312 | 324 | 322 | 4.320038 | 6.42E-175 | 1.81E-171 | up |
| ENSMUS( | Clic6 | 1372 | 1357 | 1274 | 417 | 405 | 416 | 3.055679 | 5.39E-152 | 1.27E-148 | up |
| ENSMUS( | Tmem72 | 924 | 810 | 881 | 162 | 164 | 167 | 5.022837 | 7.78E-138 | 1.57E-134 | up |
| ENSMUS( | Mfrp | 347 | 366 | 290 | 23 | 32 | 25 | 11.80902 | 5.11E-134 | 9.60E-131 | up |
| ENSMUS( | Cldn2 | 558 | 528 | 468 | 87 | 95 | 94 | 5.31112 | 1.51E-132 | 2.65E-129 | up |
| ENSMUS( | Prlr | 919 | 880 | 830 | 232 | 255 | 285 | 3.223763 | 2.84E-123 | 4.45E-120 | up |
| ENSMUS( | Snrnp70 | 3448 | 3399 | 3124 | 1536 | 1493 | 1505 | 2.07641 | 1.14E-116 | 1.69E-113 | up |
| ENSMUS( | Car12 | 2224 | 2034 | 1902 | 837 | 863 | 870 | 2.262008 | 1.73E-115 | 2.43E-112 | up |
| ENSMUS( | Atp2b3 | 1611 | 1557 | 1474 | 590 | 609 | 613 | 2.421251 | 1.40E-113 | 1.88E-110 | up |
| ENSMUS( | Ttr | 120860 | 111113 | 95775 | 30271 | 30545 | 33592 | 3.272018 | 4.27E-105 | 5.23E-102 | up |
| ENSMUS( | 1500015O | 823 | 832 | 721 | 239 | 215 | 263 | 3.130376 | 2.48E-103 | 2.79E-100 | up |
| ENSMUS( | Col8a1 | 625 | 496 | 509 | 100 | 87 | 109 | 5.199989 | 5.51E-103 | 5.96E-100 | up |
| ENSMUS( | Nktr | 702 | 772 | 677 | 183 | 204 | 190 | 3.524172 | 5.61E-96 | 5.45E-93 | up |
| ENSMUS( | Pnisr | 3758 | 3644 | 3195 | 1762 | 1733 | 1755 | 1.902986 | 8.08E-96 | 7.58E-93 | up |
| ENSMUS( | Lgals3bp | 967 | 906 | 790 | 308 | 304 | 346 | 2.62234 | 1.26E-92 | 1.14E-89 | up |
| ENSMUS( | Ttc14 | 3212 | 3039 | 2905 | 1445 | 1483 | 1455 | 1.973318 | 1.57E-92 | 1.38E-89 | up |
| ENSMUS( | Snhg11 | 13090 | 13347 | 11932 | 6018 | 6357 | 6623 | 1.909042 | 4.85E-89 | 4.14E-86 | up |
| ENSMUS( | Igf2 | 5209 | 4869 | 4337 | 2474 | 2301 | 2343 | 1.907739 | 4.52E-85 | 3.54E-82 | up |
| ENSMUS( | Sulf1 | 1097 | 1074 | 963 | 258 | 264 | 348 | 3.411661 | 7.33E-85 | 5.58E-82 | up |
| ENSMUS( | Trank1 | 3366 | 3370 | 3048 | 1623 | 1687 | 1732 | 1.833549 | 3.71E-83 | 2.68E-80 | up |
| ENSMUS( | Lmbrd2 | 431 | 444 | 409 | 102 | 109 | 115 | 3.727892 | 2.32E-81 | 1.52E-78 | up |
| ENSMUS( | Lbp | 1041 | 984 | 853 | 248 | 318 | 309 | 3.104552 | 4.57E-81 | 2.92E-78 | up |
| ENSMUS( | Folr1 | 848 | 728 | 668 | 229 | 243 | 258 | 2.898238 | 5.69E-81 | 3.56E-78 | up |
| ENSMUS( | Gabrb2 | 1256 | 1272 | 1315 | 342 | 402 | 378 | 3.24992 | 1.43E-80 | 8.59E-78 | up |
| ENSMUS( | Atp11b | 1883 | 1816 | 1666 | 814 | 889 | 873 | 1.967056 | 6.27E-78 | 3.53E-75 | up |
| ENSMUS( | Cldn1 | 421 | 398 | 388 | 100 | 104 | 102 | 3.728545 | 5.32E-77 | 2.94E-74 | up |
| ENSMUS( | A2m | 579 | 560 | 501 | 168 | 172 | 194 | 2.901925 | 1.56E-74 | 8.15E-72 | up |
| ENSMUS( | Nr2c2 | 557 | 564 | 521 | 179 | 180 | 200 | 2.779604 | 6.18E-70 | 2.95E-67 | up |
| ENSMUS( | Prepl | 5142 | 4781 | 4502 | 2614 | 2745 | 2864 | 1.6574 | 1.09E-67 | 5.01E-65 | up |
| ENSMUS( | Slc4a5 | 537 | 543 | 422 | 61 | 90 | 87 | 5.954071 | 1.09E-65 | 4.58E-63 | up |
| ENSMUS( | Zbtb16 | 461 | 466 | 452 | 137 | 143 | 165 | 2.938885 | 4.38E-61 | 1.51E-58 | up |
| ENSMUS( | Usp33 | 1058 | 965 | 915 | 399 | 420 | 486 | 2.129441 | 7.27E-60 | 2.32E-57 | up |
| ENSMUS( | Rnpc3 | 667 | 648 | 574 | 242 | 271 | 249 | 2.338115 | 6.19E-59 | 1.87E-56 | up |
| ENSMUS( | Sorbs1 | 788 | 812 | 724 | 320 | 336 | 359 | 2.165124 | 1.57E-58 | 4.69E-56 | up |
| ENSMUS( | Ints6l | 1364 | 1325 | 1228 | 663 | 636 | 667 | 1.8817 | 1.86E-58 | 5.46E-56 | up |
| ENSMUS( | Hook1 | 1731 | 1685 | 1475 | 785 | 884 | 902 | 1.7961 | 6.30E-58 | 1.81E-55 | up |
| ENSMUS( | Cgnl1 | 562 | 512 | 458 | 153 | 157 | 129 | 3.281002 | 1.29E-57 | 3.63E-55 | up |
| ENSMUS( | Col9a3 | 810 | 742 | 724 | 238 | 273 | 315 | 2.610669 | 3.69E-57 | 1.03E-54 | up |
| ENSMUS( | Rsrp1 | 6348 | 6300 | 5552 | 3608 | 3673 | 3803 | 1.54995 | 1.77E-55 | 4.61E-53 | up |
| ENSMUS( | Kcne2 | 432 | 403 | 365 | 103 | 102 | 134 | 3.34887 | 1.37E-54 | 3.51E-52 | up |
| ENSMUS( | Alg10b | 549 | 525 | 493 | 188 | 198 | 223 | 2.434661 | 2.46E-54 | 6.24E-52 | up |
| ENSMUS( | Slc2a12 | 643 | 607 | 595 | 151 | 180 | 221 | 3.172846 | 1.08E-53 | 2.65E-51 | up |
| ENSMUS( | Steap2 | 564 | 490 | 450 | 178 | 188 | 212 | 2.455735 | 1.42E-53 | 3.46E-51 | up |
| ENSMUS( | Sostdc1 | 679 | 558 | 491 | 178 | 183 | 209 | 2.854048 | 1.80E-53 | 4.32E-51 | up |
| ENSMUS( | Gm6356 | 113 | 117 | 106 | 5 | 6 | 7 | 17.70299 | 4.54E-53 | 1.08E-50 | up |
| ENSMUS( | Zfp445 | 2869 | 2910 | 2618 | 1565 | 1596 | 1725 | 1.624775 | 1.28E-52 | 3.01E-50 | up |
| ENSMUS( | Otx2 | 422 | 379 | 373 | 136 | 124 | 150 | 2.708003 | 2.48E-50 | 5.45E-48 | up |
| ENSMUS( | Aqp1 | 451 | 399 | 386 | 59 | 94 | 99 | 4.65051 | 2.66E-50 | 5.82E-48 | up |
| ENSMUS( | Lnpep | 285 | 309 | 301 | 69 | 83 | 74 | 3.75466 | 2.35E-48 | 4.74E-46 | up |
| ENSMUS( | Zfp871 | 296 | 331 | 351 | 72 | 72 | 81 | 4.138359 | 2.89E-48 | 5.73E-46 | up |
| ENSMUS( | Sgms2 | 260 | 279 | 224 | 71 | 59 | 70 | 3.596147 | 3.46E-48 | 6.82E-46 | up |
| ENSMUS( | Usp34 | 2033 | 2030 | 1896 | 1112 | 1074 | 1132 | 1.697748 | 8.44E-48 | 1.65E-45 | up |
| ENSMUS( | Elfn2 | 1041 | 1022 | 917 | 495 | 470 | 455 | 1.978527 | 3.54E-47 | 6.69E-45 | up |
| ENSMUS( | Slc12a2 | 2463 | 2430 | 2199 | 1357 | 1379 | 1451 | 1.600137 | 1.48E-46 | 2.72E-44 | up |
| ENSMUS( | Adamts1 | 522 | 571 | 493 | 213 | 203 | 223 | 2.346229 | 6.54E-46 | 1.16E-43 | up |
| ENSMUS( | Jchain | 547 | 552 | 490 | 203 | 244 | 232 | 2.211391 | 9.45E-46 | 1.66E-43 | up |
| ENSMUS( | Leng8 | 1900 | 1788 | 1741 | 943 | 1023 | 998 | 1.731758 | 1.41E-45 | 2.43E-43 | up |
| ENSMUS( | Retsat | 935 | 860 | 863 | 349 | 313 | 393 | 2.385961 | 3.39E-45 | 5.71E-43 | up |
| ENSMUS( | Atp11c | 829 | 774 | 735 | 377 | 343 | 344 | 2.072812 | 4.04E-44 | 6.50E-42 | up |
| ENSMUS( | Zkscan16 | 394 | 387 | 399 | 76 | 84 | 109 | 4.173679 | 2.34E-43 | 3.66E-41 | up |
| ENSMUS( | Slc31a1 | 1113 | 1069 | 992 | 561 | 563 | 570 | 1.769276 | 2.87E-43 | 4.47E-41 | up |
| ENSMUS( | Strip2 | 1237 | 1297 | 1158 | 642 | 636 | 663 | 1.797779 | 4.50E-43 | 6.96E-41 | up |
| ENSMUS( | Plin4 | 410 | 379 | 355 | 141 | 159 | 139 | 2.457874 | 2.48E-42 | 3.72E-40 | up |
| ENSMUS( | Cab39l | 1704 | 1730 | 1438 | 925 | 938 | 896 | 1.663246 | 5.17E-42 | 7.58E-40 | up |
| ENSMUS( | Fos | 496 | 460 | 444 | 204 | 195 | 195 | 2.225048 | 5.32E-42 | 7.76E-40 | up |
| ENSMUS( | Sema3b | 295 | 285 | 258 | 79 | 98 | 90 | 2.964437 | 6.93E-42 | 1.01E-39 | up |
| ENSMUS( | Hipk2 | 258 | 299 | 314 | 65 | 64 | 78 | 4.011185 | 1.05E-41 | 1.51E-39 | up |
| ENSMUS( | Klf9 | 2766 | 2628 | 2493 | 1513 | 1668 | 1699 | 1.528251 | 3.14E-40 | 4.34E-38 | up |
| ENSMUS( | Abca4 | 358 | 347 | 335 | 88 | 57 | 94 | 4.12107 | 1.03E-39 | 1.38E-37 | up |
| ENSMUS( | Ndst1 | 1275 | 1179 | 1119 | 640 | 655 | 711 | 1.68343 | 1.49E-39 | 1.99E-37 | up |
| ENSMUS( | Npas4 | 292 | 290 | 234 | 70 | 49 | 54 | 4.4278 | 1.59E-39 | 2.11E-37 | up |
| ENSMUS( | Adcy9 | 606 | 520 | 531 | 231 | 247 | 234 | 2.197952 | 4.04E-39 | 5.26E-37 | up |
| ENSMUS( | Timp3 | 1322 | 1296 | 1297 | 577 | 556 | 664 | 2.065988 | 4.50E-39 | 5.83E-37 | up |

| | | | | | | | | | | | |
|---|---|---|---|---|---|---|---|---|---|---|---|
| ENSMUS( | Myef2 | 1265 | 1246 | 1181 | 658 | 709 | 717 | 1.675891 | 4.67E-39 | 6.03E-37 | up |
| ENSMUS( | Htr2c | 1645 | 1547 | 1574 | 778 | 846 | 843 | 1.829349 | 1.45E-38 | 1.84E-36 | up |
| ENSMUS( | Pik3c2a | 592 | 601 | 519 | 265 | 255 | 297 | 1.979576 | 2.81E-38 | 3.49E-36 | up |
| ENSMUS( | Prpf39 | 1046 | 1018 | 899 | 495 | 561 | 572 | 1.718853 | 6.07E-38 | 7.44E-36 | up |
| ENSMUS( | Col8a2 | 327 | 341 | 318 | 82 | 112 | 90 | 3.284716 | 1.33E-37 | 1.63E-35 | up |
| ENSMUS( | Slco1a4 | 1069 | 1103 | 950 | 544 | 563 | 632 | 1.696854 | 1.73E-37 | 2.06E-35 | up |
| ENSMUS( | Vps13a | 871 | 893 | 827 | 445 | 463 | 460 | 1.790976 | 2.10E-37 | 2.50E-35 | up |
| ENSMUS( | Ahnak | 717 | 668 | 638 | 309 | 322 | 377 | 1.899609 | 4.01E-37 | 4.75E-35 | up |
| ENSMUS( | Slc13a4 | 805 | 740 | 668 | 372 | 395 | 383 | 1.814121 | 5.57E-37 | 6.53E-35 | up |
| ENSMUS( | Rbm5 | 2503 | 2481 | 2296 | 1428 | 1567 | 1564 | 1.509768 | 5.68E-37 | 6.64E-35 | up |
| ENSMUS( | 4932438A | 2093 | 2039 | 1801 | 1133 | 1240 | 1271 | 1.53728 | 7.50E-37 | 8.73E-35 | up |
| ENSMUS( | Gm10031 | 327 | 315 | 264 | 95 | 115 | 124 | 2.560909 | 1.54E-36 | 1.77E-34 | up |
| ENSMUS( | Ryr3 | 647 | 686 | 514 | 257 | 279 | 255 | 2.195372 | 2.82E-36 | 3.17E-34 | up |
| ENSMUS( | Cd74 | 512 | 499 | 463 | 233 | 199 | 220 | 2.13423 | 2.41E-35 | 2.57E-33 | up |
| ENSMUS( | Fam120c | 588 | 554 | 565 | 255 | 264 | 255 | 2.08681 | 3.19E-35 | 3.37E-33 | up |
| ENSMUS( | Dhx9 | 1986 | 1765 | 1548 | 932 | 924 | 877 | 1.823413 | 1.37E-34 | 1.41E-32 | up |
| ENSMUS( | Pkd1 | 958 | 1011 | 860 | 423 | 506 | 513 | 1.854688 | 1.44E-34 | 1.47E-32 | up |
| ENSMUS( | Trpm7 | 1422 | 1350 | 1245 | 789 | 802 | 795 | 1.5889 | 1.46E-34 | 1.49E-32 | up |
| ENSMUS( | Eml6 | 326 | 306 | 281 | 96 | 123 | 122 | 2.531465 | 1.85E-34 | 1.88E-32 | up |
| ENSMUS( | Stx3 | 897 | 805 | 797 | 402 | 449 | 482 | 1.774128 | 3.04E-34 | 3.05E-32 | up |
| ENSMUS( | Ranbp2 | 2981 | 2835 | 2757 | 1672 | 1686 | 1786 | 1.576471 | 4.09E-34 | 4.02E-32 | up |
| ENSMUS( | Smg1 | 2041 | 2064 | 1897 | 1205 | 1206 | 1281 | 1.537159 | 5.40E-34 | 5.28E-32 | up |
| ENSMUS( | Vat1l | 1224 | 1276 | 1205 | 612 | 656 | 697 | 1.786503 | 1.33E-33 | 1.28E-31 | up |
| ENSMUS( | Cp | 780 | 796 | 750 | 420 | 396 | 402 | 1.805243 | 1.44E-33 | 1.38E-31 | up |
| ENSMUS( | Cep290 | 307 | 288 | 294 | 116 | 114 | 112 | 2.458704 | 1.76E-33 | 1.67E-31 | up |
| ENSMUS( | Xkr4 | 267 | 305 | 298 | 78 | 92 | 99 | 3.076234 | 3.38E-33 | 3.14E-31 | up |
| ENSMUS( | Klhl34 | 596 | 549 | 569 | 265 | 262 | 257 | 2.068081 | 3.68E-33 | 3.39E-31 | up |
| ENSMUS( | Flnb | 695 | 699 | 571 | 340 | 351 | 343 | 1.789574 | 7.93E-33 | 7.15E-31 | up |
| ENSMUS( | Elovl7 | 692 | 686 | 612 | 317 | 355 | 380 | 1.788531 | 1.13E-32 | 1.00E-30 | up |
| ENSMUS( | Nrp1 | 756 | 724 | 675 | 376 | 374 | 411 | 1.754482 | 1.15E-32 | 1.01E-30 | up |
| ENSMUS( | Sfrp1 | 466 | 499 | 415 | 212 | 216 | 231 | 1.977306 | 3.60E-32 | 3.04E-30 | up |
| ENSMUS( | Phf20l1 | 1571 | 1626 | 1403 | 917 | 962 | 956 | 1.531681 | 4.33E-32 | 3.65E-30 | up |
| ENSMUS( | Ly6c1 | 1158 | 1089 | 900 | 589 | 569 | 571 | 1.71165 | 4.68E-32 | 3.92E-30 | up |
| ENSMUS( | Ankrd52 | 454 | 487 | 412 | 201 | 232 | 213 | 1.977328 | 1.18E-31 | 9.78E-30 | up |
| ENSMUS( | Ly6a | 704 | 574 | 543 | 263 | 267 | 311 | 2.042459 | 5.82E-31 | 4.67E-29 | up |
| ENSMUS( | Nvl | 1136 | 1182 | 1075 | 632 | 630 | 683 | 1.650293 | 6.61E-31 | 5.26E-29 | up |
| ENSMUS( | Kcnma1 | 491 | 496 | 464 | 239 | 240 | 244 | 1.897594 | 3.55E-30 | 2.71E-28 | up |
| ENSMUS( | Ncam2 | 516 | 561 | 543 | 229 | 242 | 251 | 2.128333 | 7.87E-30 | 5.89E-28 | up |
| ENSMUS( | Paxbp1 | 801 | 767 | 687 | 427 | 411 | 396 | 1.722015 | 8.14E-30 | 6.08E-28 | up |
| ENSMUS( | Car14 | 402 | 332 | 306 | 143 | 119 | 148 | 2.38877 | 2.38E-29 | 1.75E-27 | up |
| ENSMUS( | Tmem245 | 1310 | 1251 | 1145 | 772 | 714 | 787 | 1.539173 | 3.62E-29 | 2.63E-27 | up |
| ENSMUS( | Oca2 | 163 | 176 | 140 | 46 | 42 | 46 | 3.369039 | 8.10E-29 | 5.77E-27 | up |
| ENSMUS( | Klhl3 | 231 | 216 | 175 | 61 | 80 | 72 | 2.748659 | 1.24E-28 | 8.76E-27 | up |
| ENSMUS( | Sult1c2 | 78 | 69 | 61 | 7 | 4 | 8 | 10.33481 | 1.31E-28 | 9.20E-27 | up |
| ENSMUS( | Rreb1 | 978 | 905 | 881 | 527 | 449 | 493 | 1.776586 | 1.43E-28 | 1.00E-26 | up |
| ENSMUS( | Ccnl1 | 1229 | 1132 | 1073 | 699 | 693 | 703 | 1.547249 | 1.57E-28 | 1.10E-26 | up |
| ENSMUS( | Atp7a | 115 | 166 | 143 | 22 | 27 | 27 | 5.307213 | 1.63E-28 | 1.14E-26 | up |
| ENSMUS( | Pcolce2 | 287 | 269 | 242 | 111 | 101 | 107 | 2.357938 | 2.21E-28 | 1.54E-26 | up |
| ENSMUS( | Guf1 | 866 | 842 | 755 | 445 | 493 | 490 | 1.628716 | 3.59E-28 | 2.47E-26 | up |
| ENSMUS( | Cmip | 1297 | 1351 | 1153 | 775 | 747 | 840 | 1.519862 | 4.90E-28 | 3.35E-26 | up |
| ENSMUS( | Zbtb37 | 185 | 186 | 145 | 44 | 57 | 57 | 3.078874 | 8.24E-28 | 5.55E-26 | up |
| ENSMUS( | Flt1 | 570 | 500 | 487 | 234 | 282 | 256 | 1.904271 | 1.03E-27 | 6.87E-26 | up |
| ENSMUS( | Supt16 | 825 | 777 | 717 | 416 | 473 | 447 | 1.638743 | 1.13E-27 | 7.54E-26 | up |
| ENSMUS( | Nhsl2 | 1042 | 1052 | 861 | 546 | 616 | 595 | 1.585098 | 1.23E-27 | 8.10E-26 | up |
| ENSMUS( | Lepr | 239 | 263 | 222 | 101 | 86 | 85 | 2.50876 | 1.52E-27 | 9.94E-26 | up |
| ENSMUS( | Mdm4 | 1379 | 1392 | 1223 | 841 | 819 | 850 | 1.501799 | 1.91E-27 | 1.23E-25 | up |
| ENSMUS( | Gse1 | 365 | 371 | 350 | 149 | 175 | 185 | 2.022108 | 2.26E-27 | 1.44E-25 | up |
| ENSMUS( | Npr3 | 705 | 692 | 609 | 374 | 379 | 375 | 1.677227 | 3.48E-27 | 2.18E-25 | up |
| ENSMUS( | Mga | 1245 | 1253 | 1119 | 743 | 768 | 738 | 1.517952 | 3.52E-27 | 2.20E-25 | up |
| ENSMUS( | Baiap2l1 | 170 | 194 | 188 | 52 | 60 | 46 | 3.308236 | 6.82E-27 | 4.20E-25 | up |
| ENSMUS( | Sacm1l | 647 | 663 | 644 | 279 | 268 | 337 | 2.097532 | 1.59E-26 | 9.60E-25 | up |
| ENSMUS( | H2-D1 | 1244 | 1184 | 1096 | 686 | 758 | 753 | 1.515303 | 1.65E-26 | 9.96E-25 | up |
| ENSMUS( | Igfbp2 | 1769 | 1541 | 1440 | 987 | 933 | 963 | 1.552363 | 1.98E-26 | 1.19E-24 | up |
| ENSMUS( | Emb | 939 | 879 | 767 | 485 | 502 | 554 | 1.582623 | 2.57E-26 | 1.53E-24 | up |
| ENSMUS( | Tenm1 | 473 | 440 | 442 | 204 | 178 | 235 | 2.081086 | 3.10E-26 | 1.84E-24 | up |
| ENSMUS( | Lyst | 1444 | 1481 | 1349 | 836 | 903 | 951 | 1.503337 | 3.19E-26 | 1.90E-24 | up |
| ENSMUS( | Frem1 | 203 | 217 | 215 | 63 | 81 | 72 | 2.78941 | 3.72E-26 | 2.19E-24 | up |
| ENSMUS( | Trpv4 | 172 | 181 | 182 | 54 | 52 | 62 | 3.024576 | 3.84E-26 | 2.25E-24 | up |
| ENSMUS( | Pcdhgc5 | 634 | 639 | 555 | 230 | 318 | 306 | 2.024227 | 4.77E-26 | 2.78E-24 | up |
| ENSMUS( | Wdr48 | 904 | 929 | 799 | 493 | 553 | 512 | 1.594004 | 6.29E-26 | 3.64E-24 | up |
| ENSMUS( | Fam13b | 549 | 541 | 520 | 290 | 298 | 270 | 1.772677 | 8.64E-26 | 4.94E-24 | up |
| ENSMUS( | Atm | 257 | 260 | 282 | 88 | 86 | 107 | 2.706349 | 1.15E-25 | 6.45E-24 | up |
| ENSMUS( | Kcna1 | 419 | 509 | 480 | 146 | 177 | 148 | 2.835918 | 1.24E-25 | 6.93E-24 | up |
| ENSMUS( | Kif3a | 1757 | 1686 | 1646 | 1035 | 1017 | 1091 | 1.531892 | 1.27E-25 | 7.11E-24 | up |
| ENSMUS( | Prpf4b | 2215 | 2255 | 2099 | 1351 | 1368 | 1421 | 1.500822 | 1.37E-25 | 7.62E-24 | up |
| ENSMUS( | Btg2 | 590 | 564 | 561 | 311 | 305 | 308 | 1.755504 | 2.02E-25 | 1.12E-23 | up |
| ENSMUS( | Zcchc7 | 1276 | 1208 | 1001 | 677 | 683 | 740 | 1.56308 | 3.18E-25 | 1.74E-23 | up |
| ENSMUS( | Fam208b | 443 | 499 | 419 | 205 | 213 | 239 | 1.960009 | 3.29E-25 | 1.79E-23 | up |
| ENSMUS( | Drc7 | 290 | 317 | 257 | 134 | 120 | 129 | 2.126826 | 3.88E-25 | 2.09E-23 | up |
| ENSMUS( | Ahctf1 | 1109 | 1075 | 1054 | 601 | 630 | 680 | 1.604759 | 4.28E-25 | 2.30E-23 | up |

| | | | | | | | | | | |
|---|---|---|---|---|---|---|---|---|---|---|
| ENSMUS( | Ankrd12 | 1292 | 1229 | 1194 | 745 | 746 | 825 | 1.517981 | 5.40E-25 | 2.86E-23 up |
| ENSMUS( | Tmprss11a | 177 | 206 | 176 | 37 | 29 | 53 | 4.465363 | 5.54E-25 | 2.93E-23 up |
| ENSMUS( | Golgb1 | 1511 | 1390 | 1357 | 788 | 858 | 953 | 1.550887 | 6.16E-25 | 3.25E-23 up |
| ENSMUS( | Sh3d19 | 565 | 543 | 502 | 278 | 308 | 299 | 1.718452 | 6.82E-25 | 3.58E-23 up |
| ENSMUS( | Faxc | 350 | 408 | 329 | 124 | 152 | 163 | 2.344025 | 1.33E-24 | 6.85E-23 up |
| ENSMUS( | Zfp26 | 421 | 453 | 405 | 202 | 229 | 226 | 1.842124 | 1.52E-24 | 7.80E-23 up |
| ENSMUS( | Pcm1 | 1665 | 1705 | 1577 | 1037 | 1020 | 1052 | 1.504396 | 1.82E-24 | 9.23E-23 up |
| ENSMUS( | Eml5 | 1082 | 1111 | 935 | 652 | 618 | 688 | 1.507375 | 2.36E-24 | 1.19E-22 up |
| ENSMUS( | Zfp292 | 1036 | 985 | 909 | 606 | 583 | 629 | 1.521857 | 2.83E-24 | 1.42E-22 up |
| ENSMUS( | Mtmr9 | 512 | 511 | 509 | 249 | 241 | 289 | 1.865556 | 3.67E-24 | 1.83E-22 up |
| ENSMUS( | Arhgef11 | 754 | 714 | 659 | 395 | 425 | 433 | 1.603438 | 3.84E-24 | 1.91E-22 up |
| ENSMUS( | Dgkg | 1322 | 1478 | 1333 | 725 | 825 | 819 | 1.652699 | 7.08E-24 | 3.46E-22 up |
| ENSMUS( | Ice1 | 1323 | 1319 | 1177 | 720 | 801 | 872 | 1.509326 | 7.99E-24 | 3.88E-22 up |
| ENSMUS( | Apold1 | 144 | 147 | 143 | 43 | 37 | 53 | 3.098191 | 1.20E-23 | 5.78E-22 up |
| ENSMUS( | Egr2 | 146 | 127 | 111 | 36 | 30 | 24 | 3.99569 | 1.96E-23 | 9.35E-22 up |
| ENSMUS( | Akap9 | 1046 | 1020 | 981 | 623 | 609 | 661 | 1.522861 | 2.06E-23 | 9.81E-22 up |
| ENSMUS( | Focad | 569 | 564 | 528 | 295 | 312 | 331 | 1.675441 | 2.13E-23 | 1.01E-21 up |
| ENSMUS( | Zcchc6 | 746 | 681 | 599 | 366 | 311 | 397 | 1.77893 | 2.58E-23 | 1.22E-21 up |
| ENSMUS( | Klhl24 | 1456 | 1404 | 1307 | 907 | 865 | 848 | 1.50076 | 2.62E-23 | 1.24E-21 up |
| ENSMUS( | Ubxn2a | 892 | 840 | 742 | 500 | 488 | 529 | 1.538102 | 6.03E-23 | 2.77E-21 up |
| ENSMUS( | Slc37a2 | 104 | 122 | 89 | 25 | 20 | 31 | 3.915658 | 6.43E-23 | 2.95E-21 up |
| ENSMUS( | Wdr86 | 166 | 173 | 144 | 52 | 54 | 58 | 2.780138 | 7.46E-23 | 3.41E-21 up |
| ENSMUS( | Xaf1 | 298 | 273 | 227 | 122 | 105 | 132 | 2.093006 | 1.07E-22 | 4.86E-21 up |
| ENSMUS( | Lrriq1 | 110 | 135 | 120 | 34 | 34 | 25 | 3.705732 | 1.17E-22 | 5.31E-21 up |
| ENSMUS( | Golga4 | 1171 | 1171 | 1110 | 702 | 740 | 725 | 1.506505 | 2.54E-22 | 1.12E-20 up |
| ENSMUS( | Cep70 | 424 | 433 | 369 | 196 | 230 | 202 | 1.84098 | 2.89E-22 | 1.27E-20 up |
| ENSMUS( | Gm14410 | 325 | 333 | 324 | 155 | 153 | 167 | 1.958902 | 3.69E-22 | 1.61E-20 up |
| ENSMUS( | Cfap69 | 335 | 339 | 292 | 148 | 170 | 157 | 1.918767 | 4.32E-22 | 1.87E-20 up |
| ENSMUS( | Heg1 | 512 | 532 | 472 | 285 | 275 | 295 | 1.675396 | 6.17E-22 | 2.65E-20 up |
| ENSMUS( | Ctsh | 197 | 226 | 199 | 83 | 86 | 75 | 2.408587 | 8.06E-22 | 3.43E-20 up |
| ENSMUS( | Wfdc2 | 171 | 173 | 127 | 49 | 51 | 61 | 2.754528 | 8.14E-22 | 3.46E-20 up |
| ENSMUS( | Nipbl | 825 | 894 | 759 | 477 | 463 | 521 | 1.603048 | 8.39E-22 | 3.55E-20 up |
| ENSMUS( | Col4a3 | 71 | 71 | 71 | 13 | 11 | 11 | 5.752998 | 9.46E-22 | 3.98E-20 up |
| ENSMUS( | Col19a1 | 125 | 108 | 112 | 35 | 30 | 30 | 3.426551 | 9.61E-22 | 4.04E-20 up |
| ENSMUS( | Fam178a | 1056 | 1069 | 890 | 587 | 593 | 698 | 1.515966 | 1.62E-21 | 6.71E-20 up |
| ENSMUS( | Vps13c | 1162 | 1244 | 1081 | 728 | 702 | 730 | 1.524818 | 1.97E-21 | 8.07E-20 up |
| ENSMUS( | Fzd4 | 478 | 485 | 416 | 248 | 258 | 260 | 1.69861 | 2.20E-21 | 8.98E-20 up |
| ENSMUS( | Spint2 | 646 | 702 | 644 | 353 | 353 | 393 | 1.717226 | 2.39E-21 | 9.74E-20 up |
| ENSMUS( | Ctps2 | 635 | 618 | 487 | 300 | 311 | 344 | 1.715646 | 2.41E-21 | 9.78E-20 up |
| ENSMUS( | Tbc1d32 | 414 | 423 | 353 | 218 | 192 | 194 | 1.854788 | 2.50E-21 | 1.02E-19 up |
| ENSMUS( | Fam135b | 128 | 139 | 142 | 45 | 37 | 38 | 3.229459 | 2.58E-21 | 1.04E-19 up |
| ENSMUS( | Nbeal1 | 678 | 650 | 585 | 292 | 375 | 358 | 1.763592 | 2.68E-21 | 1.08E-19 up |
| ENSMUS( | Med12 | 335 | 328 | 258 | 131 | 161 | 160 | 1.91936 | 2.71E-21 | 1.09E-19 up |
| ENSMUS( | Bst2 | 287 | 213 | 226 | 92 | 80 | 102 | 2.501235 | 2.75E-21 | 1.11E-19 up |
| ENSMUS( | Acer2 | 290 | 250 | 260 | 125 | 124 | 121 | 2.042563 | 2.88E-21 | 1.16E-19 up |
| ENSMUS( | Col6a1 | 509 | 440 | 437 | 200 | 239 | 264 | 1.866288 | 4.56E-21 | 1.81E-19 up |
| ENSMUS( | Abca8b | 637 | 594 | 517 | 336 | 349 | 347 | 1.595781 | 6.61E-21 | 2.59E-19 up |
| ENSMUS( | Iqgap1 | 564 | 518 | 445 | 282 | 259 | 235 | 1.848421 | 7.88E-21 | 3.08E-19 up |
| ENSMUS( | Cfh | 1563 | 1600 | 1272 | 901 | 899 | 963 | 1.512154 | 9.86E-21 | 3.81E-19 up |
| ENSMUS( | Slc8a1 | 232 | 196 | 201 | 92 | 89 | 84 | 2.238956 | 1.05E-20 | 4.07E-19 up |
| ENSMUS( | Syt14 | 595 | 603 | 555 | 349 | 333 | 357 | 1.594889 | 1.26E-20 | 4.80E-19 up |
| ENSMUS( | Tet2 | 530 | 591 | 486 | 282 | 267 | 323 | 1.74238 | 1.39E-20 | 5.28E-19 up |
| ENSMUS( | Cep162 | 407 | 401 | 357 | 215 | 182 | 200 | 1.840187 | 1.57E-20 | 5.94E-19 up |
| ENSMUS( | Kif13b | 146 | 139 | 140 | 50 | 50 | 44 | 2.788252 | 2.26E-20 | 8.47E-19 up |
| ENSMUS( | Cul4b | 1199 | 1256 | 1172 | 681 | 582 | 663 | 1.781363 | 2.52E-20 | 9.40E-19 up |
| ENSMUS( | Ctsc | 612 | 584 | 530 | 324 | 324 | 377 | 1.591565 | 2.79E-20 | 1.04E-18 up |
| ENSMUS( | Sstr4 | 141 | 122 | 116 | 29 | 44 | 41 | 3.143216 | 3.14E-20 | 1.17E-18 up |
| ENSMUS( | Gabpb2 | 442 | 436 | 422 | 237 | 243 | 245 | 1.696432 | 3.96E-20 | 1.45E-18 up |
| ENSMUS( | Prr32 | 282 | 263 | 208 | 117 | 121 | 106 | 2.053793 | 4.02E-20 | 1.48E-18 up |
| ENSMUS( | Vcl | 264 | 268 | 216 | 109 | 127 | 111 | 2.029199 | 4.76E-20 | 1.74E-18 up |
| ENSMUS( | Itga4 | 509 | 479 | 442 | 271 | 273 | 274 | 1.649693 | 4.93E-20 | 1.80E-18 up |
| ENSMUS( | Adcy6 | 346 | 357 | 331 | 165 | 179 | 197 | 1.810608 | 5.28E-20 | 1.93E-18 up |
| ENSMUS( | Tbc1d8b | 468 | 477 | 460 | 221 | 259 | 277 | 1.760454 | 5.54E-20 | 2.01E-18 up |
| ENSMUS( | Zc3h11a | 83 | 92 | 71 | 22 | 16 | 14 | 4.435124 | 8.88E-20 | 3.20E-18 up |
| ENSMUS( | Sgk3 | 521 | 440 | 423 | 264 | 245 | 257 | 1.702613 | 1.17E-19 | 4.17E-18 up |
| ENSMUS( | 4931406P1 | 177 | 166 | 157 | 63 | 56 | 74 | 2.451668 | 1.76E-19 | 6.20E-18 up |
| ENSMUS( | Fhad1 | 200 | 241 | 233 | 70 | 87 | 95 | 2.547492 | 2.06E-19 | 7.18E-18 up |
| ENSMUS( | Piezo1 | 95 | 94 | 108 | 18 | 29 | 23 | 4.039463 | 2.39E-19 | 8.26E-18 up |
| ENSMUS( | Ctdspl2 | 707 | 712 | 625 | 416 | 414 | 438 | 1.521961 | 2.62E-19 | 9.05E-18 up |
| ENSMUS( | Celsr3 | 610 | 648 | 549 | 356 | 364 | 378 | 1.553988 | 3.09E-19 | 1.06E-17 up |
| ENSMUS( | Pqlc3 | 275 | 239 | 187 | 88 | 97 | 99 | 2.31754 | 4.13E-19 | 1.41E-17 up |
| ENSMUS( | 8030462N | 391 | 415 | 385 | 217 | 216 | 214 | 1.741151 | 5.19E-19 | 1.75E-17 up |
| ENSMUS( | Slc4a2 | 795 | 752 | 788 | 326 | 406 | 380 | 1.991312 | 6.30E-19 | 2.11E-17 up |
| ENSMUS( | Sntb2 | 755 | 655 | 615 | 413 | 413 | 383 | 1.577059 | 6.48E-19 | 2.17E-17 up |
| ENSMUS( | Senp5 | 447 | 427 | 428 | 221 | 257 | 251 | 1.691677 | 6.59E-19 | 2.21E-17 up |
| ENSMUS( | H2-Q7 | 207 | 222 | 175 | 81 | 88 | 94 | 2.166733 | 9.49E-19 | 3.14E-17 up |
| ENSMUS( | BC067074 | 92 | 106 | 89 | 24 | 27 | 27 | 3.480583 | 9.61E-19 | 3.18E-17 up |
| ENSMUS( | Zfp970 | 319 | 339 | 301 | 158 | 174 | 170 | 1.8062 | 1.02E-18 | 3.37E-17 up |
| ENSMUS( | Nos1ap | 492 | 528 | 510 | 168 | 231 | 200 | 2.422653 | 1.10E-18 | 3.63E-17 up |
| ENSMUS( | Brwd3 | 450 | 515 | 431 | 229 | 254 | 259 | 1.779493 | 1.19E-18 | 3.90E-17 up |

| | | | | | | | | | | | |
|---|---|---|---|---|---|---|---|---|---|---|---|
| ENSMUS( | Mfsd4a | 587 | 609 | 520 | 344 | 333 | 367 | 1.551972 | 1.83E-18 | 5.90E-17 | up |
| ENSMUS( | St6galnac2 | 127 | 134 | 106 | 18 | 29 | 36 | 4.193247 | 1.85E-18 | 5.95E-17 | up |
| ENSMUS( | Als2 | 659 | 679 | 623 | 395 | 410 | 419 | 1.515208 | 2.69E-18 | 8.52E-17 | up |
| ENSMUS( | Kmt2e | 1308 | 1475 | 1330 | 806 | 843 | 865 | 1.549469 | 2.74E-18 | 8.65E-17 | up |
| ENSMUS( | Rev1 | 326 | 286 | 274 | 142 | 154 | 160 | 1.834863 | 2.87E-18 | 9.04E-17 | up |
| ENSMUS( | Kcnj13 | 166 | 160 | 134 | 56 | 57 | 64 | 2.450741 | 3.81E-18 | 1.19E-16 | up |
| ENSMUS( | Angptl2 | 200 | 193 | 165 | 68 | 79 | 91 | 2.215732 | 3.95E-18 | 1.23E-16 | up |
| ENSMUS( | Tenm3 | 659 | 682 | 622 | 406 | 383 | 440 | 1.511222 | 4.37E-18 | 1.35E-16 | up |
| ENSMUS( | S100a9 | 156 | 136 | 147 | 41 | 56 | 61 | 2.639788 | 5.33E-18 | 1.63E-16 | up |
| ENSMUS( | Pdxk | 698 | 736 | 730 | 404 | 416 | 412 | 1.664935 | 6.20E-18 | 1.89E-16 | up |
| ENSMUS( | Tc2n | 76 | 80 | 77 | 20 | 21 | 14 | 3.994914 | 6.94E-18 | 2.11E-16 | up |
| ENSMUS( | Pdpr | 298 | 308 | 310 | 143 | 146 | 153 | 1.96561 | 7.17E-18 | 2.18E-16 | up |
| ENSMUS( | Zdhhc23 | 108 | 105 | 108 | 30 | 20 | 34 | 3.628681 | 7.72E-18 | 2.33E-16 | up |
| ENSMUS( | Adgrl4 | 321 | 330 | 302 | 144 | 170 | 187 | 1.802634 | 1.20E-17 | 3.57E-16 | up |
| ENSMUS( | Rims2 | 599 | 553 | 550 | 324 | 338 | 386 | 1.53821 | 1.62E-17 | 4.80E-16 | up |
| ENSMUS( | Zkscan3 | 723 | 715 | 685 | 424 | 474 | 431 | 1.510334 | 2.19E-17 | 6.38E-16 | up |
| ENSMUS( | Atr | 249 | 224 | 203 | 107 | 98 | 116 | 1.986598 | 2.24E-17 | 6.51E-16 | up |
| ENSMUS( | Klhl11 | 211 | 224 | 243 | 65 | 77 | 55 | 3.26338 | 2.41E-17 | 7.00E-16 | up |
| ENSMUS( | Ctnnal1 | 488 | 459 | 377 | 259 | 255 | 263 | 1.603007 | 2.48E-17 | 7.20E-16 | up |
| ENSMUS( | Scube3 | 109 | 118 | 103 | 36 | 25 | 39 | 3.123443 | 2.97E-17 | 8.54E-16 | up |
| ENSMUS( | Pcolce | 409 | 429 | 340 | 193 | 219 | 247 | 1.687853 | 5.76E-17 | 1.61E-15 | up |
| ENSMUS( | Palm2 | 68 | 65 | 66 | 13 | 18 | 12 | 4.372943 | 8.89E-17 | 2.43E-15 | up |
| ENSMUS( | Col11a1 | 219 | 217 | 222 | 106 | 107 | 95 | 2.021262 | 9.85E-17 | 2.69E-15 | up |
| ENSMUS( | Itga8 | 492 | 450 | 427 | 277 | 258 | 285 | 1.576401 | 1.55E-16 | 4.18E-15 | up |
| ENSMUS( | Dcc | 89 | 58 | 62 | 14 | 14 | 13 | 4.786741 | 1.93E-16 | 5.16E-15 | up |
| ENSMUS( | Zfp551 | 73 | 79 | 72 | 15 | 24 | 17 | 3.784347 | 2.22E-16 | 5.93E-15 | up |
| ENSMUS( | Kcnh7 | 595 | 598 | 576 | 349 | 353 | 388 | 1.537242 | 2.28E-16 | 6.08E-15 | up |
| ENSMUS( | Trpc5 | 157 | 131 | 122 | 35 | 52 | 35 | 3.160749 | 2.30E-16 | 6.13E-15 | up |
| ENSMUS( | Ctla2a | 287 | 287 | 256 | 149 | 141 | 146 | 1.796527 | 2.43E-16 | 6.46E-15 | up |
| ENSMUS( | Soga1 | 137 | 150 | 164 | 45 | 60 | 48 | 2.803871 | 2.73E-16 | 7.23E-15 | up |
| ENSMUS( | Xdh | 158 | 171 | 140 | 57 | 40 | 64 | 2.753556 | 5.34E-16 | 1.39E-14 | up |
| ENSMUS( | Zfp459 | 83 | 72 | 68 | 19 | 23 | 15 | 3.676446 | 5.69E-16 | 1.47E-14 | up |
| ENSMUS( | Ankrd16 | 430 | 417 | 363 | 239 | 245 | 228 | 1.600914 | 5.71E-16 | 1.47E-14 | up |
| ENSMUS( | Zfp955a | 164 | 173 | 145 | 60 | 34 | 54 | 3.070977 | 5.91E-16 | 1.52E-14 | up |
| ENSMUS( | BC005561 | 97 | 124 | 124 | 27 | 33 | 39 | 3.329032 | 5.98E-16 | 1.54E-14 | up |
| ENSMUS( | Rsbn1 | 361 | 352 | 376 | 194 | 170 | 195 | 1.847985 | 6.37E-16 | 1.63E-14 | up |
| ENSMUS( | Abcb1a | 368 | 347 | 350 | 174 | 179 | 223 | 1.754434 | 6.83E-16 | 1.75E-14 | up |
| ENSMUS( | Lrrk2 | 445 | 432 | 377 | 239 | 241 | 267 | 1.584641 | 9.41E-16 | 2.38E-14 | up |
| ENSMUS( | Ap4e1 | 162 | 186 | 175 | 79 | 61 | 76 | 2.295193 | 9.57E-16 | 2.41E-14 | up |
| ENSMUS( | Chuk | 715 | 743 | 700 | 442 | 433 | 474 | 1.514741 | 9.86E-16 | 2.49E-14 | up |
| ENSMUS( | Polr1a | 426 | 403 | 412 | 228 | 242 | 238 | 1.659568 | 1.01E-15 | 2.56E-14 | up |
| ENSMUS( | Chil3 | 69 | 83 | 50 | 8 | 4 | 3 | 12.49614 | 1.02E-15 | 2.57E-14 | up |
| ENSMUS( | B3galnt2 | 298 | 312 | 266 | 160 | 155 | 160 | 1.740164 | 1.06E-15 | 2.65E-14 | up |
| ENSMUS( | Pde4d | 347 | 375 | 359 | 181 | 192 | 203 | 1.779892 | 1.19E-15 | 2.98E-14 | up |
| ENSMUS( | Ints2 | 432 | 446 | 425 | 240 | 272 | 256 | 1.606119 | 1.35E-15 | 3.35E-14 | up |
| ENSMUS( | Cyyr1 | 118 | 129 | 125 | 51 | 43 | 47 | 2.498856 | 2.38E-15 | 5.83E-14 | up |
| ENSMUS( | Eif2s3y | 638 | 614 | 603 | 342 | 388 | 425 | 1.52237 | 2.46E-15 | 5.99E-14 | up |
| ENSMUS( | Trim39 | 435 | 452 | 378 | 244 | 271 | 246 | 1.567092 | 2.61E-15 | 6.36E-14 | up |
| ENSMUS( | Steap1 | 140 | 108 | 103 | 35 | 26 | 16 | 4.252211 | 2.65E-15 | 6.43E-14 | up |
| ENSMUS( | Mdn1 | 488 | 487 | 419 | 285 | 260 | 311 | 1.537437 | 2.66E-15 | 6.45E-14 | up |
| ENSMUS( | Amy1 | 480 | 506 | 402 | 257 | 300 | 280 | 1.562839 | 4.12E-15 | 9.83E-14 | up |
| ENSMUS( | Slc39a14 | 599 | 603 | 535 | 349 | 391 | 352 | 1.501052 | 5.11E-15 | 1.21E-13 | up |
| ENSMUS( | Pcdhac2 | 234 | 237 | 238 | 69 | 105 | 110 | 2.37526 | 5.24E-15 | 1.24E-13 | up |
| ENSMUS( | Mndal | 126 | 127 | 111 | 48 | 38 | 53 | 2.475066 | 6.81E-15 | 1.60E-13 | up |
| ENSMUS( | Adgrf5 | 356 | 376 | 361 | 205 | 186 | 218 | 1.701041 | 7.41E-15 | 1.73E-13 | up |
| ENSMUS( | Hectd2 | 415 | 392 | 310 | 199 | 143 | 167 | 2.058927 | 8.01E-15 | 1.86E-13 | up |
| ENSMUS( | Etnppl | 246 | 210 | 237 | 95 | 115 | 113 | 2.035291 | 1.20E-14 | 2.75E-13 | up |
| ENSMUS( | Szt2 | 325 | 340 | 281 | 184 | 177 | 176 | 1.659935 | 1.20E-14 | 2.76E-13 | up |
| ENSMUS( | Stra6 | 343 | 289 | 311 | 151 | 172 | 175 | 1.793012 | 1.30E-14 | 2.96E-13 | up |
| ENSMUS( | Rgs11 | 316 | 265 | 249 | 117 | 155 | 149 | 1.860636 | 1.32E-14 | 3.00E-13 | up |
| ENSMUS( | Klf7 | 176 | 171 | 159 | 73 | 65 | 90 | 2.102067 | 1.34E-14 | 3.05E-13 | up |
| ENSMUS( | Chpf2 | 274 | 220 | 220 | 121 | 127 | 107 | 1.892717 | 1.43E-14 | 3.25E-13 | up |
| ENSMUS( | F11r | 280 | 268 | 249 | 120 | 146 | 158 | 1.779635 | 1.75E-14 | 3.93E-13 | up |
| ENSMUS( | Dok6 | 86 | 104 | 106 | 30 | 21 | 20 | 3.948059 | 2.71E-14 | 5.98E-13 | up |
| ENSMUS( | Rbm47 | 84 | 98 | 82 | 21 | 22 | 34 | 3.259704 | 3.27E-14 | 7.17E-13 | up |
| ENSMUS( | Med12l | 319 | 330 | 261 | 154 | 173 | 134 | 1.854777 | 5.02E-14 | 1.09E-12 | up |
| ENSMUS( | Krt18 | 203 | 192 | 160 | 94 | 84 | 90 | 1.948161 | 5.23E-14 | 1.13E-12 | up |
| ENSMUS( | Ryr1 | 220 | 191 | 189 | 94 | 103 | 103 | 1.889326 | 7.40E-14 | 1.57E-12 | up |
| ENSMUS( | Steap4 | 81 | 67 | 58 | 2 | 9 | 3 | 13.81047 | 7.83E-14 | 1.66E-12 | up |
| ENSMUS( | Lcor | 75 | 75 | 52 | 21 | 15 | 19 | 3.441267 | 9.40E-14 | 1.98E-12 | up |
| ENSMUS( | 2410089E( | 559 | 542 | 498 | 307 | 297 | 374 | 1.547413 | 1.52E-13 | 3.16E-12 | up |
| ENSMUS( | Xpo4 | 130 | 138 | 132 | 59 | 46 | 65 | 2.231901 | 1.66E-13 | 3.44E-12 | up |
| ENSMUS( | Aff2 | 71 | 87 | 83 | 27 | 22 | 24 | 3.131175 | 2.23E-13 | 4.56E-12 | up |
| ENSMUS( | Slc16a12 | 158 | 182 | 152 | 67 | 82 | 82 | 2.016002 | 2.32E-13 | 4.76E-12 | up |
| ENSMUS( | Myo7a | 113 | 103 | 118 | 44 | 43 | 37 | 2.550779 | 2.66E-13 | 5.43E-12 | up |
| ENSMUS( | Lrrc45 | 428 | 383 | 372 | 239 | 238 | 252 | 1.532797 | 2.76E-13 | 5.62E-12 | up |
| ENSMUS( | Chrna7 | 246 | 231 | 194 | 96 | 124 | 96 | 1.99706 | 2.76E-13 | 5.62E-12 | up |
| ENSMUS( | Zcchc11 | 550 | 549 | 462 | 311 | 350 | 304 | 1.523425 | 2.83E-13 | 5.75E-12 | up |
| ENSMUS( | Gm20878 | 289 | 273 | 240 | 134 | 155 | 160 | 1.686239 | 2.90E-13 | 5.89E-12 | up |
| ENSMUS( | Ksr2 | 512 | 515 | 501 | 280 | 328 | 283 | 1.622398 | 3.31E-13 | 6.68E-12 | up |

| | | | | | | | | | | | |
|---|---|---|---|---|---|---|---|---|---|---|---|
| ENSMUS( | Zmym6 | 509 | 623 | 509 | 316 | 303 | 338 | 1.622323 | 3.41E-13 | 6.87E-12 | up |
| ENSMUS( | Htr2a | 98 | 91 | 95 | 27 | 39 | 36 | 2.642098 | 3.45E-13 | 6.94E-12 | up |
| ENSMUS( | Nfkbiz | 269 | 229 | 209 | 124 | 130 | 128 | 1.742595 | 3.73E-13 | 7.47E-12 | up |
| ENSMUS( | Mitf | 335 | 302 | 288 | 175 | 182 | 183 | 1.617063 | 3.86E-13 | 7.72E-12 | up |
| ENSMUS( | Zfp507 | 186 | 228 | 211 | 93 | 103 | 88 | 2.085729 | 4.66E-13 | 9.23E-12 | up |
| ENSMUS( | Lama5 | 185 | 162 | 147 | 70 | 80 | 85 | 1.98359 | 4.80E-13 | 9.51E-12 | up |
| ENSMUS( | Map3k3 | 378 | 352 | 330 | 213 | 218 | 210 | 1.560343 | 4.91E-13 | 9.71E-12 | up |
| ENSMUS( | Scara5 | 62 | 52 | 58 | 15 | 11 | 17 | 3.794471 | 5.37E-13 | 1.06E-11 | up |
| ENSMUS( | Slc16a9 | 70 | 77 | 68 | 24 | 16 | 25 | 3.131344 | 5.89E-13 | 1.15E-11 | up |
| ENSMUS( | Tubgcp6 | 226 | 248 | 225 | 116 | 135 | 128 | 1.746209 | 6.96E-13 | 1.36E-11 | up |
| ENSMUS( | Yeats2 | 437 | 469 | 386 | 222 | 276 | 278 | 1.573515 | 8.78E-13 | 1.70E-11 | up |
| ENSMUS( | Slc39a4 | 100 | 116 | 99 | 39 | 46 | 38 | 2.419549 | 1.08E-12 | 2.07E-11 | up |
| ENSMUS( | Col5a2 | 148 | 170 | 149 | 82 | 68 | 71 | 1.996169 | 1.55E-12 | 2.92E-11 | up |
| ENSMUS( | Uprt | 53 | 56 | 47 | 13 | 14 | 10 | 3.964653 | 1.62E-12 | 3.04E-11 | up |
| ENSMUS( | Zfp420 | 323 | 336 | 260 | 166 | 194 | 177 | 1.610866 | 1.72E-12 | 3.21E-11 | up |
| ENSMUS( | Usp40 | 382 | 324 | 318 | 207 | 211 | 204 | 1.552346 | 1.79E-12 | 3.33E-11 | up |
| ENSMUS( | Pecam1 | 379 | 362 | 343 | 225 | 198 | 185 | 1.680545 | 2.34E-12 | 4.33E-11 | up |
| ENSMUS( | Lmln | 252 | 258 | 235 | 127 | 130 | 161 | 1.688888 | 2.35E-12 | 4.33E-11 | up |
| ENSMUS( | Myof | 242 | 220 | 199 | 104 | 129 | 105 | 1.842159 | 2.43E-12 | 4.49E-11 | up |
| ENSMUS( | Dgki | 213 | 206 | 173 | 104 | 97 | 108 | 1.805151 | 2.53E-12 | 4.65E-11 | up |
| ENSMUS( | Spg11 | 202 | 199 | 205 | 101 | 100 | 114 | 1.825486 | 2.92E-12 | 5.33E-11 | up |
| ENSMUS( | Ifi44 | 64 | 56 | 57 | 13 | 20 | 12 | 3.70909 | 3.36E-12 | 6.11E-11 | up |
| ENSMUS( | Irs1 | 133 | 122 | 133 | 33 | 47 | 59 | 2.661362 | 3.74E-12 | 6.76E-11 | up |
| ENSMUS( | Sntb1 | 92 | 91 | 70 | 27 | 28 | 35 | 2.650329 | 4.02E-12 | 7.22E-11 | up |
| ENSMUS( | Phka2 | 359 | 330 | 312 | 188 | 219 | 203 | 1.549517 | 4.21E-12 | 7.55E-11 | up |
| ENSMUS( | H2-Eb1 | 187 | 175 | 152 | 86 | 89 | 82 | 1.882514 | 4.46E-12 | 7.98E-11 | up |
| ENSMUS( | Nlrc5 | 46 | 51 | 45 | 10 | 9 | 13 | 4.210806 | 4.79E-12 | 8.55E-11 | up |
| ENSMUS( | Cast | 147 | 149 | 152 | 77 | 66 | 70 | 1.991681 | 5.82E-12 | 1.03E-10 | up |
| ENSMUS( | Fan1 | 101 | 106 | 92 | 32 | 44 | 42 | 2.397778 | 6.05E-12 | 1.07E-10 | up |
| ENSMUS( | Osmr | 161 | 163 | 148 | 70 | 79 | 81 | 1.941215 | 7.06E-12 | 1.24E-10 | up |
| ENSMUS( | Plac8 | 48 | 42 | 40 | 12 | 7 | 8 | 4.521587 | 7.10E-12 | 1.25E-10 | up |
| ENSMUS( | Clcnka | 54 | 43 | 52 | 11 | 13 | 12 | 3.924503 | 7.79E-12 | 1.36E-10 | up |
| ENSMUS( | Pde11a | 118 | 128 | 129 | 45 | 62 | 49 | 2.280938 | 8.29E-12 | 1.45E-10 | up |
| ENSMUS( | Raf1 | 358 | 381 | 327 | 223 | 217 | 224 | 1.515684 | 9.76E-12 | 1.69E-10 | up |
| ENSMUS( | Lysmd3 | 333 | 301 | 282 | 189 | 178 | 185 | 1.564919 | 1.05E-11 | 1.82E-10 | up |
| ENSMUS( | Ccdc39 | 536 | 437 | 475 | 280 | 270 | 247 | 1.714646 | 1.14E-11 | 1.96E-10 | up |
| ENSMUS( | Lin7a | 237 | 201 | 203 | 99 | 110 | 134 | 1.770142 | 1.14E-11 | 1.96E-10 | up |
| ENSMUS( | D130043K | 295 | 291 | 289 | 164 | 180 | 178 | 1.58772 | 1.18E-11 | 2.03E-10 | up |
| ENSMUS( | Wfikkn2 | 113 | 142 | 110 | 51 | 54 | 40 | 2.370192 | 1.20E-11 | 2.05E-10 | up |
| ENSMUS( | Cd46 | 182 | 173 | 146 | 88 | 73 | 91 | 1.873209 | 1.20E-11 | 2.06E-10 | up |
| ENSMUS( | Zfp329 | 457 | 514 | 456 | 265 | 313 | 291 | 1.554365 | 1.33E-11 | 2.26E-10 | up |
| ENSMUS( | Map3k13 | 206 | 233 | 223 | 107 | 109 | 122 | 1.85975 | 1.48E-11 | 2.51E-10 | up |
| ENSMUS( | Sptlc3 | 29 | 33 | 37 | 5 | 4 | 6 | 6.305366 | 1.66E-11 | 2.80E-10 | up |
| ENSMUS( | Dnah9 | 365 | 318 | 297 | 202 | 180 | 221 | 1.53366 | 1.72E-11 | 2.89E-10 | up |
| ENSMUS( | Cdr2 | 372 | 323 | 313 | 208 | 207 | 209 | 1.523846 | 1.73E-11 | 2.91E-10 | up |
| ENSMUS( | Maff | 68 | 65 | 49 | 13 | 19 | 22 | 3.180589 | 1.81E-11 | 3.03E-10 | up |
| ENSMUS( | Gbp2 | 221 | 220 | 178 | 90 | 110 | 125 | 1.798941 | 1.94E-11 | 3.25E-10 | up |
| ENSMUS( | Helz2 | 78 | 74 | 61 | 22 | 25 | 24 | 2.824427 | 2.02E-11 | 3.37E-10 | up |
| ENSMUS( | Gpr63 | 107 | 62 | 78 | 25 | 23 | 24 | 3.229379 | 2.12E-11 | 3.53E-10 | up |
| ENSMUS( | Pon3 | 126 | 132 | 141 | 52 | 53 | 66 | 2.222387 | 2.28E-11 | 3.78E-10 | up |
| ENSMUS( | Adamts3 | 105 | 116 | 104 | 43 | 47 | 48 | 2.230019 | 2.58E-11 | 4.25E-10 | up |
| ENSMUS( | Pla2g3 | 101 | 103 | 110 | 30 | 34 | 49 | 2.652961 | 2.72E-11 | 4.45E-10 | up |
| ENSMUS( | Cfap54 | 450 | 424 | 388 | 279 | 266 | 247 | 1.501154 | 3.45E-11 | 5.60E-10 | up |
| ENSMUS( | Ms4a6b | 123 | 129 | 106 | 49 | 50 | 61 | 2.114713 | 3.55E-11 | 5.75E-10 | up |
| ENSMUS( | Metap1d | 285 | 231 | 203 | 125 | 121 | 136 | 1.769977 | 3.79E-11 | 6.10E-10 | up |
| ENSMUS( | Gm9938 | 54 | 52 | 58 | 14 | 19 | 13 | 3.379036 | 3.84E-11 | 6.18E-10 | up |
| ENSMUS( | Il1r1 | 472 | 406 | 339 | 250 | 241 | 239 | 1.56506 | 3.85E-11 | 6.19E-10 | up |
| ENSMUS( | Samd9l | 188 | 171 | 161 | 99 | 79 | 86 | 1.855909 | 3.92E-11 | 6.30E-10 | up |
| ENSMUS( | Filip1 | 211 | 210 | 190 | 111 | 108 | 116 | 1.723003 | 4.15E-11 | 6.64E-10 | up |
| ENSMUS( | Actbl2 | 45 | 48 | 48 | 4 | 2 | 0 | 21.80381 | 4.17E-11 | 6.67E-10 | up |
| ENSMUS( | Ccdc66 | 290 | 293 | 281 | 183 | 171 | 160 | 1.587796 | 4.30E-11 | 6.86E-10 | up |
| ENSMUS( | Igtp | 171 | 150 | 121 | 74 | 79 | 64 | 1.90858 | 4.90E-11 | 7.79E-10 | up |
| ENSMUS( | Il6ra | 178 | 160 | 134 | 74 | 86 | 78 | 1.864991 | 5.44E-11 | 8.60E-10 | up |
| ENSMUS( | Rad54l2 | 366 | 366 | 295 | 172 | 181 | 236 | 1.647451 | 5.53E-11 | 8.73E-10 | up |
| ENSMUS( | Rdh5 | 189 | 183 | 198 | 100 | 89 | 80 | 2.004939 | 5.80E-11 | 9.13E-10 | up |
| ENSMUS( | Fras1 | 224 | 195 | 168 | 99 | 114 | 89 | 1.824744 | 7.28E-11 | 1.14E-09 | up |
| ENSMUS( | Parp14 | 99 | 109 | 83 | 40 | 38 | 42 | 2.284886 | 7.33E-11 | 1.14E-09 | up |
| ENSMUS( | Pglyrp1 | 114 | 110 | 121 | 47 | 57 | 46 | 2.180896 | 7.55E-11 | 1.18E-09 | up |
| ENSMUS( | Gpam | 636 | 606 | 617 | 340 | 406 | 345 | 1.612327 | 9.03E-11 | 1.40E-09 | up |
| ENSMUS( | Hs6st3 | 53 | 56 | 48 | 17 | 16 | 11 | 3.35167 | 9.63E-11 | 1.48E-09 | up |
| ENSMUS( | Ccdc141 | 354 | 394 | 357 | 198 | 231 | 195 | 1.674778 | 1.02E-10 | 1.58E-09 | up |
| ENSMUS( | Slc16a4 | 254 | 220 | 166 | 82 | 111 | 100 | 2.049558 | 1.05E-10 | 1.61E-09 | up |
| ENSMUS( | Zc3h12c | 261 | 245 | 240 | 137 | 153 | 149 | 1.607246 | 1.16E-10 | 1.77E-09 | up |
| ENSMUS( | Gprin3 | 192 | 172 | 161 | 80 | 86 | 109 | 1.806862 | 1.24E-10 | 1.88E-09 | up |
| ENSMUS( | Fbn1 | 230 | 218 | 209 | 120 | 125 | 129 | 1.660881 | 1.30E-10 | 1.98E-09 | up |
| ENSMUS( | Fap | 126 | 110 | 91 | 30 | 15 | 49 | 3.295234 | 1.37E-10 | 2.07E-09 | up |
| ENSMUS( | Stxbp5l | 343 | 348 | 381 | 119 | 121 | 175 | 2.465283 | 1.42E-10 | 2.15E-09 | up |
| ENSMUS( | Ackr4 | 44 | 35 | 31 | 9 | 8 | 5 | 4.666398 | 1.46E-10 | 2.20E-09 | up |
| ENSMUS( | Rrh | 39 | 38 | 26 | 5 | 6 | 8 | 5.1055 | 1.51E-10 | 2.27E-09 | up |
| ENSMUS( | Slit3 | 284 | 283 | 335 | 130 | 164 | 164 | 1.878252 | 1.52E-10 | 2.29E-09 | up |

| | | | | | | | | | | |
|---|---|---|---|---|---|---|---|---|---|---|
| ENSMUS( | Intu | 312 | 315 | 281 | 179 | 187 | 197 | 1.524125 | 1.74E-10 | 2.59E-09 up |
| ENSMUS( | Cfap44 | 154 | 158 | 159 | 78 | 80 | 84 | 1.845833 | 1.79E-10 | 2.67E-09 up |
| ENSMUS( | Ocln | 371 | 359 | 291 | 172 | 225 | 210 | 1.585011 | 1.80E-10 | 2.68E-09 up |
| ENSMUS( | Slc16a6 | 149 | 146 | 147 | 82 | 65 | 75 | 1.883724 | 1.84E-10 | 2.74E-09 up |
| ENSMUS( | Ddr2 | 269 | 266 | 220 | 139 | 165 | 142 | 1.593814 | 1.92E-10 | 2.85E-09 up |
| ENSMUS( | AC153134 | 368 | 334 | 306 | 219 | 186 | 223 | 1.513954 | 1.98E-10 | 2.93E-09 up |
| ENSMUS( | Gm2026 | 74 | 83 | 78 | 33 | 32 | 24 | 2.492589 | 2.13E-10 | 3.14E-09 up |
| ENSMUS( | Oasl2 | 149 | 121 | 130 | 64 | 60 | 70 | 1.950297 | 2.16E-10 | 3.19E-09 up |
| ENSMUS( | Hif3a | 260 | 230 | 252 | 141 | 135 | 138 | 1.697024 | 2.41E-10 | 3.53E-09 up |
| ENSMUS( | Gbp9 | 130 | 102 | 113 | 51 | 38 | 58 | 2.222629 | 2.49E-10 | 3.64E-09 up |
| ENSMUS( | Syne2 | 286 | 276 | 235 | 148 | 173 | 161 | 1.558497 | 2.68E-10 | 3.91E-09 up |
| ENSMUS( | Ttc37 | 307 | 363 | 337 | 195 | 184 | 199 | 1.652298 | 2.71E-10 | 3.95E-09 up |
| ENSMUS( | Irgm1 | 359 | 337 | 313 | 173 | 157 | 237 | 1.686562 | 3.15E-10 | 4.55E-09 up |
| ENSMUS( | Iigp1 | 204 | 173 | 130 | 75 | 74 | 57 | 2.297308 | 3.17E-10 | 4.58E-09 up |
| ENSMUS( | Asah2 | 266 | 270 | 214 | 153 | 138 | 157 | 1.576314 | 3.29E-10 | 4.75E-09 up |
| ENSMUS( | Mdfic | 277 | 268 | 246 | 150 | 175 | 155 | 1.555601 | 4.18E-10 | 5.97E-09 up |
| ENSMUS( | Saa3 | 29 | 36 | 37 | 3 | 3 | 0 | 15.89097 | 4.47E-10 | 6.36E-09 up |
| ENSMUS( | Cox17 | 90 | 104 | 75 | 39 | 36 | 26 | 2.495208 | 4.54E-10 | 6.46E-09 up |
| ENSMUS( | Kcnk9 | 44 | 40 | 42 | 12 | 12 | 7 | 3.82419 | 4.62E-10 | 6.56E-09 up |
| ENSMUS( | Zfp974 | 218 | 230 | 189 | 117 | 127 | 122 | 1.641221 | 4.73E-10 | 6.71E-09 up |
| ENSMUS( | Ms4a4b | 39 | 45 | 26 | 7 | 5 | 11 | 4.506856 | 4.96E-10 | 7.01E-09 up |
| ENSMUS( | Sik2 | 93 | 118 | 113 | 33 | 48 | 48 | 2.394606 | 5.29E-10 | 7.45E-09 up |
| ENSMUS( | Nek10 | 48 | 26 | 30 | 3 | 7 | 7 | 5.776988 | 5.29E-10 | 7.46E-09 up |
| ENSMUS( | Tmem74 | 171 | 189 | 175 | 80 | 90 | 112 | 1.803071 | 5.39E-10 | 7.59E-09 up |
| ENSMUS( | Cyb5d1 | 343 | 311 | 256 | 131 | 189 | 185 | 1.698904 | 6.16E-10 | 8.61E-09 up |
| ENSMUS( | Pcdhga6 | 135 | 148 | 119 | 64 | 49 | 73 | 2.042479 | 6.47E-10 | 9.03E-09 up |
| ENSMUS( | Pigl | 140 | 117 | 122 | 64 | 60 | 60 | 1.944164 | 6.69E-10 | 9.34E-09 up |
| ENSMUS( | Fcgr4 | 39 | 29 | 49 | 6 | 6 | 9 | 5.354367 | 6.86E-10 | 9.57E-09 up |
| ENSMUS( | Podn | 114 | 139 | 147 | 54 | 59 | 56 | 2.254272 | 7.04E-10 | 9.80E-09 up |
| ENSMUS( | Wars2 | 133 | 138 | 142 | 60 | 77 | 67 | 1.921394 | 7.20E-10 | 1.00E-08 up |
| ENSMUS( | Sfrp5 | 72 | 76 | 46 | 16 | 21 | 11 | 3.763463 | 7.23E-10 | 1.00E-08 up |
| ENSMUS( | Cdh19 | 279 | 265 | 234 | 148 | 153 | 174 | 1.54681 | 9.17E-10 | 1.26E-08 up |
| ENSMUS( | Nek4 | 221 | 236 | 210 | 122 | 144 | 126 | 1.607944 | 9.82E-10 | 1.35E-08 up |
| ENSMUS( | Mc3r | 34 | 26 | 28 | 7 | 4 | 4 | 5.502772 | 1.13E-09 | 1.53E-08 up |
| ENSMUS( | Atxn7 | 437 | 502 | 435 | 231 | 291 | 273 | 1.636662 | 1.15E-09 | 1.56E-08 up |
| ENSMUS( | Zfp966 | 64 | 78 | 72 | 28 | 22 | 31 | 2.51034 | 1.26E-09 | 1.70E-08 up |
| ENSMUS( | Trim56 | 227 | 215 | 196 | 121 | 117 | 134 | 1.620095 | 1.28E-09 | 1.73E-08 up |
| ENSMUS( | Gm2163 | 126 | 104 | 116 | 61 | 44 | 55 | 2.044598 | 1.39E-09 | 1.87E-08 up |
| ENSMUS( | Dgkh | 86 | 93 | 103 | 27 | 36 | 21 | 3.183048 | 1.41E-09 | 1.89E-08 up |
| ENSMUS( | Tarbp1 | 230 | 208 | 191 | 108 | 120 | 138 | 1.624487 | 1.44E-09 | 1.93E-08 up |
| ENSMUS( | Ifi203 | 117 | 132 | 138 | 52 | 56 | 65 | 2.131648 | 1.47E-09 | 1.97E-08 up |
| ENSMUS( | Garem1 | 445 | 457 | 357 | 274 | 222 | 272 | 1.543033 | 1.50E-09 | 2.01E-08 up |
| ENSMUS( | B4galt1 | 227 | 218 | 216 | 122 | 134 | 134 | 1.604594 | 1.54E-09 | 2.05E-08 up |
| ENSMUS( | Slc26a2 | 232 | 245 | 220 | 147 | 124 | 146 | 1.580065 | 1.56E-09 | 2.08E-08 up |
| ENSMUS( | Col12a1 | 210 | 203 | 170 | 117 | 111 | 97 | 1.685436 | 1.57E-09 | 2.09E-08 up |
| ENSMUS( | Zfp605 | 312 | 301 | 284 | 170 | 128 | 202 | 1.699609 | 1.87E-09 | 2.46E-08 up |
| ENSMUS( | Cemip | 310 | 303 | 301 | 159 | 204 | 194 | 1.555492 | 1.90E-09 | 2.50E-08 up |
| ENSMUS( | Zfp185 | 171 | 155 | 155 | 90 | 64 | 59 | 2.124522 | 2.05E-09 | 2.69E-08 up |
| ENSMUS( | Postn | 185 | 161 | 133 | 88 | 87 | 84 | 1.735998 | 2.35E-09 | 3.06E-08 up |
| ENSMUS( | Cyp4f15 | 289 | 304 | 243 | 181 | 140 | 165 | 1.61929 | 2.75E-09 | 3.54E-08 up |
| ENSMUS( | Htr1b | 213 | 217 | 183 | 105 | 125 | 128 | 1.617363 | 2.98E-09 | 3.82E-08 up |
| ENSMUS( | Rnf146 | 260 | 291 | 243 | 162 | 158 | 174 | 1.519028 | 3.20E-09 | 4.09E-08 up |
| ENSMUS( | Cntrl | 243 | 199 | 210 | 125 | 127 | 126 | 1.629029 | 3.31E-09 | 4.24E-08 up |
| ENSMUS( | Tubgcp4 | 223 | 262 | 213 | 120 | 102 | 143 | 1.810714 | 3.44E-09 | 4.40E-08 up |
| ENSMUS( | Sla | 226 | 207 | 187 | 123 | 109 | 132 | 1.607171 | 3.45E-09 | 4.41E-08 up |
| ENSMUS( | Gm14391 | 98 | 124 | 93 | 44 | 38 | 26 | 2.736656 | 3.69E-09 | 4.69E-08 up |
| ENSMUS( | Kcnj6 | 276 | 268 | 259 | 133 | 176 | 165 | 1.604555 | 4.01E-09 | 5.08E-08 up |
| ENSMUS( | Ankrd26 | 276 | 264 | 279 | 169 | 164 | 157 | 1.582514 | 4.46E-09 | 5.63E-08 up |
| ENSMUS( | Col4a4 | 100 | 99 | 73 | 28 | 45 | 35 | 2.367435 | 4.57E-09 | 5.76E-08 up |
| ENSMUS( | Dgkq | 179 | 194 | 167 | 80 | 109 | 104 | 1.744556 | 4.57E-09 | 5.76E-08 up |
| ENSMUS( | Tmem27 | 59 | 70 | 64 | 5 | 20 | 15 | 4.60249 | 4.82E-09 | 6.04E-08 up |
| ENSMUS( | Eif2ak2 | 194 | 202 | 177 | 109 | 107 | 83 | 1.803709 | 5.27E-09 | 6.57E-08 up |
| ENSMUS( | Msr1 | 48 | 37 | 29 | 6 | 12 | 11 | 3.695388 | 5.76E-09 | 7.14E-08 up |
| ENSMUS( | H2-T24 | 74 | 89 | 54 | 26 | 23 | 28 | 2.645604 | 6.33E-09 | 7.80E-08 up |
| ENSMUS( | Ern1 | 208 | 212 | 170 | 110 | 117 | 118 | 1.611124 | 6.35E-09 | 7.82E-08 up |
| ENSMUS( | Slc9a7 | 80 | 83 | 98 | 38 | 29 | 34 | 2.45895 | 6.38E-09 | 7.85E-08 up |
| ENSMUS( | Khnyn | 169 | 166 | 147 | 75 | 95 | 96 | 1.71332 | 6.76E-09 | 8.29E-08 up |
| ENSMUS( | Trim30a | 142 | 127 | 130 | 57 | 46 | 76 | 2.116276 | 6.78E-09 | 8.31E-08 up |
| ENSMUS( | Cfap65 | 144 | 144 | 115 | 63 | 78 | 69 | 1.806771 | 7.21E-09 | 8.77E-08 up |
| ENSMUS( | Rpgrip1l | 543 | 497 | 519 | 336 | 338 | 301 | 1.511224 | 7.43E-09 | 9.03E-08 up |
| ENSMUS( | S100a8 | 79 | 86 | 66 | 25 | 19 | 36 | 2.73373 | 8.74E-09 | 1.05E-07 up |
| ENSMUS( | Slc25a30 | 119 | 92 | 97 | 47 | 42 | 26 | 2.510115 | 9.79E-09 | 1.17E-07 up |
| ENSMUS( | Gon4l | 335 | 368 | 336 | 172 | 222 | 223 | 1.597311 | 1.03E-08 | 1.23E-07 up |
| ENSMUS( | Ppp1r3b | 109 | 109 | 75 | 36 | 45 | 51 | 2.088243 | 1.08E-08 | 1.28E-07 up |
| ENSMUS( | Cybb | 74 | 62 | 62 | 27 | 29 | 21 | 2.418585 | 1.13E-08 | 1.34E-07 up |
| ENSMUS( | Tgtp2 | 91 | 97 | 103 | 37 | 36 | 53 | 2.20392 | 1.14E-08 | 1.35E-07 up |
| ENSMUS( | Ptprc | 178 | 173 | 138 | 83 | 99 | 92 | 1.67966 | 1.23E-08 | 1.44E-07 up |
| ENSMUS( | Icam1 | 111 | 108 | 71 | 37 | 42 | 44 | 2.210262 | 1.33E-08 | 1.56E-07 up |
| ENSMUS( | Cfap43 | 168 | 153 | 148 | 83 | 90 | 87 | 1.703785 | 1.40E-08 | 1.63E-07 up |
| ENSMUS( | Glb1l | 148 | 120 | 113 | 65 | 44 | 67 | 2.039817 | 1.48E-08 | 1.73E-07 up |

| | | | | | | | | | | | |
|---|---|---|---|---|---|---|---|---|---|---|---|
| ENSMUS( | Mmp8 | 17 | 30 | 19 | 4 | 2 | 2 | 7.736245 | 1.50E-08 | 1.75E-07 | up |
| ENSMUS( | Spef2 | 145 | 194 | 156 | 69 | 83 | 96 | 1.89564 | 1.74E-08 | 2.01E-07 | up |
| ENSMUS( | Kri1 | 121 | 138 | 128 | 74 | 64 | 65 | 1.803728 | 2.02E-08 | 2.31E-07 | up |
| ENSMUS( | Ifit3 | 354 | 271 | 270 | 176 | 181 | 182 | 1.56375 | 2.30E-08 | 2.62E-07 | up |
| ENSMUS( | Adam17 | 303 | 289 | 227 | 182 | 166 | 155 | 1.527359 | 2.38E-08 | 2.71E-07 | up |
| ENSMUS( | 2810474O | 290 | 297 | 253 | 183 | 172 | 145 | 1.580252 | 2.48E-08 | 2.82E-07 | up |
| ENSMUS( | Cd163 | 93 | 92 | 55 | 26 | 32 | 33 | 2.468932 | 2.71E-08 | 3.06E-07 | up |
| ENSMUS( | Cdh7 | 164 | 177 | 153 | 88 | 86 | 108 | 1.658797 | 2.97E-08 | 3.34E-07 | up |
| ENSMUS( | Vash1 | 102 | 104 | 72 | 47 | 38 | 44 | 2.020324 | 2.98E-08 | 3.35E-07 | up |
| ENSMUS( | Serac1 | 236 | 197 | 173 | 120 | 120 | 120 | 1.582163 | 3.03E-08 | 3.40E-07 | up |
| ENSMUS( | Pcdhga2 | 58 | 58 | 50 | 17 | 23 | 21 | 2.570422 | 3.80E-08 | 4.22E-07 | up |
| ENSMUS( | Gbp3 | 148 | 177 | 138 | 72 | 82 | 98 | 1.739746 | 3.93E-08 | 4.36E-07 | up |
| ENSMUS( | Slc26a7 | 132 | 124 | 139 | 70 | 48 | 56 | 2.149637 | 4.26E-08 | 4.70E-07 | up |
| ENSMUS( | Gm3411 | 39 | 33 | 51 | 6 | 13 | 11 | 3.937809 | 4.34E-08 | 4.77E-07 | up |
| ENSMUS( | Arhgef5 | 104 | 97 | 71 | 34 | 50 | 44 | 1.996197 | 4.62E-08 | 5.07E-07 | up |
| ENSMUS( | Sbno2 | 232 | 237 | 201 | 128 | 152 | 126 | 1.555139 | 4.73E-08 | 5.19E-07 | up |
| ENSMUS( | Paqr5 | 138 | 119 | 102 | 56 | 49 | 73 | 1.9028 | 4.79E-08 | 5.24E-07 | up |
| ENSMUS( | C3 | 87 | 88 | 63 | 40 | 26 | 33 | 2.252564 | 4.79E-08 | 5.24E-07 | up |
| ENSMUS( | Syt10 | 62 | 79 | 64 | 18 | 32 | 25 | 2.590408 | 4.86E-08 | 5.30E-07 | up |
| ENSMUS( | Thsd4 | 340 | 352 | 363 | 231 | 221 | 214 | 1.501178 | 5.11E-08 | 5.57E-07 | up |
| ENSMUS( | Ms4a4c | 17 | 21 | 25 | 2 | 4 | 3 | 6.707904 | 5.12E-08 | 5.57E-07 | up |
| ENSMUS( | Fmo2 | 99 | 72 | 91 | 35 | 34 | 42 | 2.2403 | 5.17E-08 | 5.62E-07 | up |
| ENSMUS( | Cd300lf | 79 | 49 | 64 | 15 | 23 | 27 | 2.805659 | 5.22E-08 | 5.67E-07 | up |
| ENSMUS( | Perp | 295 | 253 | 198 | 122 | 121 | 159 | 1.745694 | 5.35E-08 | 5.80E-07 | up |
| ENSMUS( | Wdfy2 | 51 | 44 | 69 | 17 | 16 | 16 | 3.201169 | 5.64E-08 | 6.11E-07 | up |
| ENSMUS( | Pla2g5 | 126 | 107 | 118 | 48 | 66 | 43 | 2.111637 | 6.20E-08 | 6.66E-07 | up |
| ENSMUS( | Tnfsf10 | 84 | 68 | 67 | 25 | 29 | 40 | 2.207469 | 6.31E-08 | 6.76E-07 | up |
| ENSMUS( | Cdc14b | 235 | 251 | 217 | 155 | 119 | 141 | 1.598531 | 6.90E-08 | 7.35E-07 | up |
| ENSMUS( | Adam32 | 25 | 21 | 26 | 6 | 3 | 4 | 5.23733 | 7.17E-08 | 7.62E-07 | up |
| ENSMUS( | Sned1 | 136 | 140 | 112 | 74 | 53 | 71 | 1.845402 | 7.83E-08 | 8.28E-07 | up |
| ENSMUS( | Zfp109 | 58 | 86 | 84 | 32 | 24 | 29 | 2.555596 | 8.05E-08 | 8.51E-07 | up |
| ENSMUS( | Nfxl1 | 267 | 297 | 234 | 151 | 183 | 152 | 1.547129 | 8.23E-08 | 8.67E-07 | up |
| ENSMUS( | Zbtb25 | 214 | 214 | 198 | 132 | 130 | 125 | 1.527347 | 8.64E-08 | 9.09E-07 | up |
| ENSMUS( | Spaca6 | 367 | 298 | 276 | 159 | 204 | 155 | 1.707414 | 9.43E-08 | 9.86E-07 | up |
| ENSMUS( | Lonrf3 | 96 | 137 | 120 | 55 | 47 | 39 | 2.368806 | 9.75E-08 | 1.02E-06 | up |
| ENSMUS( | Itga1 | 61 | 78 | 64 | 30 | 29 | 19 | 2.44997 | 1.01E-07 | 1.05E-06 | up |
| ENSMUS( | Rnf152 | 167 | 148 | 140 | 76 | 88 | 96 | 1.654408 | 1.10E-07 | 1.14E-06 | up |
| ENSMUS( | Klf12 | 37 | 39 | 34 | 6 | 9 | 2 | 6.050713 | 1.18E-07 | 1.21E-06 | up |
| ENSMUS( | Mpp7 | 140 | 148 | 134 | 84 | 54 | 63 | 1.979142 | 1.19E-07 | 1.22E-06 | up |
| ENSMUS( | Cep164 | 197 | 232 | 183 | 132 | 113 | 118 | 1.589274 | 1.19E-07 | 1.23E-06 | up |
| ENSMUS( | Lmx1a | 45 | 43 | 35 | 13 | 15 | 11 | 2.959962 | 1.20E-07 | 1.23E-06 | up |
| ENSMUS( | Ccl28 | 124 | 119 | 123 | 71 | 68 | 56 | 1.772732 | 1.22E-07 | 1.25E-06 | up |
| ENSMUS( | Mppe1 | 142 | 144 | 134 | 66 | 89 | 76 | 1.720134 | 1.23E-07 | 1.26E-06 | up |
| ENSMUS( | Ddx58 | 182 | 189 | 149 | 102 | 113 | 94 | 1.582279 | 1.25E-07 | 1.28E-06 | up |
| ENSMUS( | Pggt1b | 167 | 139 | 130 | 80 | 77 | 90 | 1.664455 | 1.32E-07 | 1.35E-06 | up |
| ENSMUS( | Odf2l | 245 | 267 | 269 | 163 | 158 | 151 | 1.568563 | 1.34E-07 | 1.37E-06 | up |
| ENSMUS( | Phf11d | 72 | 72 | 46 | 23 | 27 | 25 | 2.371664 | 1.43E-07 | 1.45E-06 | up |
| ENSMUS( | Ifitm3 | 441 | 395 | 328 | 270 | 229 | 223 | 1.51225 | 1.66E-07 | 1.67E-06 | up |
| ENSMUS( | Fam46c | 129 | 151 | 137 | 76 | 80 | 73 | 1.723827 | 1.72E-07 | 1.73E-06 | up |
| ENSMUS( | Cd244 | 16 | 12 | 18 | 0 | 2 | 2 | 11.11897 | 1.88E-07 | 1.87E-06 | up |
| ENSMUS( | Pcdhga7 | 98 | 95 | 82 | 41 | 39 | 55 | 1.927113 | 1.90E-07 | 1.89E-06 | up |
| ENSMUS( | Alms1 | 130 | 145 | 110 | 54 | 63 | 82 | 1.830631 | 1.99E-07 | 1.98E-06 | up |
| ENSMUS( | Mrs2 | 275 | 237 | 273 | 99 | 147 | 136 | 1.95307 | 2.13E-07 | 2.11E-06 | up |
| ENSMUS( | Serpinb8 | 92 | 106 | 78 | 47 | 45 | 32 | 2.086658 | 2.13E-07 | 2.11E-06 | up |
| ENSMUS( | 1700109H | 36 | 34 | 39 | 4 | 8 | 12 | 4.360738 | 2.17E-07 | 2.14E-06 | up |
| ENSMUS( | Itpripl2 | 142 | 131 | 127 | 56 | 82 | 70 | 1.819219 | 2.19E-07 | 2.16E-06 | up |
| ENSMUS( | Jmjd8 | 217 | 211 | 177 | 113 | 135 | 129 | 1.512944 | 2.24E-07 | 2.21E-06 | up |
| ENSMUS( | Nynrin | 36 | 46 | 19 | 6 | 10 | 6 | 4.267049 | 2.47E-07 | 2.43E-06 | up |
| ENSMUS( | Ccdc171 | 58 | 45 | 52 | 19 | 21 | 19 | 2.484054 | 2.48E-07 | 2.43E-06 | up |
| ENSMUS( | Alas2 | 133 | 110 | 110 | 70 | 62 | 58 | 1.748718 | 2.49E-07 | 2.44E-06 | up |
| ENSMUS( | Irgm2 | 192 | 169 | 127 | 83 | 73 | 51 | 2.197515 | 2.50E-07 | 2.46E-06 | up |
| ENSMUS( | Pcdhgb1 | 46 | 64 | 52 | 22 | 18 | 21 | 2.51535 | 2.56E-07 | 2.51E-06 | up |
| ENSMUS( | Pcdhga8 | 65 | 49 | 49 | 22 | 11 | 11 | 3.461225 | 2.72E-07 | 2.65E-06 | up |
| ENSMUS( | Mettl4 | 120 | 129 | 121 | 65 | 70 | 68 | 1.725877 | 3.03E-07 | 2.93E-06 | up |
| ENSMUS( | Zfp619 | 126 | 107 | 96 | 61 | 50 | 63 | 1.780369 | 3.05E-07 | 2.94E-06 | up |
| ENSMUS( | Pon1 | 38 | 52 | 32 | 10 | 11 | 17 | 3.03788 | 3.32E-07 | 3.19E-06 | up |
| ENSMUS( | Map3k15 | 66 | 66 | 54 | 21 | 24 | 34 | 2.229651 | 3.58E-07 | 3.43E-06 | up |
| ENSMUS( | Gm38393 | 210 | 249 | 210 | 130 | 128 | 151 | 1.549495 | 3.63E-07 | 3.47E-06 | up |
| ENSMUS( | Tmppe | 56 | 46 | 48 | 23 | 17 | 17 | 2.476888 | 3.70E-07 | 3.53E-06 | up |
| ENSMUS( | Abca7 | 186 | 187 | 148 | 106 | 106 | 104 | 1.550855 | 3.72E-07 | 3.55E-06 | up |
| ENSMUS( | Nid2 | 127 | 168 | 140 | 49 | 76 | 61 | 2.217976 | 3.73E-07 | 3.56E-06 | up |
| ENSMUS( | Gpr165 | 103 | 115 | 94 | 49 | 49 | 65 | 1.812618 | 3.78E-07 | 3.60E-06 | up |
| ENSMUS( | Cndp1 | 40 | 28 | 26 | 5 | 11 | 6 | 4.005776 | 3.97E-07 | 3.77E-06 | up |
| ENSMUS( | Zfp990 | 60 | 62 | 51 | 14 | 18 | 28 | 2.739038 | 4.13E-07 | 3.92E-06 | up |
| ENSMUS( | Slco1a5 | 34 | 30 | 36 | 9 | 9 | 11 | 3.282507 | 4.20E-07 | 3.98E-06 | up |
| ENSMUS( | Pcdhgb5 | 48 | 37 | 54 | 11 | 17 | 17 | 2.953487 | 4.25E-07 | 4.02E-06 | up |
| ENSMUS( | Gbp8 | 33 | 38 | 25 | 8 | 10 | 9 | 3.340653 | 4.45E-07 | 4.19E-06 | up |
| ENSMUS( | Glb1l2 | 76 | 83 | 57 | 34 | 26 | 39 | 2.053337 | 4.62E-07 | 4.33E-06 | up |
| ENSMUS( | Lancl3 | 37 | 32 | 21 | 7 | 11 | 6 | 3.491697 | 4.67E-07 | 4.38E-06 | up |
| ENSMUS( | Slc16a8 | 115 | 89 | 70 | 6 | 29 | 31 | 3.929082 | 4.72E-07 | 4.42E-06 | up |

| | | | | | | | | | | | |
|---|---|---|---|---|---|---|---|---|---|---|---|
| ENSMUS( | Ifih1 | 121 | 124 | 122 | 76 | 64 | 63 | 1.708685 | 4.84E-07 | 4.53E-06 | up |
| ENSMUS( | Gcfc2 | 102 | 94 | 72 | 41 | 45 | 48 | 1.879983 | 5.09E-07 | 4.76E-06 | up |
| ENSMUS( | Krt8 | 124 | 124 | 85 | 57 | 47 | 66 | 1.840179 | 5.14E-07 | 4.80E-06 | up |
| ENSMUS( | A430105I1 | 195 | 154 | 166 | 90 | 106 | 107 | 1.60753 | 5.17E-07 | 4.82E-06 | up |
| ENSMUS( | Acss3 | 60 | 41 | 59 | 21 | 21 | 15 | 2.654533 | 5.53E-07 | 5.13E-06 | up |
| ENSMUS( | Ifi204 | 53 | 45 | 53 | 10 | 4 | 19 | 4.387662 | 5.70E-07 | 5.27E-06 | up |
| ENSMUS( | Malt1 | 127 | 137 | 125 | 77 | 52 | 71 | 1.839287 | 6.31E-07 | 5.80E-06 | up |
| ENSMUS( | Cep135 | 212 | 235 | 181 | 111 | 137 | 147 | 1.501878 | 6.50E-07 | 5.96E-06 | up |
| ENSMUS( | Ms4a6c | 50 | 53 | 43 | 8 | 16 | 20 | 3.154563 | 6.89E-07 | 6.30E-06 | up |
| ENSMUS( | Glp2r | 27 | 21 | 22 | 4 | 5 | 6 | 4.420536 | 7.54E-07 | 6.86E-06 | up |
| ENSMUS( | Slc24a4 | 310 | 259 | 234 | 162 | 187 | 150 | 1.51187 | 7.61E-07 | 6.91E-06 | up |
| ENSMUS( | Slfn5 | 108 | 117 | 118 | 70 | 59 | 57 | 1.745742 | 7.83E-07 | 7.10E-06 | up |
| ENSMUS( | Uvssa | 180 | 240 | 185 | 110 | 106 | 130 | 1.656325 | 8.23E-07 | 7.45E-06 | up |
| ENSMUS( | Clvs1 | 208 | 167 | 154 | 70 | 117 | 84 | 1.836904 | 8.56E-07 | 7.72E-06 | up |
| ENSMUS( | Ifi47 | 71 | 69 | 48 | 24 | 32 | 27 | 2.123918 | 8.98E-07 | 8.08E-06 | up |
| ENSMUS( | Aldob | 53 | 68 | 56 | 25 | 26 | 25 | 2.203589 | 9.10E-07 | 8.18E-06 | up |
| ENSMUS( | Crb2 | 150 | 134 | 129 | 74 | 80 | 86 | 1.62656 | 9.28E-07 | 8.34E-06 | up |
| ENSMUS( | Mcmdc2 | 12 | 18 | 12 | 1 | 1 | 2 | 9.996907 | 1.13E-06 | 1.00E-05 | up |
| ENSMUS( | Iqcd | 33 | 36 | 35 | 9 | 13 | 11 | 2.991128 | 1.14E-06 | 1.01E-05 | up |
| ENSMUS( | Ifi207 | 34 | 26 | 23 | 7 | 7 | 8 | 3.545869 | 1.21E-06 | 1.07E-05 | up |
| ENSMUS( | Ifit1 | 106 | 138 | 104 | 57 | 39 | 61 | 2.09648 | 1.23E-06 | 1.09E-05 | up |
| ENSMUS( | Dchs2 | 21 | 20 | 23 | 4 | 6 | 3 | 4.658427 | 1.31E-06 | 1.15E-05 | up |
| ENSMUS( | Ston1 | 77 | 68 | 92 | 34 | 39 | 29 | 2.209814 | 1.32E-06 | 1.16E-05 | up |
| ENSMUS( | Sik1 | 211 | 218 | 221 | 96 | 106 | 150 | 1.759895 | 1.36E-06 | 1.20E-05 | up |
| ENSMUS( | Rtp1 | 80 | 63 | 49 | 31 | 28 | 24 | 2.158525 | 1.42E-06 | 1.24E-05 | up |
| ENSMUS( | Fgl2 | 211 | 162 | 161 | 98 | 111 | 110 | 1.577631 | 1.53E-06 | 1.33E-05 | up |
| ENSMUS( | 8430408G | 45 | 43 | 39 | 20 | 12 | 15 | 2.542332 | 1.69E-06 | 1.46E-05 | up |
| ENSMUS( | Irf7 | 69 | 68 | 71 | 32 | 29 | 37 | 2.015878 | 1.69E-06 | 1.46E-05 | up |
| ENSMUS( | Gimap8 | 55 | 40 | 47 | 19 | 17 | 20 | 2.397973 | 1.70E-06 | 1.46E-05 | up |
| ENSMUS( | Gbp7 | 207 | 189 | 152 | 107 | 77 | 126 | 1.665477 | 1.78E-06 | 1.53E-05 | up |
| ENSMUS( | Rab11fip1 | 72 | 88 | 58 | 37 | 29 | 34 | 2.047723 | 1.91E-06 | 1.63E-05 | up |
| ENSMUS( | Tbc1d2 | 118 | 96 | 72 | 34 | 52 | 45 | 2.04672 | 1.91E-06 | 1.64E-05 | up |
| ENSMUS( | Polk | 224 | 267 | 231 | 134 | 157 | 165 | 1.501452 | 1.95E-06 | 1.67E-05 | up |
| ENSMUS( | Cxcl16 | 78 | 78 | 63 | 36 | 33 | 37 | 1.945728 | 2.03E-06 | 1.73E-05 | up |
| ENSMUS( | Tmem184a | 21 | 30 | 18 | 5 | 7 | 4 | 4.041806 | 2.21E-06 | 1.87E-05 | up |
| ENSMUS( | Itpripl1 | 117 | 87 | 89 | 35 | 30 | 60 | 2.223668 | 2.22E-06 | 1.88E-05 | up |
| ENSMUS( | Pmaip1 | 53 | 48 | 48 | 20 | 17 | 24 | 2.314479 | 2.22E-06 | 1.88E-05 | up |
| ENSMUS( | Acvrl1 | 148 | 127 | 134 | 84 | 77 | 81 | 1.597302 | 2.38E-06 | 2.00E-05 | up |
| ENSMUS( | Map3k9 | 145 | 131 | 103 | 71 | 65 | 84 | 1.620844 | 2.43E-06 | 2.05E-05 | up |
| ENSMUS( | Tlr7 | 152 | 137 | 105 | 69 | 77 | 82 | 1.62389 | 2.47E-06 | 2.08E-05 | up |
| ENSMUS( | Traf3ip2 | 98 | 91 | 83 | 45 | 54 | 44 | 1.792435 | 2.49E-06 | 2.09E-05 | up |
| ENSMUS( | Thbs3 | 233 | 213 | 155 | 126 | 118 | 116 | 1.562949 | 2.56E-06 | 2.15E-05 | up |
| ENSMUS( | Fam46a | 194 | 168 | 144 | 116 | 77 | 87 | 1.693974 | 2.87E-06 | 2.39E-05 | up |
| ENSMUS( | Rnf125 | 22 | 15 | 18 | 3 | 3 | 4 | 5.21096 | 2.96E-06 | 2.45E-05 | up |
| ENSMUS( | Gm38394 | 15 | 13 | 17 | 2 | 1 | 3 | 7.189219 | 3.24E-06 | 2.67E-05 | up |
| ENSMUS( | Ifi206 | 19 | 12 | 14 | 1 | 2 | 3 | 7.139695 | 3.30E-06 | 2.72E-05 | up |
| ENSMUS( | Snx29 | 43 | 57 | 52 | 16 | 24 | 23 | 2.298301 | 3.40E-06 | 2.80E-05 | up |
| ENSMUS( | Zbp1 | 48 | 32 | 32 | 13 | 12 | 5 | 3.471279 | 3.46E-06 | 2.85E-05 | up |
| ENSMUS( | Epn3 | 181 | 132 | 109 | 66 | 73 | 84 | 1.775428 | 3.46E-06 | 2.85E-05 | up |
| ENSMUS( | Urb1 | 201 | 184 | 164 | 93 | 130 | 95 | 1.625545 | 3.55E-06 | 2.91E-05 | up |
| ENSMUS( | Lig4 | 220 | 227 | 217 | 125 | 161 | 126 | 1.524313 | 3.91E-06 | 3.19E-05 | up |
| ENSMUS( | Olfr648 | 18 | 25 | 27 | 3 | 7 | 7 | 3.960245 | 3.95E-06 | 3.22E-05 | up |
| ENSMUS( | Hcar2 | 27 | 32 | 35 | 2 | 9 | 4 | 5.979832 | 4.00E-06 | 3.26E-05 | up |
| ENSMUS( | Ms4a6d | 92 | 99 | 90 | 50 | 33 | 54 | 1.943483 | 4.26E-06 | 3.46E-05 | up |
| ENSMUS( | Defb11 | 41 | 49 | 30 | 13 | 18 | 9 | 2.800625 | 4.36E-06 | 3.53E-05 | up |
| ENSMUS( | Pcdhb19 | 130 | 133 | 124 | 73 | 71 | 85 | 1.600912 | 4.44E-06 | 3.59E-05 | up |
| ENSMUS( | Pirb | 33 | 15 | 25 | 7 | 4 | 5 | 4.292409 | 4.58E-06 | 3.69E-05 | up |
| ENSMUS( | Shc3 | 148 | 136 | 158 | 79 | 81 | 92 | 1.668336 | 4.67E-06 | 3.76E-05 | up |
| ENSMUS( | Rbm41 | 115 | 148 | 122 | 45 | 52 | 77 | 2.107396 | 4.69E-06 | 3.78E-05 | up |
| ENSMUS( | Mov10 | 160 | 149 | 145 | 87 | 94 | 99 | 1.534018 | 4.70E-06 | 3.79E-05 | up |
| ENSMUS( | Plcxd3 | 46 | 49 | 62 | 17 | 6 | 11 | 4.390848 | 4.78E-06 | 3.84E-05 | up |
| ENSMUS( | Igsf6 | 42 | 45 | 34 | 16 | 12 | 18 | 2.480252 | 4.94E-06 | 3.97E-05 | up |
| ENSMUS( | Ccr2 | 75 | 66 | 57 | 23 | 14 | 36 | 2.56977 | 5.23E-06 | 4.19E-05 | up |
| ENSMUS( | Stard9 | 120 | 126 | 115 | 56 | 75 | 79 | 1.630613 | 6.01E-06 | 4.76E-05 | up |
| ENSMUS( | Hfm1 | 65 | 48 | 55 | 26 | 19 | 31 | 2.094083 | 6.11E-06 | 4.83E-05 | up |
| ENSMUS( | Rtkn | 169 | 195 | 188 | 98 | 124 | 114 | 1.560314 | 6.33E-06 | 4.99E-05 | up |
| ENSMUS( | Slc24a5 | 109 | 98 | 61 | 44 | 39 | 44 | 1.969647 | 7.25E-06 | 5.67E-05 | up |
| ENSMUS( | Fam205c | 31 | 21 | 29 | 4 | 7 | 1 | 6.329942 | 7.25E-06 | 5.67E-05 | up |
| ENSMUS( | Slc19a3 | 81 | 72 | 55 | 28 | 25 | 42 | 2.064446 | 7.50E-06 | 5.84E-05 | up |
| ENSMUS( | Mfsd7c | 98 | 67 | 57 | 22 | 32 | 38 | 2.268984 | 7.94E-06 | 6.14E-05 | up |
| ENSMUS( | Adamts9 | 229 | 194 | 221 | 140 | 118 | 149 | 1.500547 | 7.96E-06 | 6.15E-05 | up |
| ENSMUS( | Fstl4 | 68 | 66 | 67 | 36 | 29 | 21 | 2.20005 | 8.41E-06 | 6.47E-05 | up |
| ENSMUS( | Eps8l2 | 34 | 35 | 24 | 9 | 9 | 13 | 2.826573 | 9.01E-06 | 6.90E-05 | up |
| ENSMUS( | Zfp563 | 148 | 148 | 137 | 88 | 98 | 82 | 1.524893 | 9.24E-06 | 7.05E-05 | up |
| ENSMUS( | Ptgis | 85 | 88 | 78 | 41 | 39 | 54 | 1.775282 | 9.56E-06 | 7.28E-05 | up |
| ENSMUS( | Gmnc | 226 | 175 | 142 | 109 | 107 | 103 | 1.592963 | 9.73E-06 | 7.39E-05 | up |
| ENSMUS( | Chdh | 100 | 129 | 108 | 58 | 67 | 60 | 1.724793 | 9.86E-06 | 7.48E-05 | up |
| ENSMUS( | Fam161a | 133 | 130 | 104 | 82 | 67 | 66 | 1.602007 | 9.87E-06 | 7.49E-05 | up |
| ENSMUS( | Ppara | 37 | 38 | 26 | 13 | 14 | 9 | 2.619663 | 1.10E-05 | 8.25E-05 | up |
| ENSMUS( | Tmod1 | 170 | 202 | 175 | 110 | 103 | 132 | 1.50398 | 1.15E-05 | 8.65E-05 | up |

| | | | | | | | | | | | |
|---|---|---|---|---|---|---|---|---|---|---|---|
| ENSMUS( | Ehf | 21 | 24 | 16 | 1 | 5 | 2 | 7.167545 | 1.16E-05 | 8.65E-05 | up |
| ENSMUS( | Fyb | 66 | 60 | 60 | 34 | 27 | 29 | 1.950187 | 1.16E-05 | 8.68E-05 | up |
| ENSMUS( | Gm1110 | 27 | 20 | 21 | 5 | 6 | 7 | 3.57223 | 1.16E-05 | 8.69E-05 | up |
| ENSMUS( | Col27a1 | 93 | 96 | 89 | 54 | 51 | 49 | 1.704974 | 1.17E-05 | 8.74E-05 | up |
| ENSMUS( | Mx1 | 17 | 20 | 12 | 1 | 4 | 4 | 5.152062 | 1.20E-05 | 8.94E-05 | up |
| ENSMUS( | Zgrf1 | 99 | 110 | 91 | 53 | 64 | 53 | 1.664839 | 1.20E-05 | 8.94E-05 | up |
| ENSMUS( | Nt5c1a | 43 | 33 | 28 | 11 | 12 | 15 | 2.574743 | 1.22E-05 | 9.11E-05 | up |
| ENSMUS( | Gm3373 | 26 | 28 | 16 | 8 | 7 | 3 | 3.598962 | 1.25E-05 | 9.30E-05 | up |
| ENSMUS( | Farp2 | 107 | 79 | 77 | 37 | 46 | 57 | 1.774444 | 1.33E-05 | 9.80E-05 | up |
| ENSMUS( | L3mbtl1 | 54 | 50 | 37 | 16 | 18 | 27 | 2.181149 | 1.33E-05 | 9.81E-05 | up |
| ENSMUS( | Nhlrc2 | 118 | 130 | 120 | 79 | 63 | 79 | 1.576979 | 1.38E-05 | 0.000101 | up |
| ENSMUS( | Prg4 | 198 | 159 | 137 | 97 | 103 | 103 | 1.53122 | 1.47E-05 | 0.000108 | up |
| ENSMUS( | 9930111J2 | 24 | 24 | 15 | 4 | 8 | 4 | 3.675758 | 1.62E-05 | 0.000118 | up |
| ENSMUS( | St8sia6 | 142 | 156 | 136 | 91 | 91 | 64 | 1.660568 | 1.68E-05 | 0.000122 | up |
| ENSMUS( | Robo3 | 143 | 164 | 119 | 86 | 87 | 86 | 1.547817 | 1.69E-05 | 0.000123 | up |
| ENSMUS( | Apol11b | 7 | 7 | 13 | 0 | 1 | 0 | 25.88534 | 1.76E-05 | 0.000127 | up |
| ENSMUS( | Gimap4 | 52 | 38 | 37 | 17 | 19 | 17 | 2.251502 | 1.79E-05 | 0.00013 | up |
| ENSMUS( | Tstd2 | 134 | 140 | 134 | 77 | 79 | 97 | 1.530782 | 1.81E-05 | 0.000131 | up |
| ENSMUS( | Atp10a | 125 | 147 | 123 | 75 | 86 | 53 | 1.737454 | 1.84E-05 | 0.000133 | up |
| ENSMUS( | Tat | 5 | 23 | 4 | 1 | 0 | 0 | 28.92892 | 1.85E-05 | 0.000133 | up |
| ENSMUS( | Ifi211 | 17 | 17 | 16 | 3 | 5 | 2 | 4.702838 | 1.87E-05 | 0.000134 | up |
| ENSMUS( | Coro6 | 140 | 148 | 130 | 83 | 84 | 94 | 1.514891 | 1.90E-05 | 0.000136 | up |
| ENSMUS( | Hyal1 | 68 | 51 | 43 | 25 | 28 | 22 | 2.019844 | 1.94E-05 | 0.000139 | up |
| ENSMUS( | Pm20d2 | 50 | 35 | 32 | 14 | 12 | 21 | 2.346727 | 1.98E-05 | 0.000141 | up |
| ENSMUS( | Onecut2 | 124 | 130 | 103 | 80 | 62 | 62 | 1.64362 | 2.00E-05 | 0.000143 | up |
| ENSMUS( | Chrm5 | 34 | 37 | 10 | 6 | 9 | 4 | 3.909551 | 2.00E-05 | 0.000143 | up |
| ENSMUS( | Gm14412 | 35 | 22 | 26 | 10 | 11 | 5 | 2.984808 | 2.03E-05 | 0.000144 | up |
| ENSMUS( | Brca2 | 145 | 153 | 129 | 83 | 102 | 83 | 1.502327 | 2.08E-05 | 0.000148 | up |
| ENSMUS( | Large2 | 25 | 32 | 20 | 4 | 4 | 10 | 4.067533 | 2.14E-05 | 0.000151 | up |
| ENSMUS( | Slfn2 | 98 | 91 | 61 | 41 | 45 | 29 | 2.025277 | 2.26E-05 | 0.00016 | up |
| ENSMUS( | Cd14 | 140 | 137 | 129 | 72 | 90 | 91 | 1.519664 | 2.32E-05 | 0.000163 | up |
| ENSMUS( | Polq | 87 | 83 | 67 | 47 | 42 | 39 | 1.737659 | 2.34E-05 | 0.000165 | up |
| ENSMUS( | Parp9 | 131 | 135 | 152 | 78 | 77 | 87 | 1.64462 | 2.37E-05 | 0.000167 | up |
| ENSMUS( | Phyhd1 | 24 | 31 | 29 | 11 | 10 | 7 | 2.835418 | 2.47E-05 | 0.000173 | up |
| ENSMUS( | Cgn | 49 | 62 | 44 | 21 | 22 | 29 | 2.036403 | 2.50E-05 | 0.000175 | up |
| ENSMUS( | Vgll3 | 80 | 85 | 73 | 42 | 42 | 45 | 1.743078 | 2.52E-05 | 0.000176 | up |
| ENSMUS( | Zfp458 | 82 | 87 | 73 | 48 | 40 | 44 | 1.728283 | 2.61E-05 | 0.000183 | up |
| ENSMUS( | Wdr72 | 37 | 25 | 27 | 9 | 14 | 8 | 2.695219 | 2.66E-05 | 0.000186 | up |
| ENSMUS( | Gm14403 | 115 | 112 | 112 | 62 | 71 | 70 | 1.582232 | 2.70E-05 | 0.000189 | up |
| ENSMUS( | Socs3 | 57 | 55 | 56 | 24 | 25 | 32 | 1.970461 | 2.79E-05 | 0.000194 | up |
| ENSMUS( | Zfp276 | 131 | 153 | 140 | 93 | 74 | 95 | 1.534401 | 2.88E-05 | 0.0002 | up |
| ENSMUS( | Zfp526 | 96 | 84 | 85 | 43 | 52 | 54 | 1.683797 | 2.93E-05 | 0.000203 | up |
| ENSMUS( | Ddb2 | 136 | 126 | 117 | 86 | 59 | 69 | 1.667456 | 2.98E-05 | 0.000206 | up |
| ENSMUS( | 4933428G | 21 | 18 | 16 | 6 | 4 | 3 | 3.951105 | 3.04E-05 | 0.00021 | up |
| ENSMUS( | Olfr1507 | 16 | 10 | 11 | 1 | 1 | 3 | 7.056252 | 3.10E-05 | 0.000214 | up |
| ENSMUS( | Cxcl9 | 33 | 27 | 30 | 12 | 10 | 10 | 2.655139 | 3.23E-05 | 0.000222 | up |
| ENSMUS( | Erg | 64 | 53 | 56 | 33 | 20 | 24 | 2.115854 | 3.39E-05 | 0.000232 | up |
| ENSMUS( | Ifi209 | 16 | 30 | 22 | 4 | 6 | 8 | 3.612264 | 3.39E-05 | 0.000232 | up |
| ENSMUS( | Mmrn2 | 74 | 83 | 91 | 38 | 44 | 46 | 1.847515 | 3.39E-05 | 0.000232 | up |
| ENSMUS( | Zfp382 | 70 | 91 | 42 | 26 | 28 | 34 | 2.159081 | 3.45E-05 | 0.000236 | up |
| ENSMUS( | Uba7 | 53 | 59 | 47 | 22 | 22 | 32 | 1.982083 | 3.74E-05 | 0.000254 | up |
| ENSMUS( | Zfp366 | 18 | 17 | 13 | 3 | 3 | 4 | 4.522771 | 3.88E-05 | 0.000263 | up |
| ENSMUS( | Wdr78 | 165 | 205 | 152 | 101 | 103 | 121 | 1.51793 | 3.92E-05 | 0.000266 | up |
| ENSMUS( | Cxcl1 | 38 | 33 | 23 | 3 | 2 | 12 | 5.273657 | 3.93E-05 | 0.000266 | up |
| ENSMUS( | Prkg1 | 102 | 101 | 102 | 45 | 64 | 66 | 1.656471 | 3.98E-05 | 0.00027 | up |
| ENSMUS( | Cfp | 150 | 130 | 111 | 70 | 83 | 92 | 1.504403 | 4.00E-05 | 0.00027 | up |
| ENSMUS( | Gm16432 | 158 | 160 | 170 | 75 | 88 | 115 | 1.673816 | 4.05E-05 | 0.000274 | up |
| ENSMUS( | Fzd3 | 132 | 110 | 128 | 44 | 63 | 81 | 1.876818 | 4.07E-05 | 0.000275 | up |
| ENSMUS( | Plek2 | 40 | 39 | 23 | 15 | 14 | 10 | 2.4303 | 4.12E-05 | 0.000278 | up |
| ENSMUS( | Slfn1 | 18 | 18 | 16 | 4 | 3 | 5 | 4.104309 | 4.18E-05 | 0.000282 | up |
| ENSMUS( | Schip1 | 103 | 95 | 92 | 58 | 59 | 52 | 1.618516 | 4.23E-05 | 0.000285 | up |
| ENSMUS( | Cntnap3 | 115 | 129 | 110 | 66 | 81 | 70 | 1.541576 | 4.30E-05 | 0.000289 | up |
| ENSMUS( | Tcaf2 | 39 | 39 | 34 | 13 | 17 | 16 | 2.300646 | 4.38E-05 | 0.000294 | up |
| ENSMUS( | Pcdhga5 | 82 | 65 | 72 | 36 | 38 | 44 | 1.758046 | 4.44E-05 | 0.000298 | up |
| ENSMUS( | Kcna3 | 18 | 19 | 27 | 0 | 4 | 3 | 8.863515 | 4.63E-05 | 0.000309 | up |
| ENSMUS( | Nostrin | 75 | 60 | 58 | 39 | 30 | 31 | 1.813014 | 4.68E-05 | 0.000312 | up |
| ENSMUS( | D16Ertd47 | 101 | 93 | 80 | 48 | 48 | 62 | 1.637649 | 4.79E-05 | 0.00032 | up |
| ENSMUS( | Naaladl2 | 85 | 76 | 67 | 40 | 44 | 41 | 1.717163 | 5.07E-05 | 0.000337 | up |
| ENSMUS( | Gm4951 | 34 | 26 | 23 | 11 | 9 | 9 | 2.680086 | 5.26E-05 | 0.000348 | up |
| ENSMUS( | Ak9 | 62 | 40 | 46 | 24 | 23 | 20 | 2.075679 | 5.29E-05 | 0.00035 | up |
| ENSMUS( | Cd274 | 78 | 77 | 71 | 45 | 42 | 37 | 1.717633 | 5.63E-05 | 0.000371 | up |
| ENSMUS( | Ms4a4a | 21 | 14 | 27 | 5 | 2 | 7 | 4.269449 | 5.75E-05 | 0.000379 | up |
| ENSMUS( | Best3 | 19 | 17 | 23 | 8 | 4 | 4 | 3.48975 | 5.77E-05 | 0.00038 | up |
| ENSMUS( | Sytl3 | 29 | 25 | 10 | 7 | 4 | 4 | 3.918974 | 5.81E-05 | 0.000382 | up |
| ENSMUS( | Pcdhgc4 | 86 | 111 | 80 | 29 | 54 | 45 | 2.047128 | 5.85E-05 | 0.000384 | up |
| ENSMUS( | Cd4 | 17 | 18 | 16 | 6 | 3 | 3 | 3.986142 | 5.86E-05 | 0.000385 | up |
| ENSMUS( | Gm3558 | 28 | 41 | 24 | 14 | 9 | 10 | 2.640123 | 5.98E-05 | 0.000392 | up |
| ENSMUS( | Igfn1 | 40 | 18 | 42 | 9 | 11 | 12 | 2.989899 | 6.14E-05 | 0.000402 | up |
| ENSMUS( | Styx | 32 | 41 | 39 | 19 | 13 | 15 | 2.259127 | 6.36E-05 | 0.000414 | up |
| ENSMUS( | Cep152 | 104 | 88 | 84 | 50 | 50 | 61 | 1.619465 | 6.51E-05 | 0.000423 | up |

| | | | | | | | | | | | |
|---|---|---|---|---|---|---|---|---|---|---|---|
| ENSMUS( | Csf2rb2 | 44 | 51 | 46 | 18 | 26 | 22 | 2.025666 | 6.79E-05 | 0.00044 | up |
| ENSMUS( | Plekhh2 | 160 | 139 | 126 | 68 | 102 | 86 | 1.565635 | 6.82E-05 | 0.000442 | up |
| ENSMUS( | Ifi213 | 34 | 38 | 15 | 8 | 3 | 3 | 5.705371 | 6.90E-05 | 0.000446 | up |
| ENSMUS( | Mylk2 | 16 | 15 | 3 | 2 | 0 | 1 | 10.30428 | 7.00E-05 | 0.000452 | up |
| ENSMUS( | Ttc21a | 65 | 59 | 60 | 37 | 23 | 27 | 1.993488 | 7.34E-05 | 0.000473 | up |
| ENSMUS( | Zfp457 | 29 | 24 | 27 | 10 | 6 | 12 | 2.713615 | 7.38E-05 | 0.000474 | up |
| ENSMUS( | Hgf | 77 | 84 | 85 | 54 | 39 | 44 | 1.700656 | 7.44E-05 | 0.000478 | up |
| ENSMUS( | Apobec3 | 114 | 131 | 97 | 74 | 68 | 47 | 1.695435 | 7.61E-05 | 0.000488 | up |
| ENSMUS( | Il4ra | 107 | 105 | 92 | 48 | 67 | 67 | 1.579988 | 7.63E-05 | 0.000489 | up |
| ENSMUS( | Crybg3 | 105 | 95 | 87 | 56 | 53 | 61 | 1.593202 | 7.71E-05 | 0.000494 | up |
| ENSMUS( | C1s1 | 36 | 43 | 25 | 18 | 10 | 14 | 2.314086 | 7.76E-05 | 0.000497 | up |
| ENSMUS( | Alpk1 | 74 | 100 | 81 | 53 | 39 | 41 | 1.81049 | 7.78E-05 | 0.000498 | up |
| ENSMUS( | Gm9821 | 19 | 19 | 11 | 2 | 1 | 5 | 5.801224 | 7.94E-05 | 0.000508 | up |
| ENSMUS( | Mtcp1 | 72 | 67 | 68 | 33 | 40 | 39 | 1.750903 | 7.95E-05 | 0.000508 | up |
| ENSMUS( | Zfp955b | 126 | 116 | 115 | 80 | 71 | 72 | 1.511386 | 8.00E-05 | 0.000511 | up |
| ENSMUS( | Inmt | 65 | 59 | 40 | 25 | 31 | 25 | 1.892573 | 8.09E-05 | 0.000516 | up |
| ENSMUS( | Pcdhgb6 | 57 | 50 | 45 | 24 | 25 | 25 | 1.934702 | 8.18E-05 | 0.000521 | up |
| ENSMUS( | Cdc42bpg | 117 | 97 | 110 | 57 | 55 | 79 | 1.612671 | 8.32E-05 | 0.000529 | up |
| ENSMUS( | Tbc1d4 | 183 | 160 | 90 | 79 | 78 | 87 | 1.653223 | 8.54E-05 | 0.000542 | up |
| ENSMUS( | Muc15 | 22 | 21 | 16 | 4 | 6 | 1 | 4.980598 | 8.54E-05 | 0.000542 | up |
| ENSMUS( | Ly6c2 | 81 | 67 | 88 | 39 | 40 | 44 | 1.826916 | 9.04E-05 | 0.000571 | up |
| ENSMUS( | Col14a1 | 38 | 27 | 33 | 9 | 6 | 16 | 3.015508 | 9.07E-05 | 0.000573 | up |
| ENSMUS( | Usp51 | 28 | 28 | 35 | 11 | 10 | 14 | 2.486732 | 9.16E-05 | 0.000578 | up |
| ENSMUS( | Zfp979 | 86 | 92 | 75 | 41 | 47 | 58 | 1.640145 | 9.41E-05 | 0.000593 | up |
| ENSMUS( | Pla2g2f | 39 | 38 | 47 | 22 | 16 | 17 | 2.141858 | 9.73E-05 | 0.000611 | up |
| ENSMUS( | Catsperg2 | 10 | 11 | 8 | 1 | 1 | 1 | 9.090396 | 9.75E-05 | 0.000612 | up |
| ENSMUS( | C1rb | 11 | 13 | 17 | 2 | 4 | 2 | 4.900448 | 9.98E-05 | 0.000626 | up |
| ENSMUS( | Cyb561a3 | 119 | 102 | 100 | 60 | 66 | 71 | 1.539884 | 0.000101 | 0.000631 | up |
| ENSMUS( | Tex9 | 46 | 57 | 39 | 23 | 27 | 18 | 1.95894 | 0.000101 | 0.000631 | up |
| ENSMUS( | Ier3 | 118 | 108 | 115 | 77 | 72 | 60 | 1.540655 | 0.000101 | 0.000632 | up |
| ENSMUS( | Adamts6 | 50 | 49 | 40 | 18 | 27 | 16 | 2.139889 | 0.000104 | 0.000647 | up |
| ENSMUS( | Cd8b1 | 6 | 16 | 11 | 0 | 2 | 2 | 7.971968 | 0.000108 | 0.000673 | up |
| ENSMUS( | 2700049A | 158 | 145 | 137 | 67 | 112 | 80 | 1.604208 | 0.000112 | 0.000695 | up |
| ENSMUS( | Fam198a | 109 | 107 | 121 | 73 | 63 | 64 | 1.598693 | 0.000113 | 0.0007 | up |
| ENSMUS( | Zfp433 | 27 | 29 | 19 | 7 | 9 | 10 | 2.714158 | 0.000113 | 0.000703 | up |
| ENSMUS( | Il18bp | 102 | 76 | 72 | 49 | 44 | 50 | 1.643359 | 0.000114 | 0.000708 | up |
| ENSMUS( | Fas | 63 | 75 | 71 | 36 | 45 | 34 | 1.722123 | 0.000115 | 0.000711 | up |
| ENSMUS( | Amer1 | 147 | 144 | 160 | 96 | 91 | 97 | 1.508147 | 0.000117 | 0.000725 | up |
| ENSMUS( | 1700024P1 | 76 | 83 | 65 | 44 | 44 | 38 | 1.671962 | 0.000119 | 0.000733 | up |
| ENSMUS( | Col6a3 | 113 | 84 | 68 | 43 | 37 | 57 | 1.816937 | 0.000127 | 0.000779 | up |
| ENSMUS( | Corin | 46 | 31 | 25 | 14 | 7 | 5 | 3.623306 | 0.000127 | 0.000781 | up |
| ENSMUS( | Dclre1c | 202 | 167 | 185 | 76 | 105 | 140 | 1.643404 | 0.00013 | 0.000798 | up |
| ENSMUS( | Caprin2 | 144 | 154 | 124 | 77 | 70 | 109 | 1.560911 | 0.000135 | 0.000826 | up |
| ENSMUS( | Cntn5 | 60 | 64 | 67 | 30 | 31 | 42 | 1.767967 | 0.000138 | 0.000839 | up |
| ENSMUS( | Lrrc9 | 40 | 36 | 34 | 17 | 14 | 17 | 2.162652 | 0.000141 | 0.000858 | up |
| ENSMUS( | Gm5431 | 34 | 36 | 27 | 14 | 14 | 12 | 2.276481 | 0.000153 | 0.000925 | up |
| ENSMUS( | Sfxn2 | 61 | 50 | 58 | 32 | 22 | 34 | 1.821659 | 0.000154 | 0.000928 | up |
| ENSMUS( | Galnt15 | 64 | 68 | 40 | 24 | 25 | 35 | 1.923162 | 0.000163 | 0.000978 | up |
| ENSMUS( | Ccdc170 | 32 | 26 | 36 | 11 | 12 | 15 | 2.363519 | 0.000167 | 0.001001 | up |
| ENSMUS( | Zfp90 | 44 | 37 | 25 | 19 | 13 | 14 | 2.145071 | 0.000171 | 0.001022 | up |
| ENSMUS( | Gpr141 | 11 | 20 | 19 | 0 | 3 | 1 | 11.9943 | 0.000174 | 0.001037 | up |
| ENSMUS( | Gzmb | 17 | 22 | 13 | 2 | 5 | 7 | 3.525389 | 0.000174 | 0.001037 | up |
| ENSMUS( | Ngp | 70 | 53 | 37 | 19 | 24 | 31 | 2.027147 | 0.000175 | 0.001042 | up |
| ENSMUS( | Zfp599 | 112 | 119 | 96 | 74 | 63 | 68 | 1.502132 | 0.000178 | 0.001059 | up |
| ENSMUS( | Pcdhac1 | 31 | 22 | 22 | 9 | 7 | 11 | 2.619278 | 0.000179 | 0.001064 | up |
| ENSMUS( | Tjp3 | 63 | 54 | 48 | 25 | 30 | 31 | 1.808766 | 0.000186 | 0.001101 | up |
| ENSMUS( | Plag1 | 38 | 49 | 51 | 13 | 25 | 17 | 2.393313 | 0.000187 | 0.001104 | up |
| ENSMUS( | Bmp5 | 29 | 48 | 35 | 16 | 13 | 17 | 2.309125 | 0.000193 | 0.001136 | up |
| ENSMUS( | Lilrb4a | 21 | 23 | 13 | 5 | 8 | 4 | 3.122805 | 0.000195 | 0.00115 | up |
| ENSMUS( | Sp100 | 77 | 87 | 75 | 38 | 54 | 34 | 1.789285 | 0.000204 | 0.001197 | up |
| ENSMUS( | Spn | 34 | 23 | 33 | 11 | 13 | 12 | 2.373927 | 0.00021 | 0.001228 | up |
| ENSMUS( | Otogl | 66 | 51 | 50 | 20 | 35 | 26 | 1.943245 | 0.000212 | 0.001241 | up |
| ENSMUS( | Csf2rb | 52 | 46 | 65 | 31 | 22 | 24 | 2.015317 | 0.000227 | 0.00132 | up |
| ENSMUS( | Mum1l1 | 156 | 158 | 126 | 85 | 111 | 79 | 1.503722 | 0.000227 | 0.001322 | up |
| ENSMUS( | Cdh3 | 37 | 22 | 40 | 8 | 13 | 16 | 2.567985 | 0.000239 | 0.001387 | up |
| ENSMUS( | Nes | 73 | 65 | 55 | 38 | 30 | 39 | 1.697364 | 0.000245 | 0.001416 | up |
| ENSMUS( | Cpxm2 | 80 | 82 | 61 | 40 | 42 | 47 | 1.627416 | 0.000255 | 0.001471 | up |
| ENSMUS( | Gramd1c | 102 | 92 | 81 | 57 | 63 | 47 | 1.546628 | 0.000276 | 0.001582 | up |
| ENSMUS( | Rnf207 | 69 | 70 | 54 | 24 | 41 | 36 | 1.802654 | 0.00028 | 0.001602 | up |
| ENSMUS( | Shroom4 | 104 | 86 | 94 | 60 | 61 | 54 | 1.53253 | 0.000286 | 0.001635 | up |
| ENSMUS( | Sema3f | 96 | 79 | 98 | 56 | 42 | 60 | 1.642299 | 0.000289 | 0.00165 | up |
| ENSMUS( | Slc25a13 | 99 | 102 | 76 | 63 | 51 | 56 | 1.529772 | 0.000296 | 0.001685 | up |
| ENSMUS( | Sema3g | 61 | 61 | 57 | 27 | 34 | 37 | 1.731776 | 0.000311 | 0.001767 | up |
| ENSMUS( | Serpina3f | 13 | 18 | 15 | 4 | 5 | 3 | 3.620478 | 0.000312 | 0.001771 | up |
| ENSMUS( | Myh7 | 13 | 20 | 13 | 2 | 6 | 4 | 3.629361 | 0.000314 | 0.001778 | up |
| ENSMUS( | Pcdha7 | 13 | 14 | 17 | 3 | 4 | 4 | 3.825747 | 0.000315 | 0.001785 | up |
| ENSMUS( | Sec14l5 | 67 | 44 | 44 | 8 | 15 | 30 | 2.783432 | 0.000336 | 0.001896 | up |
| ENSMUS( | Slc10a6 | 7 | 11 | 13 | 1 | 1 | 3 | 6.038228 | 0.000342 | 0.001927 | up |
| ENSMUS( | Slc22a18 | 35 | 41 | 40 | 19 | 19 | 17 | 1.999356 | 0.000376 | 0.0021 | up |
| ENSMUS( | Mansc1 | 136 | 98 | 104 | 71 | 64 | 74 | 1.524302 | 0.000384 | 0.00214 | up |

| | | | | | | | | | | | | |
|---|---|---|---|---|---|---|---|---|---|---|---|---|
| ENSMUS( | Mpg | 123 | 121 | 85 | 78 | 60 | 64 | 1.52406 | 0.00039 | 0.002171 | up |
| ENSMUS( | Madcam1 | 24 | 14 | 10 | 4 | 3 | 5 | 3.724981 | 0.000411 | 0.002274 | up |
| ENSMUS( | Cklf | 87 | 85 | 93 | 58 | 54 | 51 | 1.540666 | 0.000434 | 0.002388 | up |
| ENSMUS( | Nbeal2 | 112 | 116 | 95 | 71 | 49 | 75 | 1.563159 | 0.000436 | 0.002397 | up |
| ENSMUS( | Loxl4 | 15 | 14 | 8 | 3 | 1 | 4 | 4.337376 | 0.000441 | 0.002424 | up |
| ENSMUS( | Acap1 | 18 | 11 | 14 | 2 | 5 | 4 | 3.704578 | 0.000444 | 0.002436 | up |
| ENSMUS( | Gm4070 | 14 | 12 | 10 | 2 | 3 | 0 | 6.655737 | 0.000447 | 0.002452 | up |
| ENSMUS( | Gbp5 | 135 | 113 | 66 | 53 | 55 | 36 | 2.020105 | 0.00046 | 0.002514 | up |
| ENSMUS( | Caps2 | 45 | 56 | 53 | 30 | 26 | 26 | 1.780598 | 0.000476 | 0.002589 | up |
| ENSMUS( | Trmt13 | 96 | 83 | 69 | 43 | 53 | 55 | 1.546293 | 0.000485 | 0.002638 | up |
| ENSMUS( | Gdpd3 | 67 | 51 | 54 | 28 | 27 | 40 | 1.715205 | 0.00049 | 0.002659 | up |
| ENSMUS( | Lilr4b | 16 | 17 | 19 | 3 | 8 | 5 | 3.098894 | 0.000501 | 0.002711 | up |
| ENSMUS( | Pyroxd1 | 80 | 116 | 76 | 53 | 32 | 40 | 2.04548 | 0.000542 | 0.002909 | up |
| ENSMUS( | Tyms | 101 | 103 | 111 | 65 | 72 | 62 | 1.501616 | 0.000549 | 0.00294 | up |
| ENSMUS( | Ngfr | 40 | 36 | 32 | 19 | 16 | 16 | 1.990744 | 0.000572 | 0.003053 | up |
| ENSMUS( | Map3k14 | 76 | 72 | 60 | 44 | 26 | 42 | 1.748424 | 0.000584 | 0.003108 | up |
| ENSMUS( | Mak | 67 | 60 | 48 | 25 | 36 | 37 | 1.682842 | 0.000587 | 0.003122 | up |
| ENSMUS( | Mecom | 66 | 53 | 60 | 33 | 33 | 35 | 1.677634 | 0.000608 | 0.00322 | up |
| ENSMUS( | Pnp2 | 8 | 7 | 4 | 0 | 0 | 1 | 18.3516 | 0.000613 | 0.003239 | up |
| ENSMUS( | Oas1g | 10 | 22 | 5 | 2 | 1 | 3 | 5.763325 | 0.000613 | 0.003241 | up |
| ENSMUS( | Usp18 | 55 | 27 | 25 | 15 | 12 | 15 | 2.370768 | 0.000622 | 0.003285 | up |
| ENSMUS( | Adamts14 | 36 | 37 | 24 | 14 | 18 | 12 | 2.059773 | 0.000623 | 0.00329 | up |
| ENSMUS( | C1rl | 16 | 10 | 12 | 2 | 2 | 5 | 4.026328 | 0.000632 | 0.003335 | up |
| ENSMUS( | Adamtsl1 | 141 | 95 | 142 | 66 | 81 | 81 | 1.57822 | 0.000637 | 0.003357 | up |
| ENSMUS( | Tert | 19 | 18 | 16 | 7 | 3 | 7 | 2.943754 | 0.000637 | 0.003357 | up |
| ENSMUS( | Ubap1l | 18 | 32 | 13 | 8 | 7 | 4 | 3.077726 | 0.000645 | 0.003392 | up |
| ENSMUS( | Dnah11 | 54 | 59 | 46 | 30 | 34 | 23 | 1.716035 | 0.000666 | 0.003491 | up |
| ENSMUS( | Ripk4 | 31 | 41 | 47 | 23 | 15 | 16 | 2.098948 | 0.000693 | 0.00362 | up |
| ENSMUS( | Nlrp1a | 19 | 14 | 10 | 1 | 5 | 2 | 5.017592 | 0.000697 | 0.003639 | up |
| ENSMUS( | Frrs1 | 79 | 90 | 71 | 50 | 41 | 56 | 1.542609 | 0.000703 | 0.003664 | up |
| ENSMUS( | Pcdhb16 | 123 | 147 | 115 | 57 | 91 | 90 | 1.532771 | 0.000714 | 0.003716 | up |
| ENSMUS( | D930048N | 51 | 43 | 36 | 27 | 19 | 16 | 1.957753 | 0.000733 | 0.003808 | up |
| ENSMUS( | Cntnap5c | 62 | 44 | 66 | 28 | 26 | 34 | 1.864429 | 0.000749 | 0.003882 | up |
| ENSMUS( | Ceacam1 | 120 | 83 | 58 | 45 | 47 | 31 | 1.966659 | 0.000753 | 0.003897 | up |
| ENSMUS( | Nek5 | 74 | 44 | 50 | 16 | 22 | 33 | 2.24114 | 0.000753 | 0.003897 | up |
| ENSMUS( | Milr1 | 22 | 11 | 19 | 5 | 4 | 7 | 3.094082 | 0.000772 | 0.003986 | up |
| ENSMUS( | Gm14137 | 11 | 18 | 19 | 6 | 6 | 2 | 3.247932 | 0.000774 | 0.003994 | up |
| ENSMUS( | Hist2h4 | 14 | 13 | 16 | 4 | 3 | 5 | 3.421218 | 0.000823 | 0.004231 | up |
| ENSMUS( | Wdhd1 | 93 | 76 | 83 | 48 | 56 | 53 | 1.518496 | 0.000827 | 0.004247 | up |
| ENSMUS( | Armc3 | 91 | 70 | 75 | 54 | 45 | 34 | 1.66583 | 0.000843 | 0.004324 | up |
| ENSMUS( | Oas1a | 30 | 27 | 26 | 12 | 8 | 16 | 2.186506 | 0.000899 | 0.004594 | up |
| ENSMUS( | Ghsr | 45 | 51 | 47 | 24 | 21 | 32 | 1.765946 | 0.000929 | 0.004736 | up |
| ENSMUS( | Dnah6 | 181 | 145 | 167 | 120 | 92 | 87 | 1.555135 | 0.000953 | 0.004845 | up |
| ENSMUS( | Nkain3 | 78 | 69 | 52 | 32 | 42 | 44 | 1.585516 | 0.000956 | 0.004855 | up |
| ENSMUS( | Gimap3 | 26 | 22 | 17 | 2 | 9 | 1 | 5.039964 | 0.000957 | 0.004863 | up |
| ENSMUS( | Myo5c | 76 | 77 | 35 | 21 | 21 | 36 | 2.254334 | 0.000991 | 0.00502 | up |
| ENSMUS( | Il1r2 | 16 | 20 | 10 | 3 | 6 | 5 | 3.080427 | 0.001031 | 0.005195 | up |
| ENSMUS( | Kif27 | 44 | 63 | 67 | 28 | 25 | 37 | 1.853836 | 0.001054 | 0.005297 | up |
| ENSMUS( | Spag16 | 115 | 105 | 91 | 70 | 42 | 74 | 1.576796 | 0.001114 | 0.005565 | up |
| ENSMUS( | Dysf | 41 | 52 | 39 | 27 | 24 | 19 | 1.772606 | 0.001127 | 0.005624 | up |
| ENSMUS( | Ifi27l2a | 69 | 71 | 48 | 33 | 25 | 45 | 1.719232 | 0.001138 | 0.005671 | up |
| ENSMUS( | Cabp4 | 6 | 3 | 9 | 0 | 1 | 0 | 17.24732 | 0.001145 | 0.005701 | up |
| ENSMUS( | Ppl | 54 | 43 | 46 | 21 | 24 | 33 | 1.740239 | 0.001174 | 0.005834 | up |
| ENSMUS( | Marveld3 | 9 | 26 | 9 | 2 | 3 | 5 | 4.167648 | 0.001174 | 0.005834 | up |
| ENSMUS( | Thbs1 | 84 | 82 | 80 | 60 | 40 | 39 | 1.666318 | 0.001177 | 0.005845 | up |
| ENSMUS( | Gzmm | 31 | 30 | 33 | 10 | 17 | 17 | 2.036818 | 0.001177 | 0.005845 | up |
| ENSMUS( | Dnase1 | 51 | 46 | 43 | 17 | 31 | 21 | 1.914846 | 0.001197 | 0.005939 | up |
| ENSMUS( | Fgd5 | 89 | 104 | 79 | 67 | 45 | 51 | 1.56869 | 0.001258 | 0.006206 | up |
| ENSMUS( | Pask | 44 | 48 | 36 | 25 | 23 | 20 | 1.76693 | 0.00138 | 0.006751 | up |
| ENSMUS( | Pcdhga11 | 25 | 32 | 24 | 14 | 10 | 12 | 2.120882 | 0.001409 | 0.006878 | up |
| ENSMUS( | Gnmt | 23 | 33 | 25 | 8 | 15 | 13 | 2.136334 | 0.001409 | 0.006878 | up |
| ENSMUS( | Pcdha9 | 22 | 34 | 16 | 10 | 8 | 9 | 2.493871 | 0.00142 | 0.00693 | up |
| ENSMUS( | D130040H | 70 | 55 | 44 | 22 | 39 | 29 | 1.760996 | 0.001451 | 0.007072 | up |
| ENSMUS( | Pcdhgb4 | 44 | 50 | 42 | 28 | 14 | 24 | 1.945058 | 0.001468 | 0.007142 | up |
| ENSMUS( | Ccr1 | 78 | 79 | 73 | 31 | 40 | 59 | 1.684327 | 0.001543 | 0.00747 | up |
| ENSMUS( | Arhgap4 | 79 | 85 | 62 | 43 | 42 | 56 | 1.511899 | 0.00155 | 0.0075 | up |
| ENSMUS( | Birc3 | 71 | 62 | 44 | 21 | 27 | 43 | 1.834988 | 0.001552 | 0.007506 | up |
| ENSMUS( | Gm28729 | 24 | 29 | 38 | 10 | 13 | 16 | 2.247377 | 0.001555 | 0.007516 | up |
| ENSMUS( | Setdb2 | 113 | 113 | 123 | 87 | 59 | 60 | 1.602123 | 0.001558 | 0.007531 | up |
| ENSMUS( | Syne3 | 25 | 33 | 24 | 14 | 13 | 10 | 2.0833 | 0.001584 | 0.007643 | up |
| ENSMUS( | Gm17655 | 37 | 38 | 35 | 22 | 16 | 18 | 1.85319 | 0.001615 | 0.007779 | up |
| ENSMUS( | Ltf | 33 | 30 | 13 | 10 | 8 | 11 | 2.431332 | 0.001723 | 0.008252 | up |
| ENSMUS( | Tead4 | 10 | 10 | 15 | 3 | 3 | 3 | 3.728347 | 0.001729 | 0.008276 | up |
| ENSMUS( | Ciita | 13 | 9 | 7 | 3 | 2 | 1 | 4.461504 | 0.001746 | 0.008347 | up |
| ENSMUS( | Vwa5b1 | 74 | 73 | 78 | 50 | 50 | 41 | 1.510059 | 0.00181 | 0.008623 | up |
| ENSMUS( | Fbxo48 | 5 | 16 | 8 | 3 | 1 | 1 | 5.424274 | 0.001841 | 0.008762 | up |
| ENSMUS( | Ccdc187 | 59 | 81 | 65 | 24 | 47 | 31 | 1.904545 | 0.001859 | 0.008838 | up |
| ENSMUS( | Plbd1 | 38 | 35 | 29 | 20 | 14 | 17 | 1.878196 | 0.001881 | 0.008934 | up |
| ENSMUS( | Gm3526 | 6 | 7 | 9 | 2 | 0 | 1 | 6.966029 | 0.001889 | 0.008963 | up |
| ENSMUS( | Fpr2 | 15 | 11 | 9 | 1 | 3 | 5 | 3.69217 | 0.001937 | 0.009167 | up |

| | | | | | | | | | | | |
|---|---|---|---|---|---|---|---|---|---|---|---|
| ENSMUS( | Itpr3 | 71 | 60 | 57 | 40 | 42 | 29 | 1.58987 | 0.001977 | 0.009337 | up |
| ENSMUS( | Chaf1a | 41 | 38 | 37 | 16 | 18 | 27 | 1.807909 | 0.001993 | 0.00941 | up |
| ENSMUS( | Efhc1 | 68 | 79 | 67 | 46 | 48 | 32 | 1.59886 | 0.001995 | 0.009416 | up |
| ENSMUS( | Cxcl13 | 24 | 20 | 16 | 9 | 2 | 7 | 3.12081 | 0.002038 | 0.009603 | up |
| ENSMUS( | H2-DMb2 | 28 | 39 | 38 | 18 | 17 | 17 | 1.921699 | 0.002071 | 0.009744 | up |
| ENSMUS( | Cldn9 | 29 | 35 | 33 | 10 | 20 | 9 | 2.353572 | 0.002086 | 0.009806 | up |
| ENSMUS( | C5ar1 | 18 | 24 | 20 | 6 | 11 | 8 | 2.352747 | 0.002125 | 0.009971 | up |
| ENSMUS( | Gbp6 | 44 | 37 | 36 | 21 | 25 | 16 | 1.773046 | 0.002153 | 0.010089 | up |
| ENSMUS( | Krt9 | 46 | 35 | 34 | 13 | 21 | 25 | 1.84534 | 0.00216 | 0.010119 | up |
| ENSMUS( | Ifitm6 | 30 | 15 | 23 | 8 | 11 | 8 | 2.375127 | 0.002197 | 0.010279 | up |
| ENSMUS( | Crhr2 | 90 | 62 | 69 | 35 | 53 | 40 | 1.627428 | 0.002199 | 0.010285 | up |
| ENSMUS( | Ifnlr1 | 4 | 8 | 7 | 0 | 1 | 1 | 9.206608 | 0.002291 | 0.010659 | up |
| ENSMUS( | Upb1 | 17 | 12 | 7 | 5 | 3 | 2 | 3.306992 | 0.002363 | 0.010972 | up |
| ENSMUS( | Trim12a | 10 | 5 | 11 | 1 | 3 | 1 | 4.951271 | 0.002379 | 0.011039 | up |
| ENSMUS( | Oprd1 | 47 | 37 | 38 | 24 | 21 | 21 | 1.740458 | 0.002404 | 0.011142 | up |
| ENSMUS( | Ammecr1 | 76 | 70 | 82 | 39 | 53 | 51 | 1.517867 | 0.002445 | 0.011298 | up |
| ENSMUS( | Pabpc5 | 15 | 13 | 23 | 5 | 7 | 5 | 2.880167 | 0.002532 | 0.011659 | up |
| ENSMUS( | Col20a1 | 48 | 62 | 46 | 31 | 27 | 33 | 1.619514 | 0.002556 | 0.011753 | up |
| ENSMUS( | Psmb9 | 75 | 68 | 61 | 43 | 27 | 48 | 1.632717 | 0.002642 | 0.012112 | up |
| ENSMUS( | Pcdhga9 | 70 | 72 | 53 | 38 | 30 | 49 | 1.571884 | 0.002667 | 0.012212 | up |
| ENSMUS( | Npffr1 | 73 | 68 | 52 | 44 | 35 | 40 | 1.521139 | 0.002668 | 0.012213 | up |
| ENSMUS( | Itk | 29 | 27 | 15 | 8 | 8 | 15 | 2.150652 | 0.002682 | 0.012274 | up |
| ENSMUS( | Abhd14b | 100 | 95 | 94 | 53 | 47 | 79 | 1.535183 | 0.002685 | 0.012281 | up |
| ENSMUS( | Angptl4 | 99 | 109 | 72 | 69 | 47 | 39 | 1.684411 | 0.002699 | 0.012339 | up |
| ENSMUS( | Neb | 48 | 59 | 50 | 34 | 23 | 35 | 1.616787 | 0.002703 | 0.012356 | up |
| ENSMUS( | Rgag1 | 31 | 33 | 25 | 18 | 15 | 10 | 1.935104 | 0.002707 | 0.012368 | up |
| ENSMUS( | Scgb3a1 | 15 | 25 | 15 | 9 | 7 | 5 | 2.453087 | 0.002717 | 0.012406 | up |
| ENSMUS( | Dhx58 | 37 | 30 | 28 | 6 | 9 | 21 | 2.519569 | 0.002742 | 0.012506 | up |
| ENSMUS( | Gm21897 | 30 | 28 | 40 | 17 | 10 | 6 | 2.808655 | 0.002776 | 0.012636 | up |
| ENSMUS( | Ugt1a7c | 5 | 2 | 10 | 1 | 0 | 0 | 16.10892 | 0.002786 | 0.012683 | up |
| ENSMUS( | Nepro | 111 | 82 | 93 | 60 | 68 | 43 | 1.572988 | 0.0028 | 0.012739 | up |
| ENSMUS( | Map3k8 | 51 | 39 | 29 | 19 | 23 | 21 | 1.765686 | 0.002834 | 0.012862 | up |
| ENSMUS( | Gm3591 | 12 | 18 | 14 | 6 | 1 | 3 | 4.137466 | 0.002834 | 0.012862 | up |
| ENSMUS( | Gm3739 | 116 | 105 | 81 | 53 | 48 | 86 | 1.525272 | 0.002863 | 0.012978 | up |
| ENSMUS( | Slamf7 | 10 | 6 | 5 | 1 | 2 | 0 | 6.430745 | 0.002906 | 0.013145 | up |
| ENSMUS( | Ddx60 | 51 | 41 | 38 | 11 | 26 | 26 | 1.954489 | 0.002935 | 0.013265 | up |
| ENSMUS( | Exph5 | 27 | 43 | 29 | 12 | 15 | 21 | 1.960866 | 0.002939 | 0.013279 | up |
| ENSMUS( | Bspry | 41 | 47 | 28 | 21 | 15 | 26 | 1.758298 | 0.002941 | 0.013287 | up |
| ENSMUS( | Pot1b | 67 | 57 | 55 | 37 | 29 | 43 | 1.552052 | 0.002983 | 0.013458 | up |
| ENSMUS( | Crb3 | 35 | 58 | 44 | 10 | 25 | 21 | 2.332216 | 0.003067 | 0.013792 | up |
| ENSMUS( | Pcdhgb2 | 59 | 53 | 48 | 30 | 27 | 38 | 1.591995 | 0.00313 | 0.014057 | up |
| ENSMUS( | Traf3ip3 | 26 | 14 | 20 | 4 | 4 | 11 | 3.019936 | 0.003141 | 0.014098 | up |
| ENSMUS( | Efcc1 | 42 | 65 | 66 | 30 | 34 | 31 | 1.739532 | 0.003206 | 0.014372 | up |
| ENSMUS( | Magix | 16 | 12 | 7 | 3 | 5 | 2 | 3.233458 | 0.003252 | 0.014557 | up |
| ENSMUS( | Mylk4 | 7 | 9 | 7 | 2 | 1 | 1 | 5.392912 | 0.003263 | 0.0146 | up |
| ENSMUS( | Vmn1r58 | 5 | 9 | 2 | 0 | 0 | 1 | 15.36338 | 0.0033 | 0.01475 | up |
| ENSMUS( | Gm853 | 9 | 14 | 8 | 4 | 1 | 3 | 3.636619 | 0.003508 | 0.015574 | up |
| ENSMUS( | Clec4a3 | 54 | 51 | 42 | 29 | 24 | 33 | 1.611258 | 0.003573 | 0.015842 | up |
| ENSMUS( | Hist1h2bg | 12 | 6 | 9 | 3 | 1 | 2 | 4.228218 | 0.00358 | 0.01587 | up |
| ENSMUS( | C3ar1 | 63 | 48 | 49 | 29 | 32 | 35 | 1.573188 | 0.003803 | 0.016736 | up |
| ENSMUS( | Dennd2c | 20 | 17 | 21 | 6 | 11 | 7 | 2.295072 | 0.003818 | 0.016798 | up |
| ENSMUS( | Acvr2b | 20 | 32 | 17 | 10 | 7 | 12 | 2.241609 | 0.003847 | 0.016908 | up |
| ENSMUS( | Cage1 | 61 | 62 | 44 | 30 | 23 | 42 | 1.657988 | 0.003872 | 0.016999 | up |
| ENSMUS( | Trim12c | 37 | 30 | 34 | 14 | 18 | 21 | 1.810791 | 0.003895 | 0.017083 | up |
| ENSMUS( | Ptar1 | 33 | 25 | 24 | 13 | 14 | 13 | 1.927137 | 0.004068 | 0.017762 | up |
| ENSMUS( | Slfn3 | 10 | 2 | 4 | 0 | 1 | 0 | 14.74886 | 0.004103 | 0.017881 | up |
| ENSMUS( | Slc16a14 | 66 | 79 | 48 | 44 | 29 | 39 | 1.614513 | 0.004166 | 0.018117 | up |
| ENSMUS( | Slfn4 | 11 | 17 | 8 | 4 | 5 | 2 | 3.041262 | 0.004172 | 0.018136 | up |
| ENSMUS( | Lax1 | 5 | 22 | 8 | 1 | 3 | 4 | 4.171732 | 0.004182 | 0.018161 | up |
| ENSMUS( | Cd300lg | 6 | 5 | 9 | 1 | 2 | 0 | 6.312718 | 0.004278 | 0.018537 | up |
| ENSMUS( | Lmntd2 | 5 | 7 | 8 | 2 | 1 | 0 | 6.242343 | 0.004285 | 0.018565 | up |
| ENSMUS( | Jaml | 25 | 16 | 8 | 5 | 4 | 7 | 2.839971 | 0.004316 | 0.018665 | up |
| ENSMUS( | Pim1 | 29 | 38 | 29 | 17 | 17 | 16 | 1.812394 | 0.004455 | 0.019226 | up |
| ENSMUS( | Asb4 | 25 | 22 | 15 | 8 | 8 | 11 | 2.155215 | 0.004507 | 0.019423 | up |
| ENSMUS( | Myo1f | 52 | 66 | 59 | 34 | 34 | 42 | 1.52988 | 0.004728 | 0.02027 | up |
| ENSMUS( | Zfp54 | 48 | 45 | 27 | 16 | 24 | 25 | 1.729839 | 0.004732 | 0.020275 | up |
| ENSMUS( | Akr1c14 | 36 | 38 | 37 | 18 | 20 | 23 | 1.728395 | 0.004797 | 0.02052 | up |
| ENSMUS( | Tns4 | 11 | 14 | 14 | 5 | 6 | 2 | 2.829021 | 0.004825 | 0.020626 | up |
| ENSMUS( | Acacb | 165 | 155 | 97 | 96 | 88 | 71 | 1.522332 | 0.004938 | 0.021065 | up |
| ENSMUS( | Gapdhs | 30 | 32 | 19 | 14 | 10 | 16 | 1.898329 | 0.004963 | 0.021155 | up |
| ENSMUS( | Zc3h12d | 7 | 9 | 8 | 1 | 1 | 3 | 4.619074 | 0.005045 | 0.021467 | up |
| ENSMUS( | Tlr2 | 68 | 67 | 64 | 36 | 51 | 27 | 1.644081 | 0.00518 | 0.021984 | up |
| ENSMUS( | Ikbke | 23 | 27 | 26 | 12 | 12 | 13 | 1.951006 | 0.005626 | 0.023693 | up |
| ENSMUS( | Il18rap | 9 | 10 | 9 | 1 | 1 | 4 | 4.496517 | 0.005711 | 0.024008 | up |
| ENSMUS( | Spint1 | 25 | 37 | 35 | 16 | 13 | 21 | 1.855202 | 0.005851 | 0.024536 | up |
| ENSMUS( | Siglec1 | 18 | 19 | 17 | 1 | 9 | 5 | 3.422431 | 0.005933 | 0.024836 | up |
| ENSMUS( | Rab20 | 34 | 22 | 27 | 15 | 13 | 14 | 1.863473 | 0.005941 | 0.024865 | up |
| ENSMUS( | Ercc2 | 87 | 79 | 62 | 61 | 41 | 34 | 1.564034 | 0.006102 | 0.025453 | up |
| ENSMUS( | F10 | 6 | 10 | 0 | 1 | 0 | 0 | 14.14132 | 0.006107 | 0.025466 | up |
| ENSMUS( | Prrg4 | 11 | 14 | 13 | 5 | 1 | 1 | 5.068274 | 0.006117 | 0.025503 | up |

| | | | | | | | | | | |
|---|---|---|---|---|---|---|---|---|---|---|
| ENSMUS( | Tes | 63 | 52 | 43 | 37 | 24 | 35 | 1.544315 | 0.006185 | 0.025755 | up |
| ENSMUS( | Tmem71 | 23 | 17 | 14 | 5 | 2 | 10 | 3.008472 | 0.006228 | 0.02591 | up |
| ENSMUS( | Eps8l1 | 18 | 15 | 15 | 7 | 6 | 6 | 2.379328 | 0.006299 | 0.026188 | up |
| ENSMUS( | 1810011O | 46 | 42 | 35 | 20 | 23 | 28 | 1.635229 | 0.006322 | 0.026264 | up |
| ENSMUS( | Mycbpap | 59 | 56 | 45 | 41 | 15 | 28 | 1.784513 | 0.006422 | 0.026636 | up |
| ENSMUS( | Slc6a20b | 16 | 26 | 21 | 3 | 10 | 11 | 2.518186 | 0.006632 | 0.027419 | up |
| ENSMUS( | Dpep1 | 19 | 10 | 22 | 4 | 9 | 6 | 2.571487 | 0.006649 | 0.027473 | up |
| ENSMUS( | Igsf10 | 44 | 41 | 39 | 27 | 23 | 22 | 1.62261 | 0.006724 | 0.027743 | up |
| ENSMUS( | Cd3g | 10 | 13 | 8 | 3 | 1 | 5 | 3.266468 | 0.006731 | 0.02777 | up |
| ENSMUS( | Muc1 | 15 | 13 | 18 | 7 | 3 | 8 | 2.445018 | 0.006946 | 0.02852 | up |
| ENSMUS( | Dqx1 | 13 | 19 | 14 | 5 | 6 | 7 | 2.42525 | 0.006959 | 0.028557 | up |
| ENSMUS( | C1qtnf1 | 43 | 48 | 29 | 19 | 23 | 27 | 1.63453 | 0.007019 | 0.02877 | up |
| ENSMUS( | Onecut1 | 6 | 8 | 7 | 1 | 1 | 2 | 5.025423 | 0.007034 | 0.028824 | up |
| ENSMUS( | Ccdc125 | 21 | 22 | 26 | 14 | 11 | 8 | 1.979863 | 0.007065 | 0.02893 | up |
| ENSMUS( | Cfap53 | 27 | 17 | 16 | 10 | 9 | 8 | 2.074572 | 0.007099 | 0.029045 | up |
| ENSMUS( | AB124611 | 22 | 21 | 34 | 11 | 14 | 6 | 2.36934 | 0.00725 | 0.029622 | up |
| ENSMUS( | Ctla2b | 43 | 51 | 36 | 31 | 22 | 17 | 1.736448 | 0.007255 | 0.029641 | up |
| ENSMUS( | Chmp4c | 28 | 21 | 23 | 7 | 11 | 16 | 2.015851 | 0.007279 | 0.02973 | up |
| ENSMUS( | Ttll8 | 19 | 25 | 21 | 9 | 12 | 3 | 2.540155 | 0.007402 | 0.030181 | up |
| ENSMUS( | Pcdhb21 | 39 | 33 | 35 | 20 | 23 | 17 | 1.681972 | 0.007456 | 0.030358 | up |
| ENSMUS( | Lin28b | 56 | 56 | 29 | 31 | 26 | 21 | 1.676087 | 0.00751 | 0.030544 | up |
| ENSMUS( | Aire | 20 | 27 | 26 | 15 | 12 | 9 | 1.918665 | 0.007686 | 0.031167 | up |
| ENSMUS( | Ptchd1 | 27 | 28 | 22 | 16 | 12 | 7 | 2.053171 | 0.007786 | 0.031545 | up |
| ENSMUS( | Isg15 | 44 | 55 | 45 | 29 | 33 | 15 | 1.756247 | 0.008009 | 0.032377 | up |
| ENSMUS( | Mybpc2 | 8 | 14 | 10 | 2 | 3 | 5 | 3.063337 | 0.00822 | 0.033106 | up |
| ENSMUS( | Gm43951 | 18 | 11 | 16 | 1 | 7 | 1 | 4.713669 | 0.008359 | 0.033601 | up |
| ENSMUS( | Irf4 | 13 | 21 | 16 | 9 | 4 | 8 | 2.25688 | 0.008427 | 0.033825 | up |
| ENSMUS( | Ccl8 | 32 | 19 | 9 | 9 | 5 | 9 | 2.404241 | 0.008447 | 0.033889 | up |
| ENSMUS( | Disc1 | 15 | 14 | 13 | 6 | 4 | 6 | 2.47898 | 0.008487 | 0.034022 | up |
| ENSMUS( | Tsnaxip1 | 26 | 21 | 24 | 11 | 15 | 9 | 1.914182 | 0.008562 | 0.03429 | up |
| ENSMUS( | Myom1 | 45 | 52 | 50 | 32 | 34 | 22 | 1.577791 | 0.008566 | 0.034299 | up |
| ENSMUS( | Sh3rf2 | 10 | 15 | 12 | 5 | 5 | 3 | 2.683448 | 0.008573 | 0.034314 | up |
| ENSMUS( | Spink10 | 47 | 46 | 48 | 23 | 28 | 35 | 1.561037 | 0.008663 | 0.034639 | up |
| ENSMUS( | Cdca7l | 29 | 24 | 25 | 16 | 12 | 12 | 1.835866 | 0.00895 | 0.03563 | up |
| ENSMUS( | Hp | 18 | 16 | 24 | 5 | 11 | 3 | 2.901882 | 0.008981 | 0.035732 | up |
| ENSMUS( | H2-M3 | 41 | 40 | 33 | 17 | 26 | 23 | 1.629805 | 0.008991 | 0.035763 | up |
| ENSMUS( | Gm1043 | 72 | 66 | 48 | 34 | 49 | 30 | 1.539023 | 0.009105 | 0.036178 | up |
| ENSMUS( | Ccdc167 | 70 | 69 | 60 | 30 | 52 | 41 | 1.528584 | 0.009129 | 0.036265 | up |
| ENSMUS( | Rhbdf2 | 36 | 20 | 24 | 12 | 17 | 11 | 1.875484 | 0.009326 | 0.036949 | up |
| ENSMUS( | Dcdc2c | 2 | 3 | 9 | 0 | 0 | 1 | 14.33087 | 0.009597 | 0.03789 | up |
| ENSMUS( | Ms4a4d | 6 | 9 | 11 | 1 | 4 | 2 | 3.573759 | 0.009812 | 0.03859 | up |
| ENSMUS( | Arhgap28 | 39 | 38 | 31 | 22 | 20 | 20 | 1.637653 | 0.009867 | 0.038792 | up |
| ENSMUS( | Pkd1l3 | 60 | 51 | 52 | 20 | 45 | 25 | 1.710268 | 0.01014 | 0.039761 | up |
| ENSMUS( | Atp2c2 | 39 | 28 | 36 | 14 | 15 | 26 | 1.787616 | 0.010192 | 0.039927 | up |
| ENSMUS( | Trim43b | 7 | 5 | 8 | 2 | 2 | 0 | 4.682418 | 0.010197 | 0.039937 | up |
| ENSMUS( | Armc12 | 7 | 8 | 5 | 1 | 2 | 1 | 4.679228 | 0.010298 | 0.04029 | up |
| ENSMUS( | Serpina1b | 30 | 34 | 42 | 18 | 21 | 19 | 1.746463 | 0.010438 | 0.040792 | up |
| ENSMUS( | Cldn3 | 11 | 16 | 18 | 9 | 5 | 4 | 2.372347 | 0.01046 | 0.040854 | up |
| ENSMUS( | Clec4a1 | 24 | 30 | 18 | 16 | 8 | 9 | 2.034279 | 0.01052 | 0.041065 | up |
| ENSMUS( | 9930012K | 34 | 26 | 27 | 11 | 20 | 16 | 1.749112 | 0.010553 | 0.041179 | up |
| ENSMUS( | Kif11 | 41 | 45 | 52 | 26 | 32 | 17 | 1.744595 | 0.011175 | 0.043251 | up |
| ENSMUS( | Bcl6b | 41 | 30 | 32 | 18 | 18 | 23 | 1.650109 | 0.011542 | 0.044523 | up |
| ENSMUS( | Kcnh5 | 41 | 46 | 44 | 28 | 23 | 29 | 1.553527 | 0.011672 | 0.044958 | up |
| ENSMUS( | Aim1 | 34 | 21 | 34 | 11 | 13 | 21 | 1.894527 | 0.011917 | 0.045803 | up |
| ENSMUS( | Glcci1 | 17 | 10 | 12 | 3 | 6 | 6 | 2.460306 | 0.01224 | 0.046923 | up |
| ENSMUS( | Zfp977 | 17 | 20 | 16 | 9 | 5 | 10 | 2.090178 | 0.012305 | 0.047129 | up |
| ENSMUS( | Gm7173 | 58 | 42 | 45 | 34 | 26 | 31 | 1.500412 | 0.012308 | 0.047129 | up |
| ENSMUS( | Htr1f | 38 | 28 | 26 | 18 | 21 | 12 | 1.685628 | 0.012552 | 0.047923 | up |
| ENSMUS( | Slc12a1 | 4 | 4 | 7 | 0 | 1 | 1 | 7.333809 | 0.012551 | 0.047923 | up |
| ENSMUS( | Trpm5 | 7 | 2 | 6 | 1 | 0 | 1 | 7.145822 | 0.012624 | 0.04815 | up |
| ENSMUS( | Macc1 | 5 | 6 | 4 | 1 | 1 | 0 | 6.922802 | 0.012664 | 0.048265 | up |
| ENSMUS( | Sgsh | 67 | 52 | 62 | 42 | 43 | 27 | 1.522366 | 0.012803 | 0.048755 | up |
| ENSMUS( | Clic3 | 34 | 25 | 25 | 7 | 19 | 14 | 1.983953 | 0.012943 | 0.049216 | up |
| ENSMUS( | Zfp973 | 6 | 11 | 7 | 3 | 2 | 0 | 4.446654 | 0.012989 | 0.049362 | up |
| ENSMUS( | Zfp442 | 42 | 41 | 60 | 31 | 25 | 18 | 1.839565 | 0.013013 | 0.049433 | up |
| ENSMUS( | B4galnt3 | 13 | 9 | 5 | 2 | 2 | 4 | 3.154048 | 0.013015 | 0.049433 | up |
| ENSMUS( | Pcdha3 | 26 | 40 | 26 | 19 | 19 | 12 | 1.726749 | 0.013131 | 0.049814 | up |
| ENSMUS( | Pcsk1n | 1801 | 1808 | 1679 | 8427 | 4161 | 4215 | 0.295218 | 5.29E-254 | 2.98E-250 | down |
| ENSMUS( | Cck | 2934 | 2666 | 2394 | 8658 | 4447 | 4272 | 0.430195 | 1.59E-143 | 3.43E-140 | down |
| ENSMUS( | Fth1 | 33657 | 33713 | 30458 | 57642 | 56235 | 55778 | 0.54427 | 6.53E-124 | 1.08E-120 | down |
| ENSMUS( | Olig1 | 1792 | 1718 | 1539 | 3019 | 3075 | 3159 | 0.514895 | 7.52E-108 | 9.63E-105 | down |
| ENSMUS( | Fabp7 | 561 | 545 | 438 | 1211 | 1263 | 1338 | 0.3814 | 1.33E-104 | 1.56E-101 | down |
| ENSMUS( | Rpl35a | 1102 | 1045 | 926 | 1920 | 1920 | 2121 | 0.486543 | 2.94E-101 | 3.07E-98 | down |
| ENSMUS( | Nnat | 5999 | 5954 | 5443 | 9258 | 9045 | 9365 | 0.593862 | 1.63E-96 | 1.64E-93 | down |
| ENSMUS( | Trnp1 | 1496 | 1401 | 1257 | 2658 | 2461 | 2544 | 0.510851 | 4.60E-88 | 3.81E-85 | down |
| ENSMUS( | Cnih2 | 6305 | 5989 | 5099 | 9988 | 9810 | 10249 | 0.545269 | 7.37E-87 | 5.93E-84 | down |
| ENSMUS( | Ptms | 5314 | 5374 | 4807 | 7786 | 8142 | 8059 | 0.61007 | 1.73E-82 | 1.22E-79 | down |
| ENSMUS( | Mt3 | 4998 | 4772 | 4122 | 6915 | 7108 | 7494 | 0.608809 | 3.80E-82 | 2.61E-79 | down |
| ENSMUS( | Dpysl2 | 3116 | 2988 | 2657 | 4453 | 4633 | 4921 | 0.590453 | 6.02E-82 | 4.04E-79 | down |
| ENSMUS( | Syn1 | 6041 | 6039 | 5383 | 9198 | 9049 | 8781 | 0.609485 | 7.71E-81 | 4.72E-78 | down |

| | | | | | | | | | | | |
|---|---|---|---|---|---|---|---|---|---|---|---|
| ENSMUS( | Ccdc85b | 473 | 466 | 441 | 1722 | 802 | 764 | 0.393271 | 1.00E-78 | 5.87E-76 | down |
| ENSMUS( | Chd9 | 1023 | 965 | 902 | 1739 | 1742 | 1739 | 0.522606 | 2.09E-78 | 1.20E-75 | down |
| ENSMUS( | Nrgn | 13431 | 12880 | 11449 | 21691 | 17295 | 18066 | 0.622978 | 6.55E-77 | 3.54E-74 | down |
| ENSMUS( | Apoe | 32385 | 30262 | 27410 | 49705 | 42358 | 43206 | 0.626907 | 4.02E-76 | 2.14E-73 | down |
| ENSMUS( | Cdk5r2 | 2552 | 2556 | 2285 | 3934 | 3732 | 3989 | 0.598924 | 3.10E-73 | 1.56E-70 | down |
| ENSMUS( | Abhd17a | 1224 | 1161 | 1106 | 2152 | 1945 | 2143 | 0.528535 | 7.85E-73 | 3.88E-70 | down |
| ENSMUS( | Ddah1 | 2085 | 1993 | 1715 | 3038 | 2989 | 3095 | 0.598402 | 3.29E-66 | 1.43E-63 | down |
| ENSMUS( | Cox4i1 | 5026 | 4933 | 4189 | 6535 | 6741 | 7545 | 0.641312 | 6.79E-66 | 2.90E-63 | down |
| ENSMUS( | Rpsa | 8014 | 7304 | 6547 | 11234 | 11095 | 11945 | 0.601523 | 1.66E-65 | 6.87E-63 | down |
| ENSMUS( | Ctxn1 | 4399 | 4507 | 3977 | 6983 | 6564 | 6781 | 0.598141 | 1.73E-65 | 7.08E-63 | down |
| ENSMUS( | Cst3 | 30055 | 28028 | 24466 | 41212 | 42253 | 46159 | 0.600649 | 2.10E-65 | 8.44E-63 | down |
| ENSMUS( | Rps5 | 3044 | 2851 | 2494 | 4163 | 4035 | 4543 | 0.620994 | 2.67E-65 | 1.06E-62 | down |
| ENSMUS( | Ndufb9 | 2834 | 2606 | 2326 | 3764 | 3829 | 4226 | 0.619976 | 3.93E-64 | 1.51E-61 | down |
| ENSMUS( | Rps15 | 5254 | 4809 | 4349 | 6674 | 6789 | 7581 | 0.646449 | 7.04E-64 | 2.68E-61 | down |
| ENSMUS( | Tmsb10 | 1616 | 1465 | 1282 | 2452 | 2394 | 2669 | 0.547176 | 1.43E-63 | 5.38E-61 | down |
| ENSMUS( | C1qtnf4 | 380 | 367 | 332 | 1213 | 657 | 675 | 0.397574 | 1.85E-63 | 6.85E-61 | down |
| ENSMUS( | Cox6a1 | 6324 | 5985 | 5160 | 8201 | 8455 | 9183 | 0.63762 | 2.14E-62 | 7.63E-60 | down |
| ENSMUS( | Rps2 | 2823 | 2747 | 2309 | 4490 | 4068 | 4265 | 0.578424 | 2.13E-61 | 7.51E-59 | down |
| ENSMUS( | Epn1 | 6579 | 6378 | 5844 | 8761 | 8938 | 9166 | 0.66094 | 3.07E-61 | 1.07E-58 | down |
| ENSMUS( | Mlf2 | 6662 | 6092 | 5617 | 9125 | 8508 | 8904 | 0.652709 | 8.99E-61 | 3.05E-58 | down |
| ENSMUS( | Vegfb | 747 | 761 | 633 | 1167 | 1260 | 1344 | 0.535853 | 1.59E-60 | 5.34E-58 | down |
| ENSMUS( | Sox10 | 385 | 381 | 359 | 754 | 717 | 796 | 0.469373 | 1.65E-60 | 5.45E-58 | down |
| ENSMUS( | Me1 | 1548 | 1419 | 1292 | 2177 | 2281 | 2316 | 0.593076 | 3.45E-60 | 1.13E-57 | down |
| ENSMUS( | Nrsn1 | 6154 | 5847 | 5409 | 7951 | 8286 | 8587 | 0.662487 | 1.22E-59 | 3.87E-57 | down |
| ENSMUS( | Rpl38 | 1845 | 1690 | 1541 | 2609 | 2504 | 2771 | 0.607463 | 3.63E-59 | 1.11E-56 | down |
| ENSMUS( | Psd | 9605 | 9333 | 8596 | 13716 | 12938 | 12599 | 0.661668 | 1.71E-58 | 5.08E-56 | down |
| ENSMUS( | Prrt1 | 2032 | 1920 | 1793 | 3259 | 2999 | 2937 | 0.589146 | 6.35E-57 | 1.73E-54 | down |
| ENSMUS( | Htra1 | 2902 | 2751 | 2381 | 3862 | 3910 | 4080 | 0.638968 | 1.39E-56 | 3.75E-54 | down |
| ENSMUS( | Fabp5 | 1754 | 1729 | 1473 | 2483 | 2480 | 2672 | 0.612253 | 1.47E-56 | 3.94E-54 | down |
| ENSMUS( | Slc25a18 | 1236 | 1164 | 1079 | 1838 | 1863 | 1843 | 0.592194 | 5.49E-55 | 1.42E-52 | down |
| ENSMUS( | Gpr37l1 | 3959 | 3676 | 3299 | 5070 | 5251 | 5331 | 0.658793 | 3.34E-54 | 8.41E-52 | down |
| ENSMUS( | Rps10 | 3288 | 2993 | 2721 | 4163 | 4270 | 4600 | 0.651759 | 7.58E-54 | 1.89E-51 | down |
| ENSMUS( | Rnd2 | 1550 | 1433 | 1289 | 2137 | 2168 | 2278 | 0.612112 | 6.25E-53 | 1.48E-50 | down |
| ENSMUS( | S100a16 | 1053 | 961 | 886 | 1455 | 1590 | 1633 | 0.58524 | 2.65E-52 | 6.11E-50 | down |
| ENSMUS( | Erdr1 | 113 | 105 | 98 | 474 | 312 | 277 | 0.278925 | 4.46E-52 | 1.02E-49 | down |
| ENSMUS( | P2ry12 | 1229 | 1149 | 991 | 1820 | 1714 | 1796 | 0.595192 | 6.92E-52 | 1.57E-49 | down |
| ENSMUS( | Rpl27 | 3108 | 2885 | 2528 | 4024 | 3997 | 4221 | 0.656023 | 5.59E-51 | 1.26E-48 | down |
| ENSMUS( | Sox2 | 721 | 672 | 619 | 1130 | 1148 | 1131 | 0.556739 | 6.54E-51 | 1.46E-48 | down |
| ENSMUS( | H2-K1 | 113 | 101 | 99 | 273 | 349 | 341 | 0.307498 | 9.92E-51 | 2.20E-48 | down |
| ENSMUS( | Bcas1 | 1998 | 1942 | 1686 | 2641 | 2801 | 2858 | 0.639434 | 2.08E-49 | 4.44E-47 | down |
| ENSMUS( | Nbl1 | 1213 | 1199 | 1086 | 1711 | 1805 | 1904 | 0.609974 | 3.92E-49 | 8.17E-47 | down |
| ENSMUS( | Cryab | 1393 | 1354 | 1100 | 2196 | 2062 | 2285 | 0.553545 | 9.36E-48 | 1.82E-45 | down |
| ENSMUS( | Agap3 | 3459 | 3369 | 2978 | 4773 | 4594 | 4592 | 0.662255 | 3.07E-47 | 5.84E-45 | down |
| ENSMUS( | S100b | 2891 | 2734 | 2303 | 4022 | 4077 | 4211 | 0.606529 | 3.64E-47 | 6.83E-45 | down |
| ENSMUS( | Dynlrb1 | 2838 | 2696 | 2362 | 3611 | 3594 | 4008 | 0.664467 | 6.94E-47 | 1.29E-44 | down |
| ENSMUS( | Maz | 1969 | 1888 | 1798 | 2676 | 2760 | 2786 | 0.650073 | 2.07E-46 | 3.73E-44 | down |
| ENSMUS( | Hmga1-rs1 | 215 | 193 | 160 | 375 | 437 | 451 | 0.423461 | 5.84E-46 | 1.04E-43 | down |
| ENSMUS( | Eif3f | 1688 | 1732 | 1562 | 2334 | 2594 | 2537 | 0.630892 | 1.27E-45 | 2.23E-43 | down |
| ENSMUS( | 1110008P1 | 1347 | 1271 | 1088 | 1836 | 1806 | 1942 | 0.625408 | 7.96E-45 | 1.32E-42 | down |
| ENSMUS( | Tpm3-rs7 | 882 | 821 | 703 | 1226 | 1260 | 1346 | 0.591709 | 1.00E-44 | 1.65E-42 | down |
| ENSMUS( | Rtn2 | 1078 | 1057 | 916 | 1568 | 1594 | 1538 | 0.611789 | 1.16E-44 | 1.90E-42 | down |
| ENSMUS( | Rpl18a | 4153 | 3711 | 3276 | 5708 | 5805 | 6306 | 0.589225 | 1.20E-43 | 1.91E-41 | down |
| ENSMUS( | Rplp1 | 9681 | 8762 | 7774 | 12526 | 12879 | 14514 | 0.619591 | 8.62E-43 | 1.32E-40 | down |
| ENSMUS( | Cox8a | 8267 | 8698 | 7364 | 12830 | 12662 | 13016 | 0.596169 | 1.28E-42 | 1.94E-40 | down |
| ENSMUS( | Dact3 | 1500 | 1494 | 1224 | 2471 | 2217 | 2346 | 0.564384 | 2.91E-42 | 4.33E-40 | down |
| ENSMUS( | Hspe1 | 1294 | 1179 | 1125 | 1743 | 1694 | 1929 | 0.633684 | 3.02E-42 | 4.47E-40 | down |
| ENSMUS( | Rnf208 | 1448 | 1404 | 1246 | 1908 | 1972 | 2143 | 0.642548 | 4.02E-42 | 5.93E-40 | down |
| ENSMUS( | Rpl31 | 3991 | 3612 | 3249 | 5045 | 4947 | 5602 | 0.65651 | 1.21E-41 | 1.73E-39 | down |
| ENSMUS( | Lhx2 | 1233 | 1157 | 993 | 1665 | 1642 | 1748 | 0.630555 | 2.99E-41 | 4.23E-39 | down |
| ENSMUS( | Drap1 | 1760 | 1671 | 1551 | 2327 | 2283 | 2516 | 0.660565 | 1.43E-40 | 2.00E-38 | down |
| ENSMUS( | Begain | 1569 | 1395 | 1265 | 2540 | 2183 | 2305 | 0.566335 | 1.46E-40 | 2.03E-38 | down |
| ENSMUS( | Pfn1 | 2021 | 1935 | 1850 | 2778 | 2647 | 2898 | 0.659528 | 3.04E-40 | 4.21E-38 | down |
| ENSMUS( | Ube2m | 1532 | 1433 | 1272 | 2059 | 1945 | 2112 | 0.653172 | 2.39E-39 | 3.14E-37 | down |
| ENSMUS( | Rps12 | 4427 | 4048 | 3549 | 5589 | 5558 | 6293 | 0.650185 | 5.46E-39 | 7.02E-37 | down |
| ENSMUS( | Ndufb5 | 1308 | 1207 | 1055 | 1673 | 1721 | 1842 | 0.642818 | 6.05E-39 | 7.74E-37 | down |
| ENSMUS( | Npy | 488 | 418 | 378 | 792 | 739 | 792 | 0.520298 | 9.55E-39 | 1.21E-36 | down |
| ENSMUS( | Lcat | 455 | 431 | 401 | 704 | 697 | 789 | 0.555502 | 1.83E-38 | 2.29E-36 | down |
| ENSMUS( | Rpl37a | 2108 | 1917 | 1664 | 2825 | 2997 | 3446 | 0.57913 | 2.56E-38 | 3.19E-36 | down |
| ENSMUS( | Map1lc3a | 1549 | 1472 | 1324 | 2031 | 2045 | 2120 | 0.661674 | 1.47E-37 | 1.77E-35 | down |
| ENSMUS( | Ssbp4 | 1389 | 1362 | 1258 | 2143 | 1995 | 1923 | 0.623938 | 1.68E-37 | 2.02E-35 | down |
| ENSMUS( | Mbd3 | 1253 | 1298 | 1085 | 1691 | 1801 | 1857 | 0.641506 | 9.60E-37 | 1.11E-34 | down |
| ENSMUS( | Abhd4 | 1407 | 1285 | 1143 | 1862 | 1751 | 1892 | 0.65646 | 1.67E-36 | 1.91E-34 | down |
| ENSMUS( | Rplp2 | 2105 | 2156 | 1868 | 2537 | 3103 | 3234 | 0.653241 | 2.25E-36 | 2.55E-34 | down |
| ENSMUS( | Rsph1 | 299 | 255 | 235 | 511 | 501 | 476 | 0.49886 | 3.11E-36 | 3.47E-34 | down |
| ENSMUS( | Mid1ip1 | 837 | 784 | 718 | 1136 | 1265 | 1196 | 0.613595 | 3.98E-36 | 4.40E-34 | down |
| ENSMUS( | Eef1g | 3964 | 3583 | 3189 | 4986 | 4999 | 5386 | 0.658441 | 9.37E-36 | 1.03E-33 | down |
| ENSMUS( | Hopx | 744 | 663 | 587 | 991 | 1052 | 1068 | 0.603856 | 1.03E-35 | 1.12E-33 | down |
| ENSMUS( | Cox5a | 297 | 264 | 285 | 1394 | 507 | 462 | 0.335 | 1.67E-35 | 1.80E-33 | down |
| ENSMUS( | Vstm2l | 282 | 280 | 235 | 645 | 536 | 462 | 0.455372 | 2.49E-35 | 2.64E-33 | down |
| ENSMUS( | Tpgs1 | 509 | 548 | 452 | 832 | 801 | 875 | 0.567884 | 4.73E-35 | 4.95E-33 | down |

| | | | | | | | | | | | |
|---|---|---|---|---|---|---|---|---|---|---|---|
| ENSMUS( | Ube2ql1 | 2529 | 2299 | 2014 | 3252 | 3201 | 3436 | 0.651883 | 1.30E-34 | 1.35E-32 | down |
| ENSMUS( | Rps16 | 2840 | 2706 | 2272 | 3611 | 3621 | 4146 | 0.647909 | 3.49E-34 | 3.49E-32 | down |
| ENSMUS( | Uchl1 | 5926 | 5356 | 4737 | 7416 | 7594 | 8144 | 0.65219 | 3.70E-34 | 3.68E-32 | down |
| ENSMUS( | Tril | 717 | 700 | 621 | 1021 | 1048 | 1044 | 0.617639 | 3.92E-34 | 3.89E-32 | down |
| ENSMUS( | Rps18 | 382 | 323 | 274 | 689 | 849 | 906 | 0.377295 | 3.65E-33 | 3.37E-31 | down |
| ENSMUS( | Selenow | 11762 | 10508 | 9134 | 16542 | 15822 | 17445 | 0.593726 | 6.02E-33 | 5.45E-31 | down |
| ENSMUS( | Cxcl14 | 1506 | 1580 | 1432 | 2326 | 2214 | 2311 | 0.623327 | 1.54E-32 | 1.35E-30 | down |
| ENSMUS( | Elob | 1932 | 1766 | 1523 | 2504 | 2436 | 2685 | 0.645071 | 2.05E-32 | 1.77E-30 | down |
| ENSMUS( | Rps29 | 2688 | 2388 | 2135 | 3488 | 3743 | 4281 | 0.591196 | 2.79E-32 | 2.39E-30 | down |
| ENSMUS( | Uqcrfs1 | 1840 | 1781 | 1615 | 2669 | 2289 | 2513 | 0.661076 | 3.42E-32 | 2.91E-30 | down |
| ENSMUS( | Usmg5 | 1841 | 1686 | 1417 | 2424 | 2435 | 2760 | 0.611331 | 4.61E-32 | 3.87E-30 | down |
| ENSMUS( | Rps28 | 1248 | 1142 | 1054 | 1532 | 1619 | 1836 | 0.652609 | 1.02E-31 | 8.47E-30 | down |
| ENSMUS( | Gstm5 | 2914 | 2788 | 2305 | 3754 | 3841 | 4092 | 0.645357 | 3.10E-31 | 2.53E-29 | down |
| ENSMUS( | Rpl41 | 6456 | 5816 | 4912 | 9189 | 8664 | 9948 | 0.581923 | 9.22E-31 | 7.30E-29 | down |
| ENSMUS( | Cxx1a | 587 | 423 | 569 | 1115 | 1069 | 1105 | 0.455356 | 9.77E-31 | 7.71E-29 | down |
| ENSMUS( | Enho | 739 | 755 | 600 | 1057 | 1115 | 1164 | 0.59144 | 1.01E-30 | 7.93E-29 | down |
| ENSMUS( | Gm4737 | 47 | 53 | 38 | 138 | 149 | 163 | 0.288947 | 1.49E-30 | 1.16E-28 | down |
| ENSMUS( | Sst | 1865 | 1650 | 1374 | 2508 | 2581 | 2867 | 0.578358 | 1.71E-30 | 1.33E-28 | down |
| ENSMUS( | Ly6h | 3414 | 3169 | 2737 | 4690 | 4251 | 4400 | 0.657401 | 1.92E-30 | 1.48E-28 | down |
| ENSMUS( | Nudt19 | 801 | 724 | 645 | 1077 | 1028 | 1077 | 0.64237 | 1.46E-29 | 1.08E-27 | down |
| ENSMUS( | mt-Atp6 | 130 | 152 | 110 | 185 | 930 | 147 | 0.290905 | 2.00E-29 | 1.47E-27 | down |
| ENSMUS( | Rpl37 | 3448 | 3003 | 2810 | 4682 | 4432 | 5208 | 0.610267 | 2.96E-29 | 2.17E-27 | down |
| ENSMUS( | Atp5j2 | 2151 | 2108 | 1735 | 2878 | 2721 | 3164 | 0.644746 | 5.00E-29 | 3.61E-27 | down |
| ENSMUS( | Srp9 | 925 | 903 | 823 | 1222 | 1210 | 1340 | 0.664182 | 8.41E-29 | 5.98E-27 | down |
| ENSMUS( | Rhog | 366 | 349 | 283 | 555 | 583 | 533 | 0.561358 | 4.01E-28 | 2.75E-26 | down |
| ENSMUS( | Atpif1 | 395 | 369 | 321 | 688 | 577 | 619 | 0.541923 | 5.42E-28 | 3.70E-26 | down |
| ENSMUS( | Pole4 | 730 | 667 | 604 | 975 | 948 | 1179 | 0.609458 | 8.03E-28 | 5.42E-26 | down |
| ENSMUS( | Prr18 | 704 | 633 | 576 | 925 | 917 | 971 | 0.641208 | 1.04E-27 | 6.91E-26 | down |
| ENSMUS( | Cops9 | 985 | 857 | 768 | 1307 | 1282 | 1487 | 0.603704 | 1.22E-27 | 8.05E-26 | down |
| ENSMUS( | Pla2g7 | 3614 | 3135 | 2895 | 4622 | 4600 | 4842 | 0.646304 | 1.62E-27 | 1.05E-25 | down |
| ENSMUS( | Rpl35 | 513 | 498 | 429 | 716 | 718 | 768 | 0.616779 | 2.33E-27 | 1.49E-25 | down |
| ENSMUS( | Rps8 | 4133 | 3738 | 3243 | 5046 | 5165 | 5601 | 0.662343 | 2.77E-27 | 1.76E-25 | down |
| ENSMUS( | Dlgap3 | 2712 | 2658 | 2530 | 4669 | 3709 | 3437 | 0.629954 | 2.80E-27 | 1.77E-25 | down |
| ENSMUS( | Tst | 433 | 425 | 395 | 633 | 630 | 696 | 0.604871 | 2.83E-27 | 1.79E-25 | down |
| ENSMUS( | Epop | 295 | 302 | 258 | 522 | 494 | 474 | 0.540639 | 2.88E-27 | 1.81E-25 | down |
| ENSMUS( | Cmtm5 | 998 | 942 | 736 | 1381 | 1407 | 1597 | 0.574249 | 4.42E-27 | 2.75E-25 | down |
| ENSMUS( | Mrpl52 | 524 | 565 | 463 | 781 | 795 | 852 | 0.603411 | 5.90E-27 | 3.64E-25 | down |
| ENSMUS( | Cldn10 | 813 | 753 | 630 | 1055 | 1082 | 1141 | 0.630761 | 9.78E-27 | 5.96E-25 | down |
| ENSMUS( | D830030K | 0 | 1 | 1 | 34 | 40 | 43 | 0.016726 | 1.33E-26 | 8.04E-25 | down |
| ENSMUS( | Gng5 | 505 | 494 | 400 | 697 | 708 | 728 | 0.617433 | 1.77E-26 | 1.07E-24 | down |
| ENSMUS( | Anapc11 | 840 | 836 | 689 | 1068 | 1093 | 1269 | 0.65065 | 3.54E-26 | 2.09E-24 | down |
| ENSMUS( | Pvalb | 584 | 547 | 484 | 795 | 746 | 870 | 0.63204 | 5.60E-26 | 3.25E-24 | down |
| ENSMUS( | Fa2h | 437 | 400 | 348 | 610 | 623 | 613 | 0.604367 | 8.19E-26 | 4.70E-24 | down |
| ENSMUS( | Nop10 | 564 | 530 | 467 | 713 | 794 | 864 | 0.621687 | 9.37E-26 | 5.34E-24 | down |
| ENSMUS( | H2afx | 242 | 259 | 218 | 419 | 383 | 435 | 0.548823 | 1.13E-25 | 6.35E-24 | down |
| ENSMUS( | Chchd2 | 3285 | 3797 | 3454 | 5378 | 5729 | 5768 | 0.591716 | 1.17E-25 | 6.58E-24 | down |
| ENSMUS( | Jund | 277 | 275 | 230 | 487 | 462 | 432 | 0.53272 | 2.14E-25 | 1.18E-23 | down |
| ENSMUS( | Rida | 847 | 894 | 751 | 1122 | 1139 | 1275 | 0.665801 | 2.35E-25 | 1.29E-23 | down |
| ENSMUS( | Selplg | 692 | 698 | 602 | 931 | 993 | 934 | 0.65727 | 2.64E-25 | 1.45E-23 | down |
| ENSMUS( | Rpl28 | 2828 | 2516 | 2198 | 3553 | 3475 | 3793 | 0.656436 | 3.06E-25 | 1.67E-23 | down |
| ENSMUS( | Atp5d | 2588 | 2793 | 2647 | 4107 | 4176 | 4143 | 0.611804 | 3.58E-25 | 1.94E-23 | down |
| ENSMUS( | Atp5l | 730 | 719 | 674 | 930 | 1027 | 1122 | 0.652832 | 3.67E-25 | 1.99E-23 | down |
| ENSMUS( | Mgst3 | 1045 | 939 | 800 | 1336 | 1347 | 1486 | 0.628915 | 4.18E-25 | 2.25E-23 | down |
| ENSMUS( | Timm8b | 1090 | 1095 | 882 | 1440 | 1476 | 1527 | 0.650191 | 5.22E-25 | 2.77E-23 | down |
| ENSMUS( | Ndufa12 | 1368 | 1191 | 1138 | 1942 | 1782 | 1838 | 0.626464 | 1.05E-24 | 5.47E-23 | down |
| ENSMUS( | Myoc | 79 | 84 | 78 | 189 | 201 | 176 | 0.402439 | 1.80E-24 | 9.20E-23 | down |
| ENSMUS( | Emc6 | 513 | 525 | 424 | 919 | 708 | 735 | 0.581587 | 2.27E-24 | 1.15E-22 | down |
| ENSMUS( | Efhd1 | 345 | 341 | 299 | 549 | 504 | 522 | 0.589599 | 2.58E-24 | 1.30E-22 | down |
| ENSMUS( | Atat1 | 555 | 544 | 458 | 774 | 754 | 762 | 0.640358 | 2.66E-24 | 1.34E-22 | down |
| ENSMUS( | Dusp28 | 205 | 200 | 191 | 353 | 390 | 342 | 0.518886 | 3.22E-24 | 1.61E-22 | down |
| ENSMUS( | Mrps21 | 610 | 595 | 487 | 781 | 819 | 857 | 0.648765 | 3.69E-24 | 1.83E-22 | down |
| ENSMUS( | Stard10 | 401 | 379 | 348 | 594 | 547 | 602 | 0.610819 | 4.18E-24 | 2.07E-22 | down |
| ENSMUS( | Ndufa6 | 1129 | 1080 | 868 | 1472 | 1471 | 1651 | 0.630695 | 4.18E-24 | 2.07E-22 | down |
| ENSMUS( | Gabarap | 3464 | 3107 | 2680 | 4356 | 4226 | 4561 | 0.662665 | 5.26E-24 | 2.59E-22 | down |
| ENSMUS( | Serp2 | 868 | 820 | 710 | 1167 | 1050 | 1206 | 0.660326 | 5.69E-24 | 2.80E-22 | down |
| ENSMUS( | Fxyd7 | 595 | 573 | 451 | 838 | 821 | 911 | 0.592741 | 1.70E-23 | 8.13E-22 | down |
| ENSMUS( | Ndufa3 | 711 | 634 | 606 | 882 | 881 | 1024 | 0.661664 | 2.13E-23 | 1.01E-21 | down |
| ENSMUS( | Gltp | 527 | 468 | 430 | 668 | 781 | 727 | 0.617477 | 2.62E-23 | 1.24E-21 | down |
| ENSMUS( | Dbi | 3481 | 2879 | 2593 | 4613 | 4325 | 4846 | 0.611248 | 5.02E-23 | 2.32E-21 | down |
| ENSMUS( | Papss2 | 484 | 498 | 412 | 664 | 680 | 712 | 0.639477 | 7.76E-23 | 3.54E-21 | down |
| ENSMUS( | Chchd10 | 241 | 219 | 192 | 380 | 348 | 378 | 0.555119 | 1.77E-22 | 7.92E-21 | down |
| ENSMUS( | Cox5b | 3139 | 3347 | 3115 | 5287 | 5044 | 5015 | 0.591651 | 2.33E-22 | 1.04E-20 | down |
| ENSMUS( | Igsf21 | 257 | 276 | 230 | 432 | 390 | 425 | 0.577247 | 2.55E-22 | 1.13E-20 | down |
| ENSMUS( | Bri3 | 353 | 340 | 298 | 498 | 474 | 563 | 0.609583 | 5.72E-22 | 2.46E-20 | down |
| ENSMUS( | Gm1673 | 26 | 22 | 13 | 254 | 86 | 47 | 0.144359 | 5.98E-22 | 2.57E-20 | down |
| ENSMUS( | Cdk2ap1 | 419 | 415 | 360 | 566 | 589 | 632 | 0.630811 | 6.97E-22 | 2.98E-20 | down |
| ENSMUS( | S100a1 | 1222 | 1031 | 956 | 1532 | 1552 | 1778 | 0.622545 | 8.66E-22 | 3.66E-20 | down |
| ENSMUS( | C1ql1 | 132 | 111 | 111 | 241 | 225 | 232 | 0.478341 | 1.68E-21 | 6.94E-20 | down |
| ENSMUS( | Ier5 | 423 | 375 | 357 | 652 | 553 | 620 | 0.596778 | 1.84E-21 | 7.58E-20 | down |
| ENSMUS( | Ndufs6 | 643 | 572 | 497 | 838 | 778 | 872 | 0.647854 | 2.93E-21 | 1.17E-19 | down |

| | | | | | | | | | | | |
|---|---|---|---|---|---|---|---|---|---|---|---|
| ENSMUS( | mt-Nd3 | 194 | 194 | 169 | 293 | 316 | 367 | 0.539687 | 3.34E-21 | 1.34E-19 | down |
| ENSMUS( | Cabp1 | 490 | 494 | 466 | 704 | 781 | 693 | 0.629188 | 3.44E-21 | 1.37E-19 | down |
| ENSMUS( | Rps21 | 2832 | 2520 | 2202 | 3530 | 3684 | 4388 | 0.614297 | 4.52E-21 | 1.79E-19 | down |
| ENSMUS( | Polr2f | 357 | 316 | 278 | 482 | 505 | 477 | 0.611193 | 6.75E-21 | 2.64E-19 | down |
| ENSMUS( | Laptm5 | 699 | 619 | 514 | 899 | 949 | 995 | 0.606166 | 1.11E-20 | 4.28E-19 | down |
| ENSMUS( | Sox1 | 288 | 288 | 247 | 431 | 456 | 416 | 0.595167 | 1.35E-20 | 5.13E-19 | down |
| ENSMUS( | Gm2564 | 41 | 44 | 41 | 119 | 114 | 120 | 0.337855 | 2.03E-20 | 7.66E-19 | down |
| ENSMUS( | Hist1h2bc | 555 | 554 | 509 | 693 | 794 | 816 | 0.66472 | 2.82E-20 | 1.05E-18 | down |
| ENSMUS( | Camk2n2 | 778 | 868 | 807 | 1654 | 1497 | 1495 | 0.499495 | 3.19E-20 | 1.18E-18 | down |
| ENSMUS( | Cxx1b | 913 | 745 | 903 | 1338 | 1365 | 1481 | 0.581078 | 3.44E-20 | 1.28E-18 | down |
| ENSMUS( | Glrx5 | 471 | 476 | 406 | 607 | 670 | 674 | 0.654538 | 3.68E-20 | 1.36E-18 | down |
| ENSMUS( | 1110065P2 | 98 | 86 | 90 | 347 | 155 | 147 | 0.395585 | 4.61E-20 | 1.69E-18 | down |
| ENSMUS( | Ttc9b | 315 | 327 | 323 | 837 | 516 | 594 | 0.467923 | 6.76E-20 | 2.45E-18 | down |
| ENSMUS( | Atn1 | 172 | 165 | 131 | 274 | 268 | 288 | 0.530402 | 1.10E-19 | 3.94E-18 | down |
| ENSMUS( | Lrrc10b | 606 | 566 | 512 | 927 | 789 | 785 | 0.633808 | 1.27E-19 | 4.54E-18 | down |
| ENSMUS( | Sox21 | 156 | 155 | 120 | 281 | 238 | 273 | 0.511585 | 1.88E-19 | 6.60E-18 | down |
| ENSMUS( | Rpl13 | 3846 | 3938 | 3984 | 10069 | 8311 | 7037 | 0.437096 | 2.02E-19 | 7.07E-18 | down |
| ENSMUS( | Cacng4 | 404 | 386 | 329 | 525 | 551 | 567 | 0.641942 | 3.57E-19 | 1.22E-17 | down |
| ENSMUS( | Praf2 | 483 | 479 | 418 | 672 | 617 | 673 | 0.663482 | 4.10E-19 | 1.39E-17 | down |
| ENSMUS( | Ass1 | 432 | 389 | 393 | 537 | 605 | 614 | 0.654277 | 1.14E-18 | 3.73E-17 | down |
| ENSMUS( | Taf10 | 341 | 304 | 283 | 481 | 430 | 495 | 0.622606 | 1.19E-18 | 3.88E-17 | down |
| ENSMUS( | Uqcrq | 2194 | 1951 | 1688 | 2665 | 2683 | 3131 | 0.648472 | 1.60E-18 | 5.17E-17 | down |
| ENSMUS( | AI413582 | 485 | 461 | 373 | 640 | 595 | 678 | 0.648937 | 2.18E-18 | 6.97E-17 | down |
| ENSMUS( | Akt1s1 | 586 | 569 | 482 | 907 | 723 | 784 | 0.637898 | 2.65E-18 | 8.39E-17 | down |
| ENSMUS( | Megf11 | 466 | 489 | 454 | 658 | 656 | 715 | 0.657481 | 3.39E-18 | 1.06E-16 | down |
| ENSMUS( | Garem2 | 254 | 269 | 232 | 401 | 390 | 381 | 0.607805 | 4.63E-18 | 1.43E-16 | down |
| ENSMUS( | Emc10 | 1598 | 1753 | 1628 | 2343 | 2402 | 2410 | 0.658946 | 4.94E-18 | 1.52E-16 | down |
| ENSMUS( | Trim8 | 1044 | 1163 | 1068 | 1621 | 1607 | 1589 | 0.643464 | 6.62E-18 | 2.01E-16 | down |
| ENSMUS( | Znhit1 | 458 | 439 | 375 | 644 | 616 | 593 | 0.645989 | 6.91E-18 | 2.10E-16 | down |
| ENSMUS( | Jup | 356 | 322 | 308 | 486 | 491 | 475 | 0.640666 | 9.77E-18 | 2.93E-16 | down |
| ENSMUS( | Dnlz | 442 | 459 | 370 | 776 | 594 | 576 | 0.613302 | 1.44E-17 | 4.27E-16 | down |
| ENSMUS( | Krtcap2 | 455 | 419 | 368 | 568 | 559 | 632 | 0.66592 | 2.02E-17 | 5.94E-16 | down |
| ENSMUS( | Znhit2 | 304 | 280 | 254 | 566 | 404 | 411 | 0.570164 | 2.92E-17 | 8.43E-16 | down |
| ENSMUS( | Gm5741 | 3 | 3 | 1 | 24 | 31 | 43 | 0.066386 | 3.24E-17 | 9.27E-16 | down |
| ENSMUS( | Arhgdig | 499 | 525 | 394 | 747 | 689 | 745 | 0.611475 | 4.09E-17 | 1.16E-15 | down |
| ENSMUS( | Pou4f1 | 0 | 1 | 0 | 26 | 18 | 28 | 0.012837 | 4.77E-17 | 1.34E-15 | down |
| ENSMUS( | Evi2a | 360 | 351 | 335 | 546 | 482 | 523 | 0.637253 | 5.10E-17 | 1.43E-15 | down |
| ENSMUS( | Akr1e1 | 199 | 164 | 162 | 294 | 288 | 285 | 0.570663 | 6.30E-17 | 1.75E-15 | down |
| ENSMUS( | Hes5 | 39 | 34 | 39 | 95 | 109 | 96 | 0.353852 | 1.13E-16 | 3.09E-15 | down |
| ENSMUS( | Plekho1 | 363 | 327 | 293 | 489 | 449 | 487 | 0.649858 | 1.88E-16 | 5.03E-15 | down |
| ENSMUS( | Dbx2 | 445 | 411 | 339 | 535 | 579 | 585 | 0.661945 | 3.62E-16 | 9.50E-15 | down |
| ENSMUS( | Cox7c | 3577 | 3176 | 2774 | 4354 | 4178 | 5033 | 0.662135 | 4.55E-16 | 1.19E-14 | down |
| ENSMUS( | Nptx2 | 312 | 303 | 256 | 422 | 431 | 427 | 0.640967 | 5.73E-16 | 1.48E-14 | down |
| ENSMUS( | Lmo1 | 457 | 410 | 377 | 579 | 625 | 564 | 0.662859 | 7.35E-16 | 1.88E-14 | down |
| ENSMUS( | Cisd3 | 249 | 242 | 198 | 332 | 347 | 377 | 0.614893 | 7.36E-16 | 1.88E-14 | down |
| ENSMUS( | Ndufa11 | 1531 | 1309 | 1187 | 1765 | 1845 | 2094 | 0.665817 | 7.64E-16 | 1.95E-14 | down |
| ENSMUS( | Gjb1 | 165 | 124 | 129 | 258 | 268 | 261 | 0.500453 | 8.96E-16 | 2.27E-14 | down |
| ENSMUS( | Tspan15 | 329 | 340 | 300 | 439 | 474 | 480 | 0.657375 | 1.16E-15 | 2.89E-14 | down |
| ENSMUS( | Plekho2 | 280 | 267 | 260 | 387 | 398 | 408 | 0.639789 | 1.83E-15 | 4.54E-14 | down |
| ENSMUS( | Mrpl23 | 487 | 473 | 399 | 622 | 585 | 737 | 0.659907 | 2.36E-15 | 5.76E-14 | down |
| ENSMUS( | Fam43b | 121 | 130 | 115 | 213 | 191 | 242 | 0.536383 | 2.70E-15 | 6.56E-14 | down |
| ENSMUS( | Pdcd7 | 351 | 388 | 330 | 497 | 487 | 560 | 0.6549 | 2.87E-15 | 6.95E-14 | down |
| ENSMUS( | Rgs10 | 562 | 460 | 391 | 718 | 712 | 766 | 0.604478 | 3.12E-15 | 7.53E-14 | down |
| ENSMUS( | Chchd1 | 424 | 359 | 355 | 533 | 532 | 697 | 0.611305 | 3.24E-15 | 7.80E-14 | down |
| ENSMUS( | Pcdh8 | 546 | 568 | 434 | 695 | 795 | 831 | 0.628488 | 3.28E-15 | 7.88E-14 | down |
| ENSMUS( | Fbll1 | 366 | 331 | 263 | 483 | 470 | 539 | 0.605008 | 3.37E-15 | 8.09E-14 | down |
| ENSMUS( | Gpr34 | 290 | 293 | 263 | 397 | 397 | 502 | 0.618169 | 7.15E-15 | 1.67E-13 | down |
| ENSMUS( | Zfp385a | 631 | 624 | 630 | 876 | 923 | 894 | 0.662923 | 7.64E-15 | 1.78E-13 | down |
| ENSMUS( | Fbxo2 | 1026 | 935 | 985 | 1752 | 1548 | 1563 | 0.572758 | 8.57E-15 | 1.98E-13 | down |
| ENSMUS( | Trappc1 | 594 | 493 | 449 | 727 | 764 | 741 | 0.647453 | 1.45E-14 | 3.28E-13 | down |
| ENSMUS( | Resp18 | 337 | 312 | 273 | 437 | 407 | 468 | 0.662549 | 2.32E-14 | 5.16E-13 | down |
| ENSMUS( | Mpv17l2 | 170 | 147 | 137 | 245 | 233 | 263 | 0.577814 | 2.93E-14 | 6.44E-13 | down |
| ENSMUS( | Mrps15 | 443 | 464 | 397 | 719 | 511 | 662 | 0.650134 | 3.70E-14 | 8.07E-13 | down |
| ENSMUS( | Opalin | 391 | 343 | 276 | 512 | 490 | 553 | 0.610327 | 5.80E-14 | 1.25E-12 | down |
| ENSMUS( | Ppp1r14a | 83 | 78 | 57 | 212 | 125 | 145 | 0.422462 | 5.82E-14 | 1.25E-12 | down |
| ENSMUS( | Lxn | 619 | 528 | 441 | 761 | 767 | 805 | 0.639613 | 6.65E-14 | 1.42E-12 | down |
| ENSMUS( | Nenf | 445 | 451 | 411 | 696 | 593 | 578 | 0.660047 | 6.95E-14 | 1.49E-12 | down |
| ENSMUS( | Fam181b | 81 | 68 | 57 | 138 | 123 | 153 | 0.46788 | 7.19E-14 | 1.53E-12 | down |
| ENSMUS( | S100a13 | 389 | 322 | 269 | 512 | 489 | 545 | 0.595412 | 9.03E-14 | 1.91E-12 | down |
| ENSMUS( | Tcf7l2 | 125 | 116 | 109 | 183 | 203 | 216 | 0.549672 | 1.09E-13 | 2.30E-12 | down |
| ENSMUS( | Zfp703 | 245 | 262 | 238 | 359 | 351 | 369 | 0.653139 | 3.12E-13 | 6.33E-12 | down |
| ENSMUS( | Tmem158 | 223 | 208 | 217 | 378 | 347 | 314 | 0.589249 | 3.17E-13 | 6.41E-12 | down |
| ENSMUS( | Tac2 | 86 | 104 | 104 | 201 | 199 | 209 | 0.459085 | 3.44E-13 | 6.92E-12 | down |
| ENSMUS( | Emx1 | 120 | 123 | 105 | 196 | 197 | 198 | 0.555419 | 4.87E-13 | 9.63E-12 | down |
| ENSMUS( | Pgp | 423 | 458 | 410 | 766 | 616 | 562 | 0.625801 | 6.07E-13 | 1.19E-11 | down |
| ENSMUS( | Ttpa | 169 | 154 | 159 | 228 | 259 | 269 | 0.604134 | 6.69E-13 | 1.30E-11 | down |
| ENSMUS( | Trem2 | 221 | 183 | 173 | 285 | 268 | 318 | 0.624746 | 7.75E-13 | 1.50E-11 | down |
| ENSMUS( | Tlcd1 | 283 | 274 | 219 | 370 | 347 | 392 | 0.658587 | 8.16E-13 | 1.58E-11 | down |
| ENSMUS( | Ndp | 203 | 193 | 166 | 271 | 267 | 333 | 0.609351 | 1.09E-12 | 2.08E-11 | down |
| ENSMUS( | Pgls | 277 | 290 | 221 | 517 | 395 | 381 | 0.571452 | 1.12E-12 | 2.13E-11 | down |

| | | | | | | | | | | |
|---|---|---|---|---|---|---|---|---|---|---|
| ENSMUS( | Rps19bp1 | 243 | 236 | 219 | 382 | 337 | 335 | 0.62454 | 1.26E-12 | 2.39E-11 down |
| ENSMUS( | 2310036O | 627 | 544 | 557 | 1154 | 817 | 701 | 0.607755 | 1.28E-12 | 2.43E-11 down |
| ENSMUS( | Ypel3 | 1840 | 1619 | 1419 | 2552 | 2155 | 2280 | 0.656216 | 1.28E-12 | 2.43E-11 down |
| ENSMUS( | Mospd3 | 215 | 197 | 175 | 318 | 280 | 293 | 0.620173 | 1.40E-12 | 2.65E-11 down |
| ENSMUS( | Lims2 | 115 | 133 | 101 | 206 | 184 | 192 | 0.564741 | 2.94E-12 | 5.37E-11 down |
| ENSMUS( | F8a | 178 | 204 | 166 | 291 | 316 | 265 | 0.592464 | 3.16E-12 | 5.75E-11 down |
| ENSMUS( | Tbcc | 187 | 202 | 179 | 305 | 259 | 300 | 0.621421 | 4.03E-12 | 7.23E-11 down |
| ENSMUS( | Mrps34 | 17 | 13 | 22 | 231 | 57 | 28 | 0.154417 | 6.77E-12 | 1.19E-10 down |
| ENSMUS( | Cdc42ep2 | 127 | 120 | 93 | 192 | 177 | 197 | 0.564444 | 6.95E-12 | 1.22E-10 down |
| ENSMUS( | Gamt | 168 | 178 | 138 | 285 | 275 | 229 | 0.575991 | 7.39E-12 | 1.30E-10 down |
| ENSMUS( | Prkcdbp | 213 | 189 | 170 | 307 | 276 | 277 | 0.625723 | 8.73E-12 | 1.52E-10 down |
| ENSMUS( | Frs3 | 214 | 204 | 191 | 268 | 293 | 339 | 0.640621 | 1.15E-11 | 1.98E-10 down |
| ENSMUS( | Pam16 | 239 | 195 | 177 | 305 | 308 | 319 | 0.616758 | 1.16E-11 | 2.00E-10 down |
| ENSMUS( | Pcdhb9 | 127 | 116 | 115 | 179 | 205 | 200 | 0.579816 | 1.43E-11 | 2.42E-10 down |
| ENSMUS( | Coq4 | 248 | 228 | 181 | 352 | 340 | 311 | 0.614372 | 1.45E-11 | 2.46E-10 down |
| ENSMUS( | Ccdc153 | 133 | 105 | 99 | 198 | 166 | 193 | 0.569131 | 1.68E-11 | 2.82E-10 down |
| ENSMUS( | Gp1bb | 55 | 59 | 37 | 141 | 104 | 115 | 0.392029 | 2.38E-11 | 3.94E-10 down |
| ENSMUS( | Olfml3 | 183 | 196 | 173 | 250 | 288 | 278 | 0.639757 | 2.50E-11 | 4.12E-10 down |
| ENSMUS( | Rgcc | 354 | 313 | 293 | 583 | 393 | 416 | 0.647928 | 3.48E-11 | 5.66E-10 down |
| ENSMUS( | Gng11 | 195 | 207 | 163 | 272 | 273 | 284 | 0.64216 | 3.63E-11 | 5.86E-10 down |
| ENSMUS( | Elovl1 | 224 | 234 | 207 | 326 | 298 | 323 | 0.663262 | 3.64E-11 | 5.88E-10 down |
| ENSMUS( | Ascl1 | 92 | 76 | 61 | 162 | 144 | 131 | 0.49012 | 3.68E-11 | 5.93E-10 down |
| ENSMUS( | Ppp1r14b | 237 | 199 | 210 | 283 | 331 | 332 | 0.646239 | 4.02E-11 | 6.44E-10 down |
| ENSMUS( | BC026585 | 195 | 174 | 155 | 233 | 274 | 270 | 0.635867 | 5.69E-11 | 8.97E-10 down |
| ENSMUS( | Zfp580 | 102 | 90 | 112 | 346 | 235 | 217 | 0.36036 | 6.86E-11 | 1.07E-09 down |
| ENSMUS( | Lsm4 | 280 | 297 | 280 | 475 | 454 | 356 | 0.629159 | 7.05E-11 | 1.10E-09 down |
| ENSMUS( | Gm5617 | 35 | 44 | 35 | 102 | 76 | 83 | 0.411951 | 9.45E-11 | 1.46E-09 down |
| ENSMUS( | Sac3d1 | 102 | 99 | 92 | 167 | 165 | 159 | 0.563215 | 1.04E-10 | 1.60E-09 down |
| ENSMUS( | Tmem160 | 174 | 157 | 151 | 226 | 288 | 243 | 0.601117 | 1.18E-10 | 1.79E-09 down |
| ENSMUS( | Gmds | 139 | 141 | 118 | 182 | 217 | 221 | 0.606136 | 1.24E-10 | 1.88E-09 down |
| ENSMUS( | Fam163a | 92 | 84 | 90 | 154 | 156 | 145 | 0.553235 | 1.41E-10 | 2.13E-09 down |
| ENSMUS( | Lmcd1 | 74 | 80 | 72 | 135 | 123 | 145 | 0.530841 | 1.63E-10 | 2.44E-09 down |
| ENSMUS( | Nkx6-2 | 67 | 69 | 63 | 159 | 106 | 132 | 0.472962 | 1.67E-10 | 2.49E-09 down |
| ENSMUS( | Foxo6 | 250 | 246 | 170 | 371 | 336 | 350 | 0.59019 | 1.72E-10 | 2.56E-09 down |
| ENSMUS( | Chrna3 | 24 | 23 | 24 | 58 | 56 | 69 | 0.368348 | 2.50E-10 | 3.66E-09 down |
| ENSMUS( | Ppp2r3d | 228 | 264 | 228 | 390 | 399 | 342 | 0.601081 | 2.84E-10 | 4.13E-09 down |
| ENSMUS( | Dnajc30 | 192 | 166 | 140 | 241 | 231 | 260 | 0.639875 | 4.08E-10 | 5.84E-09 down |
| ENSMUS( | Il17d | 110 | 106 | 74 | 169 | 174 | 168 | 0.53169 | 4.26E-10 | 6.07E-09 down |
| ENSMUS( | Mvd | 300 | 249 | 223 | 338 | 384 | 443 | 0.624969 | 4.33E-10 | 6.17E-09 down |
| ENSMUS( | Slc2a5 | 51 | 49 | 60 | 108 | 107 | 128 | 0.444971 | 4.66E-10 | 6.61E-09 down |
| ENSMUS( | Gpr151 | 35 | 39 | 25 | 73 | 76 | 74 | 0.416271 | 5.17E-10 | 7.30E-09 down |
| ENSMUS( | Pigyl | 213 | 215 | 174 | 293 | 266 | 293 | 0.665375 | 5.44E-10 | 7.65E-09 down |
| ENSMUS( | Samd1 | 231 | 282 | 212 | 370 | 356 | 381 | 0.617898 | 5.68E-10 | 7.97E-09 down |
| ENSMUS( | Plxdc1 | 126 | 135 | 115 | 191 | 193 | 199 | 0.609062 | 5.88E-10 | 8.23E-09 down |
| ENSMUS( | Mpc1 | 497 | 431 | 356 | 650 | 611 | 598 | 0.648066 | 6.99E-10 | 9.74E-09 down |
| ENSMUS( | Borcs6 | 230 | 234 | 215 | 355 | 277 | 360 | 0.647263 | 8.07E-10 | 1.12E-08 down |
| ENSMUS( | Mypop | 199 | 198 | 164 | 252 | 307 | 267 | 0.639786 | 8.23E-10 | 1.14E-08 down |
| ENSMUS( | Arhgef19 | 165 | 148 | 154 | 204 | 256 | 240 | 0.631882 | 1.20E-09 | 1.62E-08 down |
| ENSMUS( | Phlda1 | 84 | 98 | 69 | 137 | 148 | 149 | 0.544456 | 1.28E-09 | 1.73E-08 down |
| ENSMUS( | D030056L | 373 | 394 | 298 | 529 | 512 | 480 | 0.657904 | 1.32E-09 | 1.78E-08 down |
| ENSMUS( | Zfp428 | 156 | 165 | 179 | 386 | 315 | 303 | 0.472059 | 1.33E-09 | 1.79E-08 down |
| ENSMUS( | Mid1 | 151 | 163 | 136 | 198 | 240 | 225 | 0.641067 | 2.11E-09 | 2.76E-08 down |
| ENSMUS( | Tmem121 | 177 | 148 | 136 | 302 | 210 | 218 | 0.59235 | 2.59E-09 | 3.34E-08 down |
| ENSMUS( | Akr7a5 | 270 | 262 | 196 | 415 | 333 | 393 | 0.598859 | 3.52E-09 | 4.50E-08 down |
| ENSMUS( | Gm266 | 44 | 31 | 38 | 90 | 75 | 71 | 0.451514 | 3.94E-09 | 5.00E-08 down |
| ENSMUS( | Ier5l | 86 | 86 | 87 | 193 | 142 | 128 | 0.52759 | 4.22E-09 | 5.33E-08 down |
| ENSMUS( | Gm13889 | 220 | 246 | 229 | 333 | 353 | 316 | 0.656413 | 4.84E-09 | 6.07E-08 down |
| ENSMUS( | Btbd17 | 153 | 153 | 120 | 238 | 200 | 209 | 0.618504 | 4.99E-09 | 6.24E-08 down |
| ENSMUS( | Scand1 | 5 | 3 | 8 | 146 | 12 | 9 | 0.090304 | 5.04E-09 | 6.30E-08 down |
| ENSMUS( | Lrrc55 | 178 | 178 | 156 | 261 | 239 | 233 | 0.658167 | 5.81E-09 | 7.20E-08 down |
| ENSMUS( | Cib1 | 256 | 207 | 190 | 309 | 313 | 311 | 0.658209 | 7.10E-09 | 8.66E-08 down |
| ENSMUS( | Zbed3 | 178 | 184 | 156 | 275 | 259 | 227 | 0.640599 | 1.24E-08 | 1.46E-07 down |
| ENSMUS( | Ost4 | 74 | 66 | 61 | 98 | 117 | 132 | 0.547714 | 1.46E-08 | 1.70E-07 down |
| ENSMUS( | mt-Atp8 | 57 | 61 | 61 | 96 | 106 | 115 | 0.536483 | 1.87E-08 | 2.14E-07 down |
| ENSMUS( | Gas1 | 152 | 161 | 139 | 225 | 209 | 215 | 0.65724 | 1.92E-08 | 2.20E-07 down |
| ENSMUS( | Bmyc | 57 | 44 | 51 | 95 | 81 | 105 | 0.512411 | 2.01E-08 | 2.30E-07 down |
| ENSMUS( | Mrpl34 | 278 | 269 | 194 | 322 | 350 | 395 | 0.652815 | 2.42E-08 | 2.75E-07 down |
| ENSMUS( | Inhba | 149 | 131 | 118 | 186 | 189 | 209 | 0.642594 | 2.49E-08 | 2.83E-07 down |
| ENSMUS( | Gm3248 | 2 | 2 | 2 | 14 | 15 | 22 | 0.112025 | 2.80E-08 | 3.15E-07 down |
| ENSMUS( | Fxn | 90 | 91 | 94 | 153 | 146 | 135 | 0.599875 | 3.43E-08 | 3.83E-07 down |
| ENSMUS( | E130114P | 126 | 119 | 97 | 203 | 162 | 172 | 0.597982 | 3.53E-08 | 3.93E-07 down |
| ENSMUS( | Fam185a | 134 | 148 | 121 | 200 | 182 | 205 | 0.648027 | 3.62E-08 | 4.04E-07 down |
| ENSMUS( | Chadl | 121 | 93 | 87 | 170 | 158 | 150 | 0.591036 | 3.64E-08 | 4.06E-07 down |
| ENSMUS( | Yjefn3 | 103 | 112 | 78 | 163 | 158 | 148 | 0.586066 | 3.74E-08 | 4.16E-07 down |
| ENSMUS( | Gabra6 | 0 | 1 | 0 | 12 | 14 | 9 | 0.026268 | 3.81E-08 | 4.22E-07 down |
| ENSMUS( | Rnf126 | 543 | 616 | 545 | 955 | 781 | 790 | 0.63693 | 5.82E-08 | 6.28E-07 down |
| ENSMUS( | Pigx | 183 | 198 | 160 | 330 | 229 | 239 | 0.637036 | 5.94E-08 | 6.40E-07 down |
| ENSMUS( | Amn | 32 | 23 | 18 | 86 | 51 | 49 | 0.364438 | 7.01E-08 | 7.46E-07 down |
| ENSMUS( | Irx2 | 1 | 3 | 2 | 15 | 28 | 11 | 0.105385 | 7.42E-08 | 7.86E-07 down |
| ENSMUS( | Sap30l | 188 | 167 | 160 | 277 | 208 | 264 | 0.648681 | 8.75E-08 | 9.20E-07 down |

| | | | | | | | | | | | |
|---|---|---|---|---|---|---|---|---|---|---|---|
| ENSMUS( | Itgam | 143 | 149 | 160 | 258 | 225 | 232 | 0.599769 | 8.82E-08 | 9.27E-07 | down |
| ENSMUS( | H1fx | 50 | 56 | 80 | 126 | 152 | 134 | 0.433271 | 9.16E-08 | 9.61E-07 | down |
| ENSMUS( | Fgfbp3 | 92 | 88 | 87 | 129 | 144 | 145 | 0.604787 | 9.69E-08 | 1.01E-06 | down |
| ENSMUS( | Nr2f6 | 56 | 52 | 48 | 120 | 84 | 92 | 0.495859 | 1.01E-07 | 1.05E-06 | down |
| ENSMUS( | Krt1 | 50 | 54 | 61 | 106 | 97 | 109 | 0.50365 | 1.01E-07 | 1.05E-06 | down |
| ENSMUS( | Pth1r | 72 | 76 | 63 | 111 | 108 | 129 | 0.572832 | 1.15E-07 | 1.19E-06 | down |
| ENSMUS( | Nkx2-2 | 46 | 49 | 33 | 77 | 84 | 79 | 0.500298 | 1.23E-07 | 1.26E-06 | down |
| ENSMUS( | Gjc2 | 51 | 49 | 42 | 102 | 73 | 92 | 0.500695 | 1.34E-07 | 1.36E-06 | down |
| ENSMUS( | Zfp771 | 67 | 77 | 92 | 439 | 127 | 156 | 0.308564 | 1.49E-07 | 1.51E-06 | down |
| ENSMUS( | Ppp1r18 | 181 | 155 | 151 | 245 | 200 | 245 | 0.665956 | 1.82E-07 | 1.82E-06 | down |
| ENSMUS( | Prr7 | 165 | 210 | 215 | 394 | 328 | 351 | 0.522961 | 2.30E-07 | 2.27E-06 | down |
| ENSMUS( | Fbxl15 | 85 | 79 | 92 | 201 | 168 | 115 | 0.499358 | 2.44E-07 | 2.40E-06 | down |
| ENSMUS( | Cbr3 | 142 | 145 | 131 | 247 | 173 | 208 | 0.627803 | 2.56E-07 | 2.51E-06 | down |
| ENSMUS( | Mien1 | 99 | 93 | 86 | 132 | 130 | 189 | 0.58433 | 2.92E-07 | 2.84E-06 | down |
| ENSMUS( | Frat1 | 144 | 158 | 151 | 208 | 240 | 202 | 0.659853 | 2.94E-07 | 2.85E-06 | down |
| ENSMUS( | Srprb | 255 | 197 | 202 | 335 | 281 | 355 | 0.635442 | 3.49E-07 | 3.34E-06 | down |
| ENSMUS( | Tysnd1 | 153 | 184 | 134 | 237 | 213 | 246 | 0.637916 | 4.16E-07 | 3.94E-06 | down |
| ENSMUS( | Psmg4 | 143 | 112 | 117 | 156 | 197 | 186 | 0.651925 | 4.21E-07 | 3.98E-06 | down |
| ENSMUS( | Paqr6 | 224 | 197 | 148 | 255 | 318 | 297 | 0.613619 | 4.21E-07 | 3.99E-06 | down |
| ENSMUS( | Pradc1 | 136 | 114 | 126 | 179 | 177 | 185 | 0.657862 | 4.43E-07 | 4.17E-06 | down |
| ENSMUS( | Rps2-ps6 | 20 | 16 | 15 | 42 | 48 | 35 | 0.382914 | 4.83E-07 | 4.52E-06 | down |
| ENSMUS( | Cbln1 | 76 | 83 | 52 | 126 | 105 | 140 | 0.533681 | 5.81E-07 | 5.37E-06 | down |
| ENSMUS( | Otx1 | 64 | 65 | 61 | 85 | 110 | 125 | 0.56364 | 7.08E-07 | 6.46E-06 | down |
| ENSMUS( | Rnaseh2c | 114 | 166 | 151 | 309 | 260 | 259 | 0.494182 | 8.45E-07 | 7.63E-06 | down |
| ENSMUS( | Kcne1l | 92 | 136 | 103 | 160 | 193 | 200 | 0.567749 | 1.08E-06 | 9.63E-06 | down |
| ENSMUS( | Phlda3 | 146 | 107 | 109 | 206 | 169 | 189 | 0.603648 | 1.36E-06 | 1.19E-05 | down |
| ENSMUS( | Gm6710 | 90 | 74 | 75 | 123 | 120 | 124 | 0.614439 | 1.40E-06 | 1.22E-05 | down |
| ENSMUS( | Ebpl | 204 | 179 | 210 | 335 | 315 | 283 | 0.6019 | 1.43E-06 | 1.25E-05 | down |
| ENSMUS( | Ethe1 | 134 | 111 | 108 | 170 | 160 | 171 | 0.66416 | 1.50E-06 | 1.30E-05 | down |
| ENSMUS( | Mfap4 | 112 | 90 | 94 | 149 | 134 | 151 | 0.643893 | 1.59E-06 | 1.38E-05 | down |
| ENSMUS( | Lsm2 | 93 | 95 | 67 | 147 | 131 | 117 | 0.604245 | 1.86E-06 | 1.60E-05 | down |
| ENSMUS( | Lsm3 | 202 | 206 | 143 | 251 | 248 | 280 | 0.664308 | 2.24E-06 | 1.90E-05 | down |
| ENSMUS( | Reep6 | 112 | 106 | 85 | 128 | 142 | 170 | 0.649456 | 2.28E-06 | 1.92E-05 | down |
| ENSMUS( | Thbs4 | 105 | 104 | 86 | 132 | 155 | 141 | 0.649314 | 3.16E-06 | 2.61E-05 | down |
| ENSMUS( | Nog | 103 | 120 | 104 | 142 | 158 | 165 | 0.666227 | 3.22E-06 | 2.66E-05 | down |
| ENSMUS( | Mocs3 | 121 | 87 | 88 | 167 | 153 | 142 | 0.601518 | 3.25E-06 | 2.68E-05 | down |
| ENSMUS( | Fam20a | 125 | 155 | 116 | 176 | 214 | 189 | 0.645327 | 5.51E-06 | 4.40E-05 | down |
| ENSMUS( | Cyp26b1 | 121 | 115 | 86 | 140 | 156 | 159 | 0.664944 | 5.86E-06 | 4.65E-05 | down |
| ENSMUS( | Gm45844 | 31 | 40 | 30 | 67 | 56 | 62 | 0.515042 | 5.99E-06 | 4.75E-05 | down |
| ENSMUS( | Acta1 | 104 | 86 | 73 | 116 | 133 | 137 | 0.640755 | 6.01E-06 | 4.76E-05 | down |
| ENSMUS( | Thoc6 | 68 | 66 | 53 | 81 | 101 | 120 | 0.584874 | 6.37E-06 | 5.02E-05 | down |
| ENSMUS( | Dohh | 115 | 113 | 85 | 140 | 143 | 160 | 0.664618 | 7.21E-06 | 5.64E-05 | down |
| ENSMUS( | Ppp1r35 | 127 | 87 | 81 | 160 | 160 | 155 | 0.581914 | 7.43E-06 | 5.78E-05 | down |
| ENSMUS( | Acot1 | 285 | 321 | 234 | 438 | 346 | 429 | 0.651709 | 7.76E-06 | 6.02E-05 | down |
| ENSMUS( | Gal3st1 | 141 | 126 | 109 | 146 | 206 | 186 | 0.658829 | 8.18E-06 | 6.30E-05 | down |
| ENSMUS( | Naa38 | 150 | 173 | 164 | 210 | 276 | 237 | 0.639122 | 8.66E-06 | 6.64E-05 | down |
| ENSMUS( | Gm8281 | 8 | 3 | 0 | 31 | 30 | 34 | 0.104095 | 1.00E-05 | 7.60E-05 | down |
| ENSMUS( | Dctpp1 | 100 | 81 | 110 | 171 | 147 | 156 | 0.583585 | 1.13E-05 | 8.46E-05 | down |
| ENSMUS( | Mettl11b | 22 | 21 | 20 | 47 | 42 | 41 | 0.457163 | 1.24E-05 | 9.24E-05 | down |
| ENSMUS( | Dmkn | 24 | 19 | 10 | 40 | 50 | 44 | 0.367017 | 1.26E-05 | 9.35E-05 | down |
| ENSMUS( | Susd3 | 21 | 19 | 14 | 44 | 41 | 32 | 0.430985 | 1.32E-05 | 9.79E-05 | down |
| ENSMUS( | Krt15 | 15 | 20 | 15 | 34 | 42 | 35 | 0.425375 | 1.43E-05 | 0.000105 | down |
| ENSMUS( | Gm3468 | 0 | 2 | 1 | 8 | 11 | 11 | 0.096107 | 1.52E-05 | 0.000111 | down |
| ENSMUS( | Fam181a | 26 | 20 | 25 | 50 | 47 | 43 | 0.479875 | 1.59E-05 | 0.000116 | down |
| ENSMUS( | Hgh1 | 97 | 118 | 108 | 155 | 143 | 172 | 0.652573 | 1.67E-05 | 0.000121 | down |
| ENSMUS( | Gadd45gip | 50 | 48 | 42 | 144 | 56 | 53 | 0.516058 | 1.71E-05 | 0.000124 | down |
| ENSMUS( | Aqp9 | 15 | 17 | 9 | 30 | 40 | 27 | 0.393915 | 1.76E-05 | 0.000127 | down |
| ENSMUS( | Smim18 | 66 | 71 | 52 | 91 | 90 | 109 | 0.614123 | 1.85E-05 | 0.000133 | down |
| ENSMUS( | Ifi202b | 2 | 5 | 2 | 15 | 9 | 19 | 0.197351 | 2.05E-05 | 0.000146 | down |
| ENSMUS( | Angptl6 | 68 | 66 | 56 | 95 | 92 | 103 | 0.617808 | 2.20E-05 | 0.000156 | down |
| ENSMUS( | Insm1 | 72 | 91 | 66 | 119 | 111 | 118 | 0.620398 | 2.24E-05 | 0.000158 | down |
| ENSMUS( | Oxld1 | 83 | 64 | 70 | 117 | 121 | 93 | 0.617267 | 2.45E-05 | 0.000172 | down |
| ENSMUS( | Polr2i | 242 | 184 | 187 | 292 | 250 | 340 | 0.656079 | 2.51E-05 | 0.000176 | down |
| ENSMUS( | Rprml | 505 | 517 | 550 | 902 | 730 | 721 | 0.632627 | 2.76E-05 | 0.000192 | down |
| ENSMUS( | A230065H | 36 | 38 | 23 | 48 | 62 | 62 | 0.528762 | 2.99E-05 | 0.000207 | down |
| ENSMUS( | Tmem114 | 28 | 20 | 26 | 55 | 42 | 44 | 0.496081 | 3.17E-05 | 0.000219 | down |
| ENSMUS( | Emid1 | 68 | 52 | 50 | 84 | 93 | 86 | 0.60793 | 3.35E-05 | 0.00023 | down |
| ENSMUS( | Gm2237 | 6 | 10 | 4 | 20 | 19 | 24 | 0.297161 | 3.36E-05 | 0.00023 | down |
| ENSMUS( | Gm10762 | 47 | 47 | 52 | 62 | 90 | 86 | 0.584287 | 3.52E-05 | 0.00024 | down |
| ENSMUS( | DHRSX | 26 | 38 | 37 | 78 | 74 | 55 | 0.463196 | 3.89E-05 | 0.000264 | down |
| ENSMUS( | Endog | 23 | 29 | 16 | 70 | 45 | 35 | 0.421355 | 4.23E-05 | 0.000285 | down |
| ENSMUS( | Otos | 1 | 3 | 3 | 8 | 12 | 17 | 0.183784 | 4.48E-05 | 0.0003 | down |
| ENSMUS( | AC123954 | 11 | 9 | 9 | 26 | 18 | 31 | 0.36574 | 5.07E-05 | 0.000337 | down |
| ENSMUS( | Wnt10a | 37 | 36 | 33 | 71 | 52 | 59 | 0.548899 | 5.24E-05 | 0.000347 | down |
| ENSMUS( | Slc5a7 | 9 | 13 | 16 | 30 | 35 | 31 | 0.37984 | 5.63E-05 | 0.000371 | down |
| ENSMUS( | Rpl13a | 98 | 81 | 97 | 125 | 141 | 136 | 0.651417 | 5.84E-05 | 0.000384 | down |
| ENSMUS( | Avil | 1 | 1 | 1 | 8 | 8 | 11 | 0.105639 | 6.61E-05 | 0.00043 | down |
| ENSMUS( | Gm11127 | 18 | 8 | 7 | 36 | 36 | 30 | 0.299195 | 7.37E-05 | 0.000474 | down |
| ENSMUS( | Btnl2 | 0 | 1 | 0 | 6 | 7 | 8 | 0.044072 | 7.83E-05 | 0.000501 | down |
| ENSMUS( | Medag | 33 | 24 | 25 | 48 | 57 | 42 | 0.524212 | 8.59E-05 | 0.000545 | down |

| | | | | | | | | | | | |
|---|---|---|---|---|---|---|---|---|---|---|---|
| ENSMUS( | Dmrta2 | 15 | 25 | 24 | 51 | 44 | 42 | 0.445101 | 9.22E-05 | 0.000582 | down |
| ENSMUS( | Hist1h2be | 70 | 63 | 60 | 81 | 95 | 106 | 0.647608 | 0.000123 | 0.000757 | down |
| ENSMUS( | Vwc2 | 54 | 55 | 48 | 77 | 69 | 92 | 0.624036 | 0.000141 | 0.000859 | down |
| ENSMUS( | Gm12355 | 54 | 50 | 46 | 67 | 70 | 104 | 0.590271 | 0.000142 | 0.000864 | down |
| ENSMUS( | Tnfsf12 | 73 | 57 | 59 | 92 | 101 | 83 | 0.644748 | 0.000144 | 0.000874 | down |
| ENSMUS( | Cxcl5 | 20 | 14 | 17 | 34 | 39 | 30 | 0.467061 | 0.000147 | 0.00089 | down |
| ENSMUS( | Kctd14 | 52 | 63 | 48 | 85 | 102 | 68 | 0.601213 | 0.000178 | 0.00106 | down |
| ENSMUS( | Cbln3 | 9 | 7 | 8 | 17 | 18 | 28 | 0.36245 | 0.000182 | 0.001077 | down |
| ENSMUS( | Serf1 | 63 | 65 | 61 | 94 | 82 | 98 | 0.652929 | 0.000192 | 0.001132 | down |
| ENSMUS( | Sncg | 76 | 52 | 64 | 84 | 105 | 106 | 0.616287 | 0.000192 | 0.001132 | down |
| ENSMUS( | Ccdc185 | 0 | 1 | 1 | 5 | 5 | 13 | 0.08596 | 0.000194 | 0.001144 | down |
| ENSMUS( | Tcf7l1 | 44 | 68 | 48 | 80 | 83 | 104 | 0.568305 | 0.000209 | 0.001224 | down |
| ENSMUS( | Ppp1r32 | 45 | 55 | 49 | 79 | 64 | 82 | 0.628049 | 0.000233 | 0.001352 | down |
| ENSMUS( | Mrps24 | 243 | 218 | 158 | 359 | 278 | 286 | 0.626672 | 0.00024 | 0.00139 | down |
| ENSMUS( | Gm10643 | 79 | 75 | 55 | 84 | 97 | 115 | 0.664561 | 0.000243 | 0.001408 | down |
| ENSMUS( | 2210011C2 | 1 | 2 | 1 | 12 | 2 | 15 | 0.130569 | 0.000274 | 0.001573 | down |
| ENSMUS( | Zfp575 | 51 | 57 | 49 | 109 | 72 | 68 | 0.592864 | 0.000278 | 0.001594 | down |
| ENSMUS( | Ino80b | 81 | 102 | 75 | 145 | 114 | 118 | 0.644044 | 0.000289 | 0.001651 | down |
| ENSMUS( | Cfap161 | 43 | 48 | 38 | 70 | 64 | 65 | 0.610715 | 0.000311 | 0.001767 | down |
| ENSMUS( | Olfr287 | 75 | 58 | 52 | 74 | 99 | 93 | 0.654261 | 0.000313 | 0.001776 | down |
| ENSMUS( | Cox6a2 | 27 | 20 | 32 | 56 | 42 | 55 | 0.492935 | 0.000313 | 0.001776 | down |
| ENSMUS( | Lrrc75a | 105 | 82 | 74 | 144 | 103 | 121 | 0.665153 | 0.000329 | 0.001857 | down |
| ENSMUS( | Myh15 | 48 | 50 | 38 | 75 | 65 | 67 | 0.617244 | 0.000338 | 0.001904 | down |
| ENSMUS( | Cnpy1 | 4 | 3 | 1 | 8 | 13 | 13 | 0.217174 | 0.000355 | 0.001991 | down |
| ENSMUS( | Gng8 | 24 | 24 | 13 | 31 | 41 | 42 | 0.500168 | 0.000368 | 0.002061 | down |
| ENSMUS( | Il13ra2 | 19 | 13 | 19 | 34 | 34 | 31 | 0.488743 | 0.000373 | 0.002085 | down |
| ENSMUS( | Sp8 | 25 | 22 | 25 | 41 | 39 | 46 | 0.542516 | 0.000435 | 0.002392 | down |
| ENSMUS( | Adra2c | 194 | 157 | 118 | 262 | 224 | 213 | 0.626291 | 0.000447 | 0.002453 | down |
| ENSMUS( | Alox8 | 19 | 38 | 29 | 46 | 64 | 80 | 0.433053 | 0.000505 | 0.002731 | down |
| ENSMUS( | 5730559C | 37 | 40 | 34 | 62 | 50 | 62 | 0.602511 | 0.000514 | 0.002774 | down |
| ENSMUS( | Gm10638 | 6 | 7 | 5 | 19 | 15 | 16 | 0.338202 | 0.000532 | 0.002863 | down |
| ENSMUS( | Wif1 | 13 | 11 | 16 | 34 | 23 | 25 | 0.464103 | 0.000582 | 0.003098 | down |
| ENSMUS( | Gm3667 | 5 | 14 | 8 | 28 | 21 | 28 | 0.332619 | 0.000582 | 0.003099 | down |
| ENSMUS( | Morn2 | 87 | 86 | 53 | 96 | 112 | 125 | 0.636097 | 0.000658 | 0.003456 | down |
| ENSMUS( | Ecel1 | 54 | 38 | 43 | 69 | 61 | 71 | 0.633808 | 0.00071 | 0.003699 | down |
| ENSMUS( | Gm14597 | 142 | 103 | 80 | 151 | 165 | 147 | 0.655332 | 0.000738 | 0.003833 | down |
| ENSMUS( | Riiad1 | 26 | 16 | 16 | 36 | 29 | 40 | 0.518464 | 0.000789 | 0.004068 | down |
| ENSMUS( | Tpsb2 | 4 | 8 | 5 | 20 | 14 | 13 | 0.340647 | 0.000824 | 0.004234 | down |
| ENSMUS( | Shox2 | 2 | 2 | 1 | 11 | 4 | 11 | 0.179254 | 0.00089 | 0.004551 | down |
| ENSMUS( | Slc30a2 | 31 | 36 | 24 | 49 | 38 | 64 | 0.568359 | 0.000934 | 0.004761 | down |
| ENSMUS( | Aldh3b2 | 20 | 24 | 17 | 34 | 33 | 41 | 0.532927 | 0.000949 | 0.004826 | down |
| ENSMUS( | Cd209b | 5 | 8 | 5 | 15 | 21 | 12 | 0.352474 | 0.000992 | 0.005023 | down |
| ENSMUS( | Ankrd2 | 1 | 1 | 1 | 10 | 12 | 1 | 0.12171 | 0.000994 | 0.00503 | down |
| ENSMUS( | Dmbt1 | 10 | 9 | 9 | 19 | 19 | 25 | 0.421385 | 0.001009 | 0.0051 | down |
| ENSMUS( | Fancc | 55 | 49 | 51 | 75 | 78 | 69 | 0.659599 | 0.001031 | 0.005196 | down |
| ENSMUS( | Rbp2 | 3 | 4 | 2 | 11 | 14 | 8 | 0.254015 | 0.001036 | 0.00522 | down |
| ENSMUS( | Wnt2 | 60 | 50 | 33 | 76 | 64 | 83 | 0.599212 | 0.001133 | 0.00565 | down |
| ENSMUS( | G0s2 | 124 | 88 | 75 | 144 | 132 | 128 | 0.664018 | 0.001293 | 0.006364 | down |
| ENSMUS( | Rarres1 | 28 | 32 | 27 | 51 | 34 | 60 | 0.568649 | 0.001303 | 0.006408 | down |
| ENSMUS( | Eva1b | 18 | 20 | 12 | 26 | 33 | 33 | 0.509859 | 0.00136 | 0.006659 | down |
| ENSMUS( | Hist2h3c2 | 9 | 13 | 17 | 22 | 29 | 35 | 0.438129 | 0.00141 | 0.006882 | down |
| ENSMUS( | Fam19a4 | 3 | 3 | 0 | 13 | 8 | 11 | 0.169601 | 0.001543 | 0.007468 | down |
| ENSMUS( | Nat8f2 | 26 | 37 | 23 | 43 | 50 | 48 | 0.574444 | 0.001589 | 0.007666 | down |
| ENSMUS( | Kcnmb1 | 29 | 20 | 24 | 39 | 46 | 35 | 0.573503 | 0.001768 | 0.008441 | down |
| ENSMUS( | Sp9 | 50 | 60 | 40 | 93 | 75 | 56 | 0.625917 | 0.001779 | 0.008486 | down |
| ENSMUS( | Gpr3 | 66 | 44 | 47 | 61 | 81 | 86 | 0.649706 | 0.002009 | 0.009474 | down |
| ENSMUS( | Gm6563 | 13 | 23 | 32 | 58 | 56 | 40 | 0.424966 | 0.002049 | 0.009648 | down |
| ENSMUS( | Fzd9 | 54 | 73 | 87 | 114 | 109 | 105 | 0.624151 | 0.002102 | 0.009874 | down |
| ENSMUS( | Asgr1 | 41 | 37 | 27 | 55 | 43 | 60 | 0.62362 | 0.002177 | 0.010198 | down |
| ENSMUS( | Pycr1 | 19 | 19 | 17 | 34 | 31 | 31 | 0.540288 | 0.002268 | 0.010564 | down |
| ENSMUS( | Tmem219 | 73 | 55 | 45 | 73 | 95 | 78 | 0.658385 | 0.002445 | 0.011298 | down |
| ENSMUS( | Klk6 | 12 | 12 | 7 | 34 | 22 | 13 | 0.415349 | 0.002469 | 0.011389 | down |
| ENSMUS( | Sfn | 9 | 13 | 8 | 28 | 12 | 26 | 0.427932 | 0.00262 | 0.012025 | down |
| ENSMUS( | Ninj2 | 8 | 16 | 16 | 24 | 27 | 34 | 0.452919 | 0.002621 | 0.012027 | down |
| ENSMUS( | Gng13 | 14 | 16 | 8 | 30 | 24 | 19 | 0.483092 | 0.002655 | 0.012164 | down |
| ENSMUS( | Cort | 30 | 23 | 39 | 54 | 55 | 53 | 0.543251 | 0.002747 | 0.012526 | down |
| ENSMUS( | Mcpt4 | 5 | 11 | 2 | 20 | 23 | 18 | 0.27255 | 0.002872 | 0.013015 | down |
| ENSMUS( | Igfbpl1 | 42 | 32 | 35 | 45 | 49 | 68 | 0.638239 | 0.003134 | 0.014068 | down |
| ENSMUS( | Scarf2 | 45 | 23 | 24 | 52 | 56 | 53 | 0.533677 | 0.003157 | 0.014167 | down |
| ENSMUS( | Fbxl8 | 15 | 7 | 11 | 20 | 27 | 19 | 0.470801 | 0.003276 | 0.014655 | down |
| ENSMUS( | Fam150b | 10 | 13 | 9 | 24 | 16 | 24 | 0.471433 | 0.0033 | 0.01475 | down |
| ENSMUS( | Aspdh | 18 | 12 | 14 | 24 | 35 | 22 | 0.511166 | 0.003351 | 0.014949 | down |
| ENSMUS( | Adam5 | 0 | 5 | 3 | 18 | 19 | 8 | 0.16975 | 0.00365 | 0.016145 | down |
| ENSMUS( | 4933407L2 | 10 | 16 | 19 | 28 | 30 | 32 | 0.4808 | 0.003712 | 0.016371 | down |
| ENSMUS( | Dbp | 298 | 337 | 362 | 666 | 467 | 379 | 0.623524 | 0.003751 | 0.016525 | down |
| ENSMUS( | Gsx1 | 2 | 4 | 1 | 9 | 8 | 9 | 0.249959 | 0.003841 | 0.016889 | down |
| ENSMUS( | Gm9008 | 18 | 10 | 17 | 27 | 28 | 28 | 0.514922 | 0.003989 | 0.017458 | down |
| ENSMUS( | Ret | 37 | 42 | 32 | 50 | 57 | 54 | 0.649918 | 0.004041 | 0.017662 | down |
| ENSMUS( | Cmtm7 | 49 | 34 | 22 | 58 | 64 | 52 | 0.559865 | 0.004158 | 0.018085 | down |
| ENSMUS( | Aldh1a3 | 3 | 1 | 0 | 10 | 11 | 7 | 0.127635 | 0.004309 | 0.01864 | down |

| | | | | | | | | | | | |
|---|---|---|---|---|---|---|---|---|---|---|---|
| ENSMUS( | 4930407I1 | 5 | 1 | 0 | 9 | 19 | 14 | 0.128103 | 0.004476 | 0.019316 | down |
| ENSMUS( | Gm3512 | 15 | 9 | 18 | 42 | 32 | 20 | 0.423986 | 0.005218 | 0.022132 | down |
| ENSMUS( | Fat2 | 2 | 2 | 4 | 7 | 6 | 14 | 0.289484 | 0.005332 | 0.02258 | down |
| ENSMUS( | Plcz1 | 19 | 15 | 19 | 31 | 32 | 26 | 0.563796 | 0.00549 | 0.023177 | down |
| ENSMUS( | Nme9 | 8 | 7 | 7 | 20 | 16 | 12 | 0.430432 | 0.005545 | 0.023374 | down |
| ENSMUS( | Irx5 | 2 | 4 | 4 | 6 | 11 | 13 | 0.321884 | 0.005615 | 0.023656 | down |
| ENSMUS( | Tnnt3 | 5 | 2 | 3 | 11 | 8 | 11 | 0.312779 | 0.005617 | 0.023662 | down |
| ENSMUS( | Ror2 | 9 | 9 | 4 | 16 | 17 | 15 | 0.42502 | 0.005636 | 0.023727 | down |
| ENSMUS( | Dnd1 | 16 | 19 | 15 | 25 | 33 | 27 | 0.555299 | 0.005783 | 0.024287 | down |
| ENSMUS( | Tbx18 | 68 | 46 | 63 | 95 | 73 | 84 | 0.664926 | 0.005835 | 0.024477 | down |
| ENSMUS( | Malsu1 | 98 | 82 | 57 | 160 | 107 | 99 | 0.601935 | 0.005914 | 0.024774 | down |
| ENSMUS( | Unc5cl | 4 | 5 | 3 | 10 | 10 | 13 | 0.342224 | 0.005974 | 0.02499 | down |
| ENSMUS( | Ripply2 | 56 | 36 | 43 | 69 | 60 | 64 | 0.6589 | 0.006036 | 0.025235 | down |
| ENSMUS( | Nxph4 | 17 | 12 | 11 | 23 | 24 | 25 | 0.521349 | 0.006045 | 0.025258 | down |
| ENSMUS( | Fxyd2 | 29 | 34 | 29 | 46 | 53 | 37 | 0.637913 | 0.006346 | 0.026355 | down |
| ENSMUS( | Irx1 | 4 | 3 | 2 | 10 | 10 | 8 | 0.298562 | 0.006375 | 0.02646 | down |
| ENSMUS( | Sapcd2 | 23 | 29 | 27 | 38 | 42 | 40 | 0.625162 | 0.006409 | 0.02659 | down |
| ENSMUS( | Rpl7a-ps5 | 1 | 2 | 0 | 5 | 6 | 6 | 0.161094 | 0.006862 | 0.028212 | down |
| ENSMUS( | Kif7 | 27 | 32 | 26 | 34 | 56 | 41 | 0.61334 | 0.006889 | 0.028309 | down |
| ENSMUS( | Nrtn | 2 | 4 | 1 | 19 | 2 | 6 | 0.237509 | 0.006954 | 0.028548 | down |
| ENSMUS( | Atp23 | 73 | 105 | 73 | 123 | 131 | 101 | 0.66572 | 0.007319 | 0.029871 | down |
| ENSMUS( | Fam227b | 41 | 43 | 27 | 55 | 43 | 59 | 0.662764 | 0.007323 | 0.029878 | down |
| ENSMUS( | Nhlh2 | 41 | 24 | 23 | 47 | 44 | 50 | 0.58391 | 0.007426 | 0.030258 | down |
| ENSMUS( | Cst6 | 56 | 41 | 67 | 78 | 73 | 94 | 0.640661 | 0.007589 | 0.030831 | down |
| ENSMUS( | Pf4 | 25 | 26 | 24 | 45 | 28 | 43 | 0.611541 | 0.008261 | 0.033257 | down |
| ENSMUS( | Sp7 | 25 | 18 | 24 | 27 | 34 | 43 | 0.614024 | 0.008404 | 0.033747 | down |
| ENSMUS( | Cartpt | 26 | 24 | 19 | 53 | 31 | 29 | 0.569887 | 0.008409 | 0.03376 | down |
| ENSMUS( | Kcnk12 | 26 | 20 | 26 | 52 | 38 | 26 | 0.584726 | 0.008455 | 0.033913 | down |
| ENSMUS( | Car15 | 7 | 11 | 4 | 9 | 16 | 30 | 0.377397 | 0.008545 | 0.034229 | down |
| ENSMUS( | Smtnl2 | 8 | 13 | 12 | 13 | 27 | 23 | 0.501447 | 0.008575 | 0.034317 | down |
| ENSMUS( | Gm5415 | 31 | 32 | 25 | 54 | 38 | 39 | 0.630038 | 0.009385 | 0.037162 | down |
| ENSMUS( | Npw | 1 | 2 | 2 | 12 | 4 | 5 | 0.226085 | 0.009409 | 0.037241 | down |
| ENSMUS( | Sox3 | 21 | 19 | 12 | 32 | 30 | 23 | 0.569024 | 0.009463 | 0.037414 | down |
| ENSMUS( | Cebpzos | 28 | 40 | 39 | 50 | 57 | 52 | 0.641214 | 0.009485 | 0.037484 | down |
| ENSMUS( | Gpr139 | 19 | 21 | 6 | 30 | 30 | 32 | 0.461626 | 0.009671 | 0.038113 | down |
| ENSMUS( | CAAA011 | 2 | 8 | 3 | 20 | 10 | 13 | 0.283581 | 0.009991 | 0.039215 | down |
| ENSMUS( | Irx6 | 1 | 2 | 1 | 5 | 6 | 7 | 0.209842 | 0.010381 | 0.040591 | down |
| ENSMUS( | Teddm2 | 14 | 11 | 18 | 23 | 21 | 31 | 0.55051 | 0.011033 | 0.042797 | down |
| ENSMUS( | Ccl21a | 1 | 5 | 3 | 6 | 14 | 8 | 0.306989 | 0.011117 | 0.043052 | down |
| ENSMUS( | Sntn | 27 | 23 | 14 | 34 | 24 | 44 | 0.587056 | 0.011473 | 0.044294 | down |
| ENSMUS( | 1700012B( | 8 | 16 | 16 | 30 | 20 | 26 | 0.504003 | 0.011481 | 0.044313 | down |
| ENSMUS( | Xlr3b | 37 | 37 | 28 | 44 | 35 | 74 | 0.631168 | 0.011566 | 0.044593 | down |
| ENSMUS( | Insc | 63 | 32 | 49 | 75 | 68 | 71 | 0.63501 | 0.011627 | 0.044803 | down |
| ENSMUS( | 3110009E1 | 31 | 21 | 25 | 32 | 46 | 36 | 0.637649 | 0.01163 | 0.044803 | down |
| ENSMUS( | Gm7347 | 0 | 0 | 1 | 5 | 2 | 5 | 0.085939 | 0.011895 | 0.045723 | down |
| ENSMUS( | Birc7 | 6 | 8 | 3 | 15 | 13 | 10 | 0.413994 | 0.012343 | 0.047233 | down |
| ENSMUS( | Npbwr1 | 27 | 21 | 15 | 38 | 28 | 31 | 0.605138 | 0.012352 | 0.047255 | down |
| ENSMUS( | Klrg1 | 27 | 24 | 25 | 48 | 28 | 40 | 0.618781 | 0.012421 | 0.047494 | down |
| ENSMUS( | Nr2c2ap | 8 | 7 | 12 | 16 | 14 | 22 | 0.500719 | 0.012818 | 0.048797 | down |
| ENSMUS( | Cd79b | 5 | 4 | 4 | 10 | 7 | 15 | 0.385297 | 0.01316 | 0.049906 | down |

**Dataset S3. List of DE genes between RS and SI (RS/SI) female offspriing**

| Gene | GeneName | RS-1_cour | RS-2_cour | RS-3_cour | SI-1_count | SI-2_count | SI-3_count | FoldChang | p | q | Up/Down |
|---|---|---|---|---|---|---|---|---|---|---|---|
| ENSMUS( | Lcn2 | 83 | 96 | 78 | 1047 | 916 | 826 | 0.095902 | 6.1E-290 | 1.7E-285 | down |
| ENSMUS( | Apod | 3547 | 3449 | 3304 | 8359 | 7578 | 6840 | 0.471339 | 6.2E-134 | 3.6E-130 | down |
| ENSMUS( | H2-D1 | 504 | 489 | 474 | 1244 | 1184 | 1096 | 0.433301 | 1.75E-90 | 7.14E-87 | down |
| ENSMUS( | Cd74 | 110 | 138 | 121 | 512 | 499 | 463 | 0.260514 | 2.97E-77 | 9.44E-74 | down |
| ENSMUS( | Retsat | 339 | 374 | 323 | 935 | 860 | 863 | 0.404463 | 1.15E-72 | 3.28E-69 | down |
| ENSMUS( | Plin4 | 84 | 67 | 53 | 410 | 379 | 355 | 0.184297 | 1.11E-65 | 2.65E-62 | down |
| ENSMUS( | Ptgds | 15333 | 15353 | 14647 | 32634 | 29077 | 25345 | 0.543591 | 2.62E-59 | 5.36E-56 | down |
| ENSMUS( | Gm8797 | 125 | 132 | 133 | 471 | 438 | 353 | 0.323665 | 3.63E-59 | 6.92E-56 | down |
| ENSMUS( | Fkbp5 | 684 | 723 | 602 | 1239 | 1297 | 1151 | 0.565132 | 1.08E-45 | 1.55E-42 | down |
| ENSMUS( | A2m | 274 | 254 | 221 | 579 | 560 | 501 | 0.474099 | 2.03E-43 | 2.42E-40 | down |
| ENSMUS( | Slc2a1 | 921 | 928 | 827 | 1637 | 1518 | 1394 | 0.611702 | 2.18E-42 | 2.4E-39 | down |
| ENSMUS( | S100a9 | 29 | 28 | 29 | 156 | 136 | 147 | 0.203724 | 2.55E-39 | 2.43E-36 | down |
| ENSMUS( | Hbb-bs | 1269 | 1436 | 995 | 2684 | 2411 | 2176 | 0.527131 | 1.59E-36 | 1.42E-33 | down |
| ENSMUS( | Lyz2 | 221 | 254 | 199 | 546 | 512 | 418 | 0.475378 | 1.83E-36 | 1.59E-33 | down |
| ENSMUS( | Sult1a1 | 258 | 240 | 192 | 580 | 554 | 446 | 0.453812 | 2.97E-35 | 2.43E-32 | down |
| ENSMUS( | Agt | 596 | 603 | 518 | 977 | 1006 | 921 | 0.613067 | 8.98E-32 | 5.84E-29 | down |
| ENSMUS( | Flt1 | 211 | 262 | 231 | 570 | 500 | 487 | 0.470906 | 3.26E-30 | 1.99E-27 | down |
| ENSMUS( | Trpm3 | 1007 | 997 | 830 | 1508 | 1529 | 1416 | 0.659211 | 2.48E-29 | 1.48E-26 | down |
| ENSMUS( | Etnppl | 80 | 74 | 75 | 246 | 210 | 237 | 0.342988 | 3.41E-29 | 1.99E-26 | down |
| ENSMUS( | Fmod | 177 | 173 | 142 | 369 | 383 | 304 | 0.484144 | 6.54E-29 | 3.74E-26 | down |
| ENSMUS( | Acer2 | 117 | 115 | 100 | 290 | 250 | 260 | 0.430292 | 2.09E-28 | 1.15E-25 | down |
| ENSMUS( | C4b | 530 | 523 | 389 | 928 | 927 | 819 | 0.557915 | 1.25E-27 | 6.74E-25 | down |
| ENSMUS( | Cmip | 865 | 864 | 705 | 1297 | 1351 | 1153 | 0.664161 | 1.77E-27 | 9.37E-25 | down |
| ENSMUS( | Fn1 | 362 | 374 | 307 | 650 | 630 | 568 | 0.585622 | 2.51E-27 | 1.3E-24 | down |
| ENSMUS( | F5 | 801 | 791 | 712 | 1239 | 1258 | 1116 | 0.66257 | 4.13E-27 | 2.04E-24 | down |
| ENSMUS( | Cp | 487 | 440 | 424 | 780 | 796 | 750 | 0.602821 | 4.08E-27 | 2.04E-24 | down |
| ENSMUS( | Slc6a20a | 239 | 271 | 246 | 510 | 451 | 448 | 0.558276 | 7.08E-26 | 3.22E-23 | down |
| ENSMUS( | H2-Q7 | 82 | 85 | 71 | 207 | 222 | 175 | 0.409736 | 4.74E-24 | 1.97E-21 | down |
| ENSMUS( | Ctsh | 91 | 78 | 78 | 197 | 226 | 199 | 0.412124 | 2.48E-23 | 9.98E-21 | down |
| ENSMUS( | Mpeg1 | 451 | 400 | 364 | 692 | 710 | 610 | 0.627213 | 2.43E-22 | 9.14E-20 | down |
| ENSMUS( | Pcdhgc5 | 309 | 378 | 265 | 634 | 639 | 555 | 0.539384 | 2.49E-22 | 9.26E-20 | down |
| ENSMUS( | Chil3 | 8 | 7 | 11 | 69 | 83 | 50 | 0.136493 | 3.8E-22 | 1.38E-19 | down |
| ENSMUS( | Hmgcs2 | 224 | 184 | 230 | 489 | 478 | 409 | 0.485618 | 7.78E-22 | 2.78E-19 | down |
| ENSMUS( | Prg4 | 63 | 59 | 46 | 198 | 159 | 137 | 0.353799 | 2.09E-21 | 7.1E-19 | down |
| ENSMUS( | Spp1 | 172 | 191 | 161 | 332 | 364 | 330 | 0.529376 | 5.86E-21 | 1.97E-18 | down |
| ENSMUS( | Zbtb16 | 260 | 283 | 239 | 461 | 466 | 452 | 0.587496 | 2.12E-20 | 6.65E-18 | down |
| ENSMUS( | Hif3a | 103 | 121 | 101 | 260 | 230 | 252 | 0.453663 | 2.29E-20 | 7.11E-18 | down |
| ENSMUS( | Saa3 | 0 | 2 | 1 | 29 | 36 | 37 | 0.030346 | 3.57E-20 | 1.1E-17 | down |
| ENSMUS( | Ly6c1 | 618 | 657 | 630 | 1158 | 1089 | 900 | 0.632664 | 6.53E-20 | 1.91E-17 | down |
| ENSMUS( | S100a8 | 18 | 23 | 14 | 79 | 86 | 66 | 0.246332 | 1.3E-18 | 3.42E-16 | down |
| ENSMUS( | Npas4 | 146 | 135 | 141 | 292 | 290 | 234 | 0.540568 | 4.22E-18 | 1.02E-15 | down |
| ENSMUS( | Ahnak | 470 | 422 | 363 | 717 | 668 | 638 | 0.642886 | 5.97E-18 | 1.41E-15 | down |
| ENSMUS( | Kirrel2 | 192 | 213 | 176 | 357 | 373 | 308 | 0.581876 | 6.77E-18 | 1.59E-15 | down |
| ENSMUS( | Jchain | 336 | 331 | 247 | 547 | 552 | 490 | 0.594937 | 2.16E-17 | 4.83E-15 | down |
| ENSMUS( | Baiap2l1 | 65 | 79 | 50 | 170 | 194 | 188 | 0.361149 | 5E-17 | 1.08E-14 | down |
| ENSMUS( | Ctla2a | 137 | 128 | 144 | 287 | 287 | 256 | 0.514722 | 5.96E-17 | 1.26E-14 | down |
| ENSMUS( | H2-Eb1 | 48 | 64 | 68 | 187 | 175 | 152 | 0.367529 | 3.07E-16 | 6.14E-14 | down |
| ENSMUS( | Tenm3 | 470 | 422 | 368 | 659 | 682 | 622 | 0.664879 | 3.34E-16 | 6.58E-14 | down |
| ENSMUS( | Lin54 | 321 | 318 | 272 | 518 | 496 | 437 | 0.652379 | 8.25E-16 | 1.56E-13 | down |
| ENSMUS( | Gse1 | 238 | 218 | 185 | 365 | 371 | 350 | 0.610768 | 1E-15 | 1.89E-13 | down |
| ENSMUS( | Arl4d | 165 | 152 | 138 | 280 | 300 | 245 | 0.57337 | 2.16E-15 | 3.91E-13 | down |
| ENSMUS( | Adamts1 | 359 | 351 | 281 | 522 | 571 | 493 | 0.646813 | 2.3E-15 | 4.14E-13 | down |
| ENSMUS( | Dlk1 | 69 | 81 | 70 | 185 | 155 | 143 | 0.474992 | 4.08E-15 | 7.03E-13 | down |
| ENSMUS( | H2-T23 | 98 | 119 | 107 | 229 | 207 | 196 | 0.534161 | 8.65E-15 | 1.44E-12 | down |
| ENSMUS( | Fmo2 | 33 | 22 | 22 | 99 | 72 | 91 | 0.303683 | 1.35E-14 | 2.18E-12 | down |
| ENSMUS( | Abcb1a | 228 | 231 | 181 | 368 | 347 | 350 | 0.621097 | 1.42E-14 | 2.28E-12 | down |
| ENSMUS( | Pnpla2 | 319 | 290 | 278 | 513 | 458 | 419 | 0.664546 | 2.3E-14 | 3.6E-12 | down |
| ENSMUS( | Pdk4 | 324 | 386 | 271 | 618 | 560 | 480 | 0.614156 | 2.69E-14 | 4.16E-12 | down |
| ENSMUS( | C1qc | 527 | 494 | 500 | 977 | 861 | 675 | 0.634173 | 9.5E-14 | 1.39E-11 | down |
| ENSMUS( | Ly6a | 309 | 328 | 336 | 704 | 574 | 543 | 0.558997 | 1.2E-13 | 1.7E-11 | down |
| ENSMUS( | Ms4a6c | 11 | 12 | 8 | 50 | 53 | 43 | 0.219593 | 1.56E-13 | 2.17E-11 | down |
| ENSMUS( | H2-Q4 | 58 | 52 | 64 | 156 | 142 | 116 | 0.441689 | 1.63E-13 | 2.26E-11 | down |
| ENSMUS( | Slc22a8 | 231 | 229 | 211 | 370 | 375 | 340 | 0.642607 | 2.29E-13 | 3.08E-11 | down |
| ENSMUS( | Ccr2 | 18 | 23 | 17 | 75 | 66 | 57 | 0.304768 | 3.17E-13 | 4.2E-11 | down |
| ENSMUS( | Xdh | 41 | 73 | 55 | 158 | 171 | 140 | 0.375272 | 3.31E-13 | 4.36E-11 | down |
| ENSMUS( | Actbl2 | 7 | 13 | 10 | 45 | 48 | 48 | 0.220624 | 4.18E-13 | 5.43E-11 | down |
| ENSMUS( | Wfikkn2 | 43 | 53 | 52 | 113 | 142 | 110 | 0.423221 | 4.35E-13 | 5.63E-11 | down |
| ENSMUS( | Omd | 50 | 33 | 29 | 128 | 115 | 99 | 0.339459 | 5.16E-13 | 6.59E-11 | down |
| ENSMUS( | Cyp4f15 | 168 | 179 | 143 | 289 | 304 | 243 | 0.608979 | 5.57E-13 | 7.05E-11 | down |
| ENSMUS( | Pla2g3 | 45 | 36 | 45 | 101 | 103 | 110 | 0.417185 | 6.82E-13 | 8.45E-11 | down |
| ENSMUS( | Fcgr4 | 6 | 3 | 6 | 39 | 29 | 49 | 0.133059 | 1.1E-12 | 1.33E-10 | down |
| ENSMUS( | Vwf | 163 | 154 | 157 | 281 | 287 | 240 | 0.612061 | 1.43E-12 | 1.7E-10 | down |
| ENSMUS( | Fcgr3 | 249 | 230 | 250 | 437 | 407 | 351 | 0.637996 | 2.86E-12 | 3.31E-10 | down |
| ENSMUS( | Ms4a6b | 52 | 35 | 48 | 123 | 129 | 106 | 0.39469 | 5.48E-12 | 6.11E-10 | down |
| ENSMUS( | Ddc | 86 | 100 | 76 | 187 | 175 | 145 | 0.537444 | 5.66E-12 | 6.25E-10 | down |
| ENSMUS( | Cdh1 | 19 | 18 | 13 | 54 | 64 | 54 | 0.299828 | 8.75E-12 | 9.45E-10 | down |
| ENSMUS( | Ms4a6d | 39 | 39 | 36 | 92 | 99 | 90 | 0.421103 | 1.24E-11 | 1.3E-09 | down |
| ENSMUS( | Hipk2 | 166 | 158 | 142 | 258 | 299 | 314 | 0.551602 | 1.5E-11 | 1.56E-09 | down |
| ENSMUS( | Cybb | 18 | 27 | 21 | 74 | 62 | 62 | 0.346756 | 2.19E-11 | 2.26E-09 | down |
| ENSMUS( | Xaf1 | 119 | 137 | 155 | 298 | 273 | 227 | 0.541414 | 2.36E-11 | 2.39E-09 | down |
| ENSMUS( | Sult1c2 | 23 | 21 | 28 | 78 | 69 | 61 | 0.364125 | 2.52E-11 | 2.52E-09 | down |

| | | | | | | | | | | | |
|---|---|---|---|---|---|---|---|---|---|---|---|
| ENSMUS( | Itih2 | 107 | 96 | 101 | 186 | 223 | 173 | 0.544551 | 3.74E-11 | 3.59E-09 | down |
| ENSMUS( | Apold1 | 54 | 65 | 71 | 144 | 147 | 143 | 0.457072 | 3.89E-11 | 3.72E-09 | down |
| ENSMUS( | Slc6a13 | 209 | 166 | 177 | 318 | 327 | 295 | 0.610689 | 3.95E-11 | 3.77E-09 | down |
| ENSMUS( | Fbln1 | 162 | 163 | 173 | 290 | 315 | 245 | 0.612882 | 4.7E-11 | 4.44E-09 | down |
| ENSMUS( | Hbb-bt | 281 | 277 | 204 | 457 | 436 | 351 | 0.635701 | 5.05E-11 | 4.74E-09 | down |
| ENSMUS( | Klf4 | 35 | 36 | 28 | 90 | 90 | 70 | 0.411698 | 6.03E-11 | 5.55E-09 | down |
| ENSMUS( | Sun2 | 222 | 267 | 228 | 386 | 425 | 342 | 0.647383 | 6.27E-11 | 5.75E-09 | down |
| ENSMUS( | Samd3 | 5 | 5 | 2 | 36 | 29 | 20 | 0.145771 | 9.43E-11 | 8.49E-09 | down |
| ENSMUS( | Rtp1 | 19 | 27 | 16 | 80 | 63 | 49 | 0.336165 | 1.18E-10 | 1.05E-08 | down |
| ENSMUS( | Ptprn2 | 803 | 1017 | 1069 | 1889 | 1658 | 1448 | 0.60716 | 1.43E-10 | 1.25E-08 | down |
| ENSMUS( | Acvrl1 | 82 | 65 | 59 | 148 | 127 | 134 | 0.52126 | 1.46E-10 | 1.26E-08 | down |
| ENSMUS( | Maff | 18 | 26 | 17 | 68 | 65 | 49 | 0.348729 | 1.64E-10 | 1.41E-08 | down |
| ENSMUS( | Cd14 | 78 | 64 | 63 | 140 | 137 | 129 | 0.52413 | 1.95E-10 | 1.65E-08 | down |
| ENSMUS( | Adgrf5 | 253 | 236 | 207 | 356 | 376 | 361 | 0.658796 | 1.97E-10 | 1.67E-08 | down |
| ENSMUS( | Ier3 | 58 | 55 | 49 | 118 | 108 | 115 | 0.491736 | 2.89E-10 | 2.4E-08 | down |
| ENSMUS( | Kcna1 | 301 | 313 | 259 | 419 | 509 | 480 | 0.639991 | 3.43E-10 | 2.8E-08 | down |
| ENSMUS( | H2-Aa | 30 | 29 | 34 | 79 | 79 | 75 | 0.41686 | 3.47E-10 | 2.81E-08 | down |
| ENSMUS( | Cdkn1a | 208 | 211 | 179 | 378 | 334 | 270 | 0.635243 | 3.77E-10 | 3.05E-08 | down |
| ENSMUS( | Erg | 17 | 24 | 17 | 64 | 53 | 56 | 0.347282 | 4.61E-10 | 3.67E-08 | down |
| ENSMUS( | H2-Q6 | 61 | 37 | 55 | 138 | 152 | 107 | 0.40485 | 1.4E-09 | 1.05E-07 | down |
| ENSMUS( | Adcy9 | 384 | 354 | 255 | 606 | 520 | 531 | 0.618486 | 1.95E-09 | 1.42E-07 | down |
| ENSMUS( | Tfcp2l1 | 68 | 77 | 58 | 145 | 127 | 118 | 0.540127 | 2.11E-09 | 1.53E-07 | down |
| ENSMUS( | Slc22a6 | 95 | 97 | 81 | 216 | 154 | 161 | 0.534838 | 2.2E-09 | 1.59E-07 | down |
| ENSMUS( | Msr1 | 10 | 12 | 7 | 48 | 37 | 29 | 0.264402 | 2.4E-09 | 1.72E-07 | down |
| ENSMUS( | Fosb | 145 | 123 | 119 | 210 | 255 | 211 | 0.593952 | 2.41E-09 | 1.72E-07 | down |
| ENSMUS( | Icam1 | 38 | 46 | 39 | 111 | 108 | 71 | 0.444769 | 2.78E-09 | 1.97E-07 | down |
| ENSMUS( | Ctxn3 | 34 | 54 | 40 | 117 | 90 | 101 | 0.431378 | 3.24E-09 | 2.25E-07 | down |
| ENSMUS( | Plvap | 140 | 150 | 116 | 221 | 237 | 203 | 0.636294 | 3.69E-09 | 2.5E-07 | down |
| ENSMUS( | Clcnka | 17 | 19 | 13 | 54 | 43 | 52 | 0.338564 | 4.59E-09 | 3.06E-07 | down |
| ENSMUS( | Irgm2 | 81 | 79 | 49 | 192 | 169 | 127 | 0.444176 | 4.72E-09 | 3.13E-07 | down |
| ENSMUS( | Eomes | 13 | 10 | 6 | 34 | 36 | 44 | 0.258566 | 5.26E-09 | 3.47E-07 | down |
| ENSMUS( | Lnpep | 194 | 207 | 153 | 285 | 309 | 301 | 0.638101 | 5.29E-09 | 3.48E-07 | down |
| ENSMUS( | Tmem252 | 71 | 56 | 41 | 129 | 132 | 110 | 0.467683 | 5.96E-09 | 3.88E-07 | down |
| ENSMUS( | Plac8 | 12 | 15 | 12 | 48 | 42 | 40 | 0.311888 | 6.09E-09 | 3.94E-07 | down |
| ENSMUS( | Thbd | 169 | 132 | 122 | 260 | 245 | 216 | 0.609206 | 6.94E-09 | 4.44E-07 | down |
| ENSMUS( | Spn | 9 | 5 | 5 | 34 | 23 | 33 | 0.217132 | 7.63E-09 | 4.83E-07 | down |
| ENSMUS( | Ccl5 | 12 | 10 | 10 | 45 | 36 | 35 | 0.287446 | 7.94E-09 | 5.01E-07 | down |
| ENSMUS( | Thsd4 | 229 | 195 | 208 | 340 | 352 | 363 | 0.621145 | 8.24E-09 | 5.17E-07 | down |
| ENSMUS( | Alas2 | 36 | 66 | 41 | 133 | 110 | 110 | 0.419852 | 8.46E-09 | 5.3E-07 | down |
| ENSMUS( | Pglyrp1 | 48 | 34 | 54 | 114 | 110 | 121 | 0.411912 | 8.98E-09 | 5.59E-07 | down |
| ENSMUS( | Cyyr1 | 52 | 73 | 43 | 118 | 129 | 125 | 0.46428 | 9.87E-09 | 6.02E-07 | down |
| ENSMUS( | Dsc3 | 42 | 23 | 41 | 103 | 92 | 94 | 0.384292 | 1.57E-08 | 9.17E-07 | down |
| ENSMUS( | C3ar1 | 20 | 16 | 22 | 63 | 48 | 49 | 0.380619 | 1.61E-08 | 9.36E-07 | down |
| ENSMUS( | C3 | 40 | 31 | 21 | 87 | 88 | 63 | 0.40014 | 1.7E-08 | 9.84E-07 | down |
| ENSMUS( | Cxcl1 | 8 | 8 | 6 | 38 | 33 | 23 | 0.244355 | 1.7E-08 | 9.84E-07 | down |
| ENSMUS( | Atp10a | 80 | 56 | 44 | 125 | 147 | 123 | 0.469464 | 2.89E-08 | 1.6E-06 | down |
| ENSMUS( | Anxa1 | 59 | 65 | 70 | 138 | 127 | 98 | 0.561776 | 3.21E-08 | 1.76E-06 | down |
| ENSMUS( | Osmr | 91 | 78 | 93 | 161 | 163 | 148 | 0.579637 | 3.29E-08 | 1.8E-06 | down |
| ENSMUS( | Htr1b | 142 | 132 | 108 | 213 | 217 | 183 | 0.646323 | 3.36E-08 | 1.83E-06 | down |
| ENSMUS( | Egr2 | 77 | 43 | 50 | 146 | 127 | 111 | 0.461009 | 3.57E-08 | 1.93E-06 | down |
| ENSMUS( | Pgm5 | 59 | 70 | 49 | 112 | 122 | 103 | 0.546481 | 4.77E-08 | 2.52E-06 | down |
| ENSMUS( | Ctla2b | 14 | 16 | 13 | 43 | 51 | 36 | 0.344431 | 5.1E-08 | 2.64E-06 | down |
| ENSMUS( | Sla | 145 | 105 | 104 | 226 | 207 | 187 | 0.593253 | 6.35E-08 | 3.23E-06 | down |
| ENSMUS( | Cxcl9 | 3 | 6 | 8 | 33 | 27 | 30 | 0.199546 | 7.06E-08 | 3.58E-06 | down |
| ENSMUS( | Galnt15 | 25 | 20 | 18 | 64 | 68 | 40 | 0.383217 | 9.4E-08 | 4.58E-06 | down |
| ENSMUS( | Itgb2 | 104 | 85 | 98 | 184 | 159 | 148 | 0.61093 | 9.62E-08 | 4.67E-06 | down |
| ENSMUS( | Ifi204 | 17 | 19 | 21 | 53 | 45 | 53 | 0.393201 | 1.1E-07 | 5.27E-06 | down |
| ENSMUS( | Slc6a12 | 53 | 67 | 50 | 129 | 115 | 85 | 0.539688 | 1.87E-07 | 8.48E-06 | down |
| ENSMUS( | Tns2 | 218 | 175 | 146 | 306 | 285 | 265 | 0.651664 | 2.38E-07 | 1.06E-05 | down |
| ENSMUS( | Ccr1 | 36 | 38 | 36 | 78 | 79 | 73 | 0.49743 | 2.42E-07 | 1.07E-05 | down |
| ENSMUS( | Filip1 | 141 | 143 | 109 | 211 | 210 | 190 | 0.665651 | 2.63E-07 | 1.15E-05 | down |
| ENSMUS( | Gzmb | 3 | 3 | 1 | 17 | 22 | 13 | 0.137923 | 3.43E-07 | 1.46E-05 | down |
| ENSMUS( | Oasl2 | 70 | 84 | 79 | 149 | 121 | 130 | 0.606775 | 3.71E-07 | 1.56E-05 | down |
| ENSMUS( | Pla2g2f | 15 | 11 | 5 | 39 | 38 | 47 | 0.253057 | 3.99E-07 | 1.66E-05 | down |
| ENSMUS( | Egr3 | 260 | 257 | 189 | 359 | 399 | 382 | 0.637832 | 4.15E-07 | 1.72E-05 | down |
| ENSMUS( | Mmp8 | 3 | 6 | 3 | 17 | 30 | 19 | 0.187348 | 4.29E-07 | 1.77E-05 | down |
| ENSMUS( | Gpr88 | 86 | 91 | 78 | 142 | 158 | 128 | 0.619206 | 4.46E-07 | 1.82E-05 | down |
| ENSMUS( | Zfp36 | 73 | 72 | 66 | 128 | 147 | 104 | 0.580834 | 5.14E-07 | 2.07E-05 | down |
| ENSMUS( | Clec4a3 | 23 | 23 | 13 | 54 | 51 | 42 | 0.413999 | 6.95E-07 | 2.72E-05 | down |
| ENSMUS( | Scgb3a1 | 3 | 4 | 2 | 15 | 25 | 15 | 0.168488 | 7.68E-07 | 2.96E-05 | down |
| ENSMUS( | Cpxm2 | 28 | 36 | 39 | 80 | 82 | 61 | 0.485932 | 7.71E-07 | 2.97E-05 | down |
| ENSMUS( | Trim30a | 78 | 89 | 69 | 142 | 127 | 130 | 0.612342 | 7.79E-07 | 3E-05 | down |
| ENSMUS( | Srgn | 170 | 156 | 175 | 334 | 325 | 218 | 0.601229 | 9.14E-07 | 3.45E-05 | down |
| ENSMUS( | Steap4 | 36 | 27 | 16 | 81 | 67 | 58 | 0.395379 | 9.25E-07 | 3.47E-05 | down |
| ENSMUS( | Zkscan16 | 281 | 276 | 202 | 394 | 387 | 399 | 0.662539 | 1.03E-06 | 3.81E-05 | down |
| ENSMUS( | Fam205c | 9 | 9 | 4 | 31 | 21 | 29 | 0.276762 | 1.32E-06 | 4.68E-05 | down |
| ENSMUS( | C430049E | 8 | 6 | 3 | 21 | 32 | 19 | 0.242328 | 1.32E-06 | 4.68E-05 | down |
| ENSMUS( | Efna1 | 83 | 91 | 100 | 170 | 153 | 144 | 0.614079 | 1.58E-06 | 5.52E-05 | down |
| ENSMUS( | Kcna5 | 21 | 12 | 15 | 36 | 45 | 47 | 0.386704 | 1.59E-06 | 5.56E-05 | down |
| ENSMUS( | Slc37a2 | 61 | 60 | 56 | 104 | 122 | 89 | 0.585808 | 1.74E-06 | 6.03E-05 | down |
| ENSMUS( | Crabp2 | 43 | 50 | 45 | 103 | 105 | 67 | 0.526742 | 2.23E-06 | 7.49E-05 | down |
| ENSMUS( | Trabd2b | 41 | 33 | 37 | 73 | 80 | 67 | 0.525912 | 2.33E-06 | 7.77E-05 | down |
| ENSMUS( | Gch1 | 5 | 5 | 6 | 27 | 21 | 19 | 0.251275 | 2.68E-06 | 8.71E-05 | down |

| | | | | | | | | | | | |
|---|---|---|---|---|---|---|---|---|---|---|---|
| ENSMUS( | Apobec3 | 72 | 58 | 69 | 114 | 131 | 97 | 0.608925 | 2.76E-06 | 8.94E-05 | down |
| ENSMUS( | Ccl12 | 12 | 11 | 13 | 41 | 27 | 45 | 0.330024 | 2.88E-06 | 9.3E-05 | down |
| ENSMUS( | Nppc | 49 | 54 | 43 | 93 | 87 | 88 | 0.563834 | 3.11E-06 | 9.93E-05 | down |
| ENSMUS( | Sema3g | 31 | 29 | 16 | 61 | 61 | 57 | 0.435493 | 3.16E-06 | 0.000101 | down |
| ENSMUS( | Pcdhga6 | 94 | 69 | 57 | 135 | 148 | 119 | 0.566085 | 3.29E-06 | 0.000104 | down |
| ENSMUS( | Il4ra | 62 | 43 | 55 | 107 | 105 | 92 | 0.549842 | 3.41E-06 | 0.000108 | down |
| ENSMUS( | BC067074 | 50 | 64 | 47 | 92 | 106 | 89 | 0.581001 | 4.16E-06 | 0.000129 | down |
| ENSMUS( | Klhl11 | 161 | 143 | 125 | 211 | 224 | 243 | 0.651837 | 4.26E-06 | 0.000132 | down |
| ENSMUS( | Fcgr2b | 95 | 99 | 71 | 173 | 129 | 143 | 0.615739 | 4.33E-06 | 0.000134 | down |
| ENSMUS( | Sstr4 | 91 | 72 | 67 | 141 | 122 | 116 | 0.630146 | 4.43E-06 | 0.000137 | down |
| ENSMUS( | Ms4a4c | 5 | 7 | 3 | 17 | 21 | 25 | 0.241122 | 5.19E-06 | 0.000157 | down |
| ENSMUS( | Cyp1b1 | 94 | 128 | 96 | 173 | 189 | 153 | 0.641495 | 5.2E-06 | 0.000157 | down |
| ENSMUS( | Adm | 20 | 16 | 10 | 46 | 41 | 31 | 0.403405 | 5.21E-06 | 0.000158 | down |
| ENSMUS( | Ptprc | 108 | 116 | 75 | 178 | 173 | 138 | 0.633152 | 6.07E-06 | 0.000181 | down |
| ENSMUS( | Slfn8 | 8 | 12 | 10 | 32 | 45 | 22 | 0.3185 | 6.56E-06 | 0.000193 | down |
| ENSMUS( | Psmb9 | 37 | 36 | 31 | 75 | 68 | 61 | 0.530019 | 7.22E-06 | 0.00021 | down |
| ENSMUS( | Rin3 | 26 | 29 | 21 | 65 | 53 | 45 | 0.485155 | 7.34E-06 | 0.000213 | down |
| ENSMUS( | Ifitm1 | 86 | 75 | 61 | 134 | 115 | 115 | 0.631204 | 8.37E-06 | 0.00024 | down |
| ENSMUS( | Gpr35 | 5 | 1 | 3 | 14 | 23 | 15 | 0.180556 | 1.01E-05 | 0.000285 | down |
| ENSMUS( | Socs3 | 25 | 34 | 22 | 57 | 55 | 56 | 0.497127 | 1.22E-05 | 0.000334 | down |
| ENSMUS( | Phyhd1 | 7 | 13 | 8 | 24 | 31 | 29 | 0.343111 | 1.48E-05 | 0.000396 | down |
| ENSMUS( | Gm9008 | 3 | 4 | 1 | 18 | 10 | 17 | 0.179547 | 1.54E-05 | 0.000409 | down |
| ENSMUS( | Slc47a1 | 46 | 45 | 31 | 74 | 75 | 76 | 0.557635 | 1.62E-05 | 0.000427 | down |
| ENSMUS( | Fgl2 | 109 | 84 | 110 | 211 | 162 | 161 | 0.594996 | 1.8E-05 | 0.000467 | down |
| ENSMUS( | Hcar2 | 7 | 10 | 1 | 27 | 32 | 35 | 0.19114 | 1.83E-05 | 0.000473 | down |
| ENSMUS( | Klf2 | 63 | 50 | 66 | 109 | 108 | 103 | 0.58456 | 1.89E-05 | 0.000486 | down |
| ENSMUS( | Igtp | 91 | 112 | 77 | 171 | 150 | 121 | 0.658897 | 1.92E-05 | 0.000494 | down |
| ENSMUS( | Htr2a | 57 | 64 | 45 | 98 | 91 | 95 | 0.602796 | 1.96E-05 | 0.0005 | down |
| ENSMUS( | Map3k6 | 32 | 30 | 36 | 72 | 56 | 62 | 0.539255 | 2.02E-05 | 0.000514 | down |
| ENSMUS( | Ifitm6 | 5 | 7 | 6 | 30 | 15 | 23 | 0.275726 | 2.12E-05 | 0.000536 | down |
| ENSMUS( | Hspa12b | 50 | 39 | 34 | 91 | 74 | 60 | 0.569757 | 2.14E-05 | 0.00054 | down |
| ENSMUS( | Tap1 | 84 | 72 | 54 | 114 | 130 | 107 | 0.617785 | 2.28E-05 | 0.000571 | down |
| ENSMUS( | Cd163 | 43 | 29 | 39 | 93 | 92 | 55 | 0.488189 | 2.42E-05 | 0.0006 | down |
| ENSMUS( | Dgkh | 46 | 61 | 49 | 86 | 93 | 103 | 0.57065 | 2.6E-05 | 0.000637 | down |
| ENSMUS( | Fyb | 27 | 36 | 14 | 66 | 60 | 60 | 0.422964 | 2.8E-05 | 0.000677 | down |
| ENSMUS( | Tnxb | 72 | 88 | 70 | 125 | 128 | 112 | 0.654344 | 3.02E-05 | 0.000723 | down |
| ENSMUS( | Hp | 4 | 7 | 4 | 18 | 16 | 24 | 0.263822 | 3.39E-05 | 0.000796 | down |
| ENSMUS( | Tlr13 | 64 | 93 | 64 | 126 | 138 | 110 | 0.613172 | 3.94E-05 | 0.000902 | down |
| ENSMUS( | Aff2 | 49 | 44 | 41 | 71 | 87 | 83 | 0.574364 | 4.19E-05 | 0.000951 | down |
| ENSMUS( | Hk2 | 70 | 73 | 39 | 117 | 109 | 105 | 0.564476 | 4.35E-05 | 0.000981 | down |
| ENSMUS( | Pcdhga4 | 44 | 50 | 40 | 87 | 76 | 72 | 0.592321 | 4.82E-05 | 0.001066 | down |
| ENSMUS( | Ngp | 27 | 14 | 16 | 70 | 53 | 37 | 0.373533 | 5.2E-05 | 0.001139 | down |
| ENSMUS( | Ms4a4b | 2 | 12 | 13 | 39 | 45 | 26 | 0.262208 | 5.35E-05 | 0.001162 | down |
| ENSMUS( | H2-T24 | 44 | 28 | 32 | 74 | 89 | 54 | 0.501418 | 5.78E-05 | 0.001236 | down |
| ENSMUS( | H2-Ab1 | 40 | 38 | 33 | 70 | 76 | 56 | 0.572247 | 6.19E-05 | 0.00131 | down |
| ENSMUS( | Adamts14 | 15 | 11 | 13 | 36 | 37 | 24 | 0.42236 | 6.21E-05 | 0.001313 | down |
| ENSMUS( | Gpr65 | 5 | 2 | 5 | 12 | 18 | 22 | 0.239101 | 6.38E-05 | 0.001343 | down |
| ENSMUS( | Cdh5 | 86 | 118 | 88 | 154 | 156 | 166 | 0.633359 | 6.52E-05 | 0.001367 | down |
| ENSMUS( | Cd52 | 43 | 40 | 44 | 86 | 76 | 61 | 0.597589 | 7.21E-05 | 0.001496 | down |
| ENSMUS( | Mmrn2 | 42 | 55 | 40 | 74 | 83 | 91 | 0.568124 | 7.26E-05 | 0.001504 | down |
| ENSMUS( | Apol11b | 0 | 0 | 1 | 7 | 7 | 13 | 0.040758 | 7.27E-05 | 0.001504 | down |
| ENSMUS( | Ifi44 | 37 | 20 | 25 | 64 | 56 | 57 | 0.480991 | 8.12E-05 | 0.001655 | down |
| ENSMUS( | Tc2n | 45 | 42 | 48 | 76 | 80 | 77 | 0.603588 | 8.3E-05 | 0.001683 | down |
| ENSMUS( | Col12a1 | 147 | 87 | 133 | 210 | 203 | 170 | 0.660176 | 8.73E-05 | 0.001762 | down |
| ENSMUS( | Samd9l | 116 | 93 | 122 | 188 | 171 | 161 | 0.666548 | 8.84E-05 | 0.001784 | down |
| ENSMUS( | Cxcl16 | 46 | 43 | 36 | 78 | 78 | 63 | 0.593246 | 8.88E-05 | 0.001788 | down |
| ENSMUS( | Fas | 33 | 40 | 44 | 63 | 75 | 71 | 0.583385 | 0.000159 | 0.002941 | down |
| ENSMUS( | Slfn2 | 32 | 47 | 47 | 98 | 91 | 61 | 0.532168 | 0.000162 | 0.002988 | down |
| ENSMUS( | Pcna-ps2 | 36 | 35 | 40 | 59 | 75 | 62 | 0.590722 | 0.000172 | 0.003132 | down |
| ENSMUS( | Pcdhga2 | 29 | 37 | 20 | 58 | 58 | 50 | 0.533783 | 0.000223 | 0.003886 | down |
| ENSMUS( | Il1r2 | 3 | 6 | 3 | 16 | 20 | 10 | 0.271742 | 0.00023 | 0.003981 | down |
| ENSMUS( | Helz2 | 50 | 44 | 31 | 78 | 74 | 61 | 0.607372 | 0.00023 | 0.003982 | down |
| ENSMUS( | Fibin | 76 | 47 | 58 | 114 | 94 | 103 | 0.604767 | 0.000235 | 0.004058 | down |
| ENSMUS( | Anpep | 43 | 36 | 48 | 72 | 79 | 72 | 0.595354 | 0.000237 | 0.004086 | down |
| ENSMUS( | Palm2 | 24 | 41 | 37 | 68 | 65 | 66 | 0.534321 | 0.000243 | 0.004175 | down |
| ENSMUS( | Ston1 | 52 | 42 | 37 | 77 | 68 | 92 | 0.567259 | 0.000248 | 0.004247 | down |
| ENSMUS( | Csf2rb | 18 | 20 | 28 | 52 | 46 | 65 | 0.421886 | 0.000253 | 0.004324 | down |
| ENSMUS( | Phf11d | 30 | 44 | 29 | 72 | 72 | 46 | 0.566049 | 0.000265 | 0.004492 | down |
| ENSMUS( | Cxcl13 | 9 | 6 | 5 | 24 | 20 | 16 | 0.346325 | 0.000278 | 0.004663 | down |
| ENSMUS( | Crispld2 | 75 | 45 | 68 | 123 | 108 | 94 | 0.607118 | 0.000279 | 0.004672 | down |
| ENSMUS( | Ifi207 | 13 | 10 | 11 | 34 | 26 | 23 | 0.428919 | 0.000282 | 0.004716 | down |
| ENSMUS( | Cklf | 54 | 58 | 54 | 87 | 85 | 93 | 0.648707 | 0.00031 | 0.005124 | down |
| ENSMUS( | Ms4a1 | 0 | 1 | 1 | 4 | 4 | 18 | 0.077818 | 0.000316 | 0.0052 | down |
| ENSMUS( | Ly9 | 1 | 4 | 4 | 12 | 13 | 14 | 0.241774 | 0.000327 | 0.005355 | down |
| ENSMUS( | Lilrb4a | 3 | 6 | 8 | 21 | 23 | 13 | 0.31882 | 0.00039 | 0.006198 | down |
| ENSMUS( | Ch25h | 9 | 8 | 11 | 30 | 24 | 18 | 0.411836 | 0.000408 | 0.00644 | down |
| ENSMUS( | C5ar1 | 7 | 7 | 8 | 18 | 24 | 20 | 0.369871 | 0.000415 | 0.006537 | down |
| ENSMUS( | Igsf6 | 25 | 10 | 12 | 42 | 45 | 34 | 0.402678 | 0.000435 | 0.006779 | down |
| ENSMUS( | Gpr84 | 4 | 11 | 8 | 24 | 24 | 20 | 0.353429 | 0.000444 | 0.006892 | down |
| ENSMUS( | Sdk1 | 42 | 45 | 37 | 83 | 79 | 52 | 0.606572 | 0.000482 | 0.007335 | down |
| ENSMUS( | Pkp3 | 1 | 2 | 1 | 8 | 11 | 8 | 0.152705 | 0.000512 | 0.007718 | down |
| ENSMUS( | Lama1 | 50 | 49 | 43 | 72 | 87 | 71 | 0.640385 | 0.000521 | 0.007839 | down |
| ENSMUS( | Hs3st3a1 | 22 | 32 | 20 | 42 | 46 | 52 | 0.541882 | 0.000523 | 0.007855 | down |

| | | | | | | | | | | | | |
|---|---|---|---|---|---|---|---|---|---|---|---|---|
| ENSMUS( | Tgtp2 | 60 | 70 | 56 | 91 | 97 | 103 | 0.659396 | 0.000563 | 0.008332 | down |
| ENSMUS( | Pcdhga7 | 68 | 57 | 32 | 98 | 95 | 82 | 0.586807 | 0.00057 | 0.008393 | down |
| ENSMUS( | Vash1 | 50 | 48 | 64 | 102 | 104 | 72 | 0.615597 | 0.000573 | 0.00843 | down |
| ENSMUS( | Ifi206 | 3 | 6 | 4 | 19 | 12 | 14 | 0.30061 | 0.00059 | 0.008635 | down |
| ENSMUS( | Sln | 14 | 18 | 6 | 37 | 41 | 23 | 0.387538 | 0.000593 | 0.008661 | down |
| ENSMUS( | Ifi213 | 13 | 9 | 11 | 34 | 38 | 15 | 0.402129 | 0.000611 | 0.008866 | down |
| ENSMUS( | Slc10a6 | 1 | 2 | 3 | 7 | 11 | 13 | 0.202308 | 0.00062 | 0.008951 | down |
| ENSMUS( | Hs6st3 | 30 | 34 | 24 | 53 | 56 | 48 | 0.579865 | 0.000627 | 0.009045 | down |
| ENSMUS( | Cd244 | 0 | 0 | 2 | 16 | 12 | 18 | 0.04846 | 0.000636 | 0.009149 | down |
| ENSMUS( | Tlr7 | 63 | 103 | 78 | 152 | 137 | 105 | 0.647912 | 0.000642 | 0.009224 | down |
| ENSMUS( | Sphk1 | 30 | 46 | 32 | 60 | 80 | 55 | 0.575364 | 0.000655 | 0.009355 | down |
| ENSMUS( | Slfn1 | 7 | 2 | 6 | 18 | 18 | 16 | 0.303331 | 0.00067 | 0.009539 | down |
| ENSMUS( | Wnt5b | 21 | 18 | 26 | 44 | 54 | 32 | 0.52882 | 0.000685 | 0.009718 | down |
| ENSMUS( | Klrk1 | 3 | 4 | 0 | 16 | 10 | 17 | 0.161432 | 0.000723 | 0.010187 | down |
| ENSMUS( | Jaml | 4 | 4 | 5 | 25 | 16 | 8 | 0.284313 | 0.000738 | 0.010325 | down |
| ENSMUS( | Clec4e | 0 | 1 | 0 | 5 | 7 | 6 | 0.055153 | 0.00076 | 0.010554 | down |
| ENSMUS( | Slc4a1 | 3 | 3 | 1 | 6 | 15 | 13 | 0.206843 | 0.000773 | 0.01071 | down |
| ENSMUS( | Zbp1 | 16 | 22 | 6 | 48 | 32 | 32 | 0.401772 | 0.000777 | 0.01074 | down |
| ENSMUS( | Pcdhga5 | 48 | 48 | 40 | 82 | 65 | 72 | 0.643315 | 0.000819 | 0.011214 | down |
| ENSMUS( | Cd300lf | 43 | 28 | 27 | 79 | 49 | 64 | 0.528195 | 0.000862 | 0.011728 | down |
| ENSMUS( | Krt9 | 15 | 15 | 23 | 46 | 35 | 34 | 0.487612 | 0.000885 | 0.011999 | down |
| ENSMUS( | Cd4 | 1 | 3 | 6 | 17 | 18 | 16 | 0.210494 | 0.000917 | 0.012312 | down |
| ENSMUS( | Cdkn1c | 42 | 40 | 35 | 79 | 62 | 52 | 0.633312 | 0.000968 | 0.012866 | down |
| ENSMUS( | Foxc2 | 12 | 9 | 7 | 32 | 30 | 13 | 0.392284 | 0.000972 | 0.012908 | down |
| ENSMUS( | Tmppe | 25 | 34 | 14 | 56 | 46 | 48 | 0.498267 | 0.001009 | 0.01332 | down |
| ENSMUS( | Ifi211 | 5 | 7 | 5 | 17 | 17 | 16 | 0.352035 | 0.001121 | 0.014515 | down |
| ENSMUS( | Cd8b1 | 3 | 3 | 1 | 6 | 16 | 11 | 0.214302 | 0.001164 | 0.014967 | down |
| ENSMUS( | Alx4 | 29 | 28 | 20 | 61 | 49 | 34 | 0.558334 | 0.001219 | 0.015556 | down |
| ENSMUS( | Retnlg | 8 | 5 | 6 | 15 | 22 | 16 | 0.372499 | 0.001255 | 0.015933 | down |
| ENSMUS( | Adamts17 | 58 | 48 | 45 | 85 | 75 | 75 | 0.666512 | 0.00129 | 0.016257 | down |
| ENSMUS( | Piezo1 | 54 | 74 | 41 | 95 | 94 | 108 | 0.582326 | 0.001311 | 0.016485 | down |
| ENSMUS( | Gbp6 | 21 | 21 | 20 | 44 | 37 | 36 | 0.552143 | 0.001331 | 0.016673 | down |
| ENSMUS( | 8430408G: | 30 | 20 | 17 | 45 | 43 | 39 | 0.545071 | 0.001335 | 0.016704 | down |
| ENSMUS( | Cyp4f17 | 57 | 36 | 54 | 94 | 73 | 82 | 0.617208 | 0.001406 | 0.017424 | down |
| ENSMUS( | Soga1 | 98 | 104 | 60 | 137 | 150 | 164 | 0.593397 | 0.001436 | 0.017697 | down |
| ENSMUS( | Itgal | 16 | 14 | 8 | 34 | 32 | 18 | 0.470484 | 0.001439 | 0.017729 | down |
| ENSMUS( | Zdhhc23 | 89 | 59 | 54 | 108 | 105 | 108 | 0.648727 | 0.001582 | 0.01912 | down |
| ENSMUS( | Mpzl2 | 4 | 3 | 2 | 16 | 5 | 17 | 0.240521 | 0.001587 | 0.019157 | down |
| ENSMUS( | Kcna3 | 11 | 6 | 8 | 18 | 19 | 27 | 0.400773 | 0.001625 | 0.019544 | down |
| ENSMUS( | Itgad | 4 | 2 | 3 | 13 | 17 | 6 | 0.265027 | 0.001669 | 0.01998 | down |
| ENSMUS( | Rnf207 | 42 | 44 | 34 | 69 | 70 | 54 | 0.646397 | 0.001715 | 0.020451 | down |
| ENSMUS( | Itpr3 | 33 | 26 | 43 | 71 | 60 | 57 | 0.572409 | 0.001724 | 0.020538 | down |
| ENSMUS( | Gpr182 | 45 | 28 | 40 | 62 | 74 | 59 | 0.605508 | 0.001846 | 0.021743 | down |
| ENSMUS( | Pcdhga3 | 48 | 38 | 31 | 56 | 77 | 60 | 0.625476 | 0.001911 | 0.022377 | down |
| ENSMUS( | Pcdhga9 | 48 | 41 | 26 | 70 | 72 | 53 | 0.609626 | 0.001926 | 0.022519 | down |
| ENSMUS( | Clec10a | 14 | 21 | 23 | 48 | 47 | 26 | 0.509282 | 0.001949 | 0.022766 | down |
| ENSMUS( | Plaur | 7 | 7 | 6 | 18 | 22 | 13 | 0.394399 | 0.00199 | 0.023123 | down |
| ENSMUS( | Fstl4 | 42 | 41 | 44 | 68 | 66 | 67 | 0.657534 | 0.002002 | 0.023221 | down |
| ENSMUS( | Cd248 | 31 | 26 | 25 | 50 | 49 | 43 | 0.60038 | 0.002003 | 0.023221 | down |
| ENSMUS( | Gzma | 2 | 2 | 5 | 15 | 21 | 5 | 0.239953 | 0.00201 | 0.023281 | down |
| ENSMUS( | Gm694 | 17 | 15 | 16 | 33 | 32 | 30 | 0.526224 | 0.00207 | 0.023864 | down |
| ENSMUS( | Pcdhga1 | 32 | 23 | 16 | 54 | 46 | 34 | 0.549685 | 0.002113 | 0.024247 | down |
| ENSMUS( | Gata2 | 33 | 31 | 26 | 52 | 44 | 69 | 0.558097 | 0.002126 | 0.024369 | down |
| ENSMUS( | Rasgef1c | 20 | 30 | 25 | 38 | 47 | 47 | 0.587661 | 0.002215 | 0.025163 | down |
| ENSMUS( | Abcc3 | 26 | 19 | 24 | 61 | 33 | 39 | 0.54471 | 0.00237 | 0.026508 | down |
| ENSMUS( | Fhdc1 | 41 | 39 | 39 | 57 | 75 | 58 | 0.651562 | 0.002411 | 0.026901 | down |
| ENSMUS( | Nlrc5 | 17 | 36 | 16 | 46 | 51 | 45 | 0.499044 | 0.002568 | 0.028264 | down |
| ENSMUS( | Car13 | 44 | 36 | 30 | 64 | 68 | 45 | 0.64738 | 0.002649 | 0.028964 | down |
| ENSMUS( | Gm1604a | 28 | 15 | 18 | 42 | 34 | 46 | 0.515075 | 0.002725 | 0.029666 | down |
| ENSMUS( | Tgtp1 | 2 | 4 | 2 | 14 | 10 | 7 | 0.269497 | 0.002805 | 0.030367 | down |
| ENSMUS( | Casp1 | 60 | 53 | 52 | 78 | 82 | 102 | 0.648118 | 0.002852 | 0.030803 | down |
| ENSMUS( | Dennd2c | 9 | 9 | 6 | 20 | 17 | 21 | 0.424414 | 0.002861 | 0.030853 | down |
| ENSMUS( | Rbp7 | 4 | 4 | 3 | 18 | 8 | 11 | 0.309415 | 0.00287 | 0.030934 | down |
| ENSMUS( | Ptgfr | 27 | 16 | 18 | 52 | 35 | 30 | 0.546103 | 0.002991 | 0.031971 | down |
| ENSMUS( | Rab32 | 42 | 43 | 35 | 61 | 70 | 58 | 0.658004 | 0.003017 | 0.032168 | down |
| ENSMUS( | Rnf125 | 9 | 9 | 3 | 22 | 15 | 18 | 0.388777 | 0.003078 | 0.032648 | down |
| ENSMUS( | A4galt | 18 | 21 | 19 | 47 | 33 | 28 | 0.563956 | 0.003099 | 0.032821 | down |
| ENSMUS( | Pirb | 2 | 10 | 8 | 33 | 15 | 25 | 0.287365 | 0.003136 | 0.033047 | down |
| ENSMUS( | Cxcr6 | 5 | 2 | 4 | 10 | 12 | 14 | 0.316299 | 0.003256 | 0.034093 | down |
| ENSMUS( | Cntn5 | 44 | 45 | 33 | 60 | 64 | 67 | 0.656894 | 0.003266 | 0.034173 | down |
| ENSMUS( | Best3 | 10 | 5 | 10 | 19 | 17 | 23 | 0.441013 | 0.003277 | 0.034262 | down |
| ENSMUS( | Slfn4 | 5 | 4 | 2 | 11 | 17 | 8 | 0.315392 | 0.003307 | 0.034488 | down |
| ENSMUS( | Myo1f | 44 | 40 | 26 | 52 | 66 | 59 | 0.637406 | 0.003353 | 0.034897 | down |
| ENSMUS( | Dok6 | 47 | 76 | 32 | 86 | 104 | 106 | 0.533263 | 0.003401 | 0.035285 | down |
| ENSMUS( | Mylk2 | 2 | 3 | 3 | 16 | 15 | 3 | 0.253598 | 0.003552 | 0.036519 | down |
| ENSMUS( | Ly6c2 | 54 | 47 | 49 | 81 | 67 | 88 | 0.657182 | 0.003578 | 0.036728 | down |
| ENSMUS( | Pgr15l | 1 | 3 | 0 | 8 | 13 | 9 | 0.133016 | 0.003695 | 0.037669 | down |
| ENSMUS( | Reg3b | 2 | 5 | 2 | 11 | 9 | 12 | 0.286797 | 0.003716 | 0.037832 | down |
| ENSMUS( | Fam180a | 38 | 43 | 31 | 81 | 56 | 49 | 0.628234 | 0.003813 | 0.038608 | down |
| ENSMUS( | Sec14l5 | 26 | 39 | 26 | 67 | 44 | 44 | 0.611536 | 0.003861 | 0.039031 | down |
| ENSMUS( | Sycp1 | 5 | 6 | 3 | 20 | 11 | 10 | 0.354955 | 0.003869 | 0.039075 | down |
| ENSMUS( | Zfp90 | 17 | 22 | 19 | 44 | 37 | 25 | 0.575667 | 0.003924 | 0.039539 | down |
| ENSMUS( | Tnfrsf11b | 18 | 30 | 16 | 43 | 34 | 44 | 0.54229 | 0.003956 | 0.039797 | down |

| | | | | | | | | | | | |
|---|---|---|---|---|---|---|---|---|---|---|---|
| ENSMUS( | Dll4 | 12 | 17 | 10 | 32 | 27 | 20 | 0.513743 | 0.003962 | 0.039842 | down |
| ENSMUS( | Il1rn | 0 | 0 | 1 | 11 | 4 | 2 | 0.068293 | 0.004119 | 0.041163 | down |
| ENSMUS( | Fzd10 | 10 | 14 | 11 | 23 | 28 | 22 | 0.498249 | 0.004131 | 0.041219 | down |
| ENSMUS( | Tead4 | 4 | 2 | 0 | 10 | 10 | 15 | 0.16915 | 0.004174 | 0.041574 | down |
| ENSMUS( | Madcam1 | 7 | 6 | 4 | 24 | 14 | 10 | 0.370875 | 0.004184 | 0.04165 | down |
| ENSMUS( | Krt80 | 0 | 0 | 1 | 6 | 6 | 3 | 0.07604 | 0.004398 | 0.043298 | down |
| ENSMUS( | Gm9733 | 0 | 0 | 1 | 6 | 5 | 4 | 0.075472 | 0.004411 | 0.043367 | down |
| ENSMUS( | Clec4a1 | 11 | 16 | 7 | 24 | 30 | 18 | 0.487424 | 0.00446 | 0.043724 | down |
| ENSMUS( | Cfi | 1 | 1 | 0 | 5 | 5 | 7 | 0.11621 | 0.004459 | 0.043724 | down |
| ENSMUS( | Cyp4f18 | 0 | 1 | 1 | 9 | 4 | 4 | 0.126653 | 0.004523 | 0.044227 | down |
| ENSMUS( | Arhgef37 | 16 | 12 | 7 | 22 | 23 | 28 | 0.48712 | 0.004641 | 0.045226 | down |
| ENSMUS( | Gbp2b | 3 | 3 | 4 | 14 | 11 | 8 | 0.321503 | 0.00486 | 0.046704 | down |
| ENSMUS( | Ifi44l | 2 | 3 | 0 | 10 | 5 | 13 | 0.175628 | 0.004865 | 0.046734 | down |
| ENSMUS( | Casp4 | 6 | 13 | 15 | 41 | 22 | 25 | 0.410307 | 0.004921 | 0.047073 | down |
| ENSMUS( | F10 | 0 | 1 | 0 | 6 | 10 | 0 | 0.064641 | 0.005047 | 0.04789 | down |
| ENSMUS( | Gimap4 | 22 | 25 | 28 | 52 | 38 | 37 | 0.620785 | 0.005062 | 0.047984 | down |
| ENSMUS( | Lefty2 | 14 | 13 | 15 | 24 | 34 | 24 | 0.535337 | 0.005149 | 0.048649 | down |
| ENSMUS( | Ptpn7 | 0 | 1 | 0 | 6 | 5 | 3 | 0.072221 | 0.00517 | 0.048803 | down |
| ENSMUS( | Gpr63 | 53 | 56 | 47 | 107 | 62 | 78 | 0.657011 | 0.005194 | 0.048962 | down |
| ENSMUS( | Pmaip1 | 34 | 28 | 30 | 53 | 48 | 48 | 0.642277 | 0.005276 | 0.049482 | down |
| ENSMUS( | Tmem71 | 11 | 5 | 7 | 23 | 17 | 14 | 0.446316 | 0.005273 | 0.049482 | down |
| ENSMUS( | Sost | 1 | 1 | 1 | 6 | 5 | 8 | 0.162354 | 0.005276 | 0.049482 | down |
| ENSMUS( | Dysf | 32 | 28 | 17 | 41 | 52 | 39 | 0.600049 | 0.005342 | 0.049924 | down |
| ENSMUS( | Rpl35a | 2323 | 2522 | 2269 | 1102 | 1045 | 926 | 2.411185 | 1.4E-151 | 1.4E-147 | up |
| ENSMUS( | Cck | 3134 | 4433 | 6458 | 2934 | 2666 | 2394 | 1.8587 | 1.51E-63 | 3.32E-60 | up |
| ENSMUS( | Fabp7 | 1044 | 1160 | 1061 | 561 | 545 | 438 | 2.208717 | 7E-56 | 1.11E-52 | up |
| ENSMUS( | Gm4737 | 188 | 175 | 151 | 47 | 53 | 38 | 3.877283 | 1.85E-39 | 1.82E-36 | up |
| ENSMUS( | Pcsk1n | 2053 | 2637 | 3240 | 1801 | 1808 | 1679 | 1.574622 | 3.11E-34 | 2.34E-31 | up |
| ENSMUS( | P2ry12 | 1611 | 1747 | 1542 | 1229 | 1149 | 991 | 1.515622 | 3.21E-34 | 2.36E-31 | up |
| ENSMUS( | Ccdc85b | 541 | 765 | 1151 | 473 | 466 | 441 | 1.882288 | 6.2E-32 | 4.22E-29 | up |
| ENSMUS( | Rps2 | 3617 | 3924 | 3945 | 2823 | 2747 | 2309 | 1.524199 | 1.78E-30 | 1.11E-27 | up |
| ENSMUS( | Cox5a | 335 | 623 | 1062 | 297 | 264 | 285 | 2.536666 | 6.52E-26 | 3.01E-23 | up |
| ENSMUS( | Ugt8a | 1025 | 1107 | 878 | 696 | 646 | 644 | 1.569023 | 6.42E-25 | 2.87E-22 | up |
| ENSMUS( | Rsph1 | 425 | 442 | 440 | 299 | 255 | 235 | 1.730904 | 1.35E-20 | 4.28E-18 | up |
| ENSMUS( | Myoc | 166 | 184 | 180 | 79 | 84 | 78 | 2.288125 | 3.78E-20 | 1.15E-17 | up |
| ENSMUS( | Erdr1 | 171 | 237 | 292 | 113 | 105 | 98 | 2.331407 | 1.14E-18 | 3.01E-16 | up |
| ENSMUS( | Gpr34 | 461 | 472 | 380 | 290 | 293 | 263 | 1.608347 | 1.84E-18 | 4.69E-16 | up |
| ENSMUS( | Gm1673 | 25 | 114 | 194 | 26 | 22 | 13 | 5.931983 | 1.1E-17 | 2.52E-15 | up |
| ENSMUS( | D830030K | 33 | 21 | 25 | 0 | 1 | 1 | 39.79852 | 2.06E-17 | 4.68E-15 | up |
| ENSMUS( | C1qtnf4 | 407 | 556 | 723 | 380 | 367 | 332 | 1.64698 | 6.19E-16 | 1.2E-13 | up |
| ENSMUS( | Inhba | 241 | 289 | 205 | 149 | 131 | 118 | 1.916105 | 1.31E-15 | 2.45E-13 | up |
| ENSMUS( | Fcrls | 451 | 475 | 425 | 337 | 294 | 292 | 1.521551 | 3.16E-15 | 5.57E-13 | up |
| ENSMUS( | Akr1e1 | 279 | 286 | 283 | 199 | 164 | 162 | 1.685187 | 8.99E-15 | 1.49E-12 | up |
| ENSMUS( | Emc6 | 604 | 735 | 764 | 513 | 525 | 424 | 1.507487 | 1.1E-13 | 1.58E-11 | up |
| ENSMUS( | Opalin | 541 | 568 | 463 | 391 | 343 | 276 | 1.623043 | 1.38E-13 | 1.93E-11 | up |
| ENSMUS( | Rpl13 | 5194 | 7924 | 8080 | 3846 | 3938 | 3984 | 1.879746 | 9.25E-12 | 9.92E-10 | up |
| ENSMUS( | 5430403G | 110 | 130 | 100 | 60 | 52 | 58 | 2.067174 | 2.52E-11 | 2.52E-09 | up |
| ENSMUS( | Muc3a | 126 | 91 | 109 | 61 | 47 | 49 | 2.167085 | 4.03E-10 | 3.23E-08 | up |
| ENSMUS( | Hist1h2be | 118 | 122 | 107 | 70 | 63 | 60 | 1.868619 | 3.68E-09 | 2.5E-07 | up |
| ENSMUS( | Gm3636 | 94 | 109 | 80 | 53 | 34 | 46 | 2.198466 | 5.21E-08 | 2.68E-06 | up |
| ENSMUS( | Gm12355 | 129 | 75 | 95 | 54 | 50 | 46 | 2.075998 | 9.95E-08 | 4.82E-06 | up |
| ENSMUS( | Gm3468 | 11 | 14 | 15 | 0 | 2 | 1 | 13.76609 | 1.18E-07 | 5.62E-06 | up |
| ENSMUS( | mt-Nd4l | 45 | 0 | 4 | 1 | 2 | 0 | 17.14013 | 1.71E-07 | 7.84E-06 | up |
| ENSMUS( | Pyurf | 217 | 301 | 231 | 166 | 189 | 161 | 1.505956 | 4.75E-07 | 1.93E-05 | up |
| ENSMUS( | Mrps34 | 19 | 62 | 119 | 17 | 13 | 22 | 4.075931 | 4.95E-07 | 2E-05 | up |
| ENSMUS( | Smyd1 | 33 | 41 | 26 | 11 | 10 | 14 | 2.918124 | 6.47E-07 | 2.55E-05 | up |
| ENSMUS( | Susd3 | 54 | 40 | 34 | 21 | 19 | 14 | 2.468217 | 1.11E-06 | 4.07E-05 | up |
| ENSMUS( | Thoc6 | 102 | 102 | 103 | 68 | 66 | 53 | 1.71715 | 1.27E-06 | 4.56E-05 | up |
| ENSMUS( | 1110065P2 | 89 | 150 | 208 | 98 | 86 | 90 | 1.722993 | 1.77E-06 | 6.14E-05 | up |
| ENSMUS( | Ppp1r14a | 87 | 125 | 154 | 83 | 78 | 57 | 1.778679 | 5.64E-06 | 0.00017 | up |
| ENSMUS( | Smim18 | 97 | 104 | 98 | 66 | 71 | 52 | 1.653809 | 7.25E-06 | 0.000211 | up |
| ENSMUS( | AC123954 | 25 | 30 | 26 | 11 | 9 | 9 | 2.908632 | 1.09E-05 | 0.000303 | up |
| ENSMUS( | Aif1 | 135 | 156 | 116 | 103 | 102 | 76 | 1.507954 | 1.21E-05 | 0.000333 | up |
| ENSMUS( | Xlr3b | 54 | 69 | 61 | 37 | 37 | 28 | 1.887288 | 1.42E-05 | 0.000382 | up |
| ENSMUS( | Gm2237 | 28 | 15 | 27 | 6 | 10 | 4 | 3.711481 | 1.63E-05 | 0.000429 | up |
| ENSMUS( | Gjc2 | 79 | 84 | 69 | 51 | 49 | 42 | 1.698433 | 2.81E-05 | 0.000677 | up |
| ENSMUS( | Gm3248 | 13 | 13 | 10 | 2 | 2 | 2 | 6.191896 | 3.2E-05 | 0.000759 | up |
| ENSMUS( | Lmcd1 | 122 | 107 | 102 | 74 | 80 | 72 | 1.519339 | 5.02E-05 | 0.001103 | up |
| ENSMUS( | Zfp580 | 141 | 200 | 191 | 102 | 90 | 112 | 1.816 | 6.16E-05 | 0.001304 | up |
| ENSMUS( | Gm8281 | 25 | 33 | 20 | 8 | 3 | 0 | 7.680275 | 8.25E-05 | 0.001677 | up |
| ENSMUS( | Gm5148 | 18 | 4 | 7 | 1 | 1 | 1 | 9.946736 | 0.000125 | 0.00238 | up |
| ENSMUS( | Mien1 | 112 | 125 | 162 | 99 | 93 | 86 | 1.5089 | 0.000343 | 0.005568 | up |
| ENSMUS( | Fbxl15 | 92 | 146 | 163 | 85 | 79 | 92 | 1.635969 | 0.000358 | 0.005765 | up |
| ENSMUS( | Xkr6 | 74 | 71 | 80 | 58 | 45 | 47 | 1.568684 | 0.000415 | 0.006537 | up |
| ENSMUS( | Gm3667 | 25 | 33 | 20 | 5 | 14 | 8 | 2.970491 | 0.000502 | 0.007586 | up |
| ENSMUS( | Rnf26 | 8 | 19 | 20 | 6 | 4 | 4 | 3.553287 | 0.000523 | 0.007855 | up |
| ENSMUS( | Zfp771 | 102 | 166 | 211 | 67 | 77 | 92 | 2.117833 | 0.000546 | 0.008132 | up |
| ENSMUS( | Gsx1 | 8 | 9 | 14 | 2 | 4 | 1 | 4.758036 | 0.000603 | 0.008775 | up |
| ENSMUS( | Oxld1 | 73 | 123 | 124 | 83 | 64 | 70 | 1.545443 | 0.000721 | 0.010167 | up |
| ENSMUS( | Malsu1 | 110 | 118 | 124 | 98 | 82 | 57 | 1.566568 | 0.000729 | 0.010227 | up |
| ENSMUS( | H2-Ob | 27 | 16 | 22 | 9 | 11 | 8 | 2.426166 | 0.000731 | 0.010239 | up |
| ENSMUS( | Acad10 | 71 | 65 | 63 | 39 | 47 | 46 | 1.558601 | 0.00079 | 0.010871 | up |
| ENSMUS( | Scand1 | 1 | 13 | 51 | 5 | 3 | 8 | 4.360909 | 0.000838 | 0.011438 | up |

| | | | | | | | | | | | |
|---|---|---|---|---|---|---|---|---|---|---|---|
| ENSMUS( | Endog | 34 | 32 | 68 | 23 | 29 | 16 | 2.112965 | 0.000852 | 0.011608 | up |
| ENSMUS( | Eme2 | 53 | 49 | 44 | 38 | 24 | 24 | 1.774074 | 0.000884 | 0.011999 | up |
| ENSMUS( | Stab2 | 90 | 58 | 66 | 49 | 23 | 31 | 2.170897 | 0.001085 | 0.014144 | up |
| ENSMUS( | Zfp428 | 167 | 293 | 299 | 156 | 165 | 179 | 1.582123 | 0.001172 | 0.015044 | up |
| ENSMUS( | Pycr1 | 50 | 36 | 22 | 19 | 19 | 17 | 2.015474 | 0.001235 | 0.01572 | up |
| ENSMUS( | Pspn | 10 | 7 | 10 | 1 | 2 | 3 | 4.586985 | 0.001375 | 0.017101 | up |
| ENSMUS( | Depdc7 | 55 | 70 | 57 | 45 | 42 | 34 | 1.569544 | 0.001421 | 0.017561 | up |
| ENSMUS( | Gm5415 | 39 | 51 | 54 | 31 | 32 | 25 | 1.718399 | 0.001628 | 0.01956 | up |
| ENSMUS( | Prss41 | 49 | 61 | 51 | 26 | 21 | 38 | 1.937191 | 0.00184 | 0.021682 | up |
| ENSMUS( | Dmkn | 38 | 30 | 37 | 24 | 19 | 10 | 2.105288 | 0.001988 | 0.023109 | up |
| ENSMUS( | Krt15 | 36 | 27 | 28 | 15 | 20 | 15 | 1.890414 | 0.002129 | 0.024394 | up |
| ENSMUS( | Tnfaip8l2 | 55 | 49 | 52 | 42 | 25 | 22 | 1.847181 | 0.002192 | 0.024962 | up |
| ENSMUS( | Kif22 | 57 | 64 | 75 | 48 | 38 | 45 | 1.563194 | 0.00243 | 0.027048 | up |
| ENSMUS( | Hspb3 | 13 | 8 | 15 | 5 | 3 | 4 | 3.157367 | 0.002507 | 0.027732 | up |
| ENSMUS( | Gm45844 | 65 | 53 | 39 | 31 | 40 | 30 | 1.603967 | 0.002533 | 0.027941 | up |
| ENSMUS( | DHRSX | 46 | 64 | 54 | 26 | 38 | 37 | 1.675163 | 0.00268 | 0.029249 | up |
| ENSMUS( | Otos | 7 | 14 | 6 | 1 | 3 | 3 | 3.885554 | 0.003115 | 0.032913 | up |
| ENSMUS( | Ckap2 | 31 | 26 | 28 | 13 | 19 | 7 | 2.302813 | 0.00342 | 0.035415 | up |
| ENSMUS( | Nr2c2ap | 17 | 23 | 18 | 8 | 7 | 12 | 2.195072 | 0.003629 | 0.037193 | up |
| ENSMUS( | Adora2a | 31 | 25 | 37 | 19 | 16 | 12 | 1.935966 | 0.004262 | 0.042263 | up |
| ENSMUS( | Rtl1 | 55 | 58 | 65 | 51 | 33 | 28 | 1.679462 | 0.004273 | 0.042302 | up |
| ENSMUS( | Avil | 4 | 8 | 6 | 1 | 1 | 1 | 6.228978 | 0.00472 | 0.045738 | up |
| ENSMUS( | Colq | 13 | 6 | 10 | 5 | 2 | 2 | 3.42155 | 0.005062 | 0.047984 | up |

**Dataset S4. KEGG enrichment analysis for DE genes between RS and control (RS/C) female offspring**

| Term | Count | Fold Enric | PValue | FDR | Genes |
|---|---|---|---|---|---|
| ECM-recep | 15 | 3.890107 | 2.40E-05 | 0.030992 | COL4A4, COL4A3, COL6A5, LAMA5, GP1BB, COL27A1, ITGA8, COL6A3, ITGA1, COL1A1, COL11A2, COL11A1, COL5A2, THBS3, SPP1 |
| Protein dig | 15 | 3.890107 | 2.40E-05 | 0.030992 | COL4A4, COL4A3, SLC8A1, SLC3A1, COL5A2, KCNJ13, COL9A2, COL9A3, COL14A1, COL6A5, COL27A1, COL6A3, COL1A1, COL11A2, COL11A1 |
| Cell adhes | 18 | 2.535773 | 6.80E-04 | 0.875469 | H2-K1, H2-Q2, ITGAL, F11R, OCLN, CLDN9, CLDN3, CLDN5, H2-D1, CDH1, CDH3, H2-Q6, NCAM2, ITGA8, CLDN1, CLDN2, H2-AA, H2-DMA |
| Focal adhe | 19 | 2.094769 | 0.004035 | 5.091959 | COL4A4, COL4A3, MYLK3, ITGA1, COL5A2, FLNB, VCL, VEGFB, COL6A5, LAMA5, COL27A1, ITGA8, COL6A3, ZYX, COL1A1, COL11A2, COL11A1, THBS3, SPP1 |
| Mineral ab | 7 | 4.096249 | 0.0064 | 7.963043 | TRPM7, MT1, SLC39A4, STEAP2, STEAP1, SLC31A1, FTH1 |
| Neuroactiv | 23 | 1.841772 | 0.006596 | 8.197113 | C3AR1, GABRB2, LEPR, GABRA6, PTH1R, HTR4, SSTR4, CRHR2, CHRM5, PRLR, GIPR, ADRA2A, ADRA1B, CHRNB4, GPR50, CHRNA7, MC3R, GLP2R, GHSR, HTR2C, CHRNA3, OPRD1, HTR2A |
| HTLV-I in | 22 | 1.819142 | 0.009212 | 11.27372 | H2-Q2, H2-K1, ITGAL, WNT10A, EGR2, NRP1, WNT3A, H2-D1, CHEK1, ATR, H2-Q6, ATM, TGFB1, WNT1, FOS, MSX1, H2-AA, WNT6, H2-DMA, TERT, CHUK, WNT8B |
| Leukocyte | 12 | 2.320877 | 0.013821 | 16.46268 | ITGAL, ITK, F11R, CLDN9, OCLN, NCF2, CLDN3, NCF4, CLDN5, CLDN1, CLDN2, VCL |
| Tight junct | 10 | 2.535773 | 0.016349 | 19.18836 | F11R, CLDN9, OCLN, CLDN3, CGN, CLDN5, CLDN1, CLDN2, CRB3, TJP3 |
| Viral myoc | 9 | 2.59997 | 0.021443 | 24.43317 | H2-K1, H2-Q2, ITGAL, CD55, H2-D1, H2-AA, MYH7, H2-DMA, H2-Q6 |
| Basal cell | 7 | 2.958402 | 0.029409 | 32.00908 | WNT10A, WNT1, WNT3A, WNT6, TCF7L2, TCF7L1, WNT8B |
| Amoebiasi | 11 | 2.145654 | 0.03156 | 33.93095 | COL4A4, COL4A3, LAMA5, COL27A1, COL1A1, SERPINB1B, COL11A2, COL11A1, COL5A2, TGFB1, VCL |
| Salivary se | 8 | 2.371113 | 0.050327 | 48.69553 | KCNMA1, LYZ2, ATP2B3, SLC12A2, RYR3, ADRA1B, SLC4A2, PRKG1 |
| Malaria | 6 | 2.852745 | 0.057033 | 53.18522 | HBA-A1, ITGAL, HBB-BS, HBB-BT, THBS3, TGFB1 |
| Pathways i | 25 | 1.444428 | 0.062306 | 56.45845 | COL4A4, DCC, WNT10A, COL4A3, RET, FLT3, WNT3A, MITF, BIRC7, CDH1, ZBTB16, MECOM, TCF7L2, TCF7L1, TGFB1, VEGFB, JUP, GNG8, WNT1, FOS, LAMA5, WNT6, GM5741, CHUK, WNT8B |
| Staphyloco | 6 | 2.738635 | 0.06584 | 58.53246 | ITGAL, C3AR1, MASP2, H2-AA, H2-DMA, C1QC |
| Rheumatoi | 8 | 2.226533 | 0.066373 | 58.83684 | ITGAL, FOS, CXCL5, H2-AA, ATP6V1G2, CCL5, H2-DMA, TGFB1 |
| Fanconi ar | 6 | 2.684936 | 0.070513 | 61.13496 | FAN1, FANCF, ATR, ATRIP, FANCB, FANCC |
| Graft-versu | 6 | 2.633303 | 0.075365 | 63.67651 | H2-K1, H2-Q2, H2-D1, H2-AA, H2-DMA, H2-Q6 |
| PI3K-Akt | 22 | 1.430436 | 0.087841 | 69.52577 | COL4A4, COL4A3, ITGA1, COL5A2, IRS1, VEGFB, GNG8, PRLR, COL6A5, LAMA5, COL27A1, ITGA8, COL6A3, MTCP1, NGFR, COL1A1, COL11A2, GM5741, COL11A1, CHUK, THBS3, SPP1 |
| Wnt signal | 11 | 1.780436 | 0.088702 | 69.89546 | SFRP5, WNT10A, WNT1, NKD1, SFRP1, WNT3A, WIF1, WNT6, TCF7L2, TCF7L1, WNT8B |
| Allograft r | 6 | 2.44521 | 0.096515 | 73.0659 | H2-K1, H2-Q2, H2-D1, H2-AA, H2-DMA, H2-Q6 |
| Acute mye | 6 | 2.44521 | 0.096515 | 73.0659 | JUP, FLT3, ZBTB16, TCF7L2, TCF7L1, CHUK |

**Dataset S5. KEGG enrichment analysis for DE genes between SI and control (SI/C) female offspring.**

| Term | Count | Fold Enric | PValue | FDR | Genes |
|---|---|---|---|---|---|
| Ribosome | 30 | 3.025174 | 1.09E-07 | 1.43E-04 | MRPS15, RPL13, RPL35, RPLP2, RPL37, RPL38, RPS2, RPS28, RPS29, RPL31, RPLP1, RPS21, MRPL34, RPSA, RPL35A, RPL27, MRPS21, RPS5, RPS8, RPL28, MRPL23, RPS18, RPL41, RPL18A, RPS16, RPL13A, RPS15, RPS12, RPL37A, RPS10 |
| Cell adhesi | 29 | 2.61746 | 3.95E-06 | 0.005169 | CLDN9, OCLN, CLDN3, H2-D1, CLDN10, CDH3, ITGAM, CD4, SELPLG, SPN, H2-K1, F11R, ICAM1, PTPRC, CD8B1, H2-M3, ITGA4, H2-Q7, H2-DMB2, NCAM2, SIGLEC1, ITGA8, CD274, H2-EB1, PECAM1, CLDN1, CLDN2, MADCAM1, H2-T24 |
| Staphylocc | 12 | 3.509202 | 4.38E-04 | 0.572408 | C3AR1, ICAM1, C5AR1, C3, H2-EB1, FCGR4, CFH, FPR2, C1S1, SELPLG, ITGAM, H2-DMB2 |
| Non-alcoh | 23 | 2.142029 | 9.39E-04 | 1.223526 | PPARA, NDUFB5, NDUFA3, SOCS3, LEPR, NDUFB9, NDUFA6, COX8A, COX7C, COX4I1, COX5A, UQCRFS1, COX5B, UQCRQ, IRS1, NDUFA12, IL6RA, NDUFA11, NDUFS6, COX6A2, ERN1, COX6A1, FAS |
| Cytokine-c | 30 | 1.812604 | 0.002031 | 2.629103 | CXCL1, IL1R2, IL1R1, CXCL5, OSMR, CSF2RB2, LEPR, CCR1, IL4RA, CXCL9, CCL8, PF4, TNFSF12, CCL28, CSF2RB, FAS, IFNLR1, IL18RAP, GM20878, CCL21A, IL6RA, INHBA, ACVR2B, TNFSF10, CXCL14, PRLR, CXCL13, CXCL16, CCR2, NGFR |
| ECM-recep | 15 | 2.492331 | 0.002363 | 3.052169 | COL4A4, COL4A3, LAMA5, GP1BB, COL27A1, ITGA8, COL6A3, ITGA1, COL6A1, ITGA4, THBS1, COL11A1, COL5A2, THBS3, THBS4 |
| Oxidative p | 20 | 2.103838 | 0.002733 | 3.521634 | ATP5D, ATP5J2, NDUFB5, NDUFA3, NDUFA6, NDUFB9, COX8A, COX7C, COX4I1, COX5A, UQCRFS1, COX5B, UQCRQ, NDUFA12, NDUFA11, NDUFS6, COX6A2, COX6A1, ATP5L, COX17 |
| Alzheimer' | 23 | 1.899991 | 0.004379 | 5.588012 | ATP5D, NDUFB5, NDUFA3, NDUFA6, NDUFB9, COX8A, COX7C, COX4I1, ITPR3, COX5A, UQCRFS1, COX5B, UQCRQ, NDUFA12, NDUFA11, NDUFS6, APOE, RYR3, COX6A2, ERN1, COX6A1, ADAM17, FAS |
| Parkinson's | 20 | 1.962641 | 0.005931 | 7.497886 | ATP5D, NDUFB5, NDUFA3, NDUFA6, NDUFB9, COX8A, UCHL1, COX7C, UBA7, COX4I1, COX5A, UQCRFS1, COX5B, UQCRQ, NDUFA12, NDUFA11, NDUFS6, COX6A2, COX6A1, LRRK2 |
| Chemokine | 24 | 1.790409 | 0.007322 | 9.179673 | CXCL1, ITK, CXCL5, CCR1, GM20878, ADCY6, GNG13, CCL21A, CXCL9, CCL8, RAF1, PF4, GNG11, CCL28, GNG8, CXCL14, ADCY9, CXCL13, CXCL16, CCR2, SHC3, GM5741, GNG5, CHUK |
| Herpes sim | 25 | 1.757413 | 0.007659 | 9.58306 | H2-K1, IFIH1, SP100, H2-M3, SOCS3, C3, TLR2, H2-D1, H2-Q7, CD74, H2-DMB2, CFP, DDX58, FOS, IKBKE, TAF10, IFIT1, IRF7, H2-EB1, H2-T24, FAS, OAS1A, EIF2AK2, OAS1G, CHUK |
| Hematopoi | 13 | 2.236256 | 0.012235 | 14.89642 | IL1R2, IL1R1, CD3G, CD8B1, GP1BB, H2-EB1, IL4RA, ITGA1, CD4, ITGA4, ITGAM, CD14, IL6RA |
| Focal adhe | 24 | 1.695266 | 0.013727 | 16.56483 | COL4A4, COL4A3, FLT1, MYLK4, ITGA1, MYLK2, RAF1, ITGA4, HGF, BIRC3, FLNB, COL5A2, VCL, VEGFB, LAMA5, COL27A1, ITGA8, COL6A3, COL6A1, THBS1, SHC3, COL11A1, THBS3, THBS4 |
| Cardiac mu | 12 | 2.278702 | 0.014803 | 17.74925 | FXYD2, COX8A, COX6A2, COX7C, COX4I1, CACNG4, COX6A1, MYH7, COX5A, UQCRFS1, COX5B, UQCRQ |
| Salivary se | 12 | 2.278702 | 0.014803 | 17.74925 | KCNMA1, FXYD2, ATP2B3, ADCY9, SLC12A2, RYR3, ADCY6, CST3, SLC4A2, ITPR3, PRKG1, DMBT1 |
| Circadian e | 14 | 2.08881 | 0.015358 | 18.35517 | NOS1AP, ADCY6, GNG13, GNG11, ITPR3, PRKG1, GNG8, FOS, KCNJ6, ADCY9, RYR3, RYR1, GNG5, GM5741 |
| Protein dig | 13 | 2.16002 | 0.015879 | 18.91859 | COL4A4, COL4A3, FXYD2, SLC8A1, COL9A3, COL14A1, COL27A1, COL6A3, COL12A1, COL6A1, COL11A1, COL5A2, KCNJ13 |
| HTLV-I in | 29 | 1.536335 | 0.022035 | 25.31846 | IL1R2, IL1R1, NRP1, ADCY6, H2-D1, ANAPC11, WNT2, FOS, POLE4, MAP3K3, CHUK, TERT, FZD9, H2-K1, WNT10A, ICAM1, CD3G, EGR2, H2-M3, FZD3, ATR, H2-Q7, FZD4, H2-DMB2, ATM, ADCY9, H2-EB1, H2-T24, MAP3K14 |
| Hepatitis C | 17 | 1.827709 | 0.022502 | 25.78455 | PPARA, OCLN, CLDN9, CLDN3, SOCS3, RAF1, CLDN10, DDX58, IKBKE, IFIT1, IRF7, CLDN1, CLDN2, OAS1A, EIF2AK2, OAS1G, CHUK |
| Graft-versu | 9 | 2.530674 | 0.023489 | 26.7602 | H2-K1, H2-M3, H2-EB1, H2-D1, GZMB, FAS, H2-T24, H2-Q7, H2-DMB2 |
| Huntingtor | 22 | 1.62463 | 0.028609 | 31.63508 | ATP5D, DNAH11, DNAH9, NDUFB5, POLR2F, NDUFA3, NDUFA6, NDUFB9, POLR2I, COX8A, COX7C, COX4I1, COX5A, UQCRFS1, COX5B, UQCRQ, NDUFA12, NDUFA11, DNAH6, NDUFS6, COX6A2, COX6A1 |
| Allograft r | 9 | 2.349912 | 0.035007 | 37.30545 | H2-K1, H2-M3, H2-EB1, H2-D1, GZMB, FAS, H2-T24, H2-Q7, H2-DMB2 |
| PI3K-Akt s | 34 | 1.416344 | 0.037033 | 39.00846 | OSMR, IL4RA, TLR2, GNG13, GNG11, GNG8, COL27A1, COL6A3, COL6A1, MTCP1, THBS1, GM5741, GNG5, COL11A1, CHUK, THBS3, THBS4, COL4A4, COL4A3, FLT1, SGK3, ITGA1, RAF1, ITGA4, HGF, COL5A2, IRS1, IL6RA, VEGFB, PRLR, LAMA5, ITGA8, NGFR, PPP2R3D |
| Cholinergi | 14 | 1.811535 | 0.043596 | 44.23551 | GNG8, FOS, CHRM5, KCNJ6, ADCY9, ADCY6, GNG13, GNG11, CHRNA7, SLC5A7, ITPR3, GM5741, GNG5, CHRNA3 |
| Mineral ab | 7 | 2.624403 | 0.046908 | 46.71339 | FXYD2, TRPM7, SLC39A4, STEAP2, STEAP1, SLC31A1, FTH1 |
| Apoptosis | 9 | 2.193251 | 0.049846 | 48.82613 | TNFSF10, CSF2RB2, ENDOG, CSF2RB, FAS, MAP3K14, BIRC3, CHUK, ATM |
| Morphine a | 12 | 1.886667 | 0.051101 | 49.70409 | GNG8, KCNJ6, ADCY9, GABRB2, GABRA6, ADCY6, GNG13, PDE11A, GNG11, PDE4D, GM5741, GNG5 |
| Bile secret | 10 | 2.059391 | 0.051162 | 49.74662 | FXYD2, SLCO1A4, ADCY9, ABCB1A, AQP9, ADCY6, SLC4A2, AQP1, SLCO1A5, SLC4A5 |
| Antigen pr | 11 | 1.961444 | 0.051474 | 49.96276 | H2-K1, CIITA, CD8B1, H2-M3, H2-EB1, H2-D1, CD4, H2-T24, H2-Q7, CD74, H2-DMB2 |
| Type I diab | 9 | 2.122501 | 0.058593 | 54.66594 | H2-K1, H2-M3, H2-EB1, H2-D1, GZMB, FAS, H2-T24, H2-Q7, H2-DMB2 |
| GABAergi | 11 | 1.848717 | 0.07143 | 62.12977 | GNG8, KCNJ6, ADCY9, GABRB2, GABRA6, ADCY6, GNG13, GNG11, GM5741, GNG5, GABARAP |
| Compleme | 10 | 1.923904 | 0.072835 | 62.87359 | C3AR1, A2M, F10, C5AR1, SERPINA1B, F5, C3, CD46, CFH, C1S1 |
| Pyrimidine | 12 | 1.754601 | 0.077938 | 65.4636 | TYMS, POLR2F, POLE4, UPRT, PNP2, UPB1, NT5C1A, POLR2I, POLR1A, CTPS2, DCTPP1, AK9 |
| Primary im | 6 | 2.580295 | 0.07864 | 65.80633 | DCLRE1C, CIITA, PTPRC, CD8B1, AIRE, CD4 |
| Measles | 15 | 1.612685 | 0.079753 | 66.34364 | IFIH1, CD3G, TLR2, CD209B, TLR7, DDX58, IKBKE, TNFSF10, CD46, IRF7, FAS, OAS1A, EIF2AK2, OAS1G, CHUK |
| p53 signali | 9 | 1.964105 | 0.084472 | 68.53638 | DDB2, PMAIP1, SFN, FAS, ATR, MDM4, THBS1, PERP, ATM |
| Tight junct | 11 | 1.787093 | 0.085463 | 68.97952 | F11R, CLDN9, OCLN, MYH15, CLDN3, CGN, CLDN1, CLDN2, CRB3, CLDN10, TJP3 |
| Vascular sr | 14 | 1.611838 | 0.092096 | 71.80127 | KCNMA1, ADCY9, ADCY6, MYLK4, RAF1, MYLK2, ITPR3, PRKG1, PLA2G3, PPP1R14A, KCNMB1, PLA2G5, PLA2G2F, ARHGEF11 |

**Dataset S6. KEGG enrichment analysis for DE genes between RS and SI (RS/SI) female offspring.**

| Term | Count | Fold Enrich | PValue | FDR | Genes |
|---|---|---|---|---|---|
| Staphylococ | 16 | 15.098896 | 4.42E-14 | 5.56E-11 | ITGAL, ICAM1, C3AR1, C5AR1, C3, C4B, FCGR4, ITGB2, H2-AB1, C1QC, FCGR3, FCGR2B, H2-OB, H2-EB1, H2-AA, CFI |
| Cell adhesio | 19 | 5.5339317 | 6.58E-09 | 8.28E-06 | ITGAL, PTPRC, ICAM1, CD8B1, H2-D1, ITGB2, CDH1, H2-AB1, H2-Q6, H2-Q7, CDH5, H2-OB, H2-EB1, H2-AA, H2-T23, CD4, MADCAM1, H2-T24, SPN |
| Type I diabe | 12 | 9.1323966 | 5.18E-08 | 6.52E-05 | PTPRN2, H2-EB1, H2-OB, H2-D1, H2-AA, H2-T23, GZMB, H2-AB1, FAS, H2-T24, H2-Q6, H2-Q7 |
| Graft-versus | 11 | 9.9812412 | 9.62E-08 | 1.21E-04 | H2-EB1, H2-OB, H2-D1, H2-AA, H2-T23, GZMB, H2-AB1, FAS, H2-T24, H2-Q6, H2-Q7 |
| Antigen pro | 13 | 7.480398 | 1.17E-07 | 1.48E-04 | CD8B1, H2-D1, H2-AB1, H2-Q6, H2-Q7, CD74, TAP1, H2-OB, H2-EB1, H2-AA, CD4, H2-T23, H2-T24 |
| Allograft re | 11 | 9.2682954 | 2.01E-07 | 2.53E-04 | H2-EB1, H2-OB, H2-D1, H2-AA, H2-T23, GZMB, H2-AB1, FAS, H2-T24, H2-Q6, H2-Q7 |
| Complemen | 12 | 7.450113 | 4.53E-07 | 5.70E-04 | VWF, C3AR1, A2M, THBD, F10, C5AR1, F5, C4B, C3, CFI, C1QC, PLAUR |
| Phagosome | 17 | 4.6908119 | 5.28E-07 | 6.64E-04 | MSR1, C3, H2-D1, FCGR4, ITGB2, H2-AB1, H2-Q6, H2-Q7, FCGR3, FCGR2B, TAP1, H2-OB, H2-EB1, H2-AA, H2-T23, H2-T24, CD14 |
| Viral myoca | 12 | 7.1671973 | 6.77E-07 | 8.52E-04 | ITGAL, ICAM1, H2-EB1, H2-OB, H2-D1, H2-AA, ITGB2, H2-T23, H2-AB1, H2-T24, H2-Q6, H2-Q7 |
| Autoimmun | 11 | 7.3102048 | 1.99E-06 | 0.0025045 | H2-EB1, H2-OB, H2-D1, H2-AA, H2-T23, GZMB, H2-AB1, FAS, H2-T24, H2-Q6, H2-Q7 |
| HTLV-I inf | 20 | 3.419134 | 4.83E-06 | 0.0060834 | ZFP36, ITGAL, IL1R2, ICAM1, WNT5B, EGR2, H2-D1, ITGB2, H2-AB1, H2-Q6, H2-Q7, CDKN1A, FZD10, ADCY9, H2-OB, H2-EB1, SLC2A1, H2-AA, H2-T23, H2-T24 |
| Herpes simp | 16 | 3.6295422 | 2.98E-05 | 0.0375034 | SOCS3, C3, H2-D1, H2-AB1, CCL5, H2-Q6, H2-Q7, CD74, CCL12, TAP1, H2-OB, H2-EB1, H2-AA, H2-T23, FAS, H2-T24 |
| Rheumatoid | 10 | 5.7541523 | 5.14E-05 | 0.0646378 | ITGAL, CCL12, ICAM1, FLT1, H2-EB1, H2-OB, H2-AA, ITGB2, H2-AB1, CCL5 |
| Cytokine-cy | 16 | 3.1196066 | 1.67E-04 | 0.2103823 | CXCL1, IL1R2, OSMR, CCR1, IL4RA, CXCL9, CCL5, INHBA, CCL12, TNFRSF11B, CXCL13, CXCL16, CXCR6, CCR2, CSF2RB, FAS |
| Tuberculosi | 13 | 3.4851854 | 3.23E-04 | 0.4054144 | C3, SPHK1, FCGR4, ITGB2, H2-AB1, CD74, FCGR3, FCGR2B, CLEC4E, H2-OB, H2-EB1, H2-AA, CD14 |
| Leishmania | 8 | 5.8980061 | 3.66E-04 | 0.4597678 | C3, H2-EB1, H2-OB, FCGR4, H2-AA, ITGB2, H2-AB1, FCGR3 |
| Hematopoie | 8 | 4.4408517 | 0.0020119 | 2.5025623 | IL1R2, CD8B1, H2-EB1, IL4RA, MS4A1, ANPEP, CD4, CD14 |
| Influenza A | 11 | 3.0352312 | 0.0032027 | 3.9565247 | CCL12, ICAM1, SOCS3, H2-EB1, H2-OB, H2-AA, H2-AB1, FAS, CCL5, CASP1, TLR7 |
| Malaria | 6 | 5.8980061 | 0.0032131 | 3.9691102 | ITGAL, CCL12, ICAM1, ITGB2, HBB-BS, HBB-BT |
| Osteoclast d | 9 | 3.3702892 | 0.0050008 | 6.1141709 | PIRB, TNFRSF11B, CYBB, FCGR2B, SOCS3, GM9733, FCGR4, FOSB, FCGR3 |
| Natural kill | 8 | 3.5949752 | 0.0065425 | 7.9285865 | ITGAL, ICAM1, CD244, KLRK1, FCGR4, ITGB2, GZMB, FAS |
| Epstein-Bar | 9 | 3.1224738 | 0.0078573 | 9.4503917 | ITGAL, ICAM1, CDKN1A, H2-D1, H2-T23, H2-T24, H2-Q6, H2-Q7, SPN |
| Intestinal in | 5 | 5.6171487 | 0.0113965 | 13.432361 | H2-EB1, H2-OB, H2-AA, MADCAM1, H2-AB1 |
| Systemic lu | 9 | 2.8888193 | 0.0122608 | 14.379982 | HIST1H2BE, C4B, C3, H2-EB1, H2-OB, FCGR4, H2-AA, H2-AB1, C1QC |
| Asthma | 4 | 7.8640082 | 0.0134189 | 15.634816 | H2-EB1, H2-OB, H2-AA, H2-AB1 |
| Pertussis | 6 | 3.8257337 | 0.0195582 | 22.008629 | C4B, C3, ITGB2, CASP1, C1QC, CD14 |
| Chemokine | 10 | 2.4073494 | 0.0224575 | 24.861781 | CXCL1, CCL12, ADCY9, CXCL13, CCR1, CXCL16, CXCR6, CCR2, CXCL9, CCL5 |
| Viral carcin | 11 | 2.2468595 | 0.0238497 | 26.19746 | CDKN1A, EGR3, EGR2, HIST1H2BE, C3, H2-D1, H2-T23, PMAIP1, H2-T24, H2-Q6, H2-Q7 |
| Legionellos | 5 | 4.1389517 | 0.0315958 | 33.23814 | CXCL1, C3, ITGB2, CASP1, CD14 |
| Primary imi | 4 | 5.5510646 | 0.0340682 | 35.351732 | PTPRC, CD8B1, TAP1, CD4 |
| Inflammato | 5 | 3.9986482 | 0.0352589 | 36.347523 | H2-EB1, H2-OB, IL4RA, H2-AA, H2-AB1 |
| African tryp | 4 | 5.3924628 | 0.0367022 | 37.535568 | ICAM1, HBB-BS, FAS, HBB-BT |
| Vascular sm | 7 | 2.6006956 | 0.051303 | 48.458348 | ADCY9, ADORA2A, MYLK2, ITPR3, PLA2G3, PPP1R14A, PLA2G2F |
| Bile secretio | 5 | 3.3228204 | 0.062202 | 55.433787 | ADCY9, ABCB1A, SLC2A1, SLC22A8, ABCC3 |
| Toxoplasmo | 6 | 2.6962314 | 0.0703405 | 60.063682 | LAMA1, IGTP, H2-EB1, H2-OB, H2-AA, H2-AB1 |
| TNF signali | 6 | 2.5972871 | 0.0797324 | 64.85427 | CXCL1, CCL12, ICAM1, SOCS3, FAS, CCL5 |

**Dataset S7. GO (biological process) enrichment analysis for DE genes  between RS and SI (RS/SI) female offspring.**

| Term | Count | Fold Enrich | PValue | FDR | Genes |
|---|---|---|---|---|---|
| immune sy | 41 | 5.4834873 | 2.92E-18 | 5.00E-15 | TNFAIP8L2, APOBEC3, CD244, IFITM1, S100A8, C3, IL4RA, S100A9, KLRK1, PGLYRP1, H2-D1, HP, LY9, GBP2B, TLR7, C1QC, CD74, RNF125, NLRC5, CASP4, CLEC4E, OASL2, TAP1, CD4, CFI, ZBP1, CD8B1, TLR13, LILRB4A, EOMES, ANXA1, H2-AB1, H2-Q7, PSMB9, LCN2, PIRB, H2-EB1, CD300LF, H2-AA, H2-T23, CD14 |
| inflammato | 30 | 4.4671915 | 3.75E-11 | 6.40E-08 | CXCL1, C3AR1, S100A8, C3, AIF1, CCR1, S100A9, CXCL9, CCL5, TLR7, ZFP580, REG3B, TNFRSF11B, CASP4, CHIL3, CXCR6, FAS, SPP1, C5AR1, C4B, SPHK1, TLR13, ANXA1, PTGFR, CD163, CCL12, CYBB, CXCL13, CCR2, CD14 |
| immune res | 26 | 4.8963923 | 1.42E-10 | 2.42E-07 | CXCL1, CD244, CCR1, CXCL9, H2-D1, IFI44L, CCL5, TLR7, CD74, TNFRSF11B, CLEC4E, OASL2, CD4, FAS, FYB, PRG4, H2-AB1, CCL12, FCGR2B, CXCL13, CCR2, H2-OB, H2-EB1, H2-AA, TGTP1, TGTP2 |
| response to | 9 | 17.731314 | 2.19E-08 | 3.74E-05 | IRGM2, IFITM1, CXCL16, H2-EB1, H2-AA, TGTP1, TGTP2, CCL5, GCH1 |
| chemotaxis | 15 | 6.5114995 | 7.28E-08 | 1.24E-04 | C3AR1, C5AR1, FLT1, S100A8, CCR1, S100A9, CXCL9, CCL5, ZFP580, CCL12, CXCL13, CXCL16, CXCR6, CKLF, CCR2 |
| positive reg | 14 | 5.9267202 | 6.84E-07 | 0.0011682 | GATA2, C3AR1, CYBB, FLT1, C5AR1, ACVRL1, CYP1B1, ADM, C3, HIPK2, SPHK1, ITGB2, CCL5, CTSH |
| innate imm | 25 | 3.2014873 | 1.16E-06 | 0.001986 | TNFAIP8L2, APOBEC3, CD244, IFITM1, S100A8, C3, C4B, S100A9, TLR13, KLRK1, ANXA1, PGLYRP1, JCHAIN, LY9, C1QC, TLR7, LCN2, NLRC5, CYBB, CASP4, CLEC4E, OASL2, CFI, CD14, ZBP1 |
| adaptive im | 14 | 5.1592313 | 3.31E-06 | 0.0056588 | CD244, CD8B1, LILRB4A, KLRK1, EOMES, JCHAIN, ANXA1, LY9, CD74, RNF125, PIRB, TAP1, CD4, H2-T23 |
| neutrophil c | 10 | 7.4237386 | 7.35E-06 | 0.0125484 | CXCL1, CCL12, C5AR1, S100A8, S100A9, CKLF, ITGB2, CCL5, FCGR3, SPP1 |
| cell adhesio | 26 | 2.7460179 | 1.03E-05 | 0.0175502 | ITGAL, CYP1B1, MPZL2, PCDHGC5, CDH1, ITGB2, LY9, PCDHGA4, CDH5, COL12A1, CD4, FN1, SPP1, ICAM1, CNTN5, SDK1, STAB2, VWF, LAMA1, OMD, PGM5, PKP3, TENM3, DSC3, MADCAM1, ITGAD |
| defense res | 11 | 6.0587286 | 1.33E-05 | 0.0226559 | REG3B, LYZ2, GBP6, C5AR1, ADM, HIST1H2BE, PGLYRP1, KLRK1, MYO1F, H2-T23, GBP2B |
| homophilic | 14 | 4.3995898 | 1.89E-05 | 0.0322221 | PCDHGA9, SDK1, PCDHGC5, PCDHGA7, CDH1, PCDHGA6, PCDHGA5, PCDHGA4, PCDHGA3, CDH5, PCDHGA2, PCDHGA1, TENM3, DSC3 |
| positive reg | 15 | 3.7850096 | 4.63E-05 | 0.0790104 | C3AR1, WNT5B, FLT1, AIF1, CCR1, SPHK1, SUN2, MYO1F, CCL5, MIEN1, SEMA3G, CXCL16, CTSH, MYOC, FN1 |
| chemokine- | 8 | 7.450734 | 9.07E-05 | 0.1548802 | CXCL1, CCL12, GPR35, CXCL13, CCR1, CCR2, CXCL9, CCL5 |
| antigen pro | 5 | 18.294213 | 1.21E-04 | 0.2066863 | FCGR2B, H2-EB1, H2-AA, H2-AB1, CD74 |
| cellular res | 7 | 8.7455262 | 1.32E-04 | 0.2251319 | GBP6, IRGM2, IGTP, TGTP1, TGTP2, GBP2B, IFI204 |
| positive reg | 12 | 4.1532808 | 1.55E-04 | 0.2639632 | CXCL1, GATA2, C3AR1, GPR35, C5AR1, ADM, CXCL13, CCR1, AGT, ITPR3, PTGFR, HTR2A |
| positive reg | 7 | 7.968146 | 2.24E-04 | 0.3817223 | ICAM1, AIF1, AGT, KLRK1, ITGB2, KLF2, KLF4 |
| positive reg | 8 | 6.4029745 | 2.39E-04 | 0.4077455 | ITGAL, PTPRC, AIF1, ANXA1, CD4, SLC4A1, CCL5, SPN |
| response to | 9 | 5.3606298 | 2.70E-04 | 0.4600301 | DDC, CDKN1A, HTR1B, CYP1B1, SLC22A8, CDH1, FAS, CCL5, GJC2 |
| antigen pro | 4 | 22.766132 | 5.63E-04 | 0.9577778 | H2-EB1, H2-OB, H2-AA, H2-AB1 |
| antigen pro | 7 | 6.6401217 | 6.13E-04 | 1.0418896 | RAB32, H2-EB1, H2-D1, H2-AA, H2-AB1, CD74, PSMB9 |
| antigen pro | 6 | 8.5372993 | 6.24E-04 | 1.0602738 | H2-Q4, H2-D1, H2-T23, H2-T24, H2-Q6, H2-Q7 |
| leukocyte c | 5 | 10.671624 | 0.0011022 | 1.8665352 | ITGAL, ICAM1, PTPRC, ITGB2, CCL5 |
| single orga | 9 | 4.2686497 | 0.0012381 | 2.0943329 | ITGAL, ICAM1, MPZL2, TNXB, PKP3, ITGB2, CDH1, KIRREL2, CDH5 |
| cell surface | 13 | 3.040682 | 0.0012694 | 2.1467342 | ITGAL, LAMA1, FZD10, FCGR2B, OSMR, CXCL13, AGT, ADGRF5, ANXA1, CD4, GPR88, SPN, FCGR3 |
| regulation c | 7 | 5.7833318 | 0.0012808 | 2.1658095 | LNPEP, C3AR1, ACVRL1, CYP4F18, AGT, SULT1A1, GCH1 |
| response to | 5 | 10.244759 | 0.0012922 | 2.1849428 | ENDOG, CXCL16, CCL5, CD14, GCH1 |
| negative re | 18 | 2.4011154 | 0.0014849 | 2.5068269 | CYP1B1, ACVRL1, IFITM1, ADORA2A, ZBTB16, CDH5, SLFN1, SLFN2, SSTR4, INHBA, TNS2, CDKN1A, ADM, DLL4, AGT, NPPC, ERDR1, KLF4 |
| defense res | 11 | 3.3740225 | 0.0016363 | 2.7590357 | APOBEC3, PTPRC, NLRC5, IFITM1, OASL2, CXCL9, SLFN8, PMAIP1, GBP2B, TLR7, ZBP1 |
| response to | 12 | 3.1202312 | 0.0017153 | 2.8904248 | CXCL1, TNFRSF11B, THBD, C5AR1, S100A8, ADM, CXCL13, CXCL9, FAS, CASP1, PTGFR, GCH1 |
| positive reg | 4 | 15.761168 | 0.0018095 | 3.0468779 | CYBB, CCR2, SPN, FCGR3 |
| positive reg | 4 | 15.761168 | 0.0018095 | 3.0468779 | CCK, TRABD2B, JCHAIN, PMAIP1 |
| defense res | 9 | 3.97426 | 0.0019549 | 3.2879357 | IRGM2, IGTP, FCGR2B, NGP, TAP1, H2-D1, TGTP1, TGTP2, CD74 |
| cellular res | 7 | 5.2730378 | 0.002069 | 3.4765926 | CCL12, GBP6, AIF1, H2-AB1, CCL5, GBP2B, H2-Q7 |
| positive reg | 4 | 14.63537 | 0.0022699 | 3.8081639 | AIF1, CCR1, CCR2, CCL5 |
| negative thy | 4 | 14.63537 | 0.0022699 | 3.8081639 | PTPRC, FAS, CD74, SPN |
| angiogenes | 13 | 2.7862316 | 0.0026259 | 4.3928749 | CYP1B1, FLT1, ACVRL1, EFNA1, APOLD1, ANPEP, VASH1, MMRN2, CCL12, DLL4, CCR2, HIF3A, FN1 |
| positive reg | 3 | 30.734278 | 0.0036348 | 6.0318189 | CXCL13, CCL5, PIEZO1 |
| positive reg | 3 | 30.734278 | 0.0036348 | 6.0318189 | CXCL13, FOXC2, PIEZO1 |
| acute-phase | 5 | 7.3176851 | 0.0045914 | 7.5612421 | REG3B, SAA3, HP, FN1, CD163 |
| cellular res | 7 | 4.4820822 | 0.004679 | 7.7001448 | LCN2, CCL12, ICAM1, SAA3, CCL5, KLF2, FN1 |
| cellular res | 6 | 5.3919785 | 0.0049687 | 8.1580194 | LCN2, CYP1B1, ANXA1, KLF2, ZFP580, KLF4 |
| heterotypic | 4 | 10.783957 | 0.0056229 | 9.1843448 | PTPRC, MADCAM1, ITGB2, ITGAD |
| response to | 7 | 4.2686497 | 0.0059342 | 9.6688751 | LCN2, IFITM1, OASL2, TLR13, TGTP1, TGTP2, CCL5 |
| blood coag | 7 | 4.2686497 | 0.0059342 | 9.6688751 | P2RY12, VWF, MAFF, THBD, F10, F5, C3 |
| regulation c | 6 | 5.0384062 | 0.0066292 | 10.741825 | CASP4, S100A8, AGT, S100A9, ANXA1, CASP1 |
| collagen fib | 5 | 6.5671533 | 0.0067872 | 10.984016 | FMOD, TNXB, CYP1B1, ADAMTS14, FOXC2 |
| response to | 15 | 2.2665396 | 0.0069135 | 11.177272 | ICAM1, ADORA2A, ANXA1, CDH1, FOSB, LCN2, INHBA, HTR1B, CDKN1A, CYBB, TNFRSF11B, APOD, NPPC, CASP1, HTR2A |
| negative re | 7 | 4.1214549 | 0.007025 | 11.347364 | ZFP36, TNFAIP8L2, IER3, ADORA2A, SOCS3, PGLYRP1, CTLA2A |
| negative re | 5 | 6.4029745 | 0.0074278 | 11.959884 | H2-AA, H2-AB1, SLFN1, SPN, PLA2G2F |
| response to | 4 | 9.7569135 | 0.0074994 | 12.068257 | CDKN1A, THBD, ANXA1, PMAIP1 |
| apoptotic p | 21 | 1.8871925 | 0.0082687 | 13.225542 | CKAP2, C5AR1, CCK, S100A8, GZMA, S100A9, GPR65, PGLYRP1, GZMB, PMAIP1, MIEN1, LCN2, TNFRSF11B, HIPK2, THOC6, HIF3A, FAS, XAF1, CASP1, HCAR2, SRGN |
| immunoglo | 4 | 9.3134175 | 0.00856 | 13.659883 | FCGR2B, C4B, IL4RA, CD74 |
| antigen pro | 3 | 19.208924 | 0.009791 | 15.473317 | ABCB1A, TAP1, H2-T23 |
| activation c | 6 | 4.5872056 | 0.0097953 | 15.479511 | XDH, CCK, S100A8, ACER2, S100A9, PMAIP1 |
| positive reg | 4 | 8.5372993 | 0.0109329 | 17.123285 | C3AR1, C5AR1, CYP1B1, C3 |
| hemostasis | 5 | 5.5678039 | 0.0121122 | 18.79551 | P2RY12, VWF, THBD, F10, F5 |
| cytolysis | 4 | 8.1958074 | 0.0122475 | 18.985326 | LYZ2, GZMA, GZMB, FGL2 |
| antigen pro | 3 | 17.074599 | 0.0124275 | 19.23722 | ABCB1A, TAP1, H2-D1 |
| cellular res | 3 | 17.074599 | 0.0124275 | 19.23722 | ICAM1, DDC, HTR1B |
| activated T | 3 | 17.074599 | 0.0124275 | 19.23722 | ITGAL, ITGB2, ITGAD |
| adhesion of | 3 | 17.074599 | 0.0124275 | 19.23722 | ICAM1, GBP6, GBP2B |
| positive reg | 7 | 3.5501641 | 0.014087 | 21.524732 | ICAM1, CCK, EFNA1, AGT, CD4, CD74, HTR2A |
| endodermal | 4 | 7.5887105 | 0.0151376 | 22.941292 | INHBA, MMP8, COL12A1, FN1 |
| drug transp | 3 | 15.367139 | 0.0153362 | 23.206315 | ABCB1A, ABCC3, SLC47A1 |
| protein hon | 10 | 2.6001927 | 0.0153694 | 23.250542 | REG3B, VWF, KCNA1, KCNA3, SLC22A6, CDH1, KCNA5, FAS, ITPR3, GCH1 |
| response to | 6 | 4.0979037 | 0.0154487 | 23.35604 | PTGDS, ADM, AIF1, SULT1A1, IL1RN, FAS |
| protein kina | 4 | 7.3176851 | 0.0167144 | 25.022188 | HTR1B, CCK, ADORA2A, DGKH |
| positive reg | 4 | 7.3176851 | 0.0167144 | 25.022188 | CXCL1, C3AR1, C5AR1, CD74 |
| cell-matrix | 6 | 3.940292 | 0.0180337 | 26.722455 | ITGAL, TNXB, AGT, MADCAM1, ITGB2, FN1 |
| cellular res | 4 | 6.8298395 | 0.0201337 | 29.354291 | ZFP36, EGR3, DLL4, CCL5 |
| antigen pro | 4 | 6.8298395 | 0.0201337 | 29.354291 | H2-D1, H2-T23, H2-Q7, PSMB9 |
| defense res | 4 | 6.8298395 | 0.0201337 | 29.354291 | GBP6, IRGM2, IL4RA, GBP2B |
| response to | 3 | 12.805949 | 0.0219241 | 31.527559 | IFITM1, TGTP1, TGTP2 |
| negative re | 3 | 12.805949 | 0.0219241 | 31.527559 | IL1R2, CDH1, CTLA2A |
| negative re | 3 | 12.805949 | 0.0219241 | 31.527559 | DLL4, KLF4, MMRN2 |
| intrinsic ap | 4 | 6.6095221 | 0.0219767 | 31.590423 | CDKN1A, HIPK2, PMAIP1, IFI204 |
| skeletal mu | 5 | 4.6567087 | 0.0221086 | 31.747901 | MAFF, EGR2, EOMES, MYLK2, SMYD1 |
| extracellula | 7 | 3.1453208 | 0.0240986 | 34.082452 | LAMA1, FBLN1, TNFRSF11B, TNXB, CRISPLD2, AGT, FN1 |
| negative re | 3 | 11.820876 | 0.0255813 | 35.772729 | ACVRL1, DLL4, VASH1 |
| mast cell ac | 3 | 11.820876 | 0.0255813 | 35.772729 | FYB, FCGR2B, FCGR3 |
| leukocyte n | 3 | 11.820876 | 0.0255813 | 35.772729 | S100A8, S100A9, ITGB2 |
| antibacteria | 4 | 6.208945 | 0.0259296 | 36.163864 | ADM, HIST1H2BE, JCHAIN, H2-T23 |
| lymphocyte | 4 | 6.208945 | 0.0259296 | 36.163864 | CCL12, CXCL16, CKLF, CCL5 |
| regulation c | 5 | 4.3409997 | 0.0277882 | 38.213193 | INHBA, TLR13, LEFTY2, MYLK2, MYOC |
| positive reg | 4 | 6.0263289 | 0.0280394 | 38.485368 | CLEC4E, AGT, CASP1, CD14 |
| phospholipa | 5 | 4.2686497 | 0.0293315 | 39.867659 | C3AR1, GPR84, HTR1B, C5AR1, HTR2A |
| macrophage | 3 | 10.976528 | 0.0294665 | 40.01046 | CCL12, GPR35, CKLF |
| drug transm | 3 | 10.976528 | 0.0294665 | 40.01046 | ABCB1A, ABCC3, SLC47A1 |
| response to | 3 | 10.976528 | 0.0294665 | 40.01046 | CDKN1A, TNFRSF11B, SLC4A1 |
| response to | 4 | 5.8541481 | 0.0302376 | 40.819571 | FCGR2B, BAIAP2L1, CASP1, CD14 |
| positive reg | 3 | 10.244759 | 0.0335696 | 44.199178 | CXCL13, CXCL9, ZFP580 |
| positive reg | 3 | 10.244759 | 0.0335696 | 44.199178 | CXCL13, CCR2, CCL5 |
| defense res | 9 | 2.4136867 | 0.034009 | 44.631053 | LYZ2, IRGM2, CLEC4E, CXCL13, PGLYRP1, HP, STAB2, SPN, PLAC8 |
| positive reg | 5 | 4.0653806 | 0.0342612 | 44.87751 | CCL12, S100A8, S100A9, CCR2, CCL5 |
| response to | 9 | 2.4011154 | 0.0348719 | 45.470005 | ACVRL1, ALAS2, FLT1, ADM, HIF3A, NPPC, APOLD1, KCNA5, CASP1 |
| regulation c | 4 | 5.5377077 | 0.0348981 | 45.495313 | P2RY12, SLN, ADORA2A, AGT |
| positive reg | 5 | 3.940292 | 0.037799 | 48.227747 | CDKN1A, AGT, SPHK1, CD74, FN1 |
| positive reg | 3 | 9.6044618 | 0.0378805 | 48.302604 | INHBA, AGT, KLF4 |
| positive reg | 3 | 9.6044618 | 0.0378805 | 48.302604 | C3AR1, C5AR1, CCL5 |
| neutrophil a | 2 | 51.223796 | 0.0385558 | 48.91907 | S100A8, S100A9 |
| positive reg | 2 | 51.223796 | 0.0385558 | 48.91907 | PTPRC, CCR2 |
| GABAergic | 2 | 51.223796 | 0.0385558 | 48.91907 | GATA2, INHBA |
| regulation c | 9 | 2.3166541 | 0.0417171 | 51.713961 | INHBA, TNFRSF11B, CASP4, AGT, LEFTY2, PMAIP1, FAS, CASP1, ALX4 |
| monocyte c | 4 | 5.1223796 | 0.0425404 | 52.417803 | CCL12, FLT1, CCR2, ANXA1 |
| response to | 4 | 5.1223796 | 0.0425404 | 52.417803 | ADM, AGT, PCSK1N, PLAC8 |
| MAPK casc | 5 | 3.7118693 | 0.0454806 | 54.853494 | ZFP36, CCL12, EFNA1, PLVAP, CCL5 |
| drug metab | 3 | 8.5372993 | 0.0470881 | 56.135017 | CYP4F18, SULT1A1, FMO2 |

| | | | | | |
|---|---|---|---|---|---|
| microglial c | 3 | 8.5372993 | 0.0470881 | 56.135017 | AIF1, CASP1, TLR7 |
| negative reg | 4 | 4.8784568 | 0.0480613 | 56.89421 | FBLN1, ACVRL1, MYO1F, SPN |
| positive reg | 14 | 1.7973262 | 0.0494929 | 57.988433 | MYLK2, RNF207, CCL5, PTGFR, ZFP580, VASH1, PLAUR, LCN2, INHBA, AGT, DLL4, CTSH, KLF4, FN1 |
| cell-cell sig | 6 | 2.9839104 | 0.0508238 | 58.982195 | LNPEP, WNT5B, CXCL13, CCR1, CCL5, GJC2 |
| blood vesse | 4 | 4.7650043 | 0.0509471 | 59.073132 | ACVRL1, DLL4, CCR2, FOXC2 |
| negative reg | 5 | 3.5572081 | 0.0517711 | 59.675993 | NGP, AGT, CCR2, ADAMTS1, VASH1 |
| positive reg | 3 | 8.0879678 | 0.0519663 | 59.817618 | IL4RA, CXCL9, SCGB3A1 |
| positive reg | 3 | 8.0879678 | 0.0519663 | 59.817618 | PTPRC, H2-D1, H2-T23 |
| positive reg | 6 | 2.955219 | 0.0525786 | 60.258743 | SSTR4, PTPRC, TNFRSF11B, FLT1, AGT, FAS |
| aging | 8 | 2.3687305 | 0.0528971 | 60.486316 | CDKN1C, APOD, ADM, AGT, ENDOG, MADCAM1, ITGB2, HTR2A |
| transmembr | 13 | 1.8294213 | 0.0548414 | 61.849568 | SLC22A8, KCNA1, CLCNKA, KCNA3, KCNA5, SLC47A1, ITPR3, ABCB1A, TAP1, SLC37A2, SLC2A1, ABCC3, SLC22A6 |
| negative reg | 6 | 2.8994602 | 0.0561933 | 62.771219 | ACVRL1, CYP1B1, IFITM1, IL1RN, ERDR1, KLF4 |
| adenylate c | 4 | 4.5532263 | 0.0569642 | 63.287333 | P2RY12, HTR1B, ADCY9, HTR2A |
| negative reg | 3 | 7.6835694 | 0.0570158 | 63.321661 | ACVRL1, DLL4, VASH1 |
| positive reg | 2 | 34.149197 | 0.0572742 | 63.493015 | ANXA1, HCAR2 |
| negative reg | 2 | 34.149197 | 0.0572742 | 63.493015 | IL1R2, IL1RN |
| B cell medi | 2 | 34.149197 | 0.0572742 | 63.493015 | PIRB, FAS |
| T cell activ | 2 | 34.149197 | 0.0572742 | 63.493015 | ITGAL, ICAM1 |
| negative reg | 2 | 34.149197 | 0.0572742 | 63.493015 | CCL12, KLRK1 |
| cellular resp | 2 | 34.149197 | 0.0572742 | 63.493015 | KLF2, KLF4 |
| cellular resp | 5 | 3.4149197 | 0.0585119 | 64.303301 | P2RY12, CCL12, MSR1, CYP1B1, CCL5 |
| cellular resp | 6 | 2.7940252 | 0.0638417 | 67.603019 | LCN2, ZFP36, CCL12, ICAM1, CCL5, KLF2 |
| cell chemot | 5 | 3.2835767 | 0.0656978 | 68.683092 | CXCL1, CCL12, CXCL13, CXCL9, SAA3 |
| cellular resp | 5 | 3.2835767 | 0.0656978 | 68.683092 | CASP4, AGT, FAS, CASP1, TLR7 |
| positive reg | 4 | 4.2686497 | 0.0665874 | 69.188666 | GATA2, FCGR2B, C3, FCGR3 |
| positive reg | 4 | 4.2686497 | 0.0665874 | 69.188666 | CDKN1A, ACER2, HP, FAS |
| vasodilation | 3 | 6.9850631 | 0.0675949 | 69.751934 | ADORA2A, AGT, GCH1 |
| cellular resp | 3 | 6.9850631 | 0.0675949 | 69.751934 | EGR3, FLT1, DLL4 |
| cellular resp | 3 | 6.9850631 | 0.0675949 | 69.751934 | SSTR4, ZFP36, ANXA1 |
| leukocyte c | 3 | 6.9850631 | 0.0675949 | 69.751934 | CCR1, S100A9, CKLF |
| positive reg | 3 | 6.9850631 | 0.0675949 | 69.751934 | PTPRC, H2-AA, CD74 |
| positive reg | 4 | 4.1815344 | 0.0699498 | 71.030975 | ZFP36, WNT5B, ZBTB16, HTR2A |
| signal trans | 33 | 1.3469205 | 0.071753 | 71.975767 | GPR182, C3AR1, GPR84, ACVRL1, WNT5B, ADORA2A, GPR63, CCR1, GPR65, GPR88, TLR7, CD74, TNFRSF11B, HTR1B, CXCR6, FAS, HCAR2, FYB, DEPDC7, C5AR1, SOCS3, TLR13, ANXA1, PTGFR, P2RY12, SSTR4, GPR35, FZD10, GPR34, TENM3, CCR2, HTR2A, RIN3 |
| cell cycle a | 5 | 3.1619627 | 0.0733212 | 72.77375 | CDKN1C, INHBA, CDKN1A, VASH1, SLFN1 |
| female preg | 5 | 3.1619627 | 0.0733212 | 72.77375 | A2M, THBD, ADM, AGT, FOSB |
| gamma-am | 2 | 25.611898 | 0.0756292 | 73.909344 | SLC6A12, SLC6A13 |
| positive reg | 2 | 25.611898 | 0.0756292 | 73.909344 | CD244, H2-T23 |
| endocardial | 2 | 25.611898 | 0.0756292 | 73.909344 | ERG, EFNA1 |
| antigen pro | 2 | 25.611898 | 0.0756292 | 73.909344 | H2-AA, H2-AB1 |
| positive reg | 2 | 25.611898 | 0.0756292 | 73.909344 | S100A8, S100A9 |
| response to | 2 | 25.611898 | 0.0756292 | 73.909344 | IER3, SPN |
| negative reg | 2 | 25.611898 | 0.0756292 | 73.909344 | GZMA, HP |
| negative reg | 2 | 25.611898 | 0.0756292 | 73.909344 | NGP, VASH1 |
| positive reg | 8 | 2.179736 | 0.0763494 | 74.254495 | CCL12, ICAM1, C5AR1, C3, CCR1, CCL5, CD74, HTR2A |
| insulin rece | 4 | 4.0175526 | 0.076899 | 74.514953 | SOGA1, BAIAP2L1, PDK4, FOXC2 |
| xenobiotic | 3 | 6.4029745 | 0.0787606 | 75.378939 | CYP1B1, SULT1A1, FMO2 |
| positive reg | 3 | 6.4029745 | 0.0787606 | 75.378939 | AIF1, ANXA1, KCNA5 |
| phagocytos | 4 | 3.940292 | 0.0804831 | 76.153757 | ITGAL, GATA2, ANXA1, ITGB2 |
| hemopoiesi | 5 | 2.9781277 | 0.0869735 | 78.871841 | GATA2, CCR2, HBB-BS, ZBTB16, HBB-BT |
| negative reg | 17 | 1.5385239 | 0.0880522 | 79.294324 | IER3, EGR3, AIF1, SOCS3, IL1RN, SPHK1, PTGFR, MIEN1, CD74, PLAC8, PLAUR, CDKN1A, THOC6, FAS, CTSH, FN1, SPP1 |
| arachidonic | 3 | 5.910438 | 0.0904523 | 80.205879 | ANXA1, PLA2G3, PLA2G2F |
| complemen | 4 | 3.725367 | 0.0916572 | 80.649149 | C4B, C3, CFI, C1QC |
| regulation c | 2 | 20.489518 | 0.0936278 | 81.353992 | HBB-BS, HBB-BT |
| B cell chem | 2 | 20.489518 | 0.0936278 | 81.353992 | CXCL13, CH25H |
| cellular oxi | 2 | 20.489518 | 0.0936278 | 81.353992 | HBB-BS, HBB-BT |
| non-canoni | 2 | 20.489518 | 0.0936278 | 81.353992 | FZD10, MYOC |
| granzyme-r | 2 | 20.489518 | 0.0936278 | 81.353992 | GZMB, SRGN |
| negative reg | 2 | 20.489518 | 0.0936278 | 81.353992 | CYP1B1, ACER2 |
| peptidyl-cy | 2 | 20.489518 | 0.0936278 | 81.353992 | S100A8, S100A9 |
| defense resp | 4 | 3.6588426 | 0.0955177 | 82.007232 | REG3B, LYZ2, ADM, CD4 |
| anion transp | 3 | 5.6915329 | 0.0964775 | 82.330647 | SLC37A2, SLC22A6, SLC4A1 |
| Notch signa | 6 | 2.4587422 | 0.0974144 | 82.641099 | DLL4, FOXC2, GZMB, HP, KCNA5, DLK1 |

**Dataset S8. Differentially methylated genes distributed in different elements.**

| Chr | Start | End | Methyl_R | Methyl_S | Diff_methy | Direction | Gene ID | Element |
|---|---|---|---|---|---|---|---|---|
| chr10 | 122802290 | 122802677 | 0.6404 | 0.1845 | 0.4559 | Hyper | chr10:122802275:122802612\|CGI | CGI |
| chr10 | 13090410 | 13091128 | 0.2253 | 0.7861 | -0.5608 | Hypo | chr10:13090532:13091299\|CGI | CGI |
| chr10 | 75062003 | 75062133 | 0.4914 | 0.1346 | 0.3567 | Hyper | chr10:75060384:75062209\|CGI | CGI |
| chr10 | 80131424 | 80131533 | 0.5086 | 0.2246 | 0.2841 | Hyper | chr10:80131424:80132234\|CGI | CGI |
| chr10 | 80131956 | 80132047 | 0.5212 | 0.8289 | -0.3078 | Hypo | chr10:80131424:80132234\|CGI | CGI |
| chr10 | 80313291 | 80313468 | 0.5305 | 0.8654 | -0.3349 | Hypo | chr10:80312493:80313377\|CGI | CGI |
| chr10 | 80381421 | 80381841 | 0.9152 | 0.483 | 0.4323 | Hyper | chr10:80381560:80382565\|CGI | CGI |
| chr10 | 90969519 | 90969860 | 0.3992 | 0.1183 | 0.2809 | Hyper | chr10:90969500:90970147\|CGI | CGI |
| chr1 | 104993858 | 104994178 | 0.8928 | 0.5056 | 0.3872 | Hyper | chr1:104993908:104994456\|CGI | CGI |
| chr11 | 102665525 | 102665668 | 0.3733 | 0.898 | -0.5247 | Hypo | chr11:102665168:102666458\|CGI | CGI |
| chr11 | 103811332 | 103811398 | 0.7898 | 0.4665 | 0.3233 | Hyper | chr11:103811332:103811622\|CGI | CGI |
| chr11 | 116232366 | 116232981 | 0.8071 | 0.2994 | 0.5077 | Hyper | chr11:116232744:116232982\|CGI | CGI |
| chr11 | 120185795 | 120186202 | 0.1476 | 0.6449 | -0.4973 | Hypo | chr11:120185431:120186075\|CGI | CGI |
| chr11 | 12025846 | 12025935 | 0.6593 | 0.9017 | -0.2424 | Hypo | chr11:12025554:12026580\|CGI | CGI |
| chr11 | 120608785 | 120608876 | 0.2346 | 0.62 | -0.3854 | Hypo | chr11:120608514:120608920\|CGI | CGI |
| chr1 | 120603979 | 120604059 | 0.2767 | 0.6117 | -0.335 | Hypo | chr1:120602843:120603990\|CGI | CGI |
| chr11 | 33123087 | 33123158 | 0.5893 | 0.2018 | 0.3875 | Hyper | chr11:33122877:33123337\|CGI | CGI |
| chr1 | 136260835 | 136260914 | 0.7221 | 0.1459 | 0.5762 | Hyper | chr1:136259769:136260914\|CGI | CGI |
| chr1 | 159232384 | 159232591 | 0.737 | 0.1922 | 0.5448 | Hyper | chr1:159231781:159232846\|CGI | CGI |
| chr11 | 6006453 | 6006856 | 0.6183 | 0.367 | 0.2512 | Hyper | chr11:6006193:6007473\|CGI | CGI |
| chr11 | 64520219 | 64520359 | 0.8003 | 0.3456 | 0.4547 | Hyper | chr11:64520264:64520799\|CGI | CGI |
| chr11 | 75195347 | 75195598 | 0.156 | 0.6641 | -0.5081 | Hypo | chr11:75193256:75195382\|CGI | CGI |
| chr1 | 189751033 | 189751207 | 0.5171 | 0.8196 | -0.3025 | Hypo | chr1:189751188:189751478\|CGI | CGI |
| chr1 | 193632865 | 193632924 | 0.1623 | 0.9069 | -0.7447 | Hypo | chr1:193632700:193632903\|CGI | CGI |
| chr11 | 97534482 | 97534908 | 0.1853 | 0.7393 | -0.554 | Hypo | chr11:97534456:97535279\|CGI | CGI |
| chr11 | 97536299 | 97536595 | 0.2496 | 0.7086 | -0.459 | Hypo | chr11:97536298:97537398\|CGI | CGI |
| chr11 | 99932029 | 99932240 | 0.7964 | 0.4956 | 0.3007 | Hyper | chr11:99932028:99932303\|CGI | CGI |
| chr12 | 109528167 | 109528626 | 0.759 | 0.3471 | 0.4119 | Hyper | chr12:109528252:109528472\|CGI | CGI |
| chr12 | 17175509 | 17175658 | 0.4896 | 0.8488 | -0.3592 | Hypo | chr12:17175491:17176726\|CGI | CGI |
| chr12 | 18610245 | 18610337 | 0.3881 | 0.0931 | 0.295 | Hyper | chr12:18609904:18610691\|CGI | CGI |
| chr12 | 19490348 | 19490434 | 0.2674 | 0.5528 | -0.2854 | Hypo | chr12:19489982:19490838\|CGI | CGI |
| chr12 | 24252465 | 24252588 | 0.5214 | 0.1511 | 0.3703 | Hyper | chr12:24251936:24252731\|CGI | CGI |
| chr12 | 30002546 | 30002700 | 0.954 | 0.6698 | 0.2841 | Hyper | chr12:30002417:30002806\|CGI | CGI |
| chr12 | 80168172 | 80168792 | 0.3642 | 0.7838 | -0.4196 | Hypo | chr12:80168268:80168631\|CGI | CGI |
| chr13 | 100616428 | 100616559 | 0.4715 | 0.0718 | 0.3996 | Hyper | chr13:100616515:100617016\|CGI | CGI |
| chr13 | 100616617 | 100616726 | 0.4075 | 0.0563 | 0.3512 | Hyper | chr13:100616515:100617016\|CGI | CGI |
| chr13 | 109768153 | 109768353 | 0.8876 | 0.137 | 0.7506 | Hyper | chr13:109768153:109768614\|CGI | CGI |
| chr13 | 119488358 | 119489121 | 0.823 | 0.3578 | 0.4652 | Hyper | chr13:119487972:119489121\|CGI | CGI |
| chr13 | 3478374 | 3478534 | 0.4411 | 0.1041 | 0.3369 | Hyper | chr13:3478175:3478490\|CGI | CGI |
| chr13 | 34916665 | 34917056 | 0.3911 | 0.7807 | -0.3896 | Hypo | chr13:34916755:34917059\|CGI | CGI |
| chr13 | 44729740 | 44729897 | 0.132 | 0.5171 | -0.3851 | Hypo | chr13:44729660:44731359\|CGI | CGI |
| chr13 | 44869945 | 44870069 | 0.3443 | 0.6059 | -0.2615 | Hypo | chr13:44869608:44870077\|CGI | CGI |
| chr13 | 53118844 | 53118898 | 0.7766 | 0.4381 | 0.3385 | Hyper | chr13:53118692:53118926\|CGI | CGI |
| chr13 | 65258078 | 65258684 | 0.6341 | 0.1408 | 0.4933 | Hyper | chr13:65258068:65258463\|CGI | CGI |
| chr13 | 67892681 | 67892799 | 0.6557 | 0.9601 | -0.3044 | Hypo | chr13:67892443:67893174\|CGI | CGI |
| chr13 | 74166816 | 74167032 | 0.1864 | 0.7805 | -0.5941 | Hypo | chr13:74166889:74167109\|CGI | CGI |
| chr13 | 78194876 | 78195220 | 0.5332 | 0.0846 | 0.4487 | Hyper | chr13:78194759:78195573\|CGI | CGI |
| chr13 | 95336416 | 95336478 | 0.4054 | 0.7635 | -0.3581 | Hypo | chr13:95336130:95336545\|CGI | CGI |
| chr13 | 9834319 | 9834561 | 0.9346 | 0.5243 | 0.4102 | Hyper | chr13:9834340:9834561\|CGI | CGI |
| chr14 | 46831811 | 46831870 | 0.0506 | 0.2741 | -0.2235 | Hypo | chr14:46831779:46832643\|CGI | CGI |
| chr14 | 55113211 | 55113434 | 0.471 | 0.8522 | -0.3812 | Hypo | chr14:55113414:55115427\|CGI | CGI |
| chr1 | 45735217 | 45735295 | 0.9302 | 0.5025 | 0.4277 | Hyper | chr1:45735236:45735659\|CGI | CGI |
| chr14 | 66045090 | 66045183 | 0.8933 | 0.5402 | 0.3531 | Hyper | chr14:66044940:66045184\|CGI | CGI |
| chr14 | 70080241 | 70080427 | 0.1562 | 0.5047 | -0.3485 | Hypo | chr14:70074548:70080496\|CGI | CGI |
| chr14 | 79661353 | 79661749 | 0.7209 | 0.3462 | 0.3747 | Hyper | chr14:79661295:79661614\|CGI | CGI |
| chr15 | 100729510 | 100729723 | 0.5494 | 0.1265 | 0.4229 | Hyper | chr15:100728988:100729740\|CGI | CGI |
| chr15 | 101054315 | 101054418 | 0.7712 | 0.4616 | 0.3096 | Hyper | chr15:101052673:101054340\|CGI | CGI |
| chr15 | 81859523 | 81859731 | 0.0914 | 0.3853 | -0.294 | Hypo | chr15:81859644:81859877\|CGI | CGI |
| chr15 | 89068179 | 89068301 | 0.4165 | 0.8205 | -0.404 | Hypo | chr15:89067581:89068614\|CGI | CGI |
| chr1 | 59483748 | 59483937 | 0.3884 | 0.0775 | 0.3108 | Hyper | chr1:59481951:59483937\|CGI | CGI |
| chr16 | 13868315 | 13868425 | 0.9229 | 0.1981 | 0.7248 | Hyper | chr16:13868019:13868377\|CGI | CGI |
| chr16 | 16507102 | 16507230 | 0.4977 | 0.8957 | -0.3981 | Hypo | chr16:16507127:16508285\|CGI | CGI |
| chr1 | 62295351 | 62295472 | 0.2426 | 0.7361 | -0.4935 | Hypo | chr1:62295416:62295694\|CGI | CGI |
| chr16 | 32149421 | 32149551 | 0.3965 | 0.1026 | 0.2939 | Hyper | chr16:32149230:32149461\|CGI | CGI |
| chr16 | 85899640 | 85899815 | 0.3638 | 0.1205 | 0.2433 | Hyper | chr16:85899485:85901102\|CGI | CGI |
| chr16 | 89867409 | 89867730 | 0.7807 | 0.2732 | 0.5075 | Hyper | chr16:89867462:89867681\|CGI | CGI |
| chr16 | 90011445 | 90011724 | 0.8688 | 0.5913 | 0.2775 | Hyper | chr16:90011465:90011735\|CGI | CGI |
| chr16 | 96280923 | 96281039 | 0.1357 | 0.4582 | -0.3225 | Hypo | chr16:96280784:96281114\|CGI | CGI |
| chr17 | 12741732 | 12741785 | 0.3558 | 0.9314 | -0.5756 | Hypo | chr17:12741440:12742489\|CGI | CGI |
| chr17 | 23806918 | 23807155 | 0.0832 | 0.5538 | -0.4705 | Hypo | chr17:23806678:23807174\|CGI | CGI |
| chr17 | 26439000 | 26439372 | 0.1892 | 0.5932 | -0.404 | Hypo | chr17:26438676:26439351\|CGI | CGI |
| chr17 | 27639163 | 27639358 | 0.8586 | 0.4762 | 0.3824 | Hyper | chr17:27639117:27639359\|CGI | CGI |
| chr17 | 35359313 | 35360018 | 0.7118 | 0.1199 | 0.5919 | Hyper | chr17:35359103:35359683\|CGI | CGI |
| chr17 | 35835358 | 35835414 | 0.5698 | 0.2467 | 0.3231 | Hyper | chr17:35835357:35835711\|CGI | CGI |
| chr17 | 45544994 | 45545073 | 0.1829 | 0.0026 | 0.1804 | Hyper | chr17:45544794:45545946\|CGI | CGI |
| chr17 | 56124127 | 56124640 | 0.7165 | 0.2701 | 0.4464 | Hyper | chr17:56123812:56124909\|CGI | CGI |
| chr17 | 56510986 | 56511040 | 0.533 | 0.2037 | 0.3293 | Hyper | chr17:56510863:56512467\|CGI | CGI |
| chr17 | 56511827 | 56512145 | 0.7251 | 0.2628 | 0.4622 | Hyper | chr17:56510863:56512467\|CGI | CGI |
| chr17 | 6948531 | 6948786 | 0.3548 | 0.075 | 0.2798 | Hyper | chr17:6947995:6948787\|CGI | CGI |
| chr17 | 84067279 | 84067406 | 0.718 | 0.3702 | 0.3478 | Hyper | chr17:84067171:84067444\|CGI | CGI |
| chr17 | 85685209 | 85685278 | 0.1561 | 0.444 | -0.2879 | Hypo | chr17:85685053:85685475\|CGI | CGI |
| chr17 | 87640043 | 87640291 | 0.806 | 0.3534 | 0.4526 | Hyper | chr17:87640236:87640518\|CGI | CGI |
| chr17 | 91088676 | 91088811 | 0.4339 | 0.8591 | -0.4251 | Hypo | chr17:91087924:91088778\|CGI | CGI |
| chr18 | 12973196 | 12973463 | 0.3574 | 0.7375 | -0.3801 | Hypo | chr18:12973116:12974420\|CGI | CGI |
| chr18 | 30267566 | 30267617 | 0.3934 | 0.0595 | 0.3339 | Hyper | chr18:30267062:30268266\|CGI | CGI |
| chr18 | 30267696 | 30267808 | 0.457 | 0.0621 | 0.3948 | Hyper | chr18:30267062:30268266\|CGI | CGI |
| chr18 | 30267906 | 30267969 | 0.4516 | 0.0585 | 0.393 | Hyper | chr18:30267062:30268266\|CGI | CGI |
| chr18 | 31938999 | 31939313 | 0.6747 | 0.3151 | 0.3596 | Hyper | chr18:31938999:31939456\|CGI | CGI |
| chr1 | 89216974 | 89217047 | 0.9478 | 0.4549 | 0.4928 | Hyper | chr1:89216766:89217487\|CGI | CGI |
| chr19 | 37686993 | 37687223 | 0.2128 | 0.5602 | -0.3474 | Hypo | chr19:37687046:37687836\|CGI | CGI |
| chr19 | 37691010 | 37691070 | 0.6906 | 0.1614 | 0.5292 | Hyper | chr19:37690830:37691070\|CGI | CGI |
| chr1 | 93940549 | 93940790 | 0.0861 | 0.8838 | -0.7977 | Hypo | chr1:93940675:93940991\|CGI | CGI |
| chr19 | 5307318 | 5307814 | 0.4541 | 0.8075 | -0.3534 | Hypo | chr19:5306752:5307815\|CGI | CGI |
| chr19 | 6413012 | 6413473 | 0.9223 | 0.6223 | 0.3 | Hyper | chr19:6413127:6413349\|CGI | CGI |
| chr19 | 7421484 | 7421623 | 0.8024 | 0.2734 | 0.529 | Hyper | chr19:7421280:7421530\|CGI | CGI |
| chr19 | 7423530 | 7423728 | 0.6758 | 0.138 | 0.5378 | Hyper | chr19:7422043:7423728\|CGI | CGI |
| chr2 | 115860575 | 115860639 | 0.4825 | 0.1313 | 0.3513 | Hyper | chr2:115860418:115860660\|CGI | CGI |
| chr2 | 116062137 | 116062239 | 0.4352 | 0.1393 | 0.2959 | Hyper | chr2:116062235:116062438\|CGI | CGI |
| chr2 | 150500473 | 150500574 | 0.6892 | 0.3997 | 0.2895 | Hyper | chr2:150500521:150500919\|CGI | CGI |
| chr2 | 152686785 | 152687142 | 0.7147 | 0.2897 | 0.425 | Hyper | chr2:152686809:152687230\|CGI | CGI |
| chr2 | 155386810 | 155386892 | 0.1957 | 0.6801 | -0.4844 | Hypo | chr2:155386547:155386840\|CGI | CGI |
| chr2 | 157039956 | 157040052 | 0.4923 | 0.88 | -0.3877 | Hypo | chr2:157040038:157040753\|CGI | CGI |
| chr2 | 157560182 | 157560249 | 0.6779 | 0.3162 | 0.3617 | Hyper | chr2:157560050:157560581\|CGI | CGI |
| chr2 | 165112365 | 165112494 | 0.1038 | 0.3643 | -0.2605 | Hypo | chr2:165112049:165112596\|CGI | CGI |
| chr2 | 174284946 | 174285292 | 0.5291 | 0.8604 | -0.3314 | Hypo | chr2:174284647:174285390\|CGI | CGI |
| chr2 | 174299759 | 174300328 | 0.6218 | 0.9289 | -0.3071 | Hypo | chr2:174298463:174300644\|CGI | CGI |
| chr2 | 181148132 | 181148199 | 0.5969 | 0.1209 | 0.4761 | Hyper | chr2:181147685:181148487\|CGI | CGI |
| chr2 | 29751670 | 29751795 | 0.1303 | 0.4956 | -0.3653 | Hypo | chr2:29751441:29751901\|CGI | CGI |
| chr2 | 32741489 | 32741790 | 0.6561 | 0.1306 | 0.5255 | Hyper | chr2:32741428:32741833\|CGI | CGI |

| | | | | | | | | |
|---|---|---|---|---|---|---|---|---|
| chr2 | 6100689 | 6100758 | 0.6251 | 0.2633 | 0.3618 | Hyper | chr2:6100358:6101023\|CGI | CGI |
| chr2 | 87525905 | 87526178 | 0.6146 | 0.2107 | 0.4039 | Hyper | chr2:87526007:87526220\|CGI | CGI |
| chr2 | 98389501 | 98389590 | 0.9271 | 0.4526 | 0.4745 | Hyper | chr2:98389470:98389901\|CGI | CGI |
| chr3 | 102011907 | 102012204 | 0.5491 | 0.1622 | 0.387 | Hyper | chr3:102011670:102013119\|CGI | CGI |
| chr3 | 102012291 | 102012351 | 0.5312 | 0.1634 | 0.3679 | Hyper | chr3:102011670:102013119\|CGI | CGI |
| chr3 | 88241318 | 88241413 | 0.6805 | 0.2342 | 0.4463 | Hyper | chr3:88240996:88241413\|CGI | CGI |
| chr4 | 101870426 | 101871571 | 0.7861 | 0.2406 | 0.5455 | Hyper | chr4:101871032:101871588\|CGI | CGI |
| chr4 | 106622330 | 106622738 | 0.1939 | 0.8612 | -0.6673 | Hypo | chr4:106622395:106623099\|CGI | CGI |
| chr4 | 120268100 | 120268174 | 0.4464 | 0.1618 | 0.2847 | Hyper | chr4:120267946:120269173\|CGI | CGI |
| chr4 | 12137050 | 12137156 | 0.9635 | 0.4074 | 0.5561 | Hyper | chr4:12137128:12137457\|CGI | CGI |
| chr4 | 125729578 | 125730002 | 0.3727 | 0.1054 | 0.2672 | Hyper | chr4:125729578:125729806\|CGI | CGI |
| chr4 | 132113730 | 132113998 | 0.6694 | 0.317 | 0.3524 | Hyper | chr4:132113502:132113980\|CGI | CGI |
| chr4 | 150910037 | 150910191 | 0.2339 | 0.6377 | -0.4038 | Hypo | chr4:150909546:150910075\|CGI | CGI |
| chr4 | 154634789 | 154634992 | 0.6545 | 0.176 | 0.4786 | Hyper | chr4:154634782:154637997\|CGI | CGI |
| chr4 | 73834769 | 73834993 | 0.9504 | 0.4898 | 0.4606 | Hyper | chr4:73834768:73835012\|CGI | CGI |
| chr4 | 78513257 | 78513730 | 0.6466 | 0.9504 | -0.3038 | Hypo | chr4:78513216:78513681\|CGI | CGI |
| chr5 | 116444456 | 116444869 | 0.7762 | 0.2132 | 0.5629 | Hyper | chr5:116444534:116444762\|CGI | CGI |
| chr5 | 118237426 | 118237571 | 0.5146 | 0.9813 | -0.4668 | Hypo | chr5:118237222:118237438\|CGI | CGI |
| chr5 | 123135435 | 123135501 | 0.4898 | 0.1763 | 0.3135 | Hyper | chr5:123135435:123135716\|CGI | CGI |
| chr5 | 135911032 | 135911399 | 0.3679 | 0.7703 | -0.4024 | Hypo | chr5:135911011:135911681\|CGI | CGI |
| chr5 | 139619427 | 139619673 | 0.8249 | 0.3967 | 0.4282 | Hyper | chr5:139619368:139619694\|CGI | CGI |
| chr5 | 143253376 | 143253558 | 0.3774 | 0.1129 | 0.2645 | Hyper | chr5:143253002:143255255\|CGI | CGI |
| chr5 | 24485139 | 24485490 | 0.5503 | 0.1634 | 0.3869 | Hyper | chr5:24485138:24485418\|CGI | CGI |
| chr5 | 77016012 | 77016309 | 0.3512 | 0.0668 | 0.2844 | Hyper | chr5:77015850:77016234\|CGI | CGI |
| chr6 | 141753848 | 141754094 | 0.4393 | 0.9102 | -0.4709 | Hypo | chr6:141753554:141753941\|CGI | CGI |
| chr6 | 17840413 | 17840717 | 0.5246 | 0.884 | -0.3594 | Hypo | chr6:17840455:17840718\|CGI | CGI |
| chr6 | 4747440 | 4747735 | 0.7328 | 0.4417 | 0.2912 | Hyper | chr6:4747208:4748412\|CGI | CGI |
| chr6 | 48439255 | 48439318 | 0.8822 | 0.5047 | 0.3774 | Hyper | chr6:48438015:48439276\|CGI | CGI |
| chr6 | 58906723 | 58907187 | 0.7644 | 0.4608 | 0.3036 | Hyper | chr6:58906723:58907147\|CGI | CGI |
| chr6 | 6864072 | 6864282 | 0.0945 | 0.7881 | -0.6936 | Hypo | chr6:6863521:6864073\|CGI | CGI |
| chr7 | 114562985 | 114563068 | 0.395 | 0.1022 | 0.2928 | Hyper | chr7:114562569:114563069\|CGI | CGI |
| chr7 | 127386582 | 127386859 | 0.1125 | 0.4392 | -0.3267 | Hypo | chr7:127386621:127386953\|CGI | CGI |
| chr7 | 141693657 | 141693819 | 0.7391 | 0.361 | 0.3781 | Hyper | chr7:141693540:141693819\|CGI | CGI |
| chr7 | 142082194 | 142082264 | 0.7136 | 0.3942 | 0.3194 | Hyper | chr7:142082125:142082742\|CGI | CGI |
| chr7 | 143295214 | 143295509 | 0.2652 | 0.6209 | -0.3557 | Hypo | chr7:143295404:143295824\|CGI | CGI |
| chr7 | 143296075 | 143296599 | 0.5193 | 0.9291 | -0.4098 | Hypo | chr7:143296313:143296559\|CGI | CGI |
| chr7 | 15731448 | 15731600 | 0.4482 | 0.9807 | -0.5325 | Hypo | chr7:15731031:15732196\|CGI | CGI |
| chr7 | 19005146 | 19005246 | 0.6586 | 0.1946 | 0.464 | Hyper | chr7:19003927:19005664\|CGI | CGI |
| chr7 | 19809500 | 19809775 | 0.1343 | 0.5429 | -0.4086 | Hypo | chr7:19809463:19809725\|CGI | CGI |
| chr7 | 25314541 | 25314857 | 0.1905 | 0.8741 | -0.6836 | Hypo | chr7:25314707:25316012\|CGI | CGI |
| chr7 | 25422651 | 25423040 | 0.8549 | 0.2973 | 0.5576 | Hyper | chr7:25422817:25423041\|CGI | CGI |
| chr7 | 31052107 | 31052315 | 0.1793 | 0.5256 | -0.3462 | Hypo | chr7:31052107:31052335\|CGI | CGI |
| chr7 | 36695217 | 36695498 | 0.1849 | 0.5812 | -0.3963 | Hypo | chr7:36695312:36698700\|CGI | CGI |
| chr7 | 44970486 | 44970668 | 0.1037 | 0.5064 | -0.4027 | Hypo | chr7:44970450:44970699\|CGI | CGI |
| chr7 | 54923188 | 54923250 | 0.2481 | 0.0108 | 0.2373 | Hyper | chr7:54923149:54923680\|CGI | CGI |
| chr7 | 6729595 | 6729846 | 0.8263 | 0.3887 | 0.4376 | Hyper | chr7:6729450:6729785\|CGI | CGI |
| chr7 | 7278105 | 7278391 | 0.8316 | 0.4146 | 0.4169 | Hyper | chr7:7277763:7278551\|CGI | CGI |
| chr8 | 105258929 | 105259232 | 0.5869 | 0.1741 | 0.4129 | Hyper | chr8:105258995:105259938\|CGI | CGI |
| chr8 | 105614362 | 105614743 | 0.6625 | 0.1349 | 0.5276 | Hyper | chr8:105613932:105614532\|CGI | CGI |
| chr8 | 120399676 | 120399758 | 0.8033 | 0.2892 | 0.5141 | Hyper | chr8:120399590:120399842\|CGI | CGI |
| chr8 | 120449898 | 120450005 | 0.619 | 0.3032 | 0.3158 | Hyper | chr8:120449898:120450261\|CGI | CGI |
| chr8 | 120598374 | 120598583 | 0.1007 | 0.7425 | -0.6419 | Hypo | chr8:120598491:120598796\|CGI | CGI |
| chr8 | 123603549 | 123603773 | 0.6895 | 0.1812 | 0.5082 | Hyper | chr8:123603301:123603623\|CGI | CGI |
| chr8 | 22013237 | 22013637 | 0.888 | 0.388 | 0.5001 | Hyper | chr8:22013383:22013586\|CGI | CGI |
| chr8 | 56942961 | 56943072 | 0.7564 | 0.3175 | 0.4389 | Hyper | chr8:56942960:56943538\|CGI | CGI |
| chr8 | 69399322 | 69399768 | 0.1025 | 0.4939 | -0.3915 | Hypo | chr8:69399389:69399768\|CGI | CGI |
| chr8 | 70111888 | 70112165 | 0.6584 | 0.2536 | 0.4047 | Hyper | chr8:70111888:70112925\|CGI | CGI |
| chr8 | 70112531 | 70112758 | 0.1975 | 0.6652 | -0.4677 | Hypo | chr8:70111888:70112925\|CGI | CGI |
| chr8 | 70486281 | 70487231 | 0.4831 | 0.3974 | 0.0858 | Hyper | chr8:70487170:70487507\|CGI | CGI |
| chr8 | 70913506 | 70913606 | 0.5257 | 0.8759 | -0.3502 | Hypo | chr8:70912997:70914749\|CGI | CGI |
| chr8 | 71480409 | 71480621 | 0.9015 | 0.3304 | 0.5711 | Hyper | chr8:71480471:71481718\|CGI | CGI |
| chr8 | 71685696 | 71685983 | 0.4095 | 0.0965 | 0.313 | Hyper | chr8:71685723:71686100\|CGI | CGI |
| chr8 | 80739084 | 80739205 | 0.539 | 0.2185 | 0.3205 | Hyper | chr8:80738420:80739970\|CGI | CGI |
| chr8 | 83668031 | 83668262 | 0.0964 | 0.525 | -0.4286 | Hypo | chr8:83668050:83668778\|CGI | CGI |
| chr8 | 84617039 | 84617392 | 0.6537 | 0.1847 | 0.4691 | Hyper | chr8:84617076:84617329\|CGI | CGI |
| chr8 | 87468806 | 87469291 | 0.1233 | 0.5001 | -0.3768 | Hypo | chr8:87468768:87469125\|CGI | CGI |
| chr8 | 87783003 | 87783150 | 0.5888 | 0.9054 | -0.3166 | Hypo | chr8:87783002:87783316\|CGI | CGI |
| chr8 | 94912952 | 94913554 | 0.1441 | 0.584 | -0.4399 | Hypo | chr8:94913065:94913767\|CGI | CGI |
| chr9 | 106226352 | 106226710 | 0.1321 | 0.515 | -0.3829 | Hypo | chr9:106226088:106226477\|CGI | CGI |
| chr9 | 106647285 | 106647506 | 0.7398 | 0.3518 | 0.388 | Hyper | chr9:106647485:106647831\|CGI | CGI |
| chr9 | 108110592 | 108110653 | 0.7748 | 0.4403 | 0.3345 | Hyper | chr9:108110489:108110745\|CGI | CGI |
| chr9 | 123157014 | 123157176 | 0.4365 | 0.884 | -0.4475 | Hypo | chr9:123156818:123157109\|CGI | CGI |
| chr9 | 14750654 | 14751033 | 0.9002 | 0.3068 | 0.5934 | Hyper | chr9:14750818:14752271\|CGI | CGI |
| chr9 | 37384548 | 37384819 | 0.2052 | 0.6167 | -0.4114 | Hypo | chr9:37384547:37384857\|CGI | CGI |
| chr9 | 4260587 | 4260755 | 0.3484 | 0.6904 | -0.342 | Hypo | chr9:4260241:4260842\|CGI | CGI |
| chr9 | 55545526 | 55545632 | 0.3448 | 0.0713 | 0.2735 | Hyper | chr9:55545460:55546720\|CGI | CGI |
| chr9 | 58311625 | 58311751 | 0.7997 | 0.2943 | 0.5053 | Hyper | chr9:58311655:58313120\|CGI | CGI |
| chr9 | 66806058 | 66806179 | 0.5442 | 0.1829 | 0.3613 | Hyper | chr9:66805821:66806225\|CGI | CGI |
| chr9 | 82830200 | 82830475 | 0.3172 | 0.9418 | -0.6246 | Hypo | chr9:82829654:82830312\|CGI | CGI |
| chr9 | 89879600 | 89879925 | 0.8415 | 0.1237 | 0.7178 | Hyper | chr9:89879659:89879905\|CGI | CGI |
| chrX | 100777383 | 100777557 | 0.5989 | 0.1278 | 0.4711 | Hyper | chrX:100777002:100777406\|CGI | CGI |
| chrX | 102933739 | 102934104 | 0.6194 | 0.098 | 0.5214 | Hyper | chrX:102933586:102934272\|CGI | CGI |
| chrX | 104482935 | 104483522 | 0.5198 | 0.1262 | 0.3936 | Hyper | chrX:104482935:104483161\|CGI | CGI |
| chrX | 104670417 | 104670839 | 0.4471 | 0.0643 | 0.3828 | Hyper | chrX:104670705:104670977\|CGI | CGI |
| chrX | 106027162 | 106027248 | 0.4657 | 0.1157 | 0.35 | Hyper | chrX:106027092:106027408\|CGI | CGI |
| chrX | 109013279 | 109013475 | 0.4335 | 0.0698 | 0.3636 | Hyper | chrX:109013333:109013641\|CGI | CGI |
| chrX | 111894414 | 111894629 | 0.8136 | 0.1981 | 0.6154 | Hyper | chrX:111894413:111895559\|CGI | CGI |
| chrX | 123103222 | 123103667 | 0.7762 | 0.307 | 0.4692 | Hyper | chrX:123103269:123103937\|CGI | CGI |
| chrX | 133898013 | 133898533 | 0.4421 | 0.1045 | 0.3376 | Hyper | chrX:133898277:133898499\|CGI | CGI |
| chrX | 134307984 | 134308294 | 0.5177 | 0.1218 | 0.3959 | Hyper | chrX:134307984:134308217\|CGI | CGI |
| chrX | 134601687 | 134601915 | 0.3862 | 0.0814 | 0.3048 | Hyper | chrX:134601154:134601915\|CGI | CGI |
| chrX | 136741903 | 136742212 | 0.6144 | 0.0754 | 0.539 | Hyper | chrX:136741524:136742212\|CGI | CGI |
| chrX | 137117959 | 137118202 | 0.8462 | 0.2626 | 0.5836 | Hyper | chrX:137118148:137120399\|CGI | CGI |
| chrX | 137570885 | 137570968 | 0.8581 | 0.1263 | 0.7319 | Hyper | chrX:137570910:137571192\|CGI | CGI |
| chrX | 138914060 | 138914663 | 0.7405 | 0.1145 | 0.626 | Hyper | chrX:138914059:138914485\|CGI | CGI |
| chrX | 139871474 | 139871733 | 0.5837 | 0.1267 | 0.4569 | Hyper | chrX:139870931:139871778\|CGI | CGI |
| chrX | 140061671 | 140061785 | 0.64 | 0.1945 | 0.4456 | Hyper | chrX:140061078:140062743\|CGI | CGI |
| chrX | 141724331 | 141724665 | 0.4906 | 0.1363 | 0.3543 | Hyper | chrX:141724517:141725435\|CGI | CGI |
| chrX | 142305873 | 142305983 | 0.8251 | 0.1981 | 0.627 | Hyper | chrX:142305513:142305959\|CGI | CGI |
| chrX | 143393870 | 143394308 | 0.6525 | 0.1141 | 0.5384 | Hyper | chrX:143393869:143394250\|CGI | CGI |
| chrX | 151343808 | 151344343 | 0.6595 | 0.1774 | 0.4822 | Hyper | chrX:151344091:151344866\|CGI | CGI |
| chrX | 151521050 | 151521292 | 0.5423 | 0.1485 | 0.3938 | Hyper | chrX:151520296:151521218\|CGI | CGI |
| chrX | 151802982 | 151803249 | 0.5456 | 0.1551 | 0.3906 | Hyper | chrX:151802982:151803955\|CGI | CGI |
| chrX | 153037422 | 153037473 | 0.5047 | 0.1244 | 0.3803 | Hyper | chrX:153037185:153037670\|CGI | CGI |
| chrX | 153037580 | 153037669 | 0.51 | 0.0923 | 0.4177 | Hyper | chrX:153037185:153037670\|CGI | CGI |
| chrX | 153139631 | 153139842 | 0.5841 | 0.1432 | 0.4409 | Hyper | chrX:153139746:153140122\|CGI | CGI |
| chrX | 157598596 | 157598879 | 0.5453 | 0.1489 | 0.3964 | Hyper | chrX:157598158:157598889\|CGI | CGI |
| chrX | 158042276 | 158042753 | 0.5632 | 0.1254 | 0.4378 | Hyper | chrX:158042456:158043904\|CGI | CGI |
| chrX | 159414180 | 159415378 | 0.6808 | 0.0982 | 0.5826 | Hyper | chrX:159414230:159415517\|CGI | CGI |
| chrX | 160502490 | 160502779 | 0.5982 | 0.1282 | 0.47 | Hyper | chrX:160502101:160502871\|CGI | CGI |
| chrX | 160994413 | 160994697 | 0.5363 | 0.1551 | 0.3812 | Hyper | chrX:160994066:160994966\|CGI | CGI |

| | | | | | | | | | |
|---|---|---|---|---|---|---|---|---|---|
| chrX | 162760193 | 162761268 | 0.6817 | 0.1099 | 0.5718 | Hyper | chrX:162760037:162761400\|CGI | CGI |
| chrX | 164076223 | 164076512 | 0.6036 | 0.1304 | 0.4732 | Hyper | chrX:164075915:164076602\|CGI | CGI |
| chrX | 166170231 | 166170358 | 0.5346 | 0.0576 | 0.4771 | Hyper | chrX:166170285:166170559\|CGI | CGI |
| chrX | 16618945 | 16619125 | 0.5954 | 0.0813 | 0.5142 | Hyper | chrX:16618454:16618959\|CGI | CGI |
| chrX | 166510715 | 166510905 | 0.6073 | 0.1764 | 0.4309 | Hyper | chrX:166510067:166510729\|CGI | CGI |
| chrX | 168674198 | 168674553 | 0.6583 | 0.087 | 0.5713 | Hyper | chrX:168673364:168674394\|CGI | CGI |
| chrX | 169994005 | 169994276 | 0.9071 | 0.2923 | 0.6148 | Hyper | chrX:169994005:169994235\|CGI | CGI |
| chrX | 20060707 | 20060958 | 0.6823 | 0.1731 | 0.5093 | Hyper | chrX:20059724:20061120\|CGI | CGI |
| chrX | 20618077 | 20618164 | 0.5078 | 0.116 | 0.3918 | Hyper | chrX:20617032:20618390\|CGI | CGI |
| chrX | 20618257 | 20618508 | 0.469 | 0.1086 | 0.3605 | Hyper | chrX:20617032:20618390\|CGI | CGI |
| chrX | 36328418 | 36328678 | 0.3943 | 0.0756 | 0.3188 | Hyper | chrX:36328331:36330273\|CGI | CGI |
| chrX | 36644743 | 36645387 | 0.7865 | 0.2048 | 0.5817 | Hyper | chrX:36644909:36645291\|CGI | CGI |
| chrX | 36807807 | 36808398 | 0.5539 | 0.1208 | 0.4332 | Hyper | chrX:36807777:36808136\|CGI | CGI |
| chrX | 37127292 | 37127506 | 0.6967 | 0.1762 | 0.5205 | Hyper | chrX:37126928:37127320\|CGI | CGI |
| chrX | 38180982 | 38181355 | 0.6343 | 0.1179 | 0.5164 | Hyper | chrX:38180828:38181252\|CGI | CGI |
| chrX | 41401497 | 41401628 | 0.5409 | 0.0929 | 0.448 | Hyper | chrX:41401379:41401588\|CGI | CGI |
| chrX | 41911146 | 41911217 | 0.5981 | 0.0952 | 0.5029 | Hyper | chrX:41911041:41911984\|CGI | CGI |
| chrX | 48454161 | 48454570 | 0.5227 | 0.1091 | 0.4136 | Hyper | chrX:48452905:48454662\|CGI | CGI |
| chrX | 49950292 | 49950477 | 0.4218 | 0.9589 | -0.5371 | Hypo | chrX:49950392:49950833\|CGI | CGI |
| chrX | 51681002 | 51681367 | 0.5111 | 0.1108 | 0.4003 | Hyper | chrX:51679908:51681589\|CGI | CGI |
| chrX | 52988018 | 52988389 | 0.4667 | 0.0874 | 0.3793 | Hyper | chrX:52988002:52988623\|CGI | CGI |
| chrX | 56374582 | 56374677 | 0.6304 | 0.1981 | 0.4322 | Hyper | chrX:56374455:56375000\|CGI | CGI |
| chrX | 57503417 | 57503590 | 0.8293 | 0.2155 | 0.6138 | Hyper | chrX:57503513:57503908\|CGI | CGI |
| chrX | 59567526 | 59567591 | 0.4412 | 0.0747 | 0.3664 | Hyper | chrX:59567234:59568293\|CGI | CGI |
| chrX | 71051016 | 71051100 | 0.4705 | 0.1279 | 0.3426 | Hyper | chrX:71050987:71051289\|CGI | CGI |
| chrX | 71664516 | 71664717 | 0.1304 | 0.6896 | -0.5592 | Hypo | chrX:71664218:71664718\|CGI | CGI |
| chrX | 7319286 | 7319620 | 0.4868 | 0.1467 | 0.3402 | Hyper | chrX:7318572:7319439\|CGI | CGI |
| chrX | 73422218 | 73422546 | 0.9296 | 0.3302 | 0.5994 | Hyper | chrX:73421939:73422448\|CGI | CGI |
| chrX | 73503594 | 73503774 | 0.5267 | 0.0874 | 0.4393 | Hyper | chrX:73503078:73503605\|CGI | CGI |
| chrX | 73659195 | 73659771 | 0.821 | 0.165 | 0.656 | Hyper | chrX:73659698:73660177\|CGI | CGI |
| chrX | 73967297 | 73967406 | 0.4348 | 0.1017 | 0.3331 | Hyper | chrX:73965822:73967884\|CGI | CGI |
| chrX | 74245734 | 74246231 | 0.6186 | 0.0898 | 0.5288 | Hyper | chrX:74245775:74246710\|CGI | CGI |
| chrX | 75142997 | 75143479 | 0.6575 | 0.1571 | 0.5004 | Hyper | chrX:75143197:75143602\|CGI | CGI |
| chrX | 7841838 | 7842043 | 0.781 | 0.1981 | 0.5828 | Hyper | chrX:7841567:7842696\|CGI | CGI |
| chrX | 7883813 | 7884141 | 0.617 | 0.1688 | 0.4482 | Hyper | chrX:7884049:7884621\|CGI | CGI |
| chrX | 7947584 | 7948092 | 0.6357 | 0.1253 | 0.5105 | Hyper | chrX:7947659:7948269\|CGI | CGI |
| chrX | 8096615 | 8096735 | 0.4108 | 0.0782 | 0.3326 | Hyper | chrX:8096486:8096908\|CGI | CGI |
| chrX | 8145133 | 8146008 | 0.7276 | 0.1056 | 0.622 | Hyper | chrX:8145265:8145872\|CGI | CGI |
| chrX | 94541838 | 94542044 | 0.4827 | 0.1134 | 0.3693 | Hyper | chrX:94541067:94542086\|CGI | CGI |
| chrX | 98890405 | 98890487 | 0.7174 | 0.2095 | 0.5079 | Hyper | chrX:98890404:98891060\|CGI | CGI |
| chrX | 98936883 | 98937547 | 0.6946 | 0.084 | 0.6106 | Hyper | chrX:98936881:98938199\|CGI | CGI |
