# Supplementary material for: A next-generation sequencing study on mechanisms by which restraint and social instability stresses of male mice alter offspring anxiety-like behavior
Source: Sci Rep. 2021 Apr 12;11:7952. doi: 10.1038/s41598-021-87060-x (PMC8042048; doi:10.1038/s41598-021-87060-x)
Supplement: Supplementary file 2 — Supplementary Information 2. [file 41598_2021_87060_MOESM2_ESM.docx]

Title: A next-generation sequencing study on mechanisms by which restraint and social instability stresses of male mice alter offspring anxiety-like behavior

Qiao-Qiao Kong^1,2^, Xiao-Dan Tian^1^, Jia Wang^1^, Hong-Jie Yuan^1^, Shu-Fen Ning^1^, Ming-Jiu Luo^1^ and Jing-He Tan^1,3^

1. Shandong Provincial Key Laboratory of Animal Biotechnology and Disease Control and Prevention, College of Animal Science and Veterinary Medicine, Shandong Agricultural University, Tai'an City, P. R. China

2. Tai’an City Central Hospital, Tai’an City, P. R. China

3. Corresponding author: Jing-He Tan, College of Animal Science and Veterinary Medicine, Shandong Agricultural University, Tai-an City, Shandong Province, P R China, Post code: 271018, Phone: 0538-8249616, FAX: 0538-8241419, Email: [tanjh@sdau.edu.cn](mailto:tanjh@sdau.edu.cn)

**Supplementary figure S1**

**Figure S1-1**

Spermatozoa

Blastocysts

Fetal hippocampi


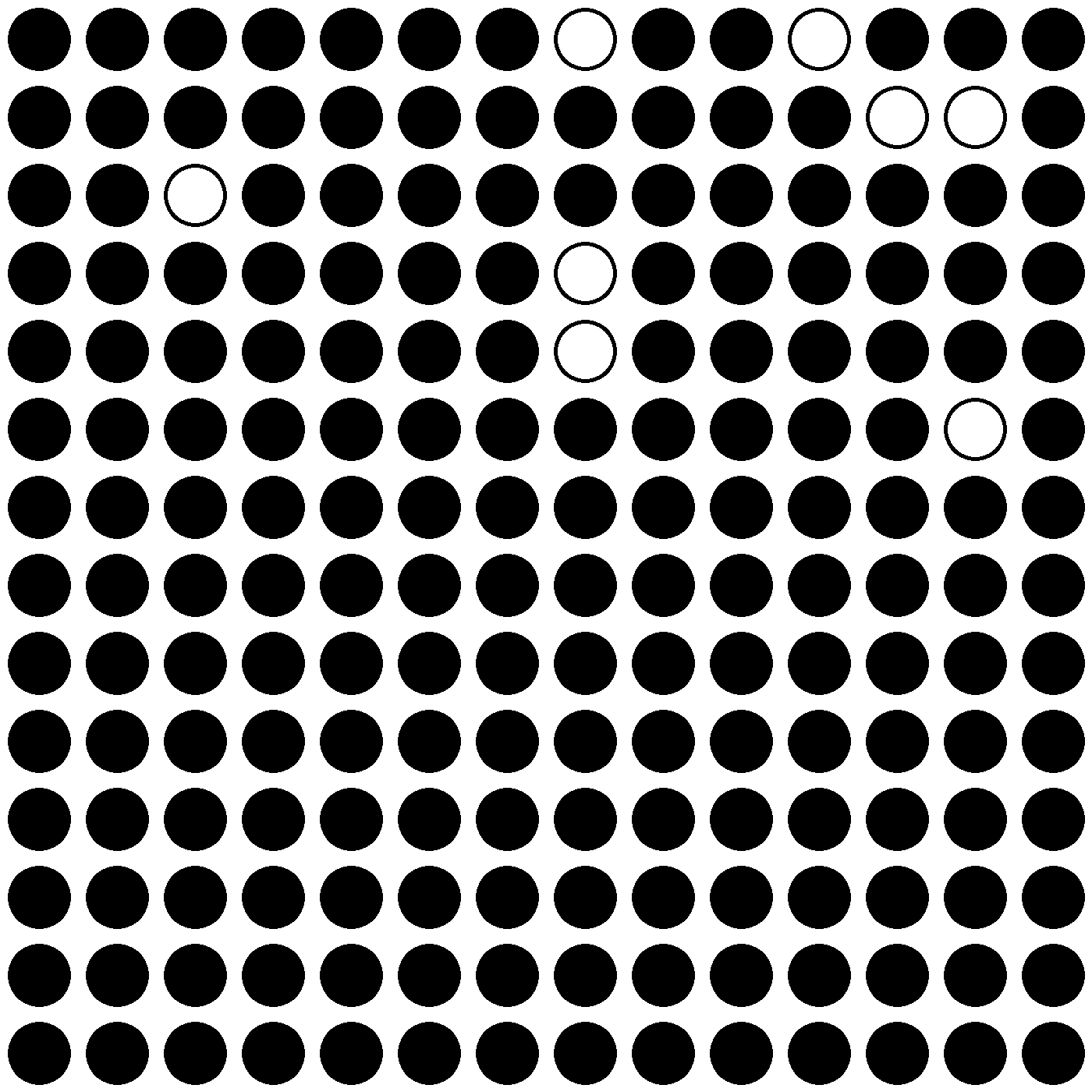


95.9% (14)


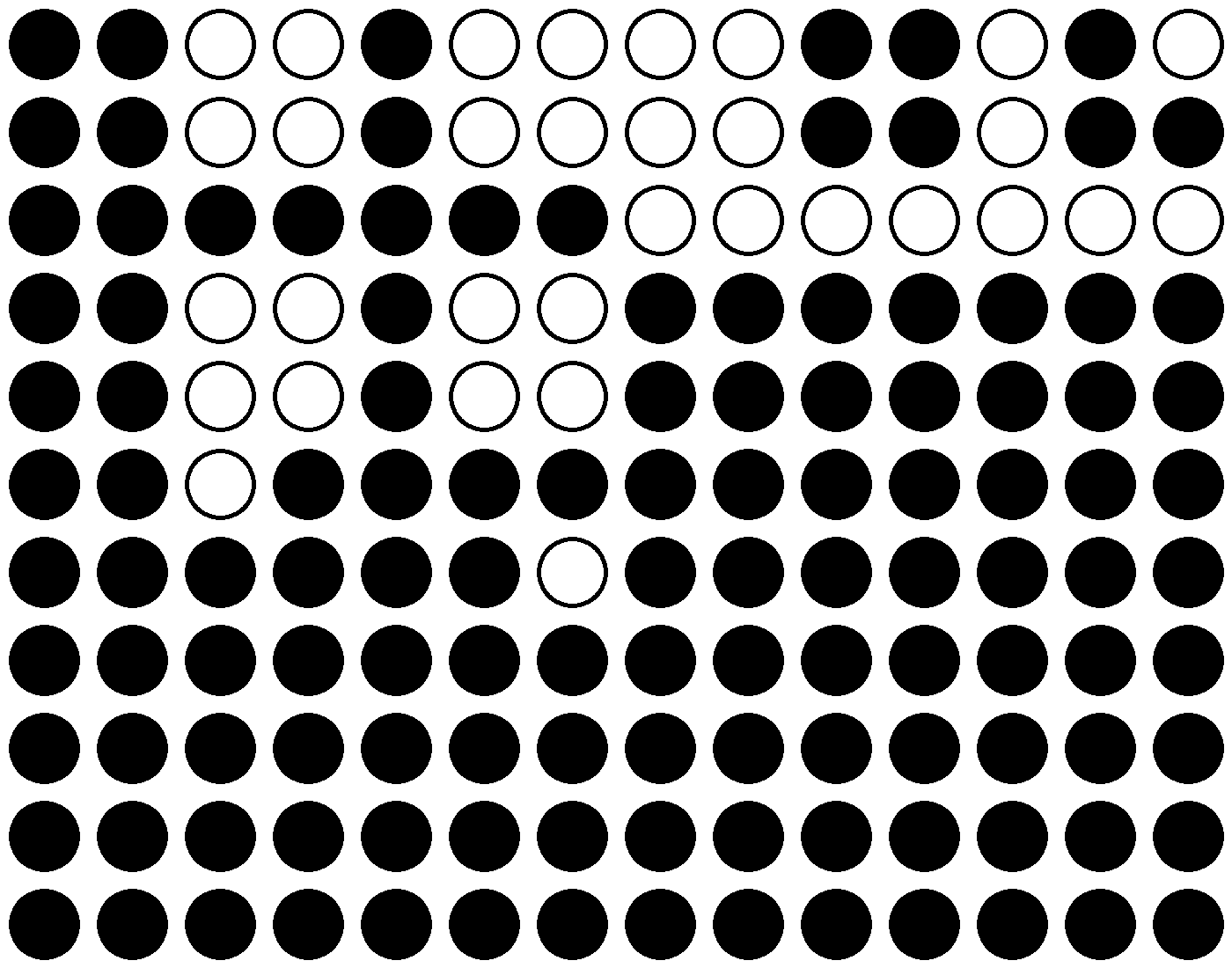


79.2% (11)


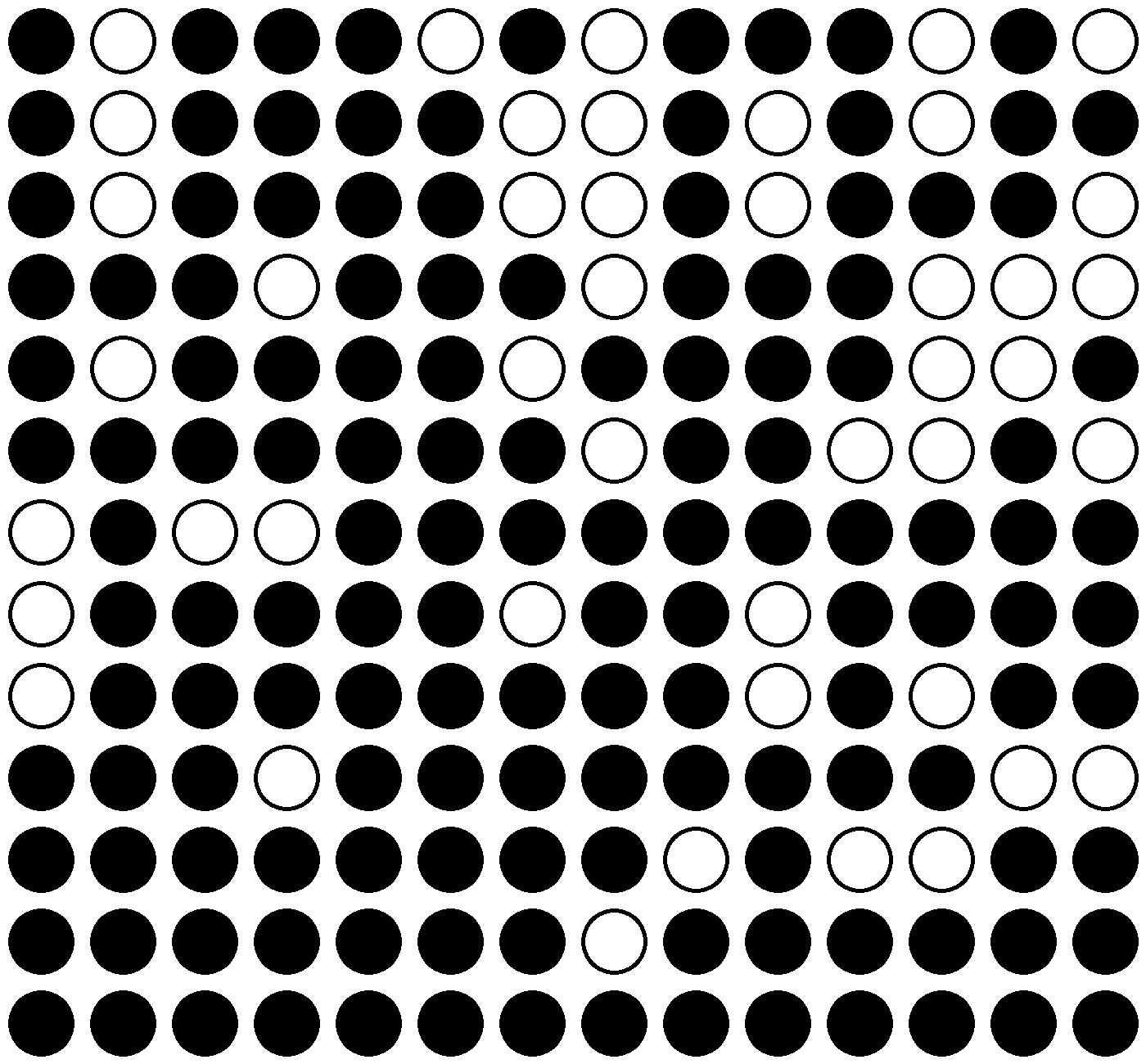


75.8% (13)


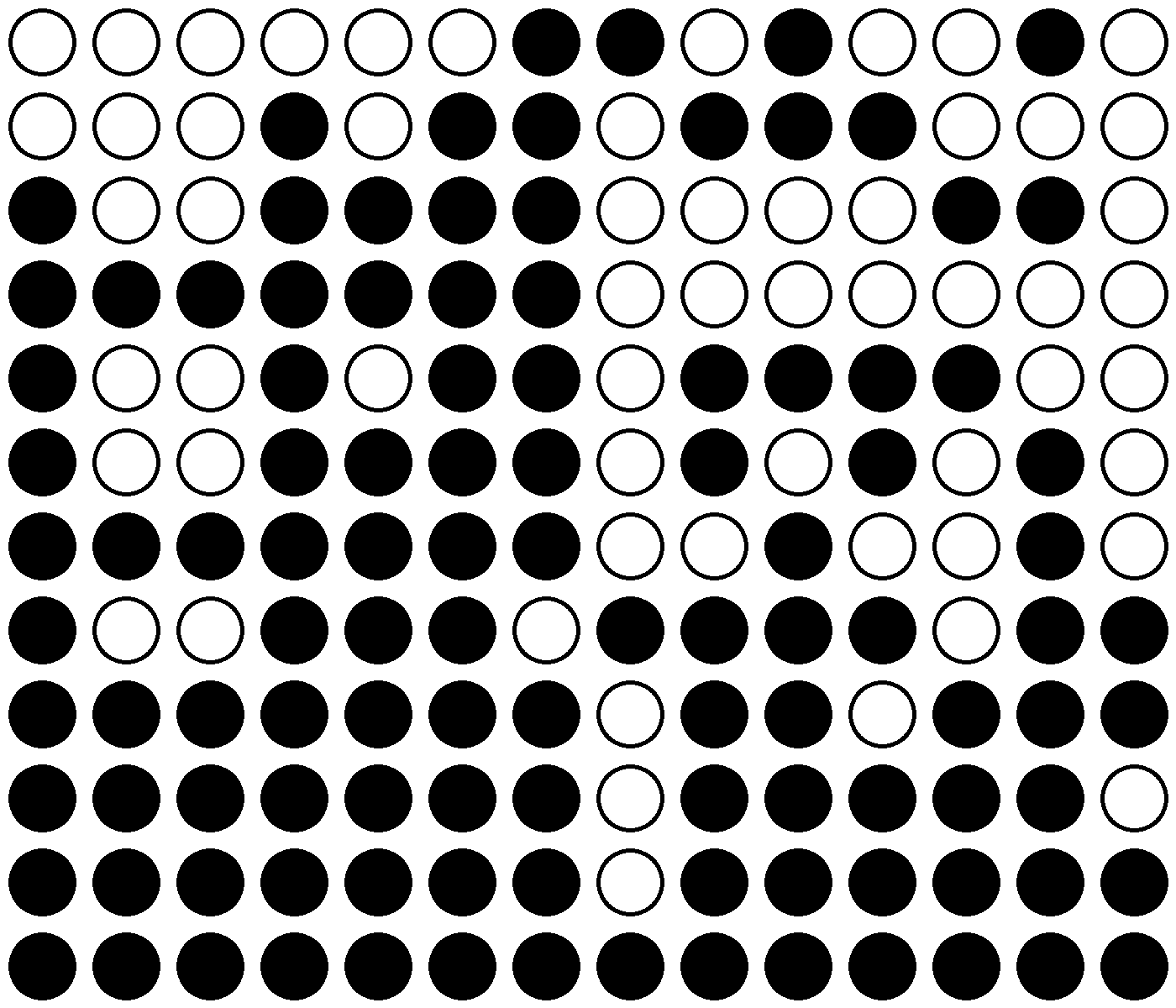


65.5% (12)


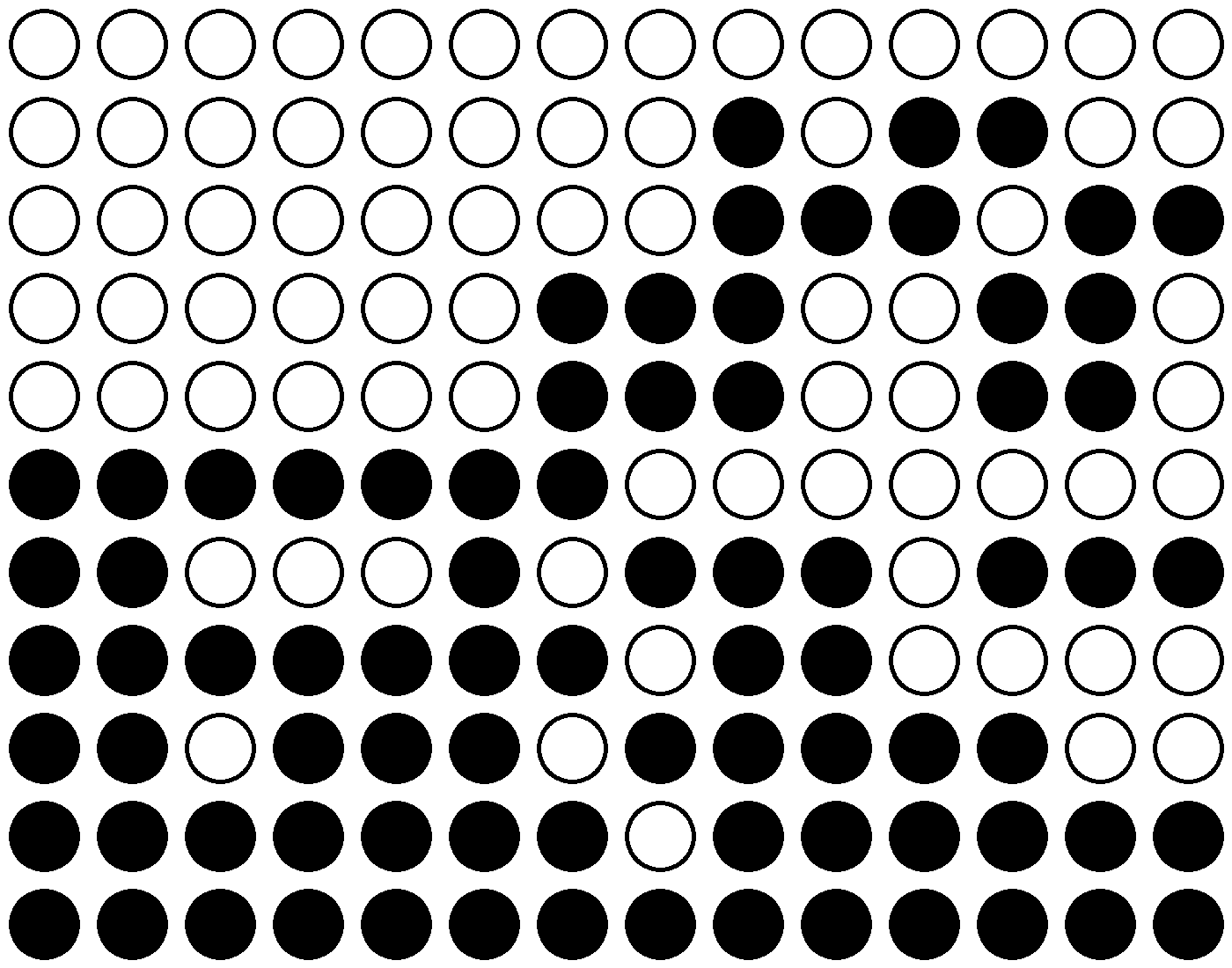


51.9% (11)


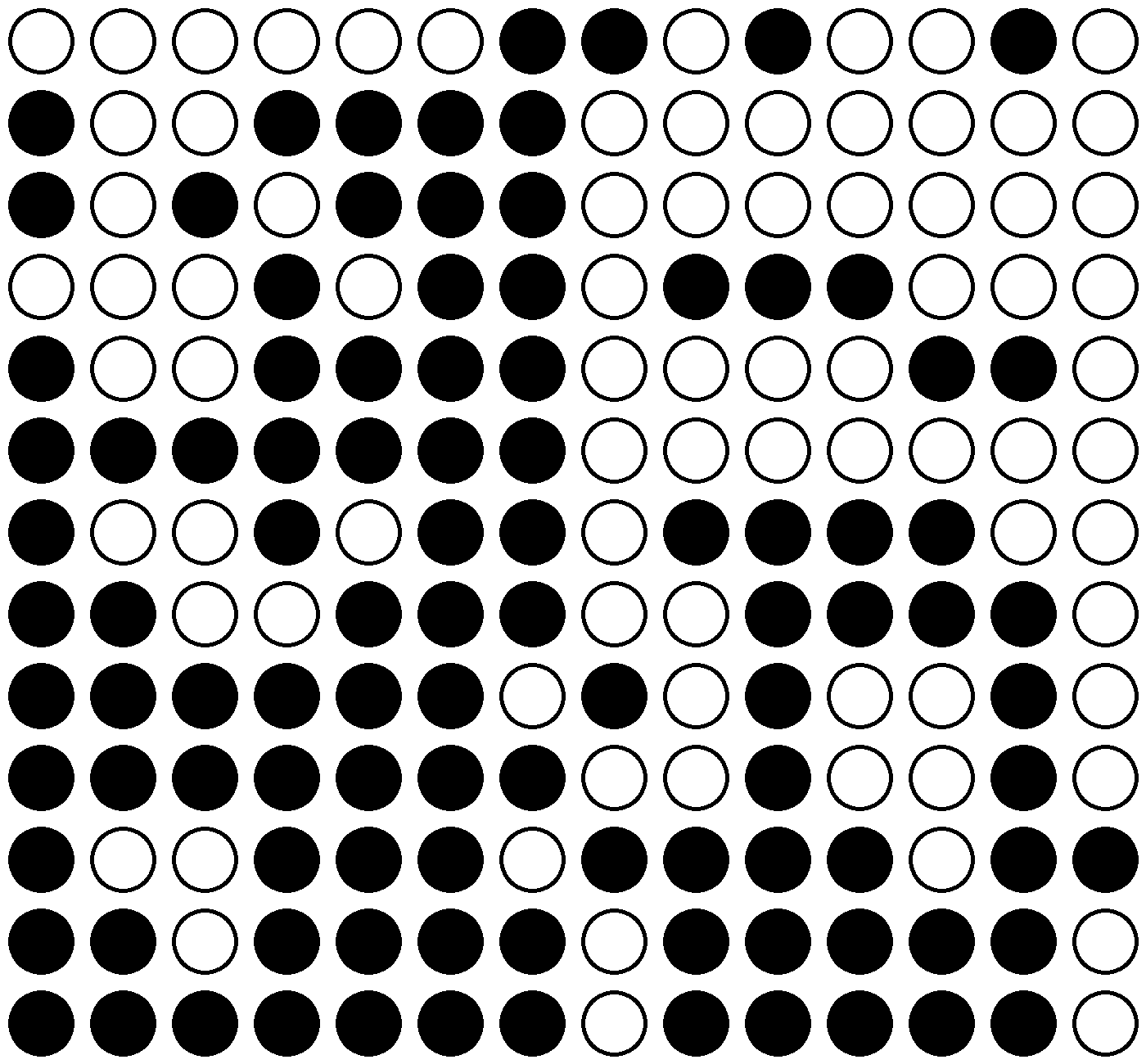


56% (13)

SI

RS

*Adora2a*-1

95.2% (13)


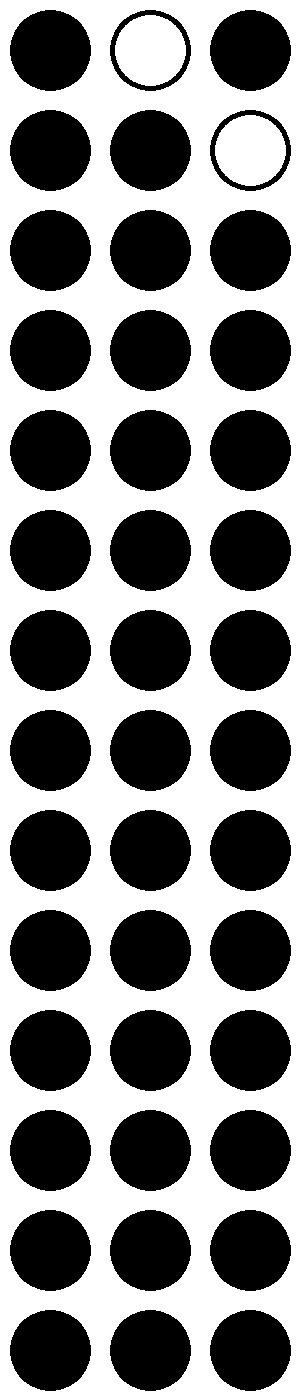

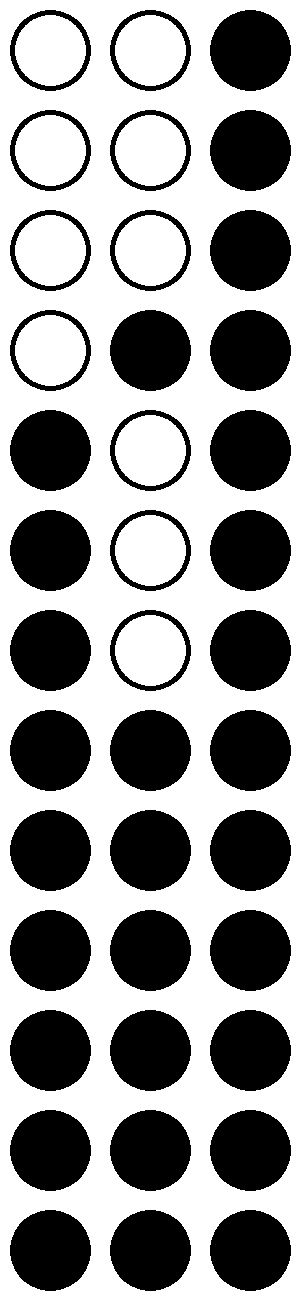

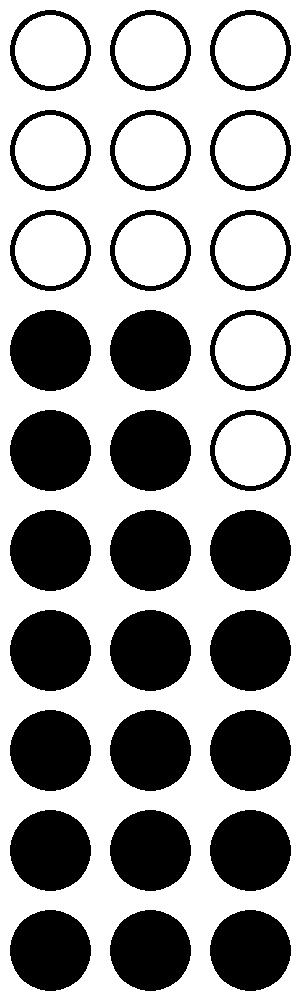

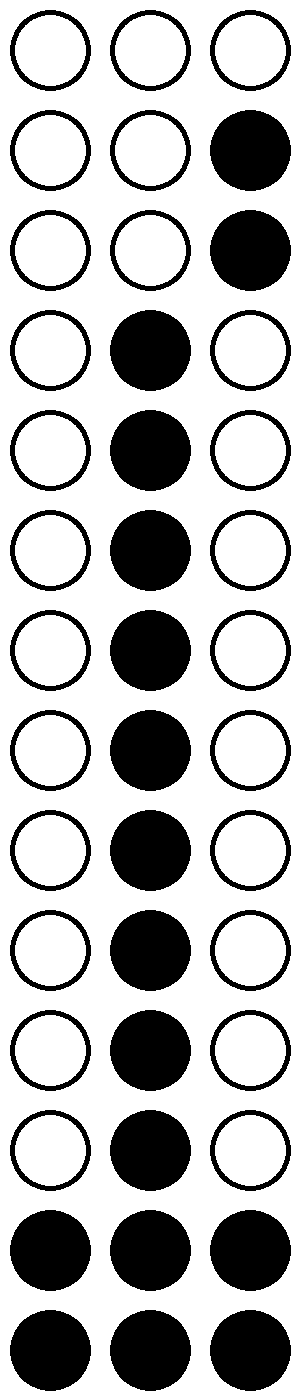

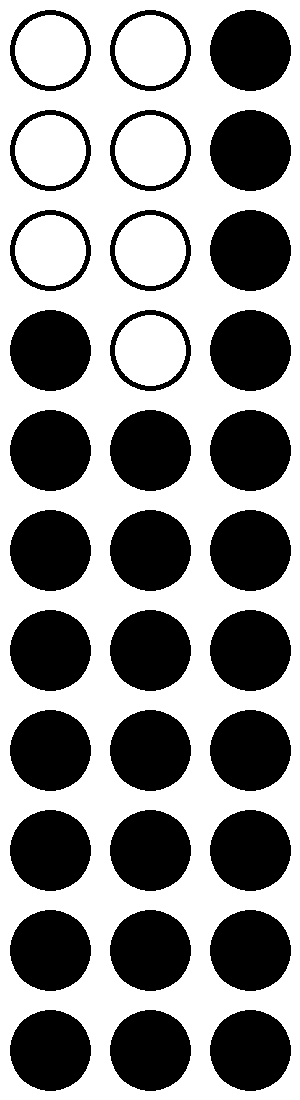

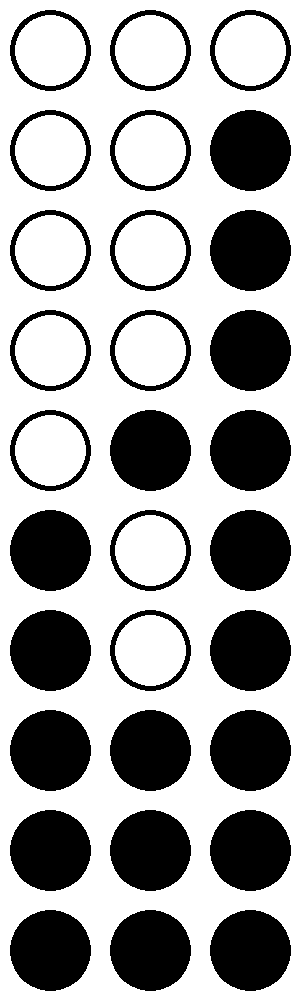


63.3% (11)

77.8% (11)

74% (13)

40.5% (14)

60% (10)

SI

RS

*Adora2a*-2


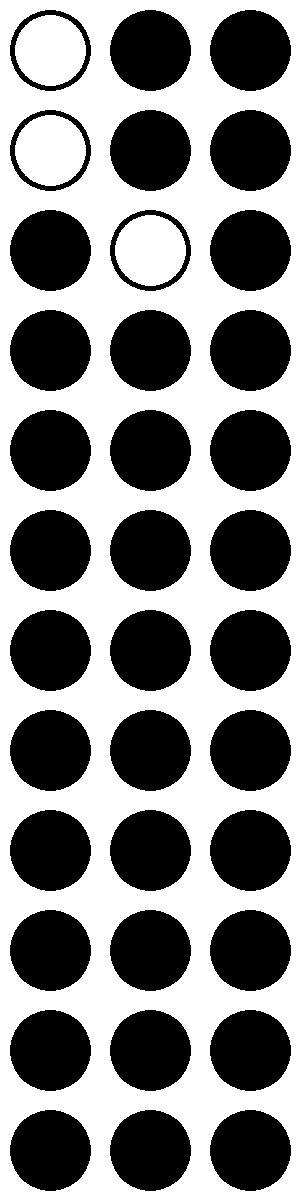

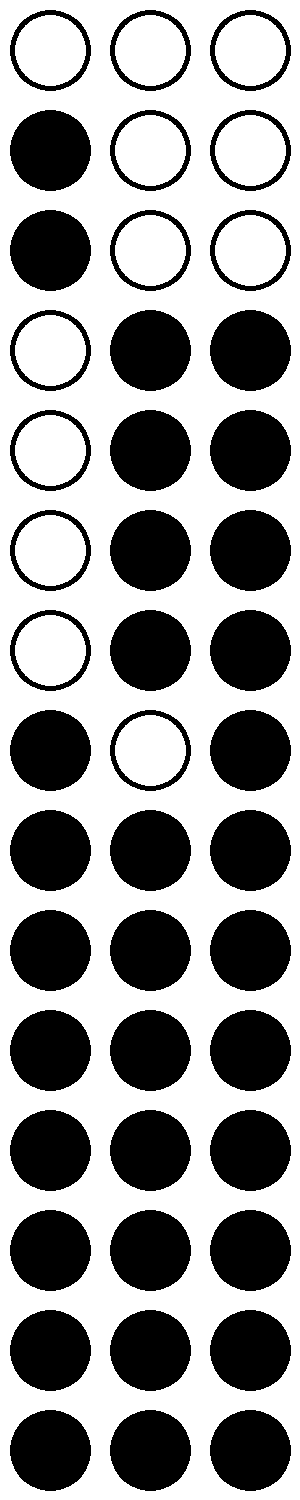

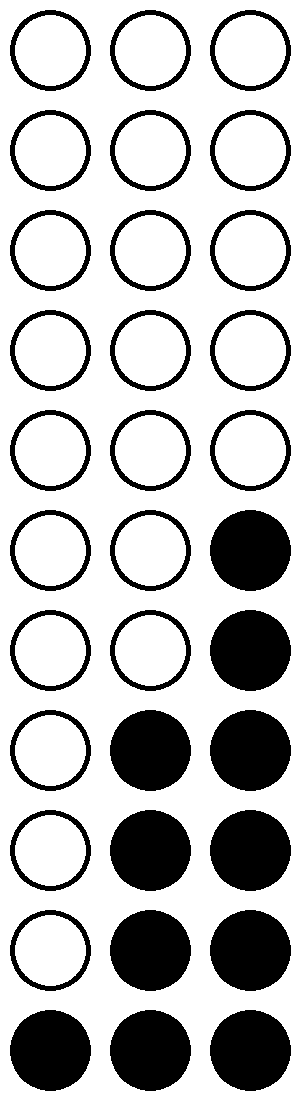

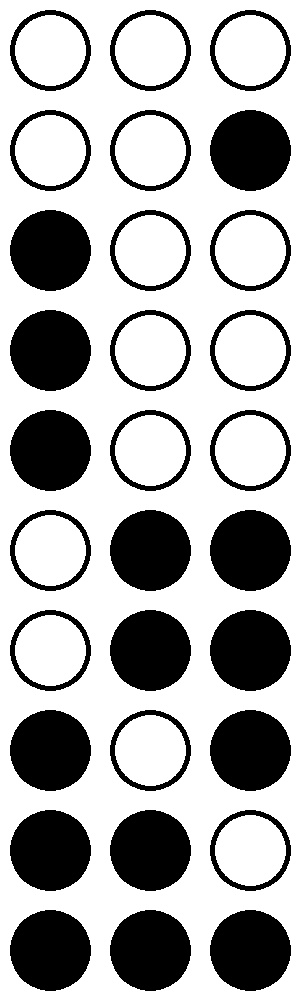

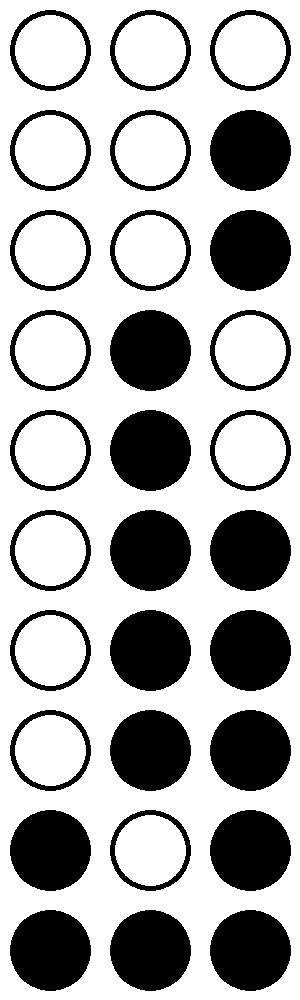

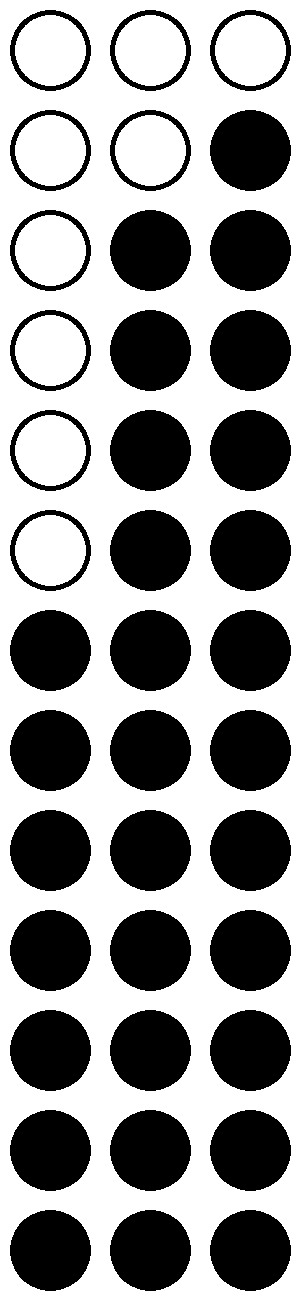


73.2% (15)

33.3% (11)

50% (11)

91.7% (12)

50% (10)

76.9% (13)

SI

RS

*Adcy9*


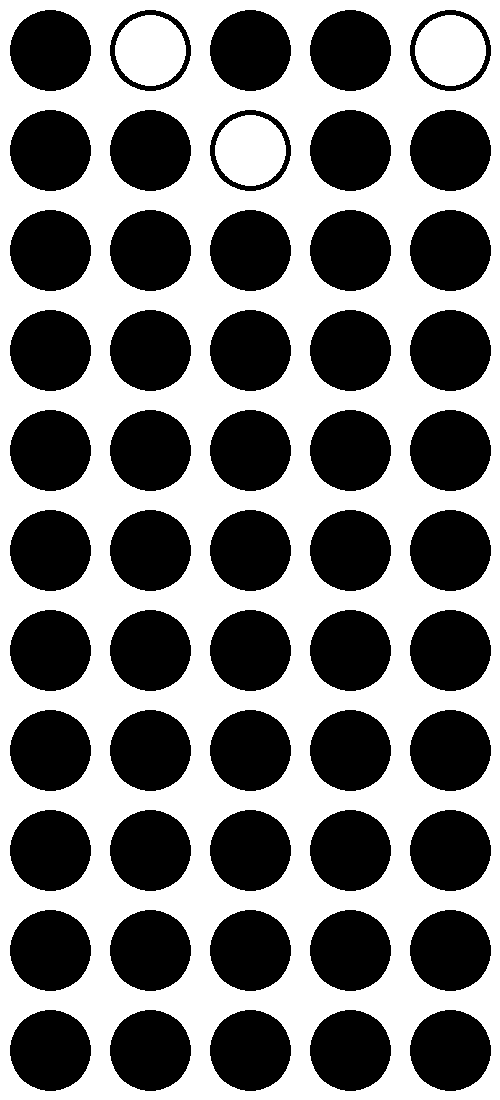

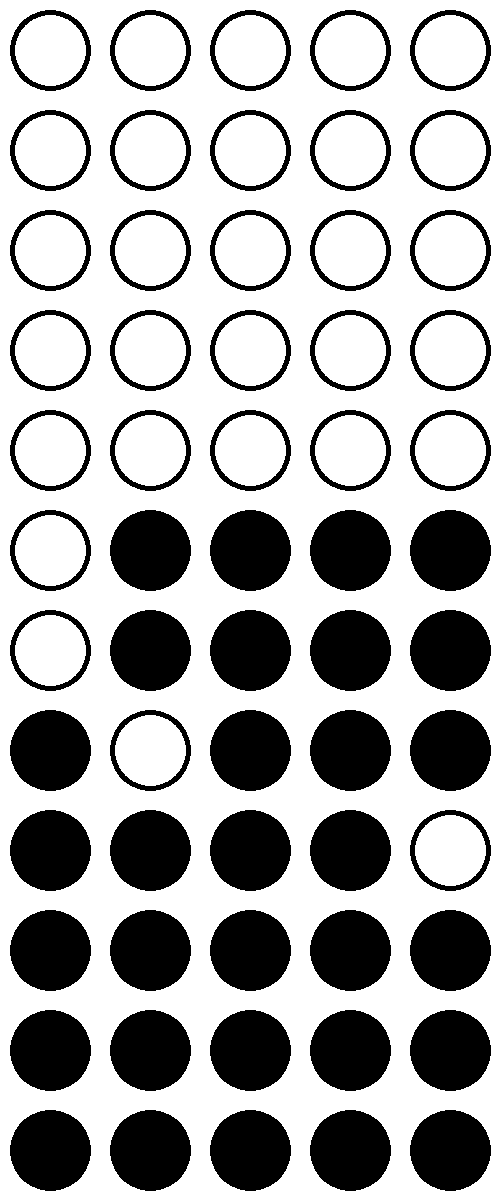


9.2% (13)

41.5% (13)

94% (11)

51.7% (12)


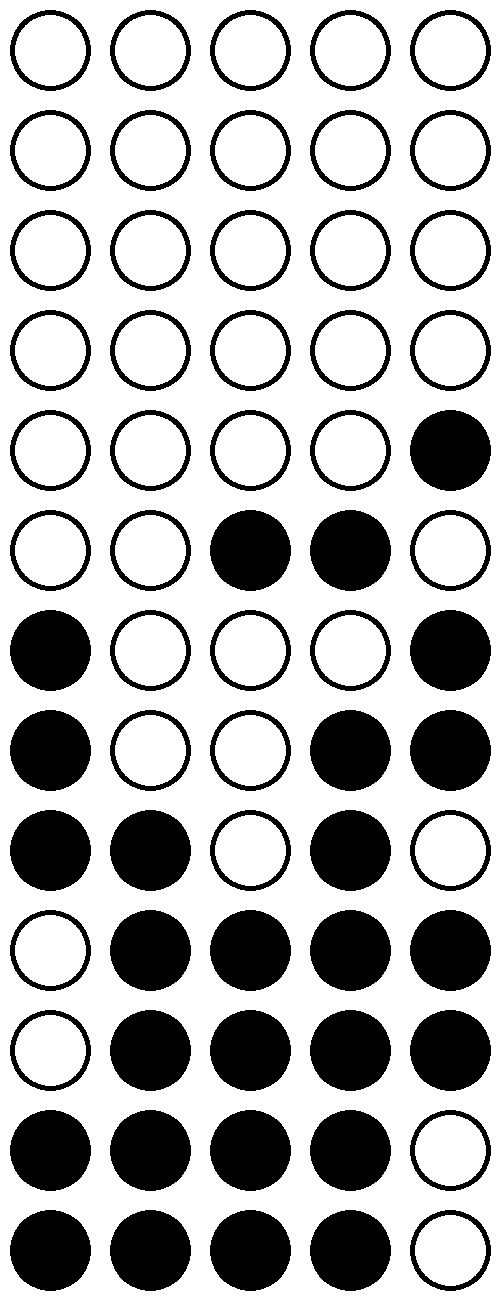

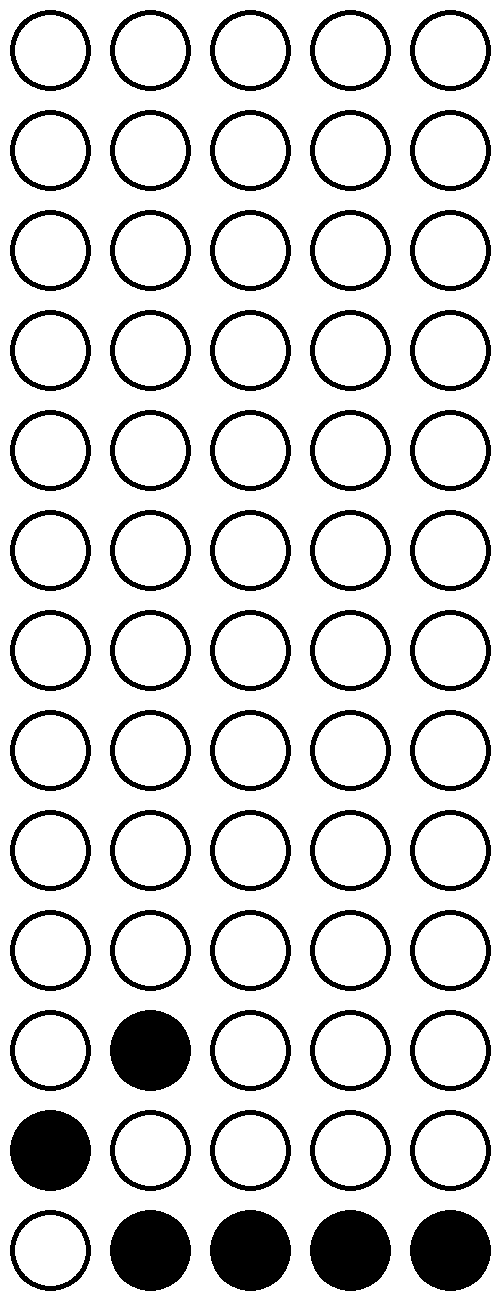

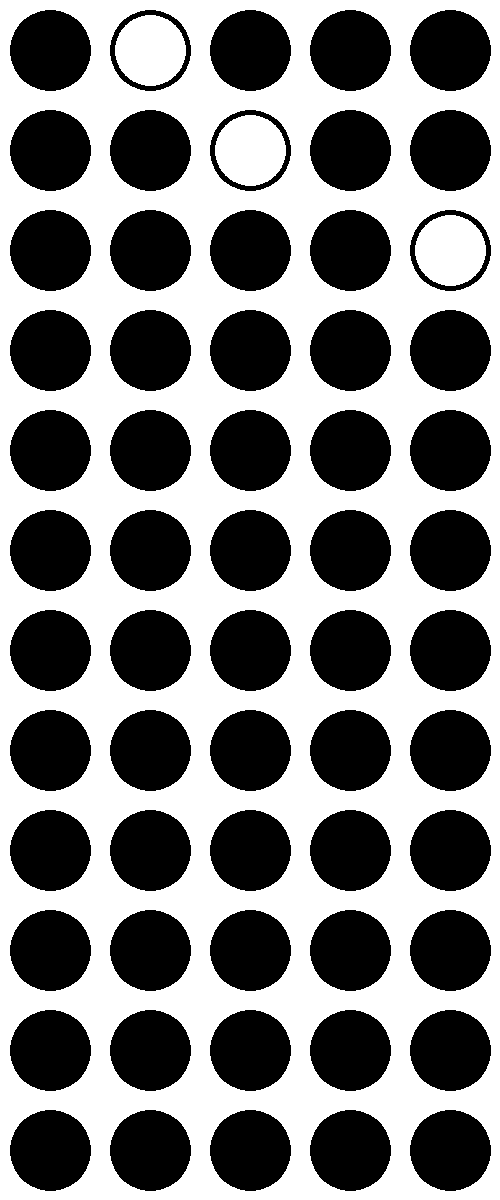

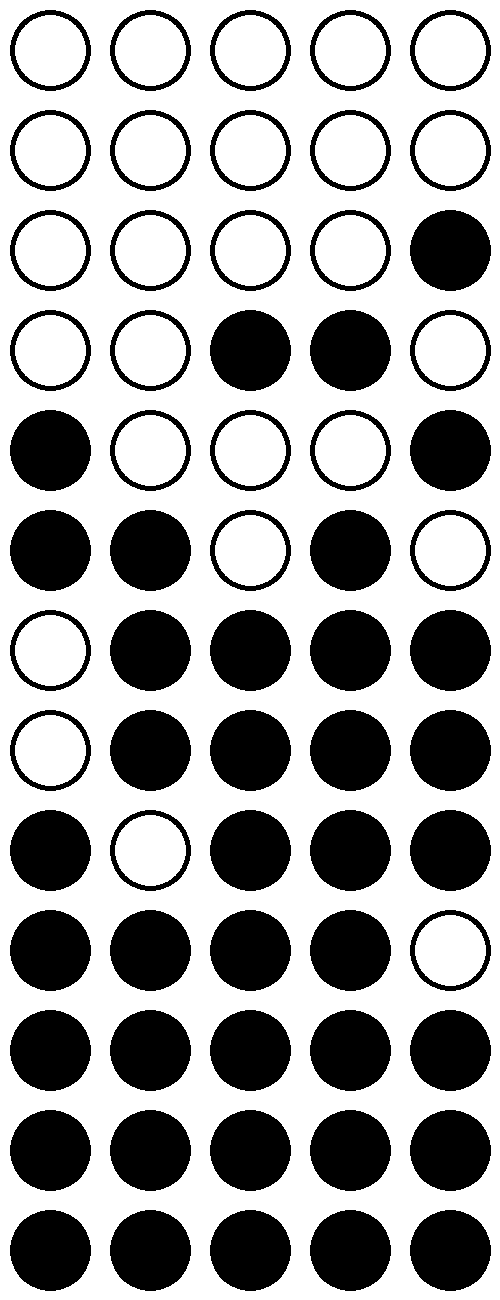


60% (13)

95% (12)

SI

RS

*Bdnf*

Spermatozoa

**Figure S1-2**

Blastocysts

Fetal hippocampi


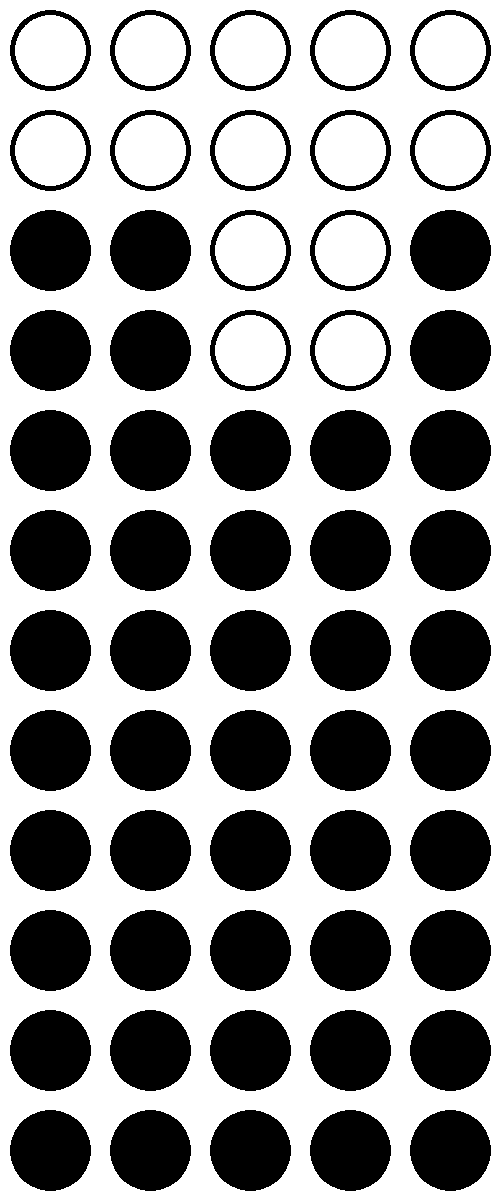

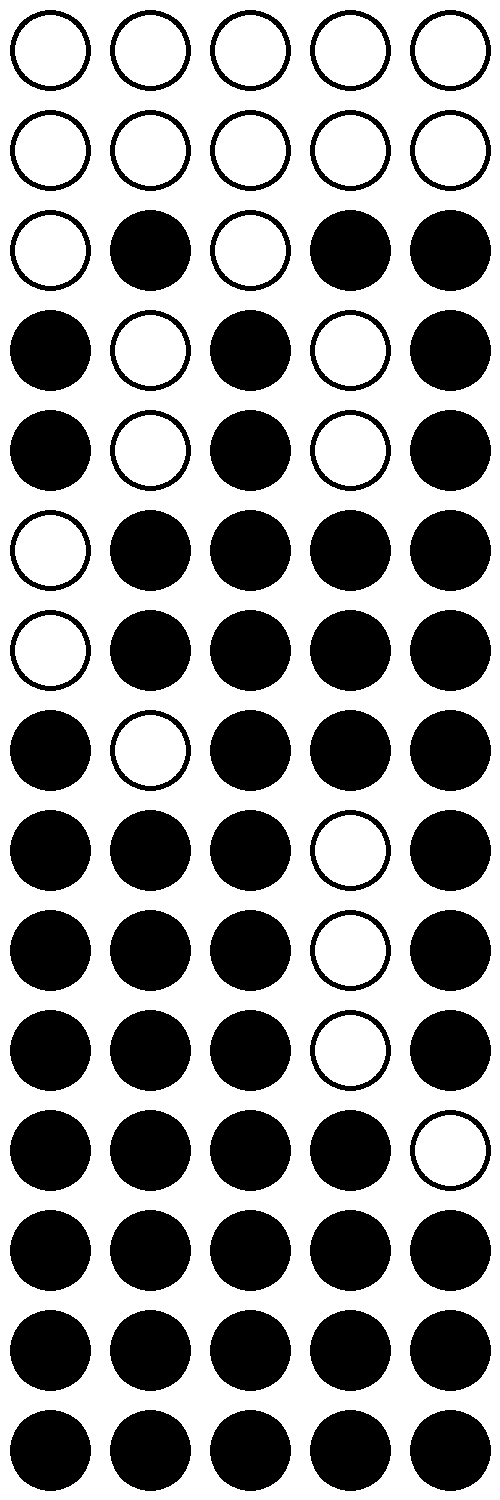

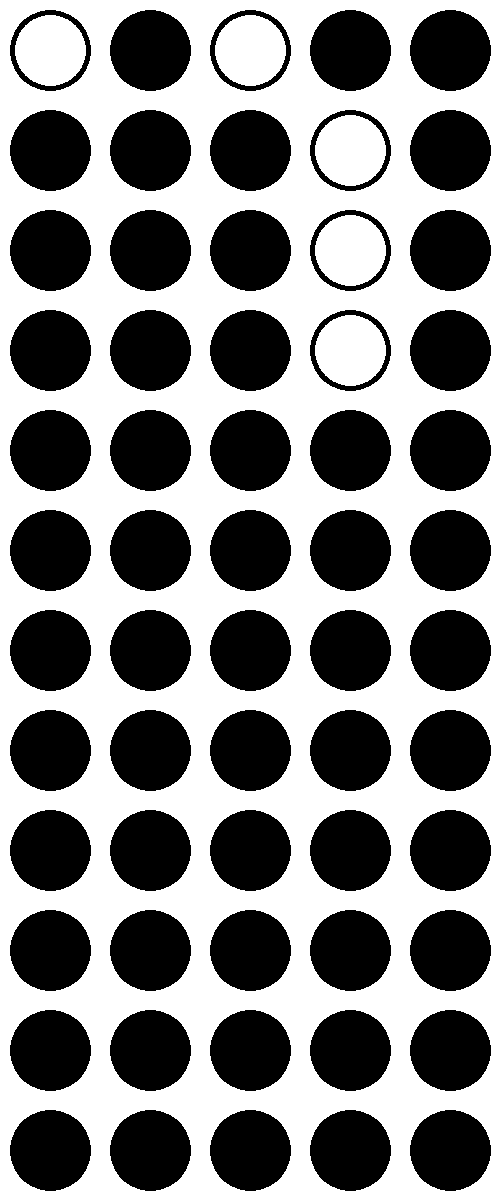

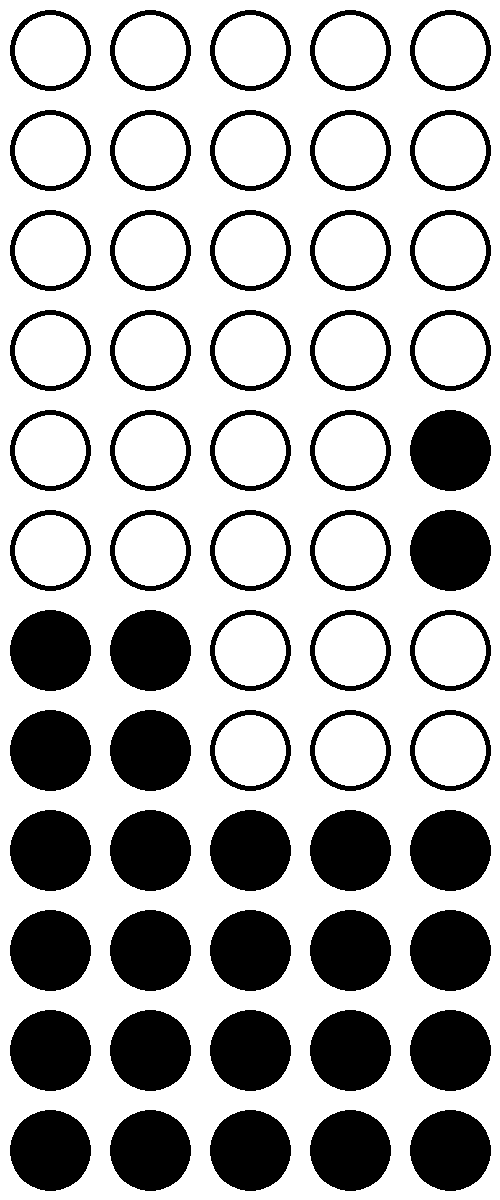

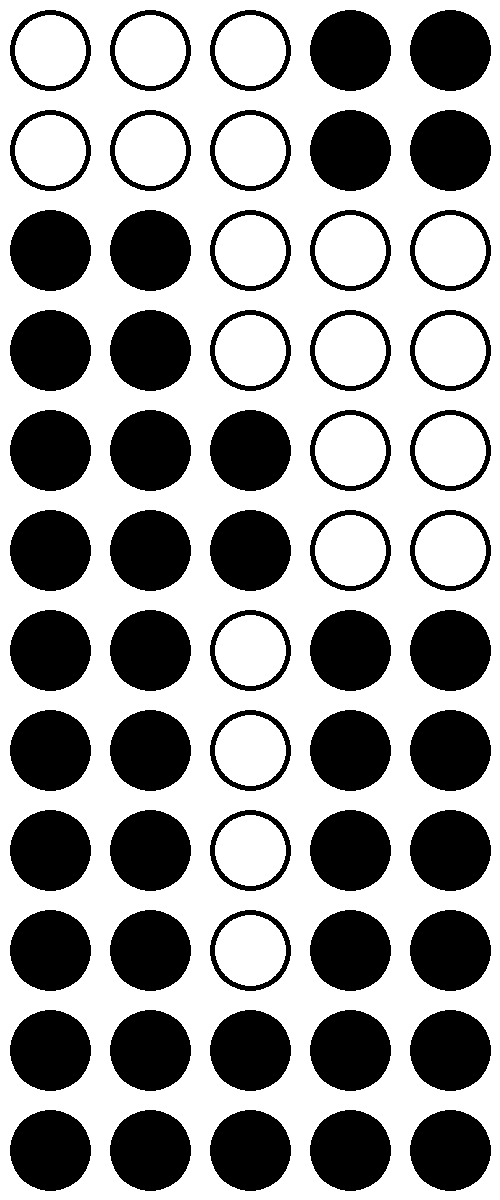

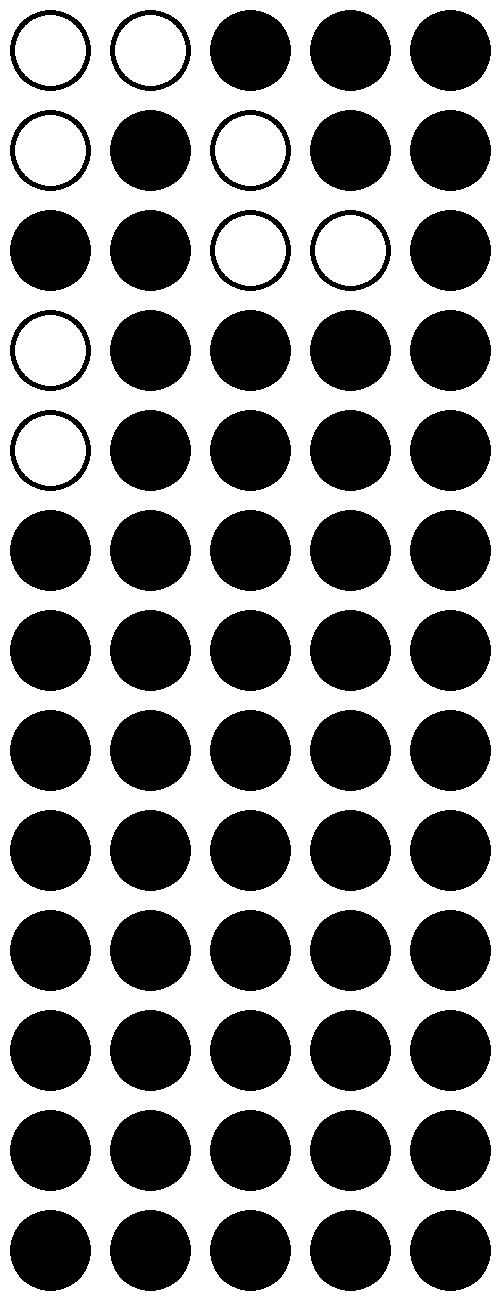


69.3% (15)

43.3% (12)

66.7% (12)

91.7% (12)

76.7% (12)

87.7% (13)

SI

RS

*Itpr3*


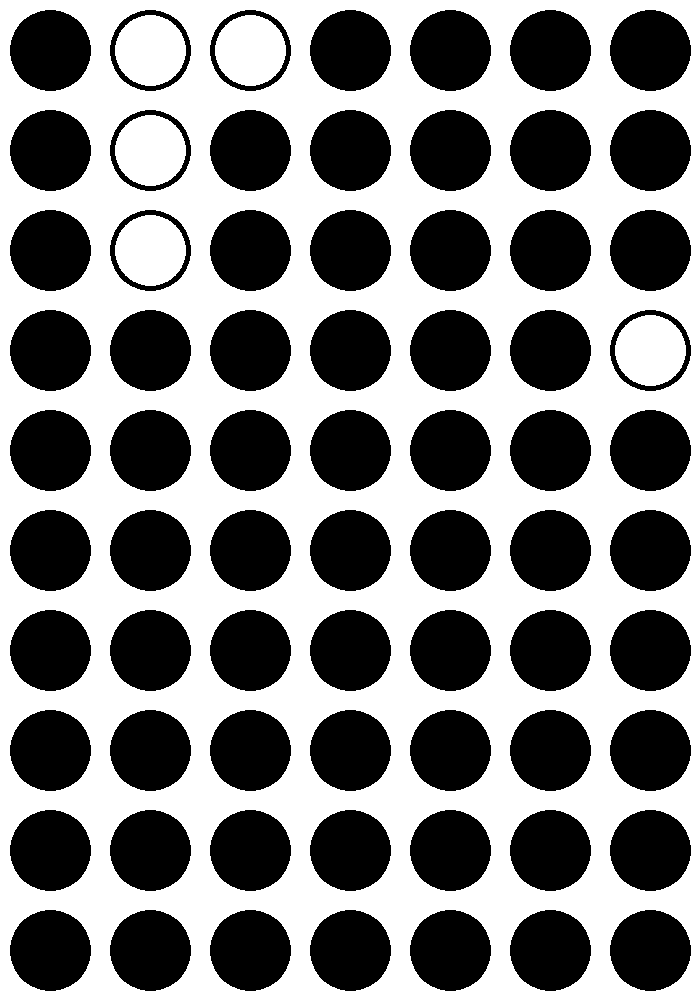

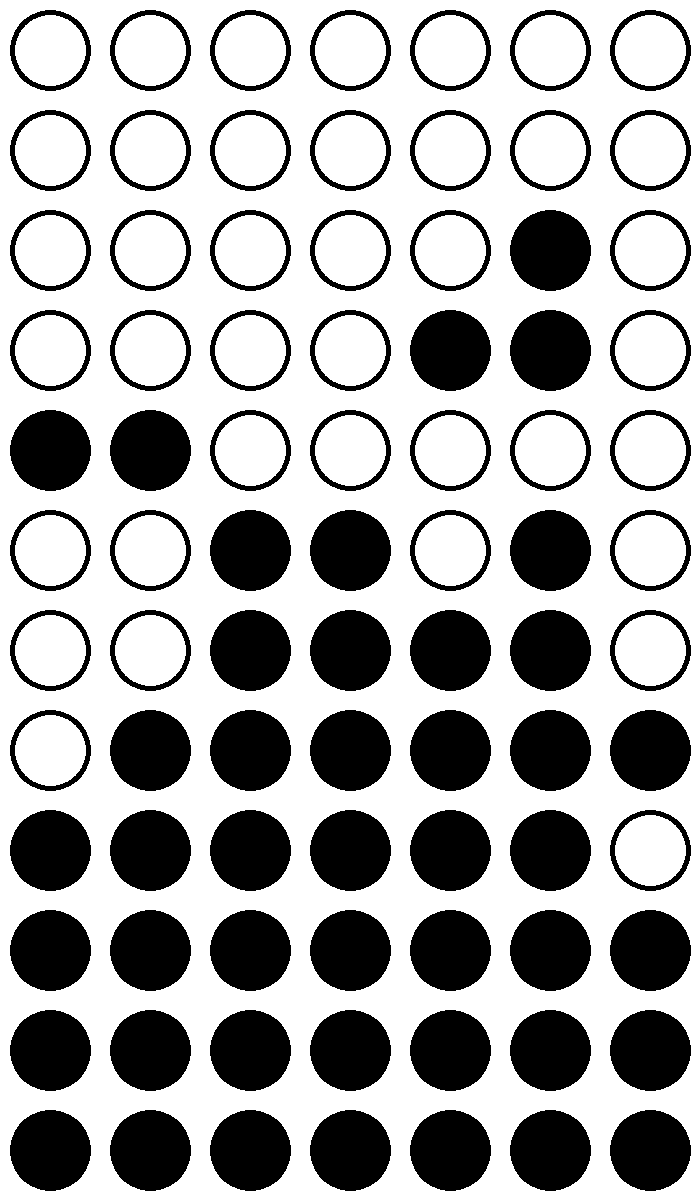


53.6% (12)

92.9% (10)

10.2% (14)

40% (10)


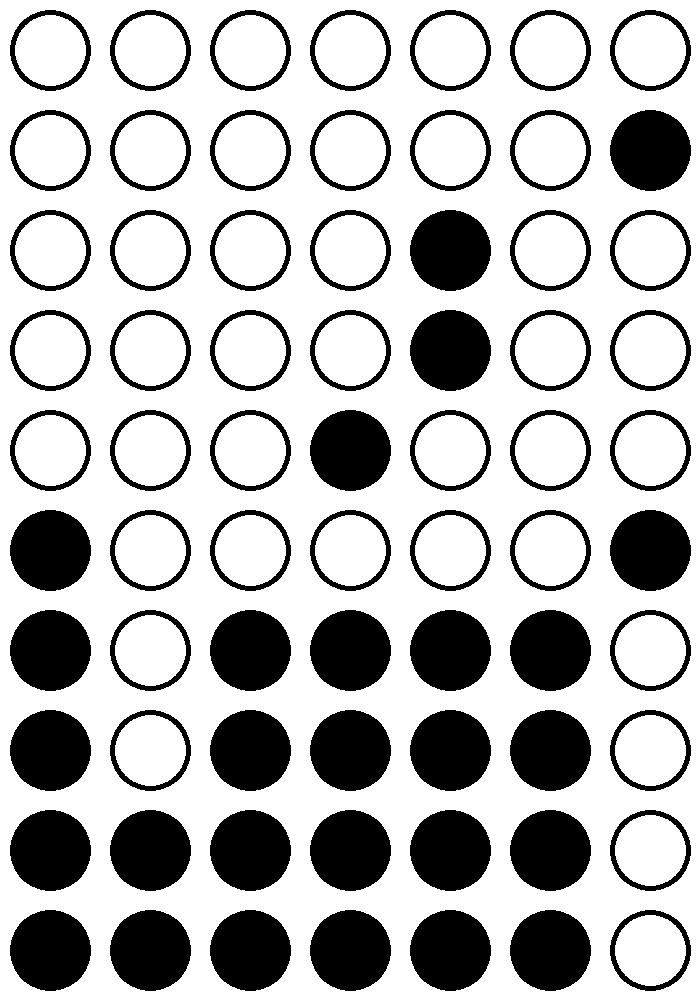

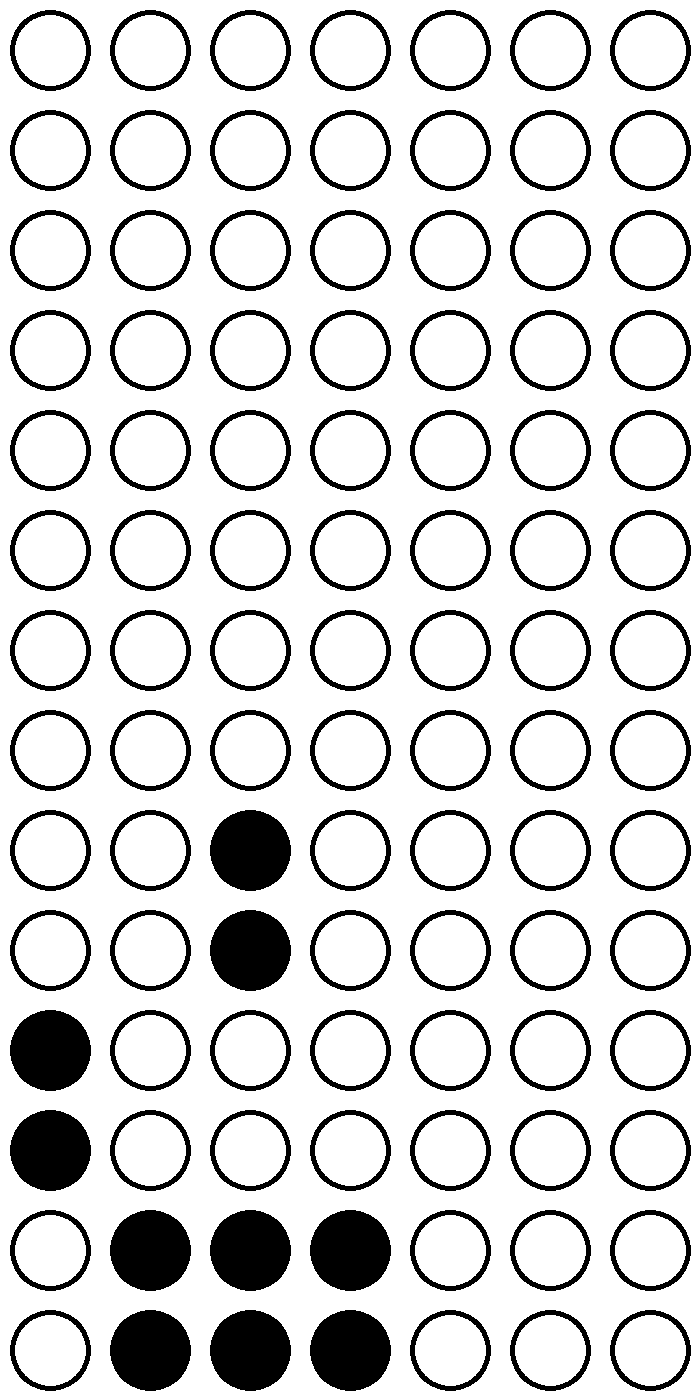


54.1% (14)


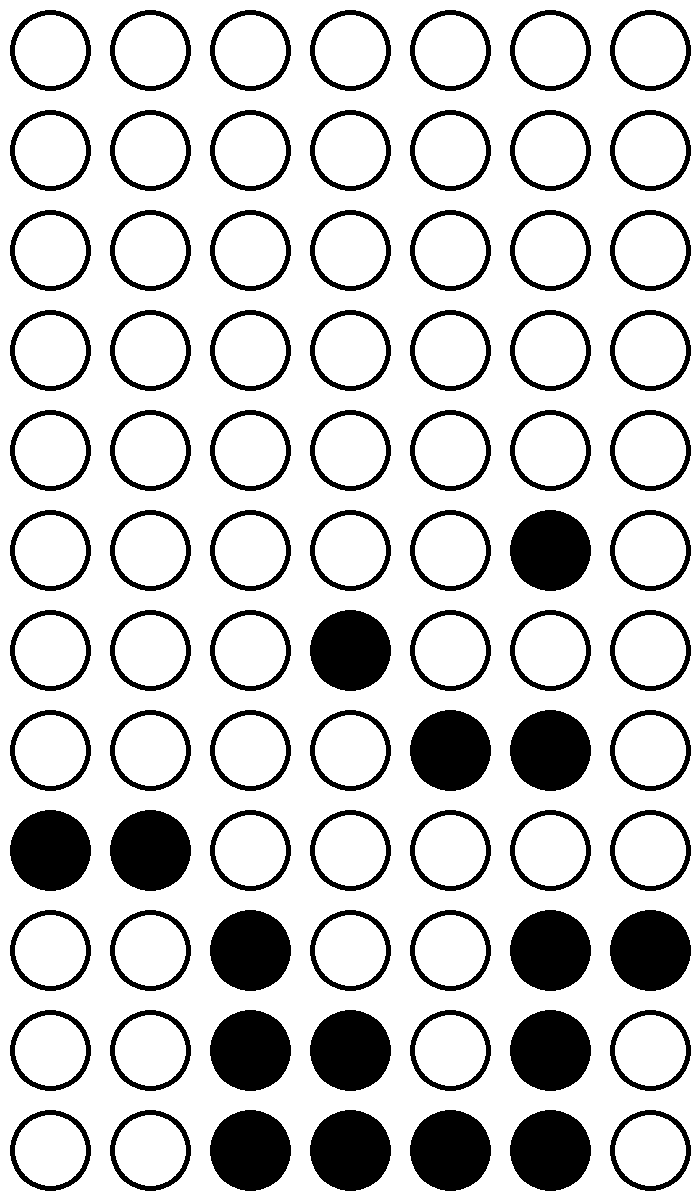

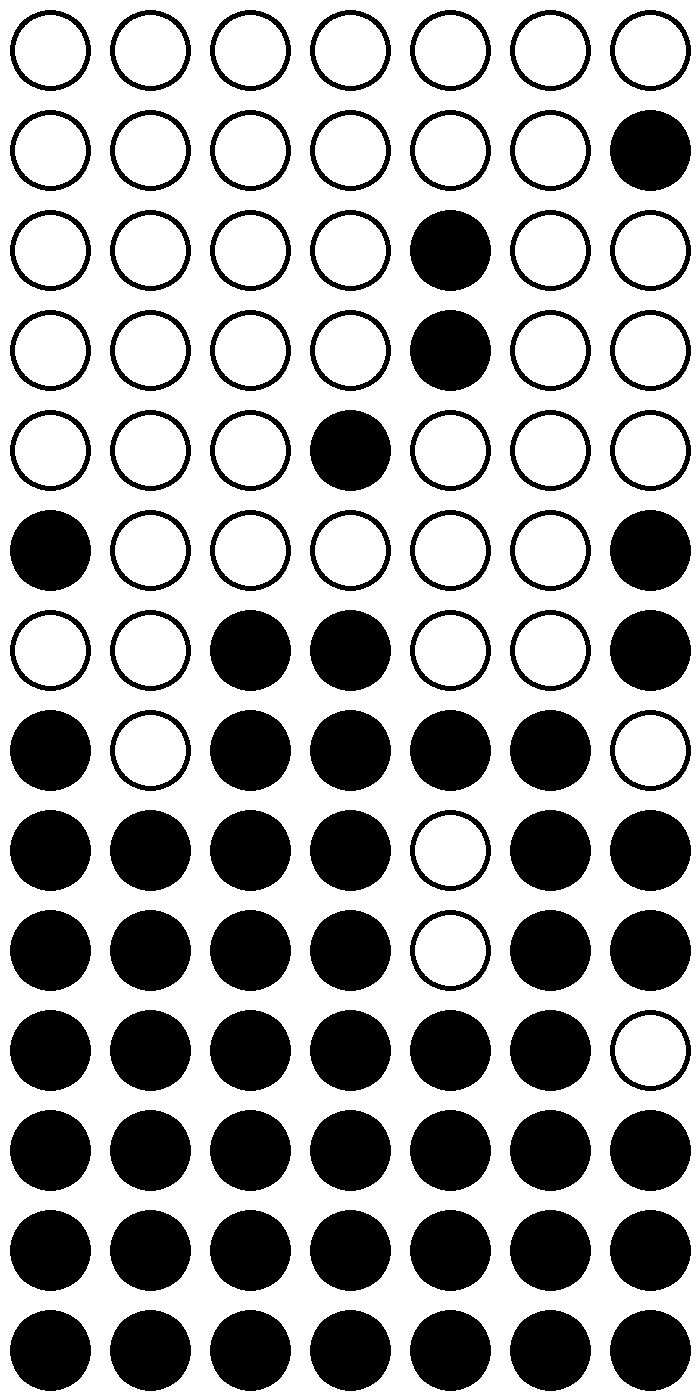


19.1% (12)

SI

RS

*Gata2-1*


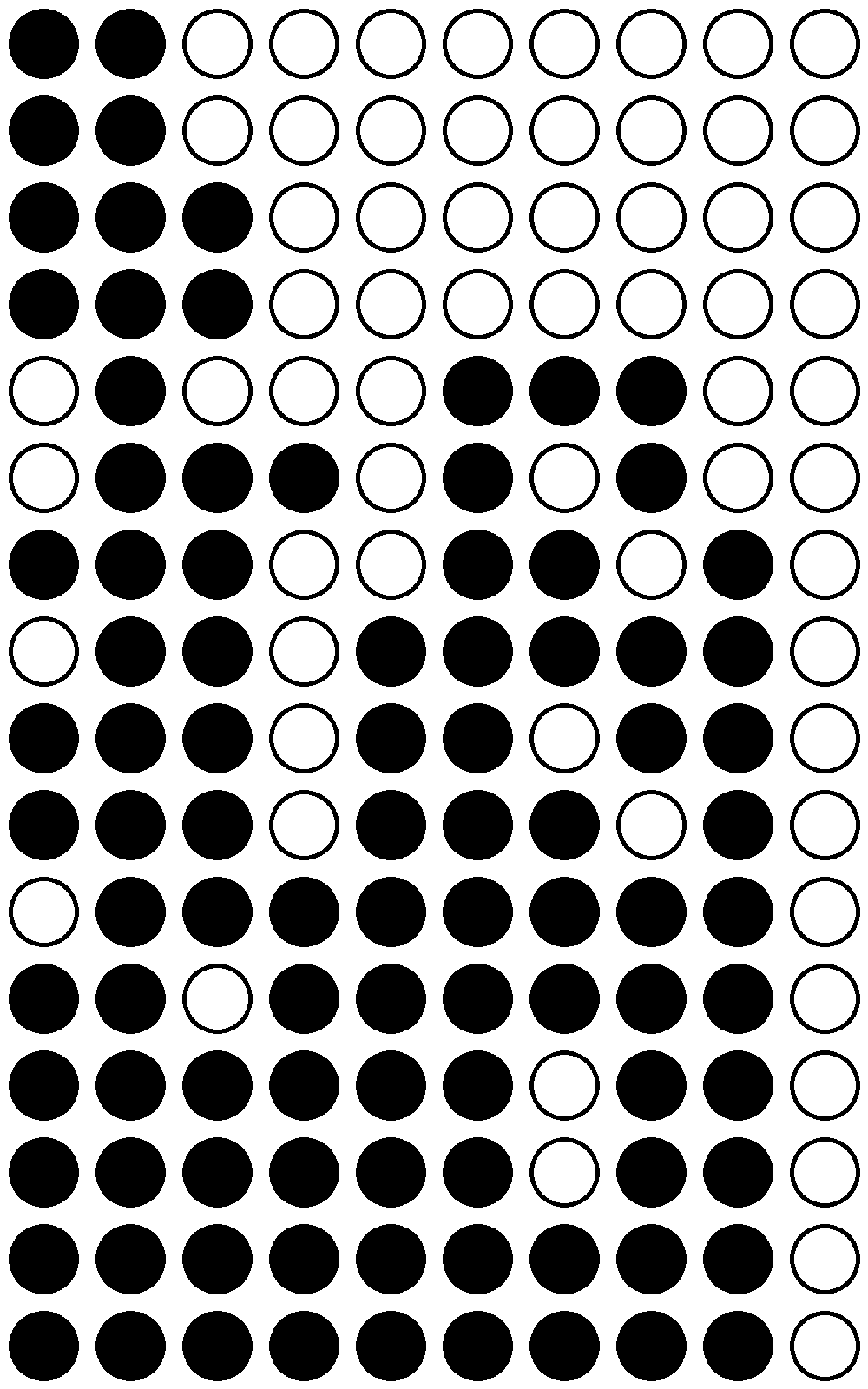


60% (16)

76.7% (12)


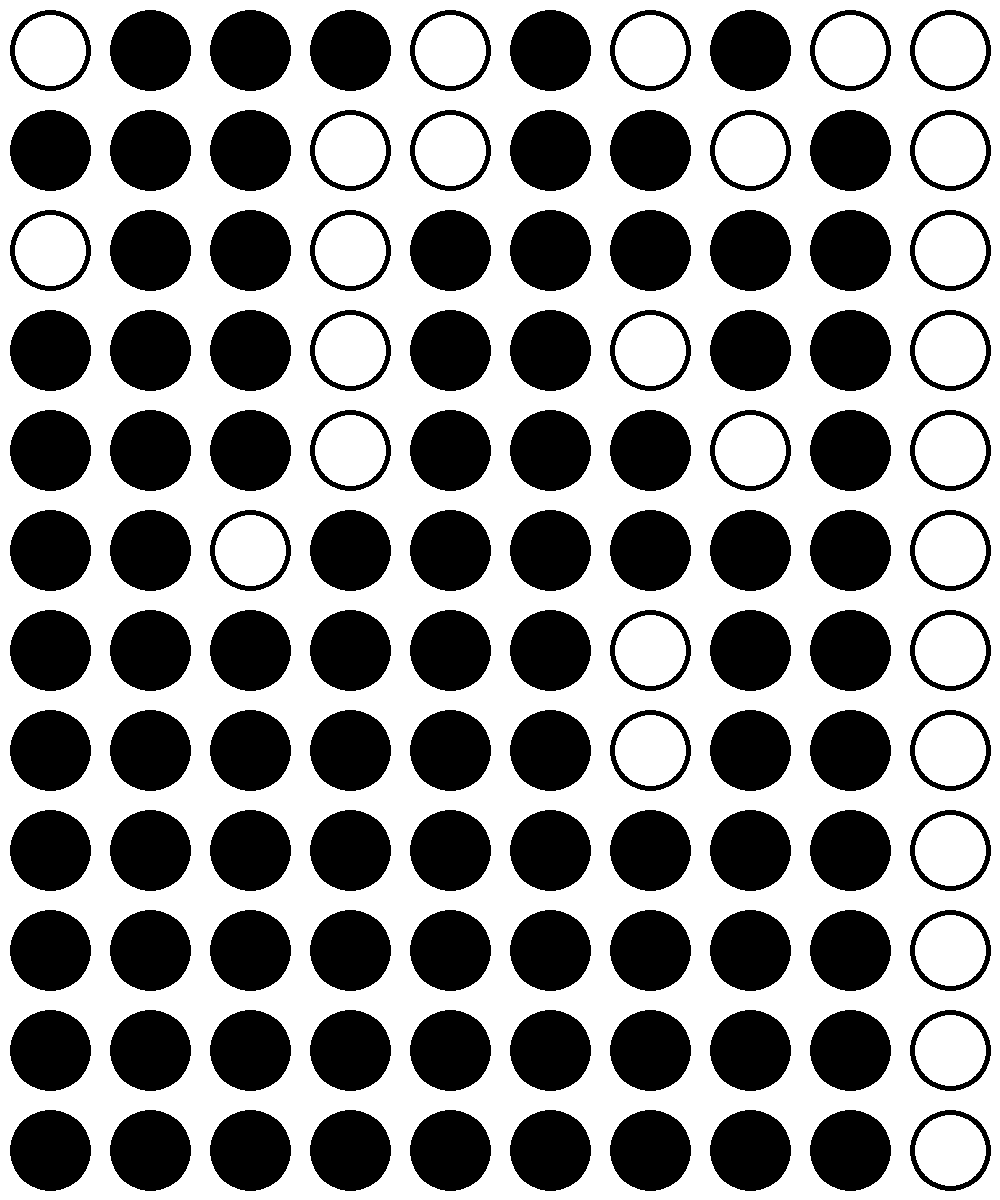


21.7% (12)

4.3% (13)


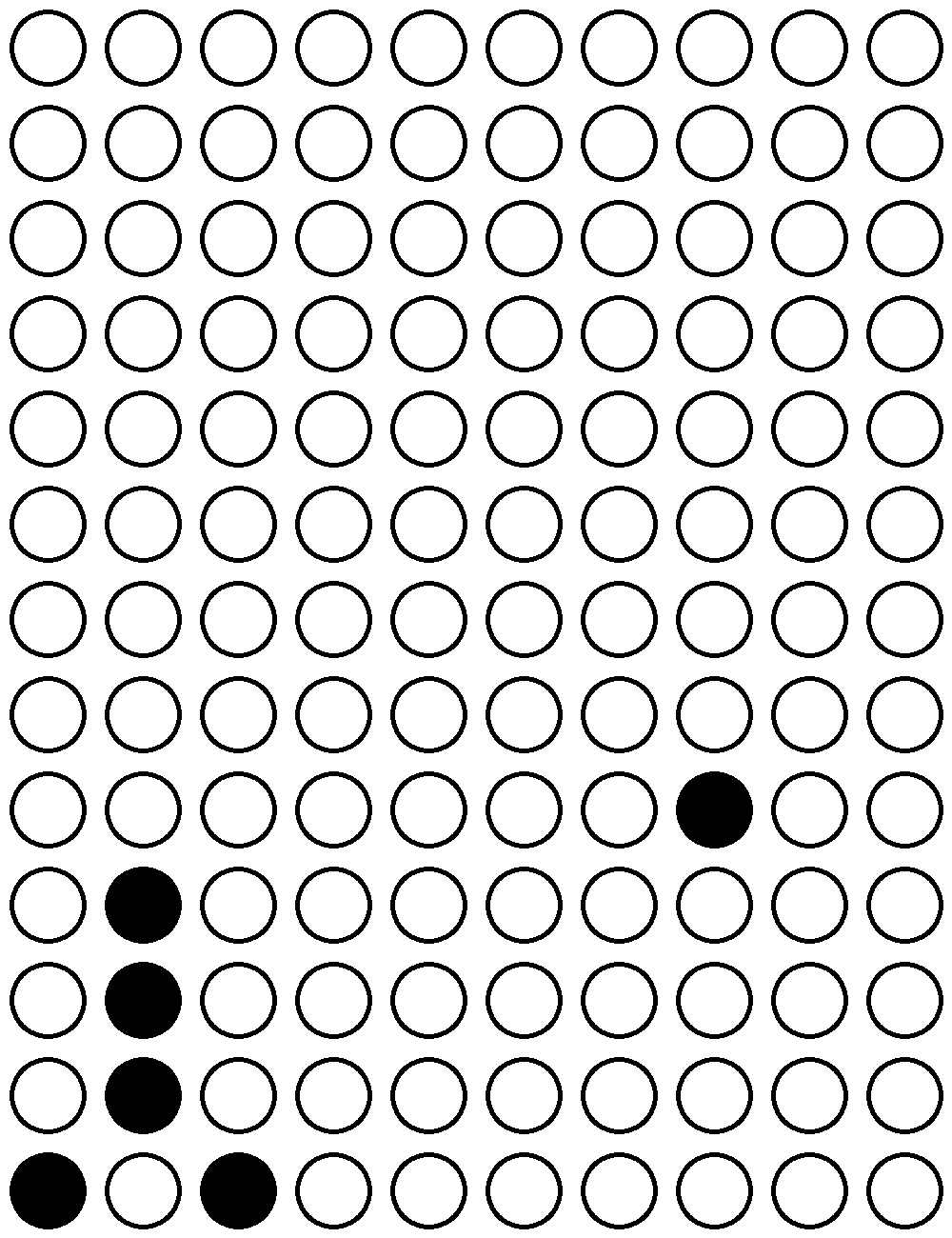

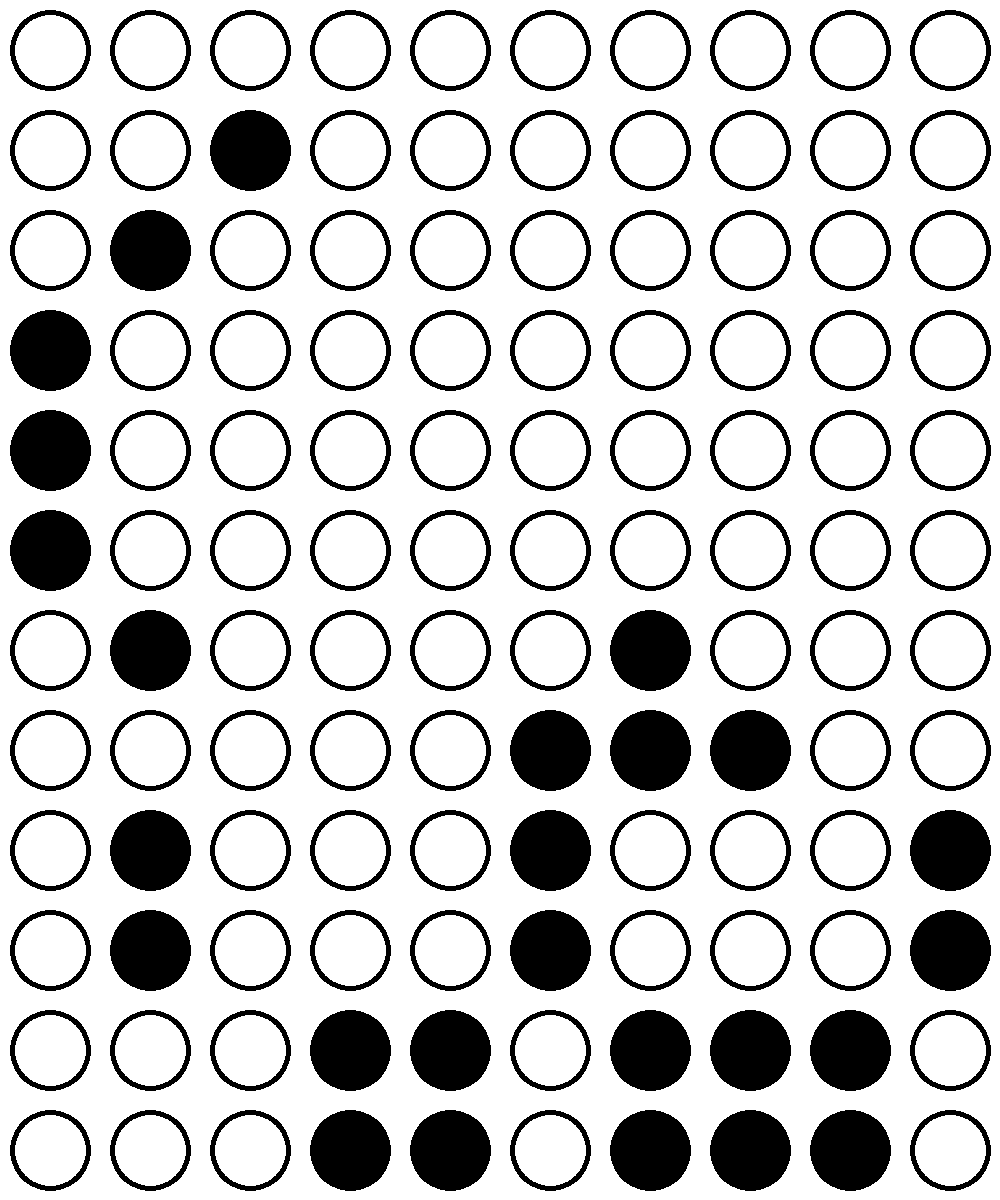

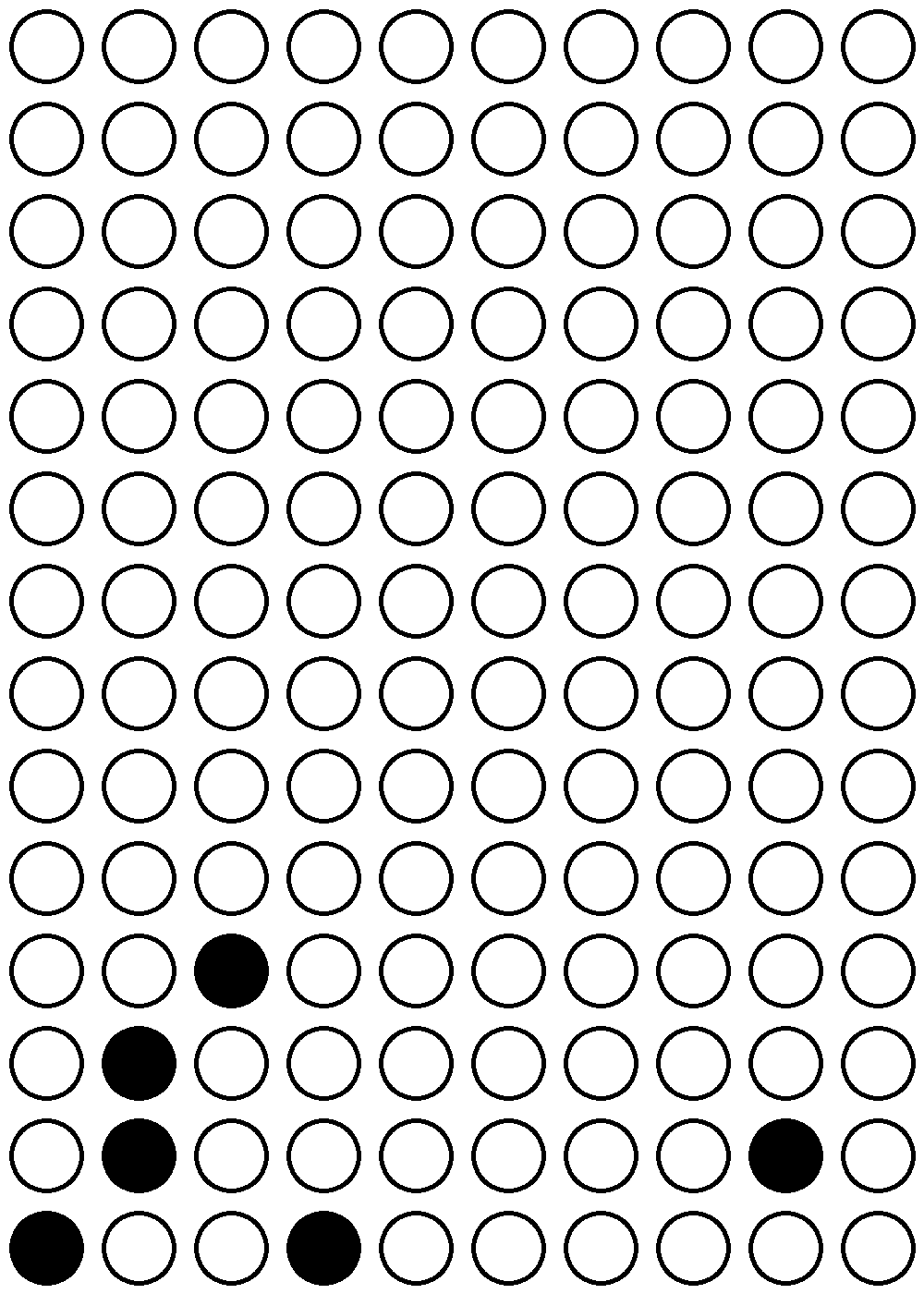

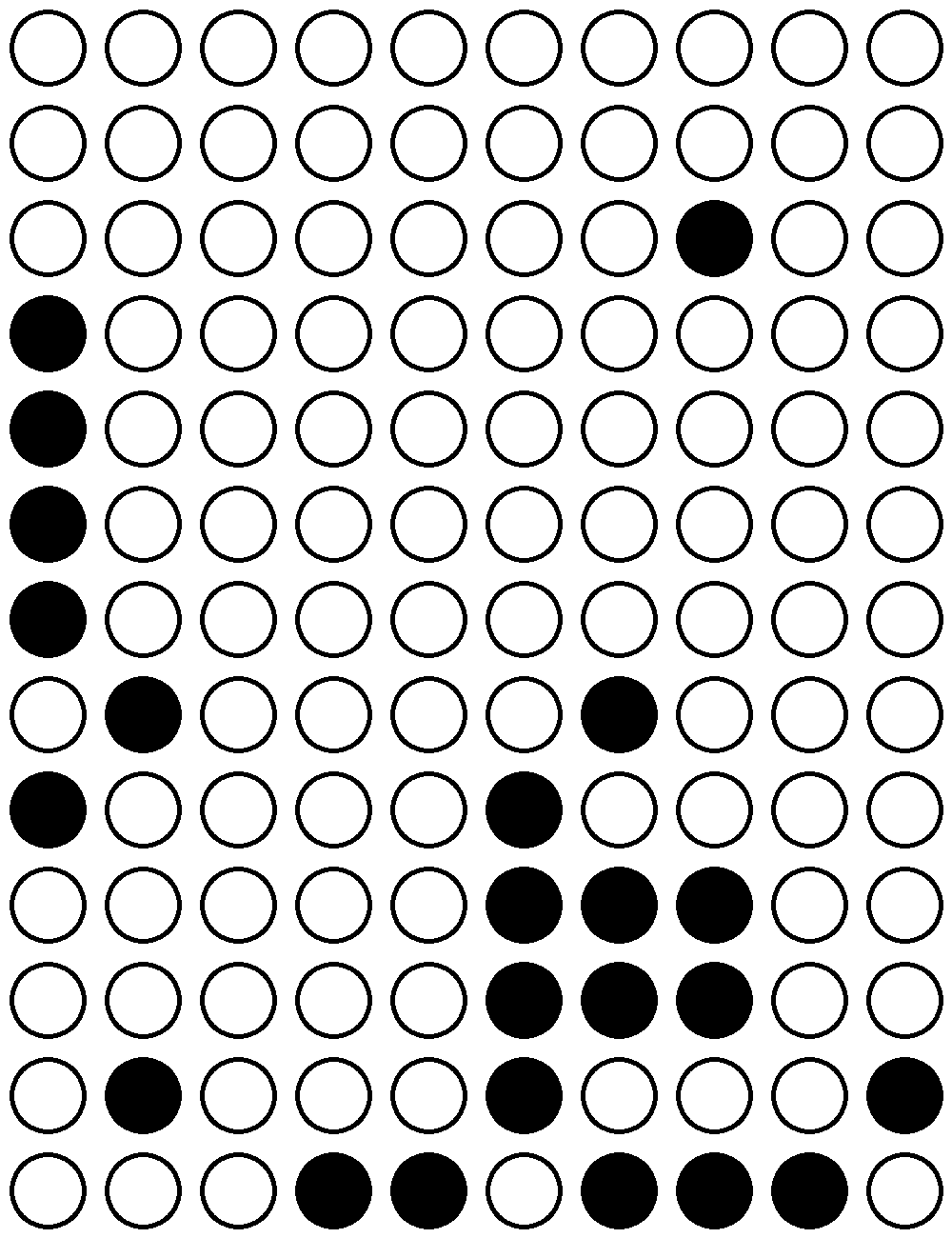


4.3% (14)

17.7% (13)

SI

RS

*Gata2-2*


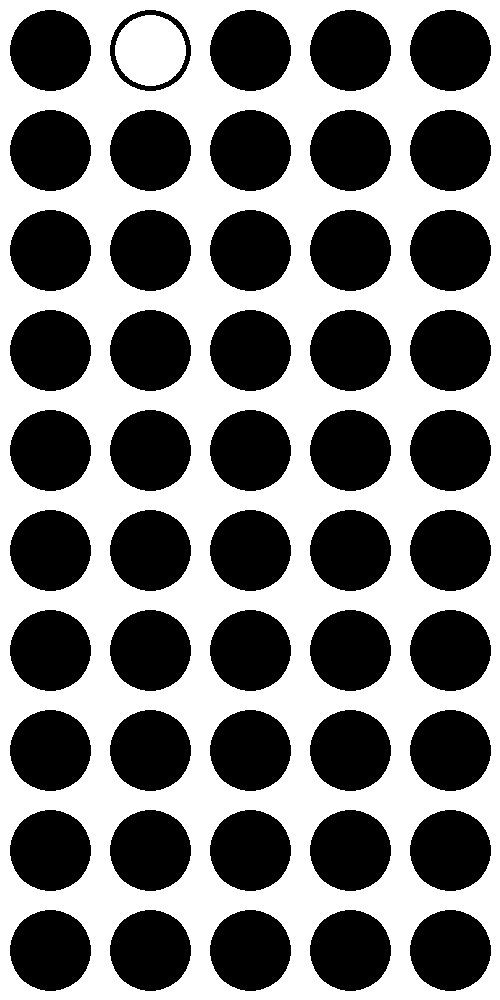

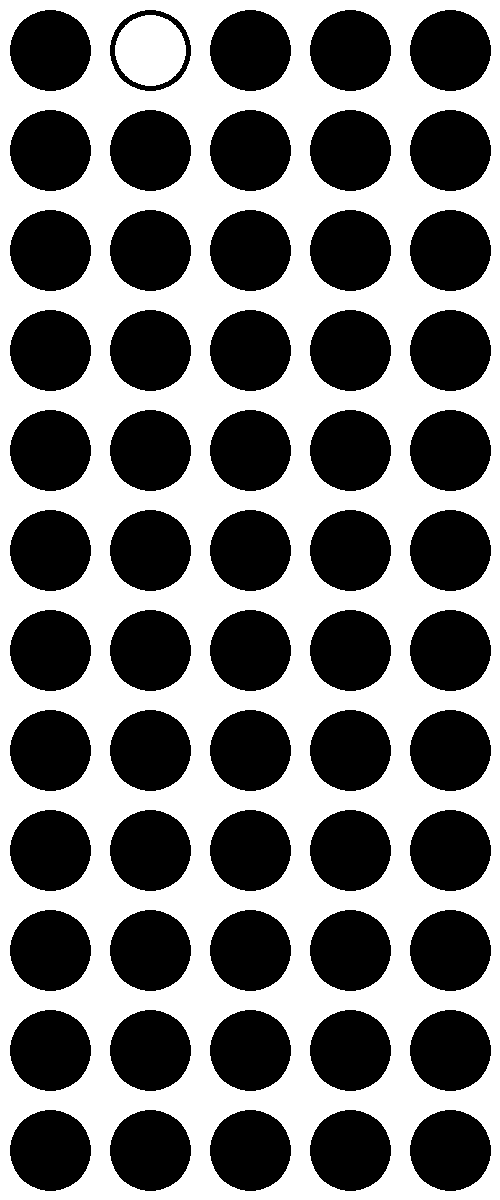


98.3% (12)

98% (10)


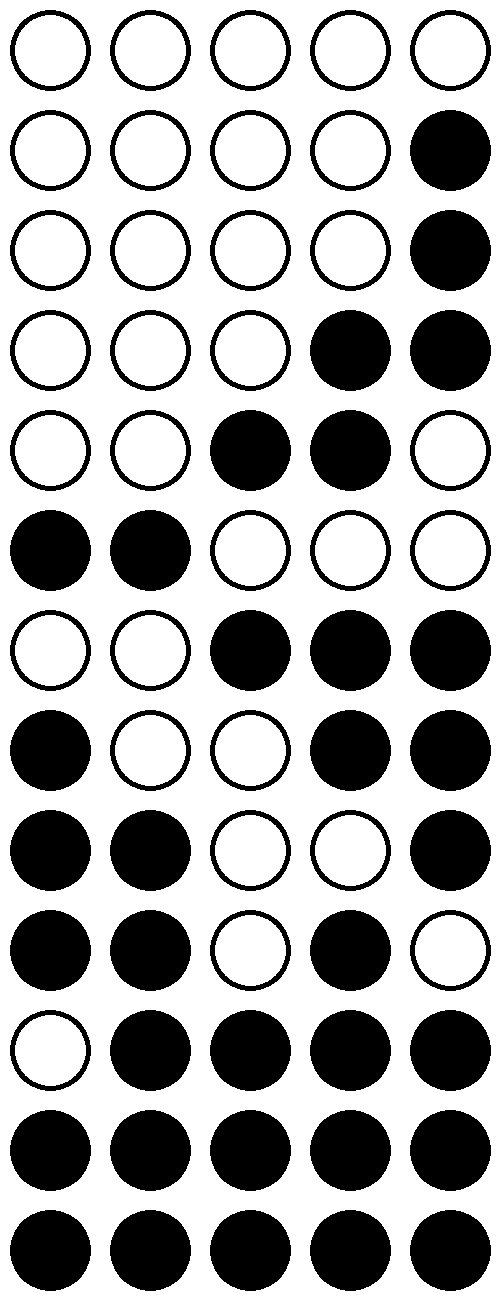

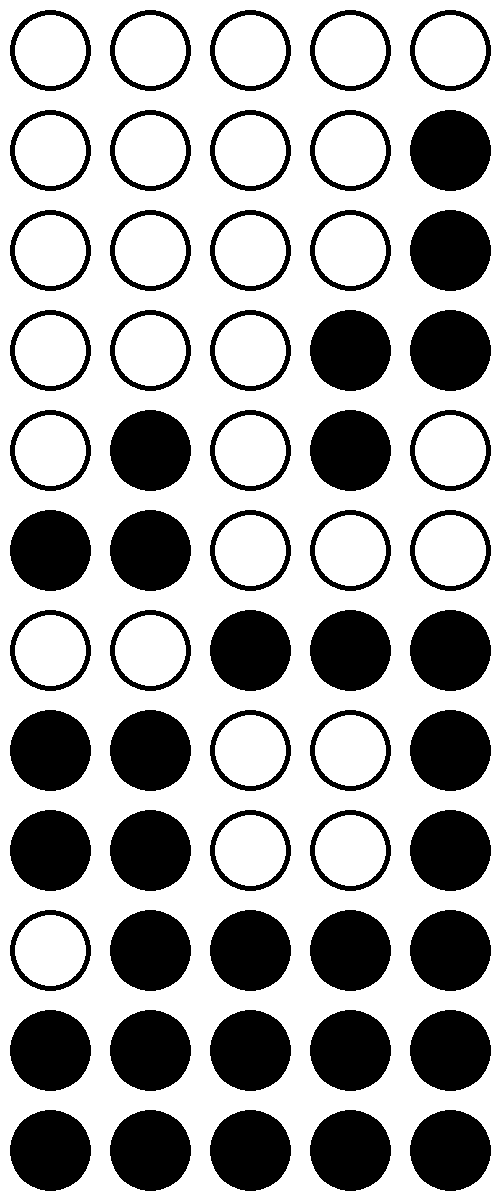


51.7% (12)

52.3% (13)

SI

RS

Not examined

Not examined

*Flt1*


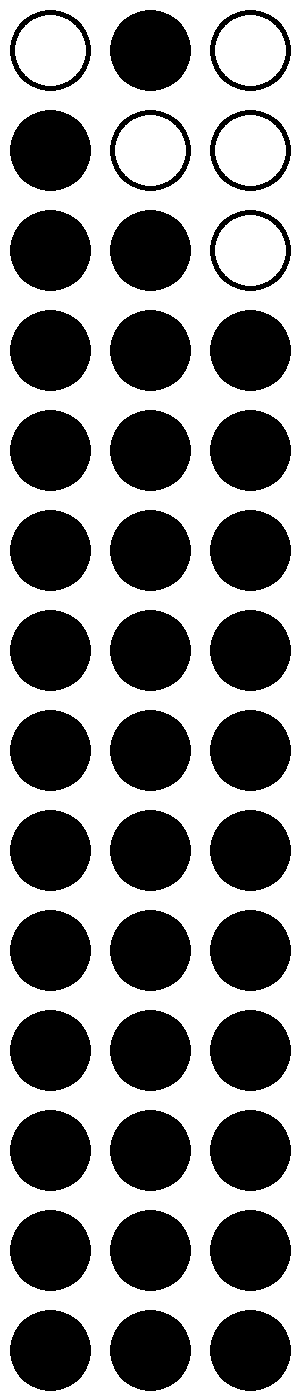

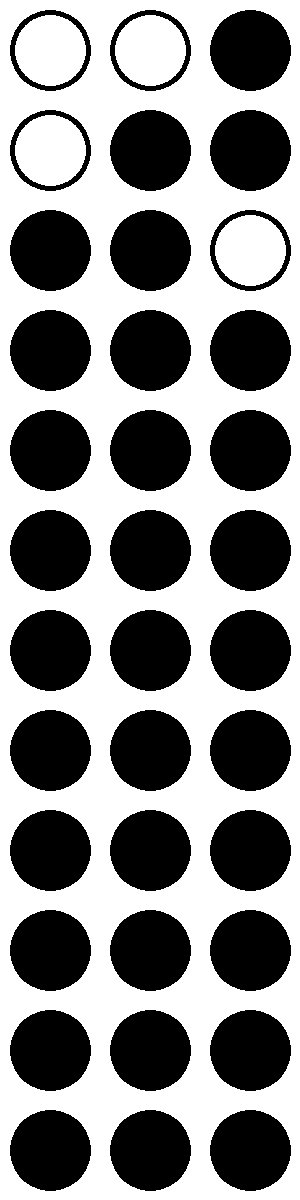


88.1% (14)

88.9% (12)


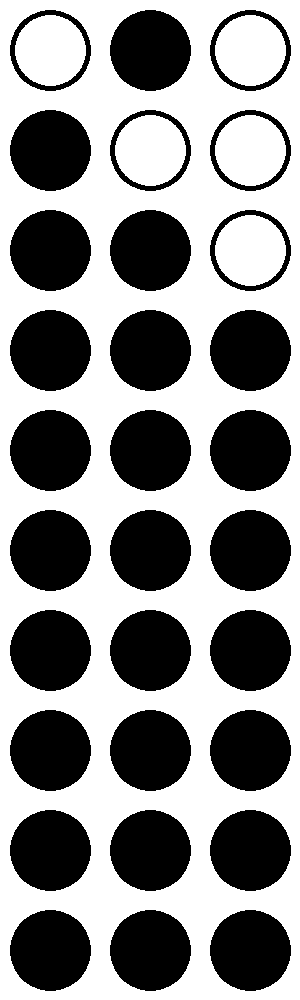


83.3% (10)

83.3% (10)


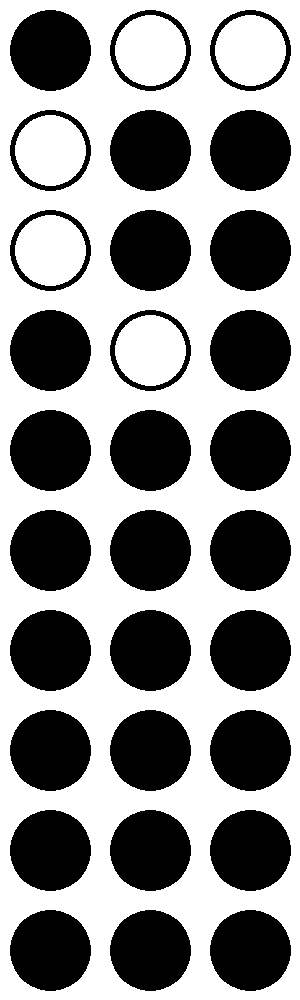


SI

RS

Not examined

Not examined

*Ddc*

*Dll4*

**Figure S1-3**


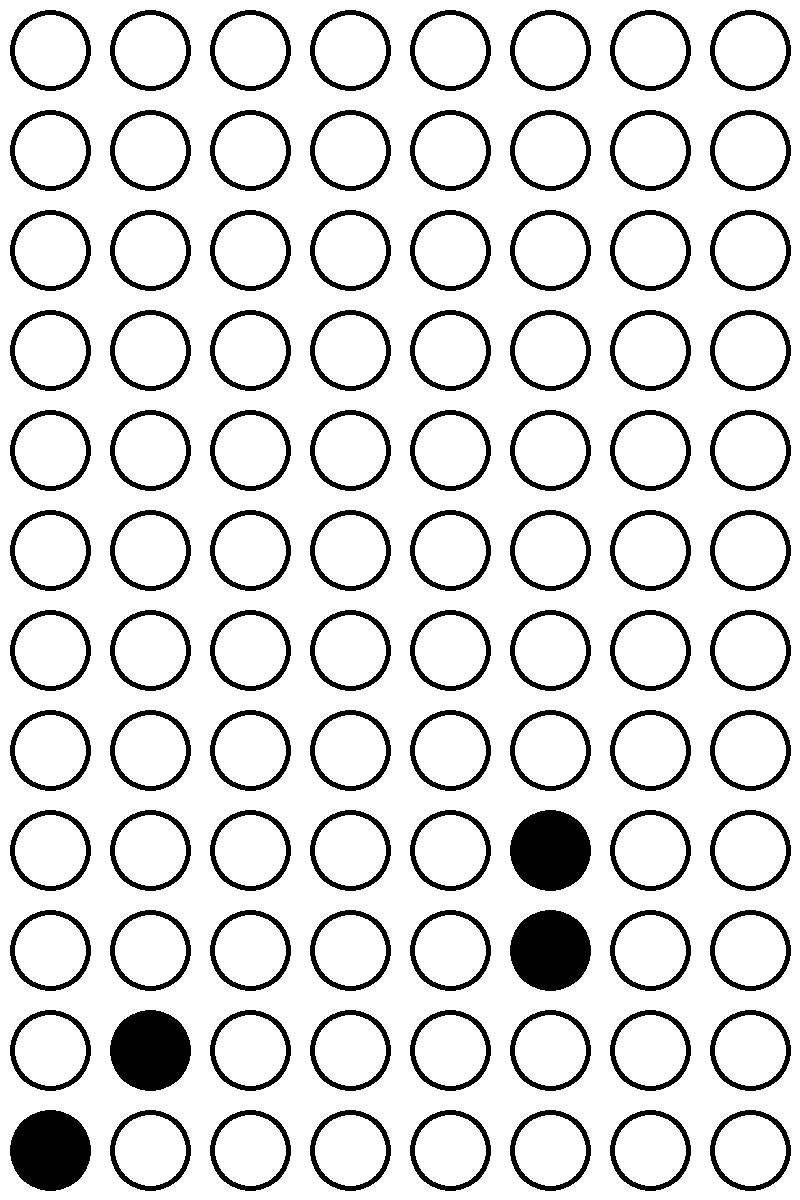


4.2% (12)


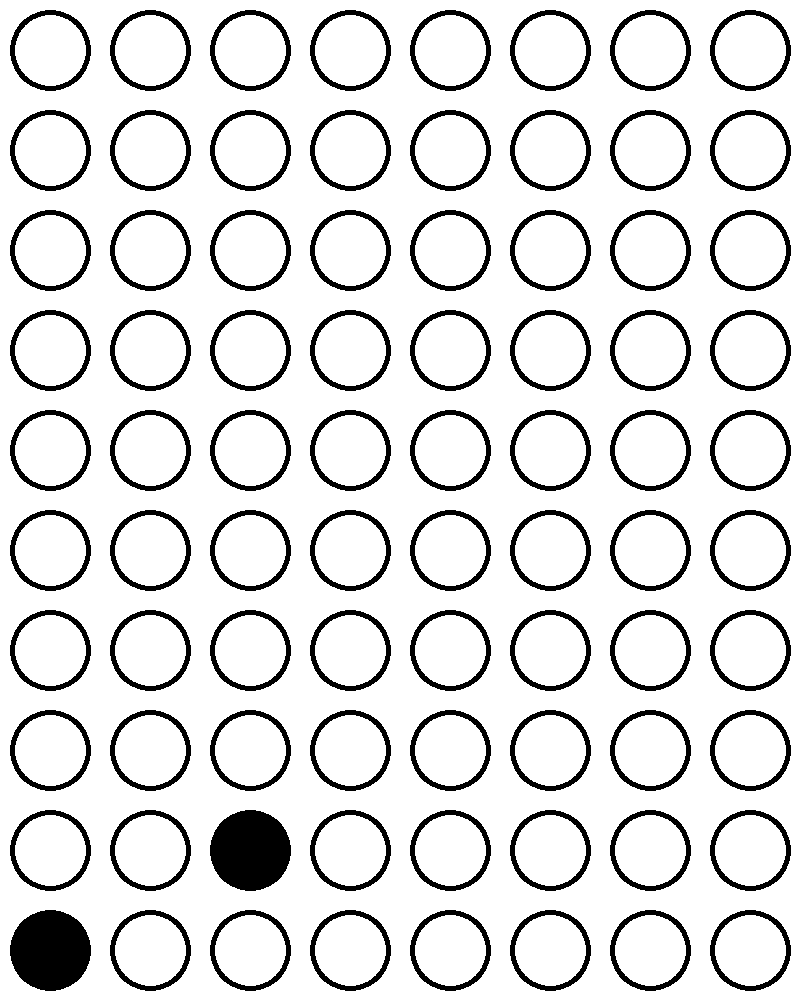


2.5% (10)


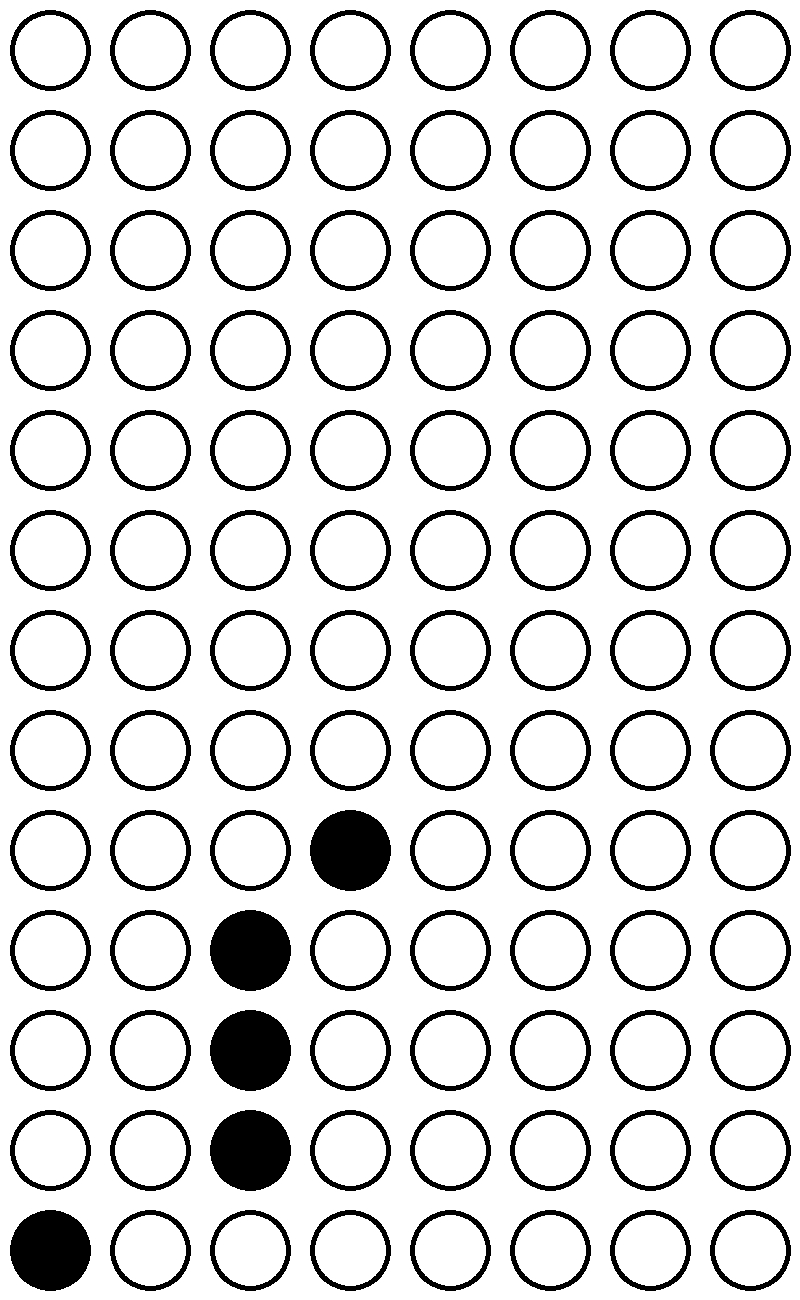


4.8% (13)


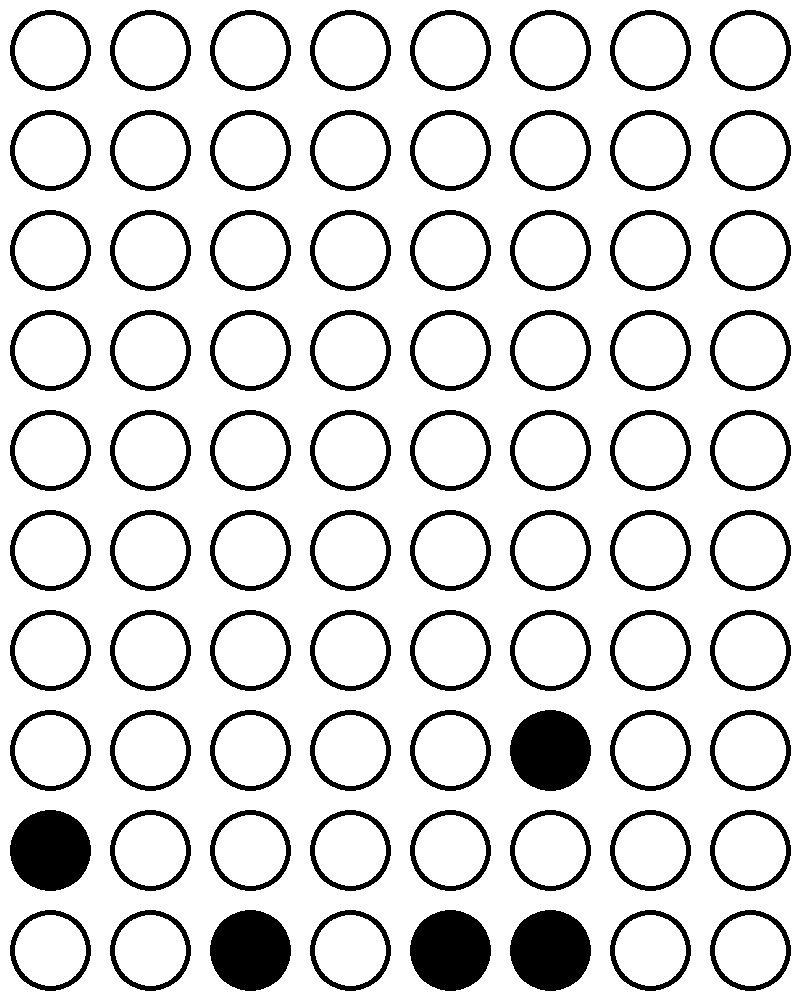


6.2% (10)

SI

RS

Not examined

Not examined

Spermatozoa

Blastocysts

Fetal hippocampi

Figure S1. Bisulfite sequencing of candidate AT genes in spermatozoa, E3.5 blastocysts, and fetal hippocampi from RS and SI mice. Genes observed are shown on the left side of the graphs. White circles indicate unmethylated CpGs, and black circles represent methylated CpGs. Values on each treatment indicate the percentage of CpG methylation, and the number in parentheses indicates clones analyzed.

**Supplementary figure S2**


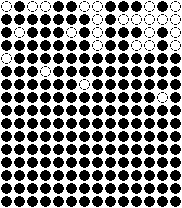


88.4% (16)

*Adora-1*


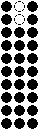


93.3% (10)

*Adora-2*


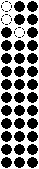


92.3% (13)

*Adcy-9*


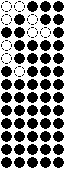


86.2% (13)

*Itpr3*


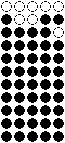


*Bdnf*

85.5% (11)


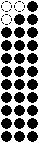


*Ddc*

90.9% (11)


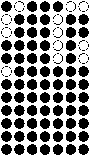


*Gata-1*

85.7% (12)


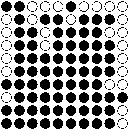


*Gata-2*

73% (10)


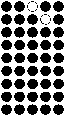


*Flt1*

95.6% (10)


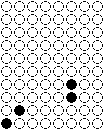


5% (10)

*Dll4*

**Figure S2**

Figure S2. Bisulfite sequencing of CAT genes in spermatozoa from unstressed control male mice. Genes observed are shown on the top of each graph. White circles indicate unmethylated CpGs, and black circles represent methylated CpGs. Values at the bottom of each graph indicate the percentage of CpG methylation, and the number in parentheses indicates clones analyzed.

**Supplementary figure S3**

**FC**

**FSI**

**MC**

**MSI**


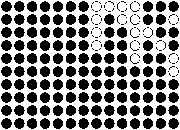


87.9% (10)


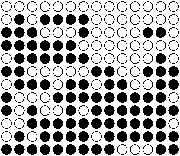


55.4% (12)

74.7% (11)


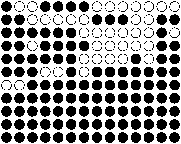


79.2% (11)


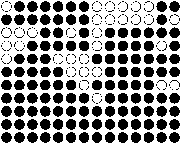


*Adora2a*-1


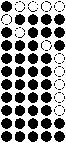


78.2% (11)

85% (12)


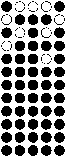


80% (10)


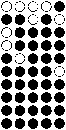


83.8% (14)


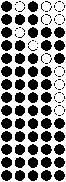


*Bdnf*


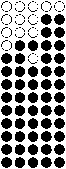


80% (13)

94% (10)


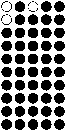


51.7% (10)


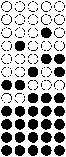


78% (10)


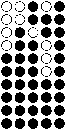


*Itpr3*


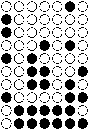


38.6% (10)

37.7% (11)


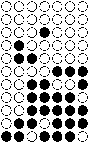


42.9% (10)


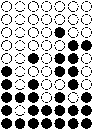


23.4% (11)


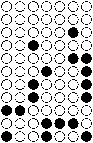


*Gata2-1*

**Figure S3**

Figure S3. Bisulfite sequencing of the 4 control sample-verified CAT genes in adult hippocampi between female control (FC) and SI (FSI) or male control (MC) and SI (MSI) offspring. Genes observed are shown on the left side of the graphs. White circles indicate unmethylated CpGs, and black circles represent methylated CpGs. Values on each treatment indicate the percentage of CpG methylation, and the number in parentheses indicates clones analyzed.
