# Supplementary material for: A next-generation sequencing study on mechanisms by which restraint and social instability stresses of male mice alter offspring anxiety-like behavior
Source: Sci Rep. 2021 Apr 12;11:7952. doi: 10.1038/s41598-021-87060-x (PMC8042048; doi:10.1038/s41598-021-87060-x)
Supplement: Supplementary file 3 — Supplementary Information 3. [file 41598_2021_87060_MOESM3_ESM.docx]

Title: A next-generation sequencing study on mechanisms by which restraint and social instability stresses of male mice alter offspring anxiety-like behavior

Qiao-Qiao Kong^1,2^, Xiao-Dan Tian^1^, Jia Wang^1^, Hong-Jie Yuan^1^, Shu-Fen Ning^1^, Ming-Jiu Luo^1^ and Jing-He Tan^1,3^

1. Shandong Provincial Key Laboratory of Animal Biotechnology and Disease Control and Prevention, College of Animal Science and Veterinary Medicine, Shandong Agricultural University, Tai'an City, P. R. China

2. Tai’an City Central Hospital, Tai’an City, P. R. China

3. Corresponding author: Jing-He Tan, College of Animal Science and Veterinary Medicine, Shandong Agricultural University, Tai-an City, Shandong Province, P R China, Post code: 271018, Phone: 0538-8249616, FAX: 0538-8241419, Email: [tanjh@sdau.edu.cn](mailto:tanjh@sdau.edu.cn)

Supplementary passages

**Supplementary passage 1**

Among the 8 KEGG pathways enriched by the 58 candidate genes, the complement and coagulation cascades (Abed et al., 2014), Rap1 signaling (Chen et al., 2005; Minato et al. 2007), neuroactive ligand-receptor interaction (Kong et al., 2014; Mitra et al., 2015), vascular smooth muscle contraction (Silver et al., 2012; Chistiakov et al., 2015), platelet activation (Zhang et al., 2017), Glycosaminoglycan biosynthesis- heparan sulfate / heparin (Chowers et al., 2001; Minge et al., 2017) and calcium signaling pathways (Erdmann et al., 2015; Tvrdik and Kalani, 2017) are involved in immune/inflammation reactions and/or brain development/disorders. Transcriptional misregulation can cause a broad range of diseases (Lee and Young, 2013).

**Supplementary passage 2**

All the top enriched genes in the 8 KEGG pathways enriched by the 58 candidate genes were involved in neural functions or disorders. Thus, Adcy9 might play a role in major depressive disorders (Toyota et al., 2002; Su et al., 2016). Adora2a was one of the anxiety-vulnerability genes (Domschke and Maron, 2013). Mice lacking Itpr3 showed abnormal behavioral and electrophysiological responses to sweet, umami and bitter substances that trigger G-protein-coupled receptor activation (Hisatsune et al., 2007). Blood von Willebrand factor (VWF) increased significantly in patients with severe phobic anxiety (Geiser et al., 2008). Inhibition of C5ar1 suppressed pathology and cognitive deficits in Alzheimer's disease mouse models (Hernandez et al., 2017). Furthermore, the Flt1 gene is involved in the regulation of hippocampal neurogenesis (During and Cao, 2006).

**Supplementary passage 3**

Most of the 16 terms enriched by our 58 candidate genes have direct or indirect relations with structure and function of blood vessels. The blood-brain barrier dysfunction may both lead to and be induced by aging, multiple sclerosis, Alzheimer’s and Parkinson’s diseases and epilepsy (Deo et al., 2013; Han et al., 2017). Furthermore, angiogenesis and inflammation are two highly linked processes (Scholz et al., 2015).

**Supplementary passage 4**

All the most frequently enriched genes have direct or indirect relations with brain neurogenesis and diseases. The involvement of Adora2a, Vwf, C5ar1 and Flt1 in brain neurogenesis and diseases has been discussed above. The Dll4 gene is involved in brain angiogenesis (Wälchli et al., 2015), and Dll4 of the Notch pathway promotes pro-inflammatory activation of macrophages in vitro and in vivo (Nakano et al., 2016); Gata2 overexpression interferes with spine formation and causes depression (Choi et al., 2014); Cdh5 has been found to be associated with autism (Redies et al., 2012); and the SSTR protein was markedly depleted in the Alzheimer’s brain (Burgos-Ramos et al., 2008).

**References**

Abed MA, Kloub MI, Moser DK. Anxiety and adverse health outcomes among cardiac patients: a biobehavioral model. J Cardiovasc Nurs 2014; 29: 354-363.

Burgos-Ramos E, Hervás-Aguilar A, Aguado-Llera D, Puebla-Jiménez L, Hernández-Pinto AM, Barrios V, Arilla-Ferreiro E. Somatostatin and Alzheimer's disease. Mol Cell Endocrinol 2008; 286: 104-111.

Chen Y, Wang PY, Ghosh A. Regulation of cortical dendrite development by Rap1 signaling. Mol Cell Neurosci 2005; 28: 215-228.

Chistiakov DA, Orekhov AN, Bobryshev YV. Vascular smooth muscle cell in atherosclerosis. Acta Physiol (Oxf) 2015; 214: 33-50.

Choi M, Wang SE, Ko SY, Kang HJ, Chae SY, Lee SH, Kim YS, Duman RS, Son H. Overexpression of human GATA-1 and GATA-2 interferes with spine formation and produces depressive behavior in rats. PLoS One 2014; 9: e109253.

Chowers Y, Lider O, Schor H, Barshack I, Tal R, Ariel A, Bar-Meir S, Cohen IR, Cahalon L. Disaccharides derived from heparin or heparan sulfate regulate IL-8 and IL-1 beta secretion by intestinal epithelial cells. Gastroenterology 2001; 120: 449-459.

Deo AK, Theil FP, Nicolas JM. Confounding parameters in preclinical assessment of blood-brain barrier permeation: an overview with emphasis on species differences and effect of disease states. Mol Pharm 2013; 10: 1581-195.

Domschke K, Maron E. Genetic factors in anxiety disorders. Mod Trends Pharmacopsychiatry 2013; 29: 24-46.

During MJ, Cao L. VEGF, a mediator of the effect of experience on hippocampal neurogenesis. Curr Alzheimer Res 2006; 3: 29-33.

Erdmann F, Kügler S, Blaesse P, Lange MD, Skryabin BV, Pape HC, Jüngling K. Neuronal expression of the human neuropeptide S receptor NPSR1 identifies NPS-induced calcium signaling pathways. PLoS One 2015; 10(2): e0117319.

Geiser F, Meier C, Wegener I, Imbierowicz K, Conrad R, Liedtke R, Oldenburg J, Harbrecht U. Association between anxiety and factors of coagulation and fibrinolysis. Psychother Psychosom 2008; 77: 377-383.

Han H, Mann A, Ekstein D, Eyal S. Breaking Bad: the Structure and Function of the Blood-Brain Barrier in Epilepsy. AAPS J 2017; 19: 973-988.

Hernandez MX, Jiang S, Cole TA, Chu SH, Fonseca MI, Fang MJ, Hohsfield LA, Torres MD, Green KN, Wetsel RA, Mortazavi A, Tenner AJ. Prevention of C5aR1 signaling delays microglial inflammatory polarization, favors clearance pathways and suppresses cognitive loss. Mol Neurodegener 2017; 12(1): 66.

Hisatsune C, Yasumatsu K, Takahashi-Iwanaga H, Ogawa N, Kuroda Y, Yoshida R, Ninomiya Y, Mikoshiba K. Abnormal taste perception in mice lacking the type 3 inositol 1,4,5-trisphosphate receptor. J Biol Chem 2007; 282: 37225-37231.

Kong SW, Sahin M, Collins CD, Wertz MH, Campbell MG, Leech JD, Krueger D, Bear MF, Kunkel LM, Kohane IS. Divergent dysregulation of gene expression in murine models of fragile X syndrome and tuberous sclerosis. Mol Autism 2014; 5: 16.

Lee TI, Young RA. Transcriptional regulation and its misregulation in disease. Cell 2013; 152: 1237-1251.

Minato N, Kometani K, Hattori M. Regulation of immune responses and hematopoiesis by the Rap1 signal. Adv Immunol 2007; 93: 229-264.

Minge D, Senkov O, Kaushik R, Herde MK, Tikhobrazova O, Wulff AB, Mironov A, van Kuppevelt TH, Oosterhof A, Kochlamazashvili G, Dityatev A, Henneberger C. Heparan Sulfates Support Pyramidal Cell Excitability, Synaptic Plasticity, and Context Discrimination. Cereb Cortex 2017; 27: 903-918.

Mitra A, Luo J, He Y, Gu Y, Zhang H, Zhao K, Cui K, Song J. Histone modifications induced by MDV infection at early cytolytic and latency phases. BMC Genomics 2015; 16: 311.

Nakano T, Fukuda D, Koga J, Aikawa M. Delta-Like Ligand 4-Notch Signaling in Macrophage Activation. Arterioscler Thromb Vasc Biol 2016; 36: 2038-2047.

Redies C, Hertel N, Hübner CA. Cadherins and neuropsychiatric disorders. Brain Res 2012; 1470: 130-144.

Scholz A, Plate KH, Reiss Y. Angiopoietin-2: a multifaceted cytokine that functions in both angiogenesis and inflammation. Ann N Y Acad Sci 2015; 1347: 45-51.

Silver M, Janousova E, Hua X, Thompson PM, Montana G; Alzheimer's Disease Neuroimaging Initiative. Identification of gene pathways implicated in Alzheimer's disease using longitudinal imaging phenotypes with sparse regression. Neuroimage 2012; 63: 1681-1694.

Su YA, Li JT, Dai WJ, Liao XM, Dong LC, Lu TL, Bousman C, Si TM. Genetic variation in the tryptophan hydroxylase 2 gene moderates depressive symptom trajectories and remission over 8 weeks of escitalopram treatment. Int Clin Psychopharmacol 2016; 31: 127-133.

Toyota T, Hattori E, Meerabux J, Yamada K, Saito K, Shibuya H, Nankai M, Yoshikawa T. Molecular analysis, mutation screening, and association study of adenylate cyclase type 9 gene (ADCY9) in mood disorders. Am J Med Genet 2002; 114: 84-92.

Tvrdik P, Kalani MYS. In Vivo Imaging of Microglial Calcium Signaling in Brain Inflammation and Injury. Int J Mol Sci 2017; 18(11). pii: E2366.

Wälchli T, Wacker A, Frei K, Regli L, Schwab ME, Hoerstrup SP, Gerhardt H, Engelhardt B. Wiring the Vascular Network with Neural Cues: A CNS Perspective. Neuron 2015; 87: 271-296.

Zhang Q, Zhang J, Yan Y, Zhang P, Zhang W, Xia R. Proinflammatory cytokines correlate with early exercise attenuating anxiety-like behavior after cerebral ischemia. Brain Behav 2017; 7: e00854.
