# Supplementary material for: A next-generation sequencing study on mechanisms by which restraint and social instability stresses of male mice alter offspring anxiety-like behavior
Source: Sci Rep. 2021 Apr 12;11:7952. doi: 10.1038/s41598-021-87060-x (PMC8042048; doi:10.1038/s41598-021-87060-x)
Supplement: Supplementary file 4 — Supplementary Information 4. [file 41598_2021_87060_MOESM4_ESM.docx]

Title: A next-generation sequencing study on mechanisms by which restraint and social instability stresses of male mice alter offspring anxiety-like behavior

Qiao-Qiao Kong^1,2^, Xiao-Dan Tian^1^, Jia Wang^1^, Hong-Jie Yuan^1^, Shu-Fen Ning^1^, Ming-Jiu Luo^1^ and Jing-He Tan^1,3^

1. Shandong Provincial Key Laboratory of Animal Biotechnology and Disease Control and Prevention, College of Animal Science and Veterinary Medicine, Shandong Agricultural University, Tai'an City, P. R. China

2. Tai’an City Central Hospital, Tai’an City, P. R. China

3. Corresponding author: Jing-He Tan, College of Animal Science and Veterinary Medicine, Shandong Agricultural University, Tai-an City, Shandong Province, P R China, Post code: 271018, Phone: 0538-8249616, FAX: 0538-8241419, Email: [tanjh@sdau.edu.cn](mailto:tanjh@sdau.edu.cn)

Table S1. Primers used for quantitative real-time PCR or bisulfite sequencing

| Genes | Primers for quantitative real-time PCR |  | Genes | Primers for DNA bisulfite sequencing |
| --- | --- | --- | --- | --- |
| HTR2A | F: CAATGTGTTTGTCTGGATTGGT |  | Adora-1 | IF: TGATTTTAGGGTAGTTTTTTTAAATTTA |
|  | R: CTGGAGCTGACTAGACTTGTAG |  |  | IR: AATCCAAACCAAACCCACTACT |
| Fkbp5 | F: GCTCCTTGATTTCAAAGGTGAG |  |  | EF: TTTTAGGGGTTTTAGTTTTTTTTGT |
|  | R: CTTCGTTTGGGTTTGAGTATCC |  |  | ER: AAACATCCTTACCCTTATAAACAAC |
| Htr1b | F: CCTTTGTAATCGCTACGGTGTA |  | Adora-2 | IF: TTAAGTATTGTAAAGGATGGATTGGA |
|  | R: GATGACACAGAGATGCATGATG |  |  | IR: ACCCCAACTAAACCCAAACTC |
| Adm | F: GAAAGAAGTGGAATAAGTGGGC |  |  | EF: GAAAGATGTGTTTATATGTAGGGGTT |
|  | R: GATCAAGAGTCTGGGTAGGAAC |  |  | ER: TCACAATCAACTCACAAAAAAAACTA |
| Nppc | F: TCAGAAAAAGGGTGACAAGACT |  | Ddc | IF: TTTTTTTAGGGTTTGGAGAAGTGTA |
|  | R: TTTGTATTTGCGCGCGTT |  |  | IR: TTTACAAAAAATACTTAAAACAAAACCTC |
| Agt | F: GGTCTCTTTCTACCTTGGATCC |  |  | EF: TGTAAAATTTATGTAGTGTAGTTGTTGT |
|  | R: GACCTTGTGTCCATCTAGTCG |  |  | ER: TATCATTTATTTAAAAATTATTTTCCACTA |
| Cck | F: ATGTCTGTGCGTGGTGATGGC |  | Adcy9 | IF: TTTTAAATTGAGTGTATGTGTTGGTTT |
|  | R: GCGGACCTGCTGGATGTATCG |  |  | IR: AAAACTACCCAAAATCACAAAAATC |
| Slc6a13 | F: GTACCACAACAATTGCTACAGG |  |  | EF: TGGAAATTTTTGTTAATGTATTTATTTTG |
|  | R: ACACACACAAACTGGCTATCTA |  |  | ER: AATTTTAAAACTTCTTATAAATTTATAA |
| Sstr4 | F: ATCTTCGTGATCCTACGCTATG |  | Itpr3 | IF: TTTTGTATAGTGGGATTTTGGGT |
|  | R: CTGAGCATGAAGAGCTCATCG |  |  | IR: TACATAATCAAATACAACAACACTC |
| Apod | F: GAAACTGCATTCAAGCCAACTA |  |  | EF: GTTGAGGATTATTTGGGGTA |
|  | R: TACTTGGTTCATGGTTCCATCA |  |  | ER: ACTAAAATATTTAAAAAACAACTACAAC |
| Itgal | F: AGTCAGCCTACATGGTTCTAAC |  | Gata-1 | IF: TTTTTTAGAGATTAGGTTTTTTTT |
|  | R: GTAAGTGTTCTGATCGCATGTC |  |  | IR: TTAAAATATATTCTTACTTTATCCTATATA |
| Pglyrp1 | F: CATTACCACAAGAATGAGCTGG |  |  | EF: GTATTTTAGGAAAAGTTTTTTTT |
|  | R: TGAAGGTGATGCCAATAGACAT |  |  | ER: AAAAACCCTAAAAAAATTAAAACCC |
| Adora2a | F: CATCAACTGCTTCACCTTCTTC |  | Gata-2 | IF: ATAGTAATTTAGGATGGTTATAGGATTAAA |
|  | R: GATCCTGTAGGCGTAGATGAAG |  |  | IR: CTCTTCTAACAACAAACTAAAAAAAA |
| Ddc | F: TATTTCTTCGCTTACTTCCCCA |  |  | EF: TTGGATTTTTAGGGGAATAGTG |
|  | R: GAGAAACCAATGCAGCCAATAG |  |  | ER: CTCTTCTAACAACAAACTAAAAAAAA |
| Adcy9 | F: GACTGGCTACTGCGGAACATCATC |  | Dll4 | IF: GATGTAGATAGAGAGGATTTGGAAGTTTA |
|  | R: CTTGGCGAACTCGAAGAGGATACG |  |  | IR: AAAATAAAAAATAAAATAAAATAAAAC |
| Itpr3 | F: CCTGTGCGTGTCCAACCGTATC |  |  | EF: AAGGAGAGAGAGGGAGTATTTTTTG |
|  | R: TTCTCGTGGTGCTCATTGTTCCTG |  |  | ER: ACTTAAATAATAATAATAAAAAATTAAAA |
| Gr | F: AGTCAAGGTTTCTGCGT |  | Flt1 | IF: GTTTTTGGTTATAGTTTTGTAAAGG |
|  | R: CCATCACTTTTGTTTCG |  |  | IR: CAACATATCTATTTCACTCAAAATACTAAT |
| Bdnf | F: GCCTCCTCTACTCTTTCTG |  |  | EF: TTTTAGGTTAGTTTTTTTAGTTAAAG |
|  | R: GGATTACACTTGGTCTCGT |  |  | ER: AAAAAATATTATACTCAAAATCATATACA |
| Gata2 | F: ACGCCTGTGGCCTCTACTAC |  | Bdnf | IF: TTAGTTTTTTTGGAATGTTTTATTT |
|  | R: GGATTTGCTGGACATCTTCC |  |  | IR: ATTATCCTCTAAAAACCATTTACCC |
|  |  |  |  | EF: TTTTAGGTTATAGTATTAATATGTGGT |
|  |  |  |  | ER: CTCCAACTAACTTTCAAAATCAAATAAA |

F: Forward; R: Reverse; IF: Internal forward; IR: Internal reverse; EF: External forward; ER: External reverse; Adora-1 and -2: Adora2a-DMR1 and -DMR2; Gata-1 and -2: Gata2-DMR1 and -DMR2.

Table S2. Numbers and litters of F1 offspring used for EPM tests and the difference between treatments.

| F1 offspring | Treatment | Litter numbers | Mouse numbers* | Open arm time (s) | Closed arm time (s) |
| --- | --- | --- | --- | --- | --- |
| Female | SI | 10 | 44 | 28.7±4.3^a^ | 198.4±8.7^a^ |
|  | Ctrl | 14 | 39 | 48.3±4.3^b^ | 170.2±7.9^b^ |
|  | RS | 11 | 39 | 62.1±4.4^c^ | 143.4±8.4^c^ |
| Male | SI | 9 | 35 | 41.7±8.4^a^ | 175.2±10.4^a^ |
|  | Ctrl | 14 | 54 | 37.7±4.5^a^ | 173.3±7.2^a^ |
|  | RS | 8 | 30 | 65.9±4.7^b^ | 150.3±7.5^b^ |

* Two to five female or male mice were used from each litter. a-c: Values with a different superscript differ significantly (P<0.05) within offspring sexes in the same column.

Table S3. Numbers and litters of F1 offspring used for OFT tests and the difference between treatments.

| F1 offspring | Treatment | Litter numbers | Mouse numbers* | Central time (s) | Central distance / total distance (%) |
| --- | --- | --- | --- | --- | --- |
| Female | SI | 10 | 30 | 38.9±4.9^a^ | 18.2±2.6^a^ |
|  | Control | 12 | 35 | 58.9±4.5^b^ | 23.0±2.1^b^ |
|  | RS | 11 | 24 | 101.2±5.2^c^ | 29.5±2.4^c^ |
| Male | SI | 9 | 28 | 39.7±9.4^a^ | 22.6±1.6^a^ |
|  | Control | 14 | 33 | 45.9±5.7^a^ | 22.2±1.6^a^ |
|  | RS | 8 | 27 | 90.3±4.9^b^ | 26.5±1.2^b^ |

* Two to five female or male mice were used from each litter. a-c: Values with a different superscript differ significantly (P<0.05) within offspring sexes in the same column.

Table S4. Numbers and litters of F1 offspring used for qRT-PCR and the difference between treatments.

| F1 offspring | Treatment | Mice examined* | Relative Gr mRNA | Relative Bdnf mRNA |
| --- | --- | --- | --- | --- |
| Female | SI | 13 | 0.57±0.05^a^ | 0.69±0.08^a^ |
|  | Control | 12 | 1.00±0.00^b^ | 1.00±0.00^b^ |
|  | RS | 15 | 2.33±0.33^c^ | 1.83±0.16^c^ |
| Male | SI | 16 | 0.86±0.05^a^ | 1.02±0.05^a^ |
|  | Control | 15 | 1.00±0.00^a^ | 1.00±0.00^a^ |
|  | RS | 15 | 1.51±0.08^b^ | 1.53±0.06^b^ |

* Each mouse from a different litter. a-c: Values with a different superscript differ significantly (P<0.05) within offspring sexes in the same column.

Table S5. Fold change of DE, and methylation difference and gene elements of DMRs in the 58 candidate genes

| **Gene name** | Fold change | Methylation difference | DMR gene elements | **Gene name** | Fold change | Methylation difference | DMR gene elements |
| --- | --- | --- | --- | --- | --- | --- | --- |
| Gm45844 | 1.60 ↑ | 0.42 ↑ | Exon, UTR5, Exon, Promoter | Gse1 | 0.61 ↓ | -0.42 ↓ | Intron |
| Lmcd1 | 1.52 ↑ | -0.36 ↓ | Intron |  |  | 0.47 ↑ | Intron |
| Sstr4 | 1.59 ↑ | -0.78 ↓ | Exon, UTR3 |  |  | -0.68 ↓ | Intron |
| Xkr6 | 1.57 ↑ | 0.54 ↑ | Intron |  |  | 0.51 ↑ | Intron |
|  |  | 0.34 ↑ | Intron |  |  | 0.32 ↑ | Intron |
|  |  | 0.44 ↑ | Intron |  |  | 0.41 ↑ | Intron |
| Adora2a | 1.94 ↑ | -0.43 ↓ | Intron, Promoter |  |  | -0.49 ↓ | Intron |
|  |  | -0.38 ↓ | Intron, Promoter, Exon, UTR5 |  |  | 0.67 ↑ | Intron |
| Rnf26 | 3.55 ↑ | -0.33 ↓ | Intron |  |  | -0.65 ↓ | Intron |
| Hspb3 | 3.16 ↑ | 0.32 ↑ | Exon |  |  | -0.57 ↓ | Intron |
| Pcdhga3 | 0.63 ↓ | -0.37 ↓ | Intron |  |  | 0.50 ↑ | Intron |
| Pcdhga6 | 0.57 ↓ | -0.37 ↓ | Exon | Tenm3 | 0.66 ↓ | 0.54 ↑ | Intron |
| Pcdhga4 | 0.59 ↓ | -0.37 ↓ | Intron |  |  | 0.42 ↑ | Intron |
| Pcdhga5 | 0.64 ↓ | -0.37 ↓ | Intron | Aff2 | 0.57 ↓ | 0.48 ↑ | Intron |
| Pcdhga2 | 0.53 ↓ | -0.37 ↓ | Intron | Slc6a13 | 0.61 ↓ | -0.46 ↓ | Intron |
| Pcdhga1 | 0.55 ↓ | -0.37 ↓ | Intron | Map3k6 | 0.54 ↓ | 0.47 ↑ | Promoter |
| Gm1604a | 0.52 ↓ | 0.75 ↑ | Intron | Dll4 | 0.51 ↓ | 0.51 ↑ | Promoter |
| Palm2 | 0.53 ↓ | 0.37 ↑ | Intron | F5 | 0.66 ↓ | 0.46 ↑ | Intron |
| Trabd2b | 0.53 ↓ | 0.64 ↑ | Intron | Vash1 | 0.62 ↓ | 0.39 ↑ | Intron |
| Zbtb16 | 0.59 ↓ | 0.43 ↑ | Intron | Ddc | 0.54 ↓ | -0.28 ↓ | Intron |
|  |  | 0.45 ↑ | Intron | Gata2 | 0.56 ↓ | 0.53 ↑ | Promoter, Exon, Intron |
|  |  | 0.36 ↑ | Intron | Piezo1 | 0.58 ↓ | 0.52 ↑ | Intron |
| Adamts17 | 0.67 ↓ | -0.35 ↓ | Intron | Adcy9 | 0.62 ↓ | 0.52 ↑ | Intron |
| Soga1 | 0.59 ↓ | -0.39 ↓ | Exon, Intron | Vwf | 0.61 ↓ | -0.39 ↓ | Exon |
| Hs6st3 | 0.58 ↓ | 0.40 ↑ | Intron | Hk2 | 0.56 ↓ | -0.49 ↓ | Exon, Intron |
| Hs3st3a1 | 0.54 ↓ | 0.45 ↑ | Exon |  |  | 0.49 ↑ | Intron |
| Itpr3 | 0.57 ↓ | 0.35 ↑ | Intron | Adamts14 | 0.42 ↓ | -0.50 ↓ | Intron |
| Sun2 | 0.65 ↓ | 0.40 ↑ | Intron |  |  | 0.48 ↑ | Promoter |
| Sdk1 | 0.61 ↓ | 0.51 ↑ | Intron | C5ar1 | 0.37 ↓ | -0.32 ↓ | Intron |
| Fstl4 | 0.66 ↓ | 0.68 ↑ | Intron | Wfikkn2 | 0.42 ↓ | -0.40 ↓ | Promoter, Intron |
|  |  | 0.30 ↑ | Intron | Erg | 0.35 ↓ | -0.49 ↓ | Intron |
| Filip1 | 0.67 ↓ | 0.46 ↑ | Intron | Itgal | 0.47 ↓ | 0.46 ↑ | Intron |
| Plvap | 0.64 ↓ | 0.59 ↑ | Exon, UTR3 | Pglyrp1 | 0.41 ↓ | 0.50 ↑ | Intron, Exon, UTR5 |
| Dgkh | 0.57 ↓ | -0.43 ↓ | Intron | Flt1 | 0.47 ↓ | 0.57 ↑ | Intron |
| Cmip | 0.66 ↓ | 0.62 ↑ | Intron |  |  | -0.57 ↓ | Intron |
|  |  | 0.55 ↑ | Intron |  |  | -0.36 ↓ | Intron |
|  |  | 0.64 ↑ | Intron | Gpr35 | 0.18 ↓ | -0.49 ↓ | Intron |
|  |  | -0.44 ↓ | Intron | Apod | 0.47 ↓ | -0.52 ↓ | Promoter |
|  |  | 0.60 ↑ | Intron, Promoter | Sema3g | 0.44 ↓ | 0.24 ↑ | Exon, Intron |
| Dysf | 0.60 ↓ | -0.40 ↓ | Intron | Cd244 | 0.05 ↓ | -0.51 ↓ | Intron |
| Lama1 | 0.64 ↓ | 0.41 ↑ | Intron |  |  |  |  |
| Thsd4 | 0.62 ↓ | -0.53 ↓ | Intron |  |  |  |  |
|  |  | 0.48 ↑ | Intron |  |  |  |  |
|  |  | -0.58 ↓ | Intron |  |  |  |  |
| Cdh5 | 0.63 ↓ | -0.38 ↓ | Promoter |  |  |  |  |
|  |  | 0.53 ↑ | Promoter |  |  |  |  |

Fold change: Values of RS samples divided by values of SI samples that were set as 1; Methylation difference: % Methylation in RS samples minus that in SI samples; ↑ and ↓: Higher and lower methylation levels, respectively.
